# Evolution of sex-dependent mtDNA transmission in freshwater mussels (Bivalvia: Unionida)

**DOI:** 10.1038/s41598-017-01708-1

**Published:** 2017-05-08

**Authors:** Davide Guerra, Federico Plazzi, Donald T. Stewart, Arthur E. Bogan, Walter R. Hoeh, Sophie Breton

**Affiliations:** 1 Université de Montréal, Département de Sciences Biologiques, Montréal, H2V 2S9 Quebéc Canada; 2 Università di Bologna, Dipartimento di Scienze Biologiche, Geologiche ed Ambientali (BiGeA), Bologna, 40126 Italy; 3 Acadia University, Department of Biology, Wolfville, B4P 2R6 Nova Scotia Canada; 4 North Carolina Museum of Natural Sciences, Raleigh, 27607 NC USA; 5 Kent State University, Department of Biological Sciences, Kent, 44242 OH USA

## Abstract

Doubly uniparental inheritance (DUI) describes a mode of mtDNA transmission widespread in gonochoric freshwater mussels (Bivalvia: Palaeoheterodonta: Unionida). In this system, both female- and male-transmitted mtDNAs, named F and M respectively, coexist in the same species. In unionids, DUI is strictly correlated to gonochorism and to the presence of the atypical open reading frames (ORFans) F-*orf* and M-*orf*, *r*espectively inside F and M mtDNAs, which are hypothesized to participate in sex determination. However, DUI is not found in all three Unionida superfamilies (confirmed in Hyrioidea and Unionoidea but not in Etherioidea), raising the question of its origin in these bivalves. To reconstruct the co-evolution of DUI and of ORFans, we sequenced the mtDNAs of four unionids (two gonochoric with DUI, one gonochoric and one hermaphroditic without DUI) and of the related gonochoric species *Neotrigonia margaritacea* (Palaeoheterodonta: Trigoniida). Our analyses suggest that rearranged mtDNAs appeared early during unionid radiation, and that a duplicated and diverged *atp8* gene evolved into the M-*orf* associated with the paternal transmission route in Hyrioidea and Unionoidea, but not in Etherioidea. We propose that novel mtDNA-encoded genes can deeply influence bivalve sex determining systems and the evolution of the mitogenomes in which they occur.

## Introduction

Bivalves are the only known group of animals in which both sexes are capable of stably transmitting their mitochondrial (mt) genome to their progeny through doubly uniparental inheritance (DUI)^1^. In DUI species, some embryos retain sperm mitochondria that carry a male-transmitted or M-type mtDNA, actively segregate them in their germ line during early development and grow as males, producing M-homoplasmic sperm as adults. By contrast, other embryos eliminate the M mtDNA, or highly reduce its copy number during early development, segregate only the female-transmitted mitochondria with the F-type mtDNA in their germ line and develop as females, in a developmental path comparable to that of other animal species^1^. The F and M mtDNAs can be extremely divergent in nucleotide (nt) sequence (up to ~40%^2^), and can also have different gene order and content. In particular, they contain additional lineage-specific open reading frames (ORFs) without recognizable homologies to known genes (hereafter called ORFans^3^). These F- and M-specific ORFans have been respectively called F-*orf* and M-*orf* ^4–9^. Another particular feature often found in DUI species are modifications to the *cox2* gene: for example, all DUI species of freshwater mussels (Palaeoheterodonta: Unionida) possess a 3′-elongated *cox2* gene in their M mtDNA^2, 10^.

DUI and the ORFans carried by the two mtDNAs have been hypothesized to be part of a sex determination system involving mitochondria, in part because bivalves lack heteromorphic sex chromosomes in their nuclear genomes and also because freshwater mussels with DUI that switched from the ancestral gonochoric reproductive mode to a derived hermaphroditism lost the M mt genome in the process^6^. The mtDNA retained in these hermaphrodites, named H, is derived from an ancestral F mitogenome and its F-*orf* is highly modified into a so-called H-*orf* ^6^. The predicted functions of the proteins encoded by the F-*orf* and M-*orf* ORFans (we will use ‘F-*ORF*’ and ‘M-*ORF*’ to refer to these proteins) support their direct involvement in the DUI mechanism: for example, F-*ORF*s are suggested to interact with nucleic acids, adhere to membranes, and have roles in signalling, and M-*ORF*s are suggested to interact with the cytoskeleton and take part in ubiquitination and apoptosis processes^8, 11, 12^. However, the precise nature of the link between DUI (and M and F ORFans) and the maintenance of gonochorism remains unknown.

To date, DUI has been detected in >100 gonochoric bivalve species, with a somewhat scattered phylogenetic distribution^13^. The complexity of this system led to the hypothesis that it evolved once in a basal, ancestral lineage of bivalves, followed by subsequent losses in the common ancestor to some modern taxa^1, 14, 15^. With a single origin of DUI and with no recombination between the two mitogenomes, all sequences of the F and M lineages should be reciprocally monophyletic, in a pattern named ‘gender-joining’^15^. Up to now however, the gender-joining clustering has been consistently observed only in species of the order Unionida (e.g., ref. 13), suggesting that DUI was present in their common ancestor. In other orders of bivalves, the F and M mtDNAs of a single species are frequently distinct from one another yet they cluster together in a ‘taxon-joining’ pattern^15^. This phylogenetic pattern may be due to ‘masculinization’ or ‘role reversal events’, a phenomenon directly demonstrated only in *Mytilus*. Masculinization involves an F mtDNA that invades the paternal route of inheritance and unseats the old M, thus becoming a new M (reviewed in ref. 16). An alternative hypothesis could be independent origins of DUI. For example, studies on F-*ORF* and M-*ORF* proteins suggested that ORFans might have various origins (viral or mitochondrial), and that their fixation might have triggered multiple rises of DUI in bivalves^8, 11, 12, 17^.

The Palaeoheterodonta is one of the most ancient lineages of Bivalves (over 470 million years; ref. 18 and references therein). It is currently represented by two orders, Trigoniida and Unionida (taxonomy from ref. 19). The order Trigoniida is constituted by only a few living species of marine clams, all belonging to the genus *Neotrigonia*, whereas the Unionida is composed of six families of freshwater mussels^19^: Iridinidae, Etheriidae, and Mycetopodidae (superfamily Etherioidea); Hyriidae (superfamily Hyrioidea^20^); and Margaritiferidae and Unionidae (superfamily Unionoidea). No general agreement about the phylogenetic relationships among these six families has been reached yet^20^. However, the occurrence of DUI in Palaeoheterodonta has been explored by ref. 14. Their results indicated that DUI is absent from the superfamily Etherioidea, and this observation led them to ask (1) whether DUI is the basal condition of Unionida that was lost in the Etherioidea lineage, or (2) if DUI was gained only in the ancestor(s) to the Unionoidea and Hyrioidea lineages^14^. The authors also tested for the presence of the M type mtDNA in the trigoniid *Neotrigonia margaritacea*, but the negative results left uncertain the presence of DUI in the order Trigoniida^14^. An update on this question was provided by ref. 21, who reported the presence of two sex-associated mitotypes with extremely low divergence from one another in *N*. *margaritacea*. The sequences obtained, however, are not publicly available at the time of writing this article, and the low divergence found calls for more in-depth studies to confirm the presence of DUI in *N*. *margaritacea*.

To reconstruct the evolution of DUI in the ancient bivalve clade Palaeoheterodonta and to better understand the link between sex determination and ORFans in F and M mtDNAs, we sequenced seven new mt genomes from five palaeoheterodont species, with or without DUI and with different reproductive strategies. For Trigoniida we obtained the mt genome, presumably the F, of *N*. *margaritacea* (gonochoric, DUI status uncertain), while for Unionida we sequenced the mtDNAs of the following species: *Mutela dubia* (Iridinidae) (gonochoric without evidence of DUI^14, 22^), *Anodontites trapesialis* (Mycetopodidae) (hermaphroditic^23^ without evidence of DUI; Hoeh W.R. personal communication), and the M and F genomes of *Hyridella menziesii* (Hyriidae) and *Cumberlandia monodonta* (Margaritiferidae) (both gonochoric with DUI^14^). These new sequences allowed us to reconstruct a robust phylogeny of Palaeoheterodonta and infer the origin and evolution of DUI and the ORFans in Unionida.

## Results

### Overview of mitochondrial genomes

**Figure 1:**
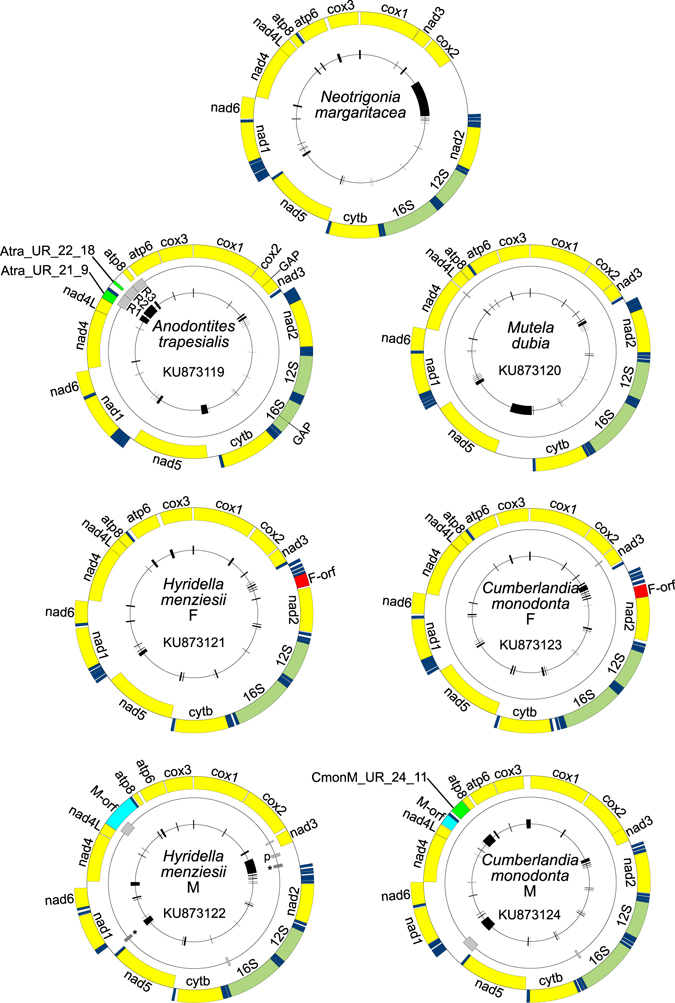
Maps of the seven mt genomes sequenced in this study. The corresponding GenBank accession numbers are given inside each genome map. Genomes are not in scale among each other, see main text for their length. Outer ring comprises all standard and putative coding sequences, identified with the following colour code: yellow, genes encoding electron transport chain and ATP-synthase subunits; dark blue, tRNA genes (see Supplementary Fig. S1 in Supplementary Information 1 for their names); pale green, rRNA genes; red, F-*orf*; bright blue, M-*orf*; bright green, additional ORFs cited in the text. Sequences are located on the outer or inner side of this circle according to their coding direction, respectively forward (clockwise) and reverse (anti-clockwise). In *A*. *trapesialis* mtDNA, the position of the two sequencing gaps is indicated on this ring. Middle ring comprises the repetitive regions, indicated in grey (not found in *N*. *margaritacea* and *H*. *menziesii* F): in *A*. *trapesialis* are specified the names R1–3 for the large tandem repeats found between *nad4L* and *atp6*; in *H*. *menziesii* M, two regions of high similarity found in different locations are marked with an asterisk (*), and a large palindrome sequence with “p”. Alignments of all repetitive regions can be found in Supplementary Information 2. Innermost ring displays the position of the unassigned regions as black segments.

Complete mtDNA sequences have been obtained for *N*. *margaritacea* (16,739 bp; putatively the F, if this species has DUI), *H*. *menziesii* (F: 16,031 bp; M: 18,140 bp), *C*. *monodonta* (F: 16,099 bp; M: 17,575 bp), and *M*. *dubia* (non-DUI mtDNA: 16,168 bp). A nearly complete mtDNA sequence was retrieved for *A*. *trapesialis* (non-DUI mtDNA: 15,117 bp), as we were not able to sequence two small segments between *cox2* and *nad2* and within the 16S rDNA. All sequences have been deposited in GenBank (accession numbers KU873118-KU873124; see Table 1). The structures of the genomes are shown in Fig. 1. All mtDNAs possess the standard set of 37 genes. *H*. *menziesii* and *C*. *monodonta* possess the additional unionid F-*orf* and M-*orf* in their F and M genomes, respectively, and the 3′-elongated *cox2* gene in their M mtDNAs (1,380 bp in *H*. *menziesii* and 1,269 bp in *C*. *monodonta*). The *cox2* genes of *N*. *margaritacea*, *M*. *dubia* and *A*. *trapesialis* do not have a 3′ elongated region. tRNA-Glu is duplicated in *C*. *monodonta* F mtDNA (see Supplementary Fig. S1 in Supplementary Information 1). The gene order of the seven mt genomes is largely similar, with the main difference being an inversion of *cox2* and *nad3* between *N*. *margaritacea* and unionids (Fig. 1 and Supplementary Fig. S1). Supplementary Information 1 contains a comparison of nucleotide content among the seven new mtDNAs and those of the freshwater mussel species listed in Table 1 (Supplementary Table S1 and Supplementary Fig. S2), as well as codon usage descriptions (Supplementary Tables S2 and S3, Supplementary Fig. S3). Repeated sequences were found in all genomes, except for *N*. *margaritacea* and *H*. *menziesii* F mtDNAs (Fig. 1). The two most notable features are (1) three large tandem repeats in *A*. *trapesialis* (331–310 bp, between *nad4L* and the end of *nad6*) that comprise pseudogenized fragments of tRNA-Asp, *atp8*, and *nad6*, and (2) a complex, ~380 bp-long repeat region in the 3′ half of *H*. *menziesii* M-*orf*. Alignments of these repeats and several other minor features are shown in Supplementary Information 2. For *H*. *menziesii* and *C*. *monodonta* mtDNAs, the overall intraspecific p-distance values between F- and M-encoded protein coding genes (PCGs) (~40%) and their proteins (48–49%) and the overall *d*_N_/*d*_S_ values between F and M genes (*H*. *menziesii*: ~0.33; *C*. *monodonta*: ~0.40), are comparable with previous observations on other DUI freshwater mussels, and equivalent values are found also for the interspecific comparisons (Supplementary Tables S4 and S5 in Supplementary Information 3) (see ref. 2 for a comparison).

**Table 1:** List of species and respective mtDNAs considered in this study.

Class	Subclass	Order	Superfamily	Family	Subfamily	Species	Sex determination	DUI	mtDNA type	Accession Number
Bivalvia	Palaeoheterodonta	Trigoniida	Trigonioidea	Trigoniidae		*Neotrigonia margaritacea*	gonochoric	?	F?	KU873118*
		Unionida	Etherioidea	Mycetopodidae		*Anodontites trapesialis*	hermaphrodite	no		KU873119*
				Iridiniidae		*Mutela dubia*	gonochoric	no		KU873120*
			Hyrioidea	Hyriidae		*Hyridella menziesii*	gonochoric	yes	F	KU873121*
									M	KU873122*
			Unionoidea	Margaritiferidae		*Cumberlandia monodonta*	gonochoric	yes	F	KU873123*
									M	KU873124*
						*Margaritifera falcata*	hermaphrodite	no	H	HM856634^6^
				Unionidae	Ambleminae	*Quadrula quadrula*	gonochoric	yes	F	FJ809750^4^
									M	FJ809751^4^
					Anodontinae	*Anodonta anatina*	gonochoric	yes	F	KF030964^63^
						*Cristaria plicata*	gonochoric	yes	F	FJ986302^a^
						*Lasmigona compressa*	hermaphrodite	no	H	HM856638^6^
						*Lasmigona subviridis*	hermaphrodite	no	H	HM856640^6^
						*Pyganodon grandis*	gonochoric	yes	F	FJ809754^4^
									M	FJ8v09755^4^
						*Utterbackia imbecillis*	hermaphrodite	no	H	HM856637^6^
						*Utterbackia peninsularis*	gonochoric	yes	F	HM856636^6^
									M	HM856635^6^
					Gonideinae	*Hyriopsis cumingii*	gonochoric	yes	F	FJ529186^b^
						*Hyriopsis schlegelii*	gonochoric	yes	F	HQ641406^c^
						*Inversidens japanensis*	gonochoric	yes	F	AB055625^d^
									M	AB055624^d^
						*Lamprotula tortuosa*	gonochoric	yes	F	KC109779^64^
						*Solenaia carinatus*	gonochoric	yes	F	KC848654^65^
									M	KC848655^65^
						*Solenaia oleivora*	gonochoric	yes	F	KF296320^66^
					Lampsilinae	*Lampsilis ornata*	gonochoric	yes	F	AY365193^67^
						*Toxolasma parvus*	hermaphrodite	no	H	HM856639^6^
						*Venustaconcha ellipsiformis*	gonochoric	yes	F	FJ809753^4^
									M	FJ809752^4^
					Unioninae	*Anodonta woodiana*	gonochoric	yes	F	HQ283344^e^
						*Unio pictorum*	gonochoric	yes	F	HM014130^68^
	Protobranchia	Solemyida				*Solemya velum*				JQ728447^51^
		Nuculida				*Nucula nucleus*				EF211991^f^
Caudofoveata						*Chaetoderma nitidulum*				EF211990^f^

For each species, the respective sex determination system and the presence of DUI is specified (DUI occurrence is uncertain at present in *N*. *margaritacea*). For each mtDNA, GenBank accession number and respective reference are indicated. mtDNA type F or M is indicated for DUI species only; *N*. *margaritacea* mtDNA here considered might be an F if DUI will be confirmed in this species. H type is indicated only for unionid species who secondarily lost DUI and became hermaphroditic. References for each sequence are indicated with superscripts after the accession numbers: asterisks (*) indicate sequences obtained in this study; numbers published data; and letters unpublished data (^a^Jiang W.P., Li J.L., Zheng R.L.; ^b^Zheng R.L., Li J.L.; ^c^Lin Q.H., Zeng L.G., Sheng J.Q., Wang J.H., Hong Y.J.; ^d^Okazaki M., Ueshima R.; ^e^Soroka M., Burzynsky A.; ^f^Dreyer H., Steyner G.).

### Phylogenetic analyses

**Figure 2:**
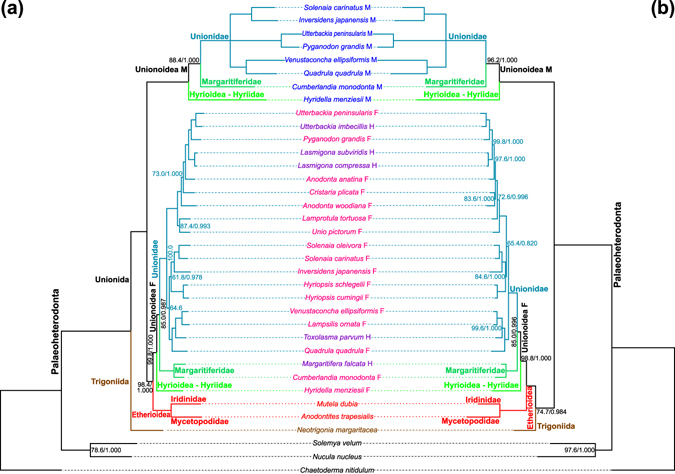
Phylogeny of Palaeoheterodonta. Trees were constructed using (**a**) nucleotide sequence of 12 mtDNA-encoded genes and (**b**) the respective inferred protein sequences of species in Table 1. Only ML trees are shown: topology of BI trees was largely congruent; the few small differences are only described in the main text and below. Support values at a node are shown only if (1) they were not 100% bootstrap support on the ML tree and 1.0 for the posterior probability values on the BI tree, or (2) when a node was only present in the ML tree. Support values, when shown, are presented next to the node as ‘ML bootstrap value/BI posterior probability’. Species names in the middle column are coded with the following colours, according to taxonomy and/or mtDNA type: black, non-Palaeoheterodonta outgroups; brown, Trigoniida; red, Etherioidea; pink, F mtDNA of DUI species; blue, M mtDNA of a DUI species; violet, H mtDNA of a secondarily hermaphroditic unionid. Branches inside Palaeoheterodonta are coloured according to taxa: brown, Trigoniida; red, Etherioidea; bright green, Hyriidae (Hyrioidea); dark green, Margaritiferidae (Unionoidea); aqua blue, Unionidae (Unionoidea). (**a**): A dissimilarity in the ‘Unionoidea F’ clade between BI and ML trees resides in the different position of the branch comprising five species of the subfamily Gonideinae (i.e., *H*. *cumingii*, *H*. *schlegelii*, *I*. *japanensis*, *S*. *carinatus*, and *S*. *oleivora*; Table 1) relative to all other Unionidae F/H mtDNAs: in the ML tree here shown, this clade is sister to the cluster containing Ambleminae and Lampsilinae (see Table 1 for those species), while in the BI (not shown) it is sister to the clade containing the other ten F and H Unionidae mt genomes. Because of the positions in both ML and BI trees of *L*. *tortuosa*, *A*. *woodiana* and *U*. *pictorum*, subfamilies Gonideinae and Unioninae (Table 1) are not supported as being monophyletic. (**b**): Both ML and BI protein-based trees differ from the nucleotide-based one in the relative position of *L*. *tortuosa*, which branches differently inside the same cluster (compare the two trees in figure). Again, Gonideinae and Unioninae are not monophyletic (Table 1).

Phylogenies of Palaeoheterodonta were reconstructed using the nt and amino acid (aa) sequences of 12 PCGs (except *atp8*) of the mt genomes in Table 1. Maximum likelihood (ML) and Bayesian inference (BI) were utilized. The resulting ML trees are shown in Fig. 2: minor differences at subfamily or species level between them and BI trees are described in Fig. 2 legend. In the nt-based phylogeny (Fig. 2a), *N*. *margaritacea* mtDNA is sister to all Unionida mtDNAs, which are separated into two major clades: the first comprises only M mt genomes of superfamilies Hyrioidea and Unionoidea, while the second the non-DUI mtDNAs of Etherioidea as a sister group to F and H mtDNAs of Hyrioidea and Unionoidea. In both of these main branches, *H*. *menziesii* mt genomes are always sister to Unionoidea ones. The three superfamilies of freshwater mussels are thus monophyletic in both main Unionida clades, as well as the families for which more than one species is available (i.e., Margaritiferidae and Unionidae; Table 1). A polytomy is found at the base of Unionidae M mtDNAs in the ML tree that is resolved in the BI one (not shown), where these genomes have the same relationships as the F mtDNAs of the respective species. In the aa-based phylogenies (Fig. 2b), Palaeoheterodonta mt genomes are split in two major branches: one in which *N*. *margaritacea* is sister to a clade comprising Etherioidea plus the F and H mt genomes of all other freshwater mussels, and another containing all Unionida M mtDNAs (Hyrioidea and Unionoidea again have the same relationships in both branches). Unionida mt genomes are therefore split on two different branches because of *N*. *margaritacea* nested position. The topology of the Hyrioidea + Unionoidea F/H clade in both trees is identical to that of the BI nt-based tree described above and in the figure legend. In the ML tree shown in Fig. 2b, the same polytomy found in the nt-based tree in the M clade is present, which is again resolved in the BI tree (not shown) as described above.

### Search for additional ORFs and functional characterization

Excluding the F-*orf* and M-*orf* in *H*. *menziesii* and *C*. *monodonta* mtDNAs, we found a total of 375 new possible ORFs coding for >10 aas in the unassigned regions (URs) of the new mt genomes. Specifically, we found 79 ORFs in *N*. *margaritacea*, 58 in *A*. *trapesialis*, 50 in *M*. *dubia*, 34 in *H*. *menziesii* F mtDNA and 60 in its M, and 41 in *C*. *monodonta* F mtDNA and 53 in its M (Supplementary Information 4). None of the 375 ORFs translated protein sequences contain statistically supported conserved domains. Among F- and M-*ORF*s, only *H*. *menziesii* M-*ORF* exhibited conserved domains (Supplementary Table S6 in Supplementary Information 4). To search for homologs, we compared the putative new ORF products, plus the *H*. *menziesii* and *C*. *monodonta* F- and M-*ORFs*, to the standard 13 mtDNA-encoded proteins, to proteins in databases, to lineage-specific ORFans of known DUI species, and to themselves (Supplementary Information 4). No significant hits were found in the comparison with standard mtDNA-encoded proteins. Searches on the Swiss-Prot database gave no results, while TrEMBL gave a total of 266 hits for 30 of the new ORFs. Most of the hits are to putative uncharacterized proteins without a functional description; however, for certain ORFs, some hits were to characterized proteins (Supplementary Information 4). The new ORFs proteins and the F- and M-*ORF* from *H*. *menziesii* and *C*. *monodonta* have no similarities to either known F- or M-*ORF*s of the DUI taxa *Mytilus* spp., *Ruditapes philippinarum*, or *Musculista senhousia*. For eight ORFs in *H*. *menziesii* M mtDNA located in two distant URs (UR3 and UR17), their similarity appears to be due to the presence of a sequence block shared between those URs (Fig. 1 and Supplementary Information 2). *C*. *monodonta* M-*ORF* has a hit with CmonM_UR_24_11 (phmmer^24^ results: E-value = from 3.0E-05 to 6.2E-05, score = from 19.0 to 19.6; Supplementary Information 4), a new ORF just upstream of it between tRNA-Asp and *atp8* (Fig. 1). A structural alignment of these two proteins made with T-Coffee Expresso^25–27^ showed high similarity, with many identical aas (score = 91, cons = 9; alignment shown in Supplementary Information 5, Supplementary Fig. S4). Finally, Hidden Markov model (HMM) profiles of unionid F-*ORF*s and M-*ORF*s produced by ref. 12 were also used to search for similarities with the translated proteins of the new ORFs (Supplementary Information 4). Apart from F- and M-*ORF*s, no other hits were found using the F-*ORF* profiles, but one hit was found with M-*ORF* profiles for Atra_UR_22_18 protein (hmmsearch^24^ results: E-value = 0.048, score = 6.5), an ORF located between tRNA-Asp and *atp8* (like CmonM_UR_24_11 in *C*. *monodonta* M mtDNA described above) inside the second large repeat in *A*. *trapesialis* mtDNA (Fig. 1), in correspondence of *atp8* pseudogenized fragments (Supplementary Information 2).

### Structural characterization of proteins

**Figure 3:**
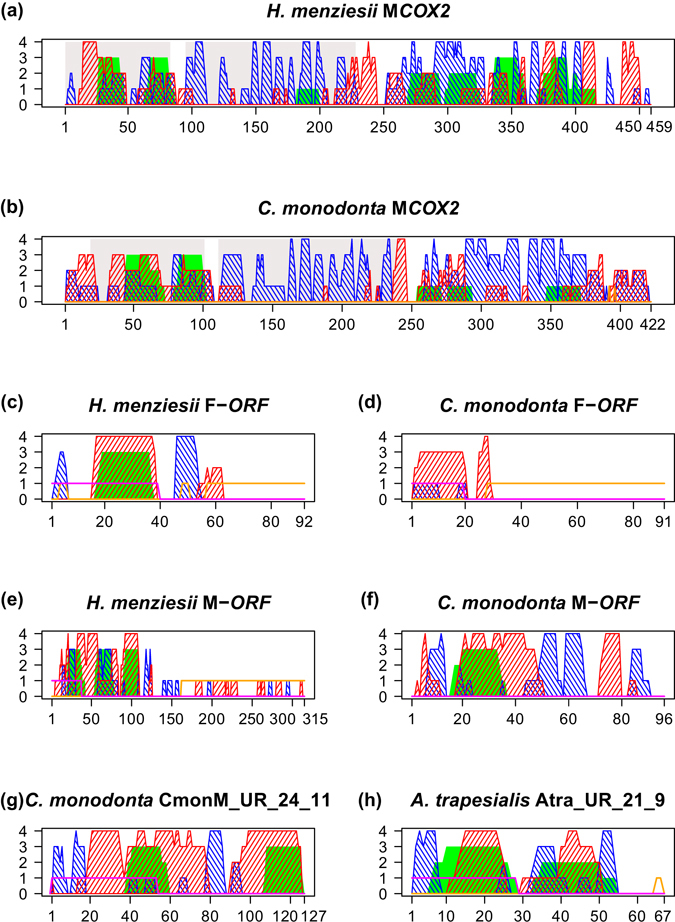
Structural characterization of proteins encoded by lineage-specific genes and ORFs. Visual summary of the characterization made with Quick2D^50^ on M*COX2*, F-*ORF*, M-*ORF*, and the putative proteins encoded by the ORFs CmonM_UR_24_11 and Atra_UR_21_9 from *Hyridella menziesii*, *Cumberlandia monodonta*, and *Anodontites trapesialis* mtDNAs. Complete Quick2D outputs are displayed in Supplementary Information 5. In each panel, on the X axis are the amino acid positions of the protein, while on the Y is the support for each feature (described below) shown in terms of how many methods indicated a certain characteristic at a given position. Colour code of features: full green areas, transmembrane regions; striped red areas, regions forming helices; striped blue areas, regions forming beta-sheets; purple lines, signal peptide signature; orange lines, disordered regions. The two grey squares on the background of panels (**a**) and (**b**) represent, from left to right, the boundaries of the conserved transmembrane and periplasmic domains of *COX2*.

We performed a structural characterization of *H*. *menziesii* and *C*. *monodonta* M*COX2*, since they are the first examples of such elongated proteins for Hyriidae and Margaritiferidae, and of the F- and M-*ORF*s of the same species to compare them with the putative proteins CmonM_UR_24_11, Atra_UR_21_9, and Atra_UR_22_18 given their overall similarity described above. Supplementary Information 5 contains all structural alignments cited below (shown in Supplementary Fig. S4) and details of the characterization, which is summarized in Fig. 3. *H*. *menziesii* and *C*. *monodonta* M*COX2* extensions (Fig. 3a,b) have a different number of predicted transmembrane sections (four versus three), more supported in *H*. *menziesii* than in *C*. *monodonta*, but the portion containing the first two helices in both proteins aligns with good quality. *H*. *menziesii* and *C*. *monodonta* F-*ORF*s (Fig. 3c,d) share a largely identical structure but they align well only in the second half of the sequence, and the first helix is predicted as having transmembrane properties only in *H*. *menziesii*. M-*ORF*s (Fig. 3e,f) are on the contrary quite variable, both in length and structure. The first half of *H*. *menziesii* M-*ORF* broadly resembles the whole protein of *C*. *monodonta* (and this part has a better overall alignment); a signal peptide in this region is recognized only in *H*. *menziesii*. The second half of *H*. *menziesii* M-*ORF* encoded by the repeat region described above (from position ~140) may form some helices and beta-sheets, but it is also characterized as largely disordered. *C*. *monodonta* CmonM_UR_24_11 protein structure (Fig. 3g) is largely overlapping with that of the same species M-*ORF* (Fig. 3f; see above), but contrary to it, a signal peptide is found at CmonM_UR_24_11 N-terminus. Atra_UR_21_9 putative protein structure (Fig. 3h) is comparable to that of *C*. *monodonta* M-*ORF* and CmonM_UR_24_11, and to the first half of *H*. *menziesii* M-*ORF*. A signal peptide is predicted at its N-terminus, as well as a small disordered C-terminal segment. Despite the presence of *atp8* fragments in Atra_UR_21_9, it does not align well with *A*. *trapesialis ATP8* protein. Overall, the proteins scoring the best alignments with Atra_UR_21_9 are *C*. *monodonta* F-*ORF* and M-*ORF*, followed by *H*. *menziesii* M-*ORF* and F-*ORF*. *H*. *menziesii* M-*ORF* has the highest number of identical aas aligned compared to all other proteins considered (24 aas). The short length of the Atra_UR_22_18 protein (22 aas; Supplementary Information 4) prevented a structural characterization. Its best alignments are with Atra_UR_21_9 protein and CmonM_UR_24_11. As for Atra_UR_21_9, the sequence of Atra_UR_22_18 aligns with *A*. *trapesialis ATP8* with low scores. These results indicate that structural similarities are maintained in the elongated M*COX2*, as well as in the lineage-specific F- and M-*ORF*s proteins, of hyriids and margaritiferids. Also, they evidence the strict relationships of the putative proteins encoded by CmonM_UR_24_11, Atra_UR_21_9, and Atra_UR_22_18 with M-*ORF*s.

## Discussion

The sequencing of mt genomes representing Palaeoheterodonta taxa for which no complete sequences were available until now sheds new light on the evolution of mtDNA and on mitochondrial inheritance systems in this ancient bivalve taxon. First, mt genome organization in Palaeoheterodonta seems to be rather stable compared to other bivalve taxa, in which important rearrangements are frequently observed among relatively close taxa within the same family or genus^28^. Indeed, the major difference between *N*. *margaritacea* and Unionida is only an inversion between *cox2* and *nad3*, and mt genomes of Etherioidea (i.e., *M*. *dubia* and *A*. *trapesialis*) practically have the same organization as the F ones of Hyrioidea and Unionoidea, apart from the absence of F-*orf* (Fig. 1). *A*. *trapesialis* mtDNA, however, shows a highly rearranged area between *nad4L* and *atp6* composed of three tandem repeats, resulting in an organization and gene order similar to those of M mt genomes in DUI unionids (Fig. 1). Specifically, our results suggest that the position of tRNA-Asp and *atp8*, identical to that of M mtDNAs^4^, is most probably the outcome of a tandem duplication of the segment containing the original functional copies followed by pseudogenization of supernumerary ones that are still recognizable in the repeated sequences (i.e., a tandem duplication-random loss event^29^). For example, ORFs Atra_UR_21_9 and Atra_UR_22_18, located in the repeats, contain recognizable parts of *atp8*. Because of these rearrangements, Atra_UR_21_9 is located in exactly the same position as the M-*orf* in DUI freshwater mussels (between *nad4L* and tRNA-Asp^4^; Fig. 1), and its putative protein also has structural analogies to the M-*ORF* of DUI unionids (Fig. 3). Again in support of the tandem duplication hypothesis, the protein translated from Atra_UR_22_18 (between tRNA-Asp and *atp8*; Fig. 1) has similarities to both the *ATP8* produced by the same genome and to the putative product of Atra_UR_21_9. The similarity between the two *A*. *trapesialis* ORFs and their location mirror what is observed in the M mtDNA of the DUI margaritiferid *C*. *monodonta* (Fig. 1), whose M-*orf* and additional ORF CmonM_UR_24_11 (Fig. 1) are located in the same positions and encode for proteins comparable in structure (Fig. 3). *H*. *menziesii* M mtDNA shows the longest *cox2* and M-*orf* genes found in the M mt genome of any DUI unionid. The M-*orf* of this species has a complex repeat region in its 3′ half downstream from the region whose protein product is more similar to other M-*ORF*s (see Fig. 3). Until now, tandem repeats in DUI-related ORFans have been observed only in the H-*orf* of hermaphroditic freshwater mussels that lost DUI secondarily^6, 12^, but to our understanding, this feature of the M-*orf* does not seem to have affected the DUI system of *H*. *menziesii*.

**Figure 4:**
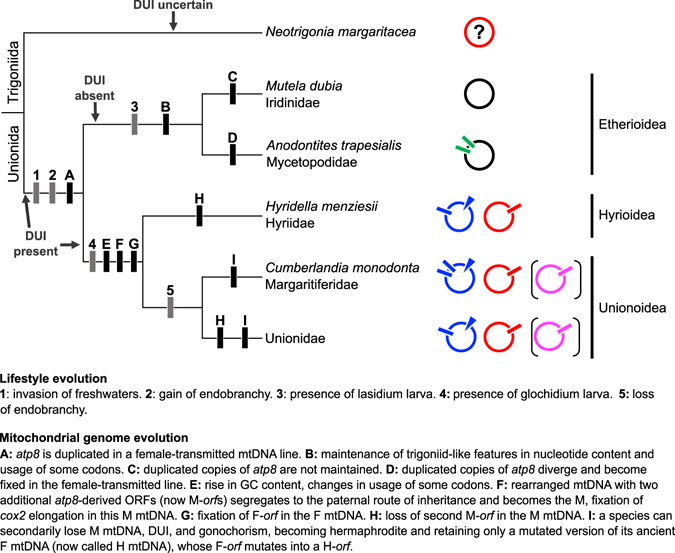
Model for the evolution of mt genomes and DUI in Palaeoheterodonta. The backbone tree represents the phylogeny obtainable by removing the M mtDNAs from the trees in Fig. 2. DUI presence/absence is specified for all major clades. At the tip of each final branch, the names of the species for which we obtained the mtDNA sequences in this study are indicated together with the respective family (no species are enlisted for Unionidae); superfamily affiliations are specified with bars on the right side of the figure. Circles beside a final branch represent a schematic mt genome structure with the following colour code: black, mtDNA of a non-DUI species; red, F mtDNA in a DUI species/family; blue, M mtDNA in a DUI species/family. Purple circles in parentheses represent the typical H mtDNA of secondarily hermaphroditic species in a given family. For *N*. *margaritacea* mt genome, the interrogation point highlights the uncertain presence of DUI in this species, while the red colour the fact that the genome we obtained, if DUI is present, would be its F. Bars on the mtDNAs represent *atp8*-derived ORFs (green), M-*orf*s (blue), F-*orf*s (red), or H-*orf*s (purple), while blue triangles represent the elongated *cox2* genes in M mtDNAs. The position of these latter features on the schematic mtDNAs reflects their actual location in the mt genomes (compare with Fig. 1). Bars inside the Unionida branches of the tree indicate evolutionary events and/or character states of lifestyle-related features as enlisted by ref. 14 (grey bars) or evolutionary events of mt genomes (black bars), described inside the figure.

By removing the sperm transmitted mt genomes from the trees in Fig. 2, we can see that the relationships among families indicated by female-transmitted mtDNAs are exactly the same in both phylogenies. The evolutionary tree of Palaeoheterodonta based on egg-transmitted mtDNAs obtained in this way (shown in Fig. 4) confirms the long-established sister group relationship of orders Trigoniida and Unionida. Inside the monophyletic order Unionida, the superfamily Etherioidea appears to be a monophyletic sister group to a clade comprising the monophyletic superfamilies Hyrioidea and Unionoidea as sister taxa. The relationship of Hyriidae with the other five families of Unionida has been highly debated^20^, but all our reconstructions have strong support (Fig. 2). As observed in previous studies (e.g., ref. 13), our phylogenies gave a gender-joining^15^ topology for palaeoheterodont mt genomes (Fig. 2). Because protein sequences are more conserved at this taxonomic level and less prone to saturation^18^, we consider this latter reconstruction to be the more supported and we thus infer that DUI was present prior to the divergence of the orders Trigoniida and Unionida. However, this interpretation must be tempered by the uncertain presence of DUI in *N*. *margaritacea* ^21^, the sister taxon to all Unionida in our analysis (Fig. 4). If DUI can be clearly demonstrated in this species, then the hypothesis that it was present in the Trigoniida-Unionida common ancestor can be supported, thus dating the presence of DUI in Palaeoheterodonta at >200 MYA (based on the estimates by ref. 30). If not currently present in the Trigoniida, DUI could have been lost independently in this order or, alternatively, DUI may not have been present in the last common ancestor of Trigoniida-Unionida and gained independently in Unionida (a conclusion different to what the aa-based tree indicates, but in accord with the nt-based tree; Fig. 2). Following a strict interpretation of the phylogeny in Fig. 2b, the two etherioids *M*. *dubia* (gonochoric) and *A*. *trapesialis* (hermaphroditic) must have lost DUI at some point during their evolution, maintaining only a female-transmitted mt genome. It is unknown, however, if this inferred loss might have been ancestral to all Etherioidea or if there were two independent events, and if such events affected their sex determination mechanism. Given the high plasticity of sex determining systems in bivalves (Breton S. *et al*. in preparation), and the extremely long evolutionary time that might separate present-day *M*. *dubia* and *A*. *trapesialis* from the putative loss event(s), the gonochorism and hermaphroditism of these two species^22, 23^ might not be directly related to the putative loss of DUI. Without additional information, it is presently more parsimonious to infer that DUI was lost once, in the common ancestor to the Etherioidea.

The hypothetical origin of freshwater mussels M-*orf* genes from a duplication of *atp8* recently proposed by ref. 12 is supported by our model of DUI evolution (Fig. 4), in which the M-*orf*s of *H*. *menziesii* and *C*. *monodonta* (and of all DUI unionid species in general) are suggested to share the same ancestry with *A*. *trapesialis* Atra_UR_21_9 and Atra_UR_22_18. These latter ORFs contain clearly recognizable pseudogenized fragments of *atp8* derived from its duplication, and the protein encoded by Atra_UR_22_18 was recognized by the M-*ORF* HMM profiles. The architecture of *A*. *trapesialis* mtDNA and of M mt genomes of distantly related DUI species (Fig. 1) must have arisen in the first phases of Unionida radiation. Given its close phylogenetic relationship to the F mtDNA of DUI unionids (Fig. 2), the rearranged mt genome of *A*. *trapesialis* most probably originated from a female-transmitted genome in the common ancestor of the two main lineages Etherioidea and Hyrioidea + Unionoidea (Fig. 4, step A). We hypothesize this ancestor to have been gonochoric, since our phylogeny suggests that it had DUI (Figs 2 and 4), and DUI has never been observed in hermaphroditic species^6, 14^. If true, a rearranged mt genome structure (1) was fixed in the maternal route of inheritance in the ancestor of etherioids, which contemporarily lost DUI, and Iridinidae lost this M-like architecture during their evolution (Fig. 4, steps C and D), and (2) invaded the paternal route of transmission in the Hyrioidea + Unionoidea lineage substituting a pre-existing M (i.e., a role reversal event^16^). Alternatively, if we hypothesize that the last common ancestor of the two main unionid lineages did not have DUI, then DUI appeared only in the Hyrioidea + Unionoidea lineage in which the rearranged mt genome became male-transmitted. This option, which would be in line with the hypothesis of multiple origins of DUI in bivalves^1, 8^, would move the origin of DUI in freshwater mussels at the earliest to the split between Hyrioidea and Unionoidea. However, this hypothesis is currently not supported by our phylogenetic analyses (Fig. 2).

The switch of the rearranged mt genome to the paternal route of inheritance in the Hyrioidea + Unionoidea line (Fig. 4, step F) might have been caused by the two new *atp8*-derived ORFs. The original ability of the *ATP8* protein to be incorporated into the ATPase complex might have been maintained in the duplicated gene products, giving them the capacity to alter membrane potential by modulating ATPase activity^12, 31^ or, at least, to be inserted into mitochondrial membranes. Our results (Supplementary Table S6) and previous studies^8, 12^ corroborate a proposed involvement of M-*ORF*s in membrane association, microtubule binding and RNA interactions, activities that can be directly related to the peculiar behaviour of sperm mitochondria in DUI species during early male development (see references in ref. 32) and to the proposed sex determining capabilities of M mtDNA. The *atp8*-derived ORFs might have granted selfish characteristics to their host mtDNA or developed the ability to distort sex determination to maleness^17^, transforming them into M-*orf*s (we will refer to them as M-*orf*1, between tRNA-Asp and *nad4L*, and M-*orf*2, between *atp8* and tRNA-Asp, for simplicity). This M lineage subsequently evolved in its own particular ways in the different families. In Hyriidae (*H*. *menziesii*) and Unionidae, M-*orf*2 was lost (Fig. 4, step H), and M-*orf*1 acquired repetitive sequences and became unusually long in the first but not in the latter of these families (Figs 1 and 4e). Only Margaritiferidae (*C*. *monodonta*) retained the ancestral state in their M mtDNA by maintaining both M-*orf*1 and M-*orf*2 (Figs 1 and 4, step G): their translated proteins are very similar in structure and sequence, which can be due to either their common ancient origin from *ATP8* (see above) and/or to convergent functions and convergent evolution. Since it is, however, the first observation of an M-*orf*2 gene, more complete M mt genomes from DUI margaritiferids will be required to clarify its evolution. Moreover, the inferred independent loss of M-*orf*2 in Hyriidae and Unionidae (see Fig. 4) questions (1) the role of this ORFan for the DUI system, if any, in Margaritiferidae, and (2) if there was a complete transfer to the nucleus in Hyriidae and Unionidae or if the second M-*orf* was simply lost from their M mtDNA.

At the same time, in the female-transmitted line, the *atp8*-derived ORFs are not maintained, the F-*orf* appears upstream *nad2* and is fixed (Fig. 4, step G) (whether this ORFan was the result of another gene duplication, specifically of *nad2*, is discussed in ref. 12). Previous studies suggested that mitochondrial ORFans in bivalves could act as sex ratio distorters and restorers, i.e., they would be key elements of a sex determination system involving F and M mt (and nuclear) genomes^6, 12, 17^. The role of the F-*orf* is still unknown, but the macromutations it accumulates when a unionoid becomes hermaphroditic and loses DUI (i.e., when a F*-orf* becomes an H*-orf*; Fig. 4, step I) support a concerted action between F-*orf* and M-*orf* in maintaining gonochorism^6, 12^. Concurrently, an elongated *cox2* was also fixed in the M line (Fig. 4, step F): although no molecular evidence has been gathered yet to support this hypothesis, it was suggested that the presence of the M*cox2* elongation in DUI freshwater mussels could prevent role reversals^10^, a phenomenon observed in *Mytilus* species who have DUI and lack such elongated gene^1^. The prevention of role reversals and recombination might have been positively selected to avoid the rise of new mt genomes possessing both F- and M-*orf* and keep the gonochoristic sex determination system safe. The connection among ORFans, DUI, and gonochorism in freshwater mussels is thus forged: this bond is surely strong, as F and M lineages have been evolving separately for several hundred million years^2, 33^, and when a modification in one of these three elements rarely occurs, it unequivocally forces the other two to degenerate^6^ (Fig. 4, step I).

## Conclusion

In this study we propose a model for the evolution of the unique mitochondrial inheritance system that is DUI in bivalves, in particular in the order Unionida. We suggest that present-day sperm-transmitted mtDNAs evolved from a deviant mt genome, with possibly selfish and/or sex distorting qualities^6, 17^, in a supposedly DUI gonochoric ancestor of all freshwater mussels. Specifically, two *atp8*-derived ORFs are suggested to have triggered a change in inheritance route of the mt genome carrying them. One of them became the M-*orf*, an ORFan gene thought to be involved in the maintenance of gonochorism in freshwater mussels with DUI^6^. How these ORFs might have established a link between mtDNA and sex determination is however still obscure. Deviant mt genomes with sex distorting qualities have been observed also outside bivalves^34^, but the general influence of mitochondria and their genome on sex determination in animals remains largely unknown. In bivalves, who lack heteromorphic sex chromosomes and are mostly gonochoric but show a myriad of variations on hermaphroditism (Breton S. *et al*. in preparation), divergent selfish mtDNAs carrying novel ORFans would have an easy way into moulding their flexible sex determination system to achieve constant transmission through the generations^17^. Our results indeed demonstrate how plastic the architecture of the small mt genome can potentially be, and how it could deeply influence the biology of its ‘host’ on large evolutionary scales beyond the mere production of energy for the cell^35^.

## Methods

### Animal sampling and genome sequencing

Animals, all of which (1) are members of the bivalve clade Palaeoheterodonta (see Table 1 for a detailed taxonomy of the species), (2) have different reproductive strategies, and (3) do or do not show DUI evidence, were collected from the following locations: *Neotrigonia margaritacea* Lamarck 1804 (Trigoniida: Trigoniidae; gonochoric, DUI status uncertain) from Gulf St. Vincent (South Australia); *Mutela dubia* Gmelin 1791 (Unionida: Iridinidae; gonochoric without DUI) from the Nile River (Al Jizah, Egypt); *Anodontites trapesialis* Lamarck 1819 (Unionida: Mycetopodidae; hermaphroditic without DUI) from the Rio Madre de Dios (Madre de Dios, Peru); *Hyridella menziesii* Gray 1843 (Unionida: Hyriidae; gonochoric with DUI) from the Taieri River (South Island, New Zealand); and *Cumberlandia monodonta* Say 1829 (Unionida: Margaritiferidae; gonochoric with DUI) from the Clinch river (Claiborne County, TN, USA). Specimens were sexed through microscopical examination of the gonads to check for the type of gametes produced. Total DNA was extracted from gonadal tissue of one hermaphroditic, or one female and one male individual for each species (except for *N*. *margaritacea* for which only one female was available) with a QIAGEN DNeasy animal kit (QIAGEN Inc., Valencia, CA, USA) using the animal tissue protocol. Sequencing of the seven mt genomes (i.e., both F and M for *H*. *menziesii* and *C*. *monodonta*, one from *M*. *dubia* and *A*. *trapesialis*, and the putative F from *N*. *margaritacea*) was performed by Genome Sequencer FLX sequencing service (McGill University, Montréal, Quebec, Canada).

### Annotation and characterization of mtDNAs

PCGs and rRNA coding genes were annotated with MacVector Sequence Analysis Software 10.0 (Accelrys Inc, San Diego, California, USA). ARWEN^36^ and tRNAscan^37^ were used to annotate tRNA genes. When ARWEN and tRNAscan failed to recognize a tRNA, the program MiTFi^38^ (implemented in MITOS^39^) was used, because it implements an alternate structure-based covariance model to detect tRNA folding. Gene order comparisons among the newly sequenced mt genomes were performed manually. Tandem repeat sequences and other repeats in the mtDNAs were defined using Tandem repeats finder^40^ and BLASTn^41^, using for the latter a single whole genome sequence as both query and subject with default parameters. Diagrams of mtDNA structures were generated using the program GenomeVx^42^. Nucleotide content of *N*. *margaritacea* and all freshwater mussel mt genomes in Table 1, as well as the codon usage (expressed as relative synonymous codon usage, RSCU) in the 13 standard PCGs of the seven newly sequenced mtDNAs, were calculated using MEGA 5.2.2^43^. Pairwise-distances (p-distances) for standard PCGs (i.e., those that code for subunits of the electron transport chain or ATPsynthase) and rRNA genes, PCGs translated proteins, as well as *d*_N_ and *d*_S_ statistics for the 13 PCGs, were calculated with MEGA 5.2.2. Alignments for these analyses were produced by MEGA 5.2.2 with MUSCLE^44^, using codon alignment for PCGs and DNA for rRNA genes. Intraspecific p-distance comparisons between F and M mtDNAs of *H*. *menziesii* and *C*. *monodonta* for PCGs were calculated, as well as the interspecific ones (i.e., a genome of one species versus the genome of another). For rRNA genes, p-distances were only calculated for the 12S and 16S genes for *H*. *menziesii* and *C*. *monodonta*. All the obtained statistics were processed with R version 3.1.0^45^ on RStudio 0.98^46^, which was also used for drawing graphs.

### Characterization of ORFs

URs of all newly sequenced mt genomes were extracted and given a sequential identification number, choosing as number 1 the UR upstream from *cox1* (see Fig. 1) and then numbering all others in a clockwise direction. We then searched the URs for additional ORFs with the EMBOSS getorf program^47^, considering only ORFs at least 33 nts in length (i.e., a minimum of 10 codons encoding aas plus a stop codon) using the invertebrate mitochondrial genetic code. These ORFs were translated into the corresponding protein sequences in two ways (again with getorf): (1) using the corresponding aa in the presence of alternative start codons, and (2) always using methionine as the first aa regardless of the predicted first codon in the ORF. Both sets of translations, plus *H*. *menziesii* and *C*. *monodonta* lineage-specific F-*orf* and M-*orf* translated protein sequences, were used for the subsequent analyses. The set of ORF proteins was searched for conserved domains against the CDD database^48^ (last accessed June 2015) with Batch Web CD-search tool^49^, using the ‘live search’ mode with default settings and a cutoff E-value of 0.001. We locally ran phmmer (present in the HMMER^24^ suite of programs) to compare the set of protein sequences against: (1) the 13 standard proteins encoded by the newly sequenced mtDNAs, to find unannotated, or degenerated but still recognizable, duplicated genes; (2) the proteins present in the Swiss-Prot and TrEMBL databases (downloaded October 2015), to find homologs; (3) all known F- and M-*ORF* proteins of non-unionid DUI species (i.e., *Mytilus edulis*, *M*. *galloprovincialis*, *M*. *trossulus*, *M*. *californianus*, *M*. *senhousia*, and *R*. *philippinarum* ^5, 8^) to find similarities in evolutionarily distant taxa; (4) F-*ORF*, M-*ORF*, and H-*ORF* proteins of freshwater mussel species used in the phylogenetic analyses (see below), to find similarities among unionids; and (5) the protein set itself, to find similar proteins encoded in the same or in different mtDNAs. Default parameters were used for all the phmmer searches (i.e., an E-value cutoff of 0.001). We also ran hmmsearch^24^ locally to search the new ORF proteins against unionoids F-*ORF* and M-*ORF* HMM profiles produced from the respective protein alignments; these profiles were built for an extensive *in silico* characterization of freshwater mussel lineage-specific ORFans by ref. 12. An E-value cutoff of 1 was used in this case to allow the profiles to detect homologs other than F- and M-*ORF*s themselves. Quick2D^50^ was used to characterize the structure of M*COX2*, M-*ORF*, and F-*ORF* of *H*. *menziesii* and *C*. *monodonta*, plus other putative proteins that were found to be comparable to lineage-specific ORFan products. Structural alignments for ORF proteins of particular interest were produced by T-Coffee Expresso^25–27^ using default parameters. The two *COX2* conserved domains inside the *H*. *menziesii* and *C*. *monodonta* M*COX2* proteins were identified with BLASTp^41^.

### Phylogenetic analyses

We performed a phylogenetic analysis on Palaeoheterodonta bivalves using all standard PCG sequences, except *atp8* because of its high variability and lack of confidence in alignment, from the seven newly sequenced mtDNAs and 26 mt genomes of other freshwater mussels belonging to 20 species (15 F, 5 H, and 6 M mtDNAs; see Table 1 for species list, mtDNAs, and references). To root the trees in the subsequent analyses, we chose to use PCG sequences from the mtDNAs of the Protobranch bivalves *Solemya velum* (Solemyida) (GenBank accession number: JQ728447^51^) and *Nucula nucleus* (Nuculida) (GenBank accession number: EF211991; Dreyer H. and Steiner G. personal communication), plus those from the aplacophoran mollusc *Chaetoderma nitidulum* (GenBank accession number: EF211990; Dreyer H. and Steiner G. personal communication). Thus, a total of 36 taxa was used to reconstruct the phylogenies. The T-Coffee algorithm^25^ was used for single alignments. For aas, we carried out structural alignments through PSI-BLAST^52^ in the succession PSI-Coffee > Expresso > accurate. For nts we used M-Coffee, starting from MAFFT^53^ and MUSCLE^44^ libraries. Alignments were masked to discard phylogenetic noise using Aliscore 2.0^54^, BMGE 1.1^55^ (using BLOSUM95 for aas), Gblocks 0.91b^56^, and Noisy^57^, with default settings. Only sites kept by at least three out of four of these programs were retained for further analyses. We selected the best-fitting partitioning scheme and molecular evolution models using PartitionFinderProtein and PartitionFinder 1.1.0^58^ under the Bayesian Information Criterion (BIC) and a greedy approach. The phylogenetic tree was estimated with both ML and BI approaches. The software RAxML 8.2.0^59^ was chosen for the ML inference under the CAT model and using 500 bootstrap replicates. BI was conducted with MrBayes 3.2.1^60^ as in ref. 18. The evolutionary models chosen with the BIC for the nt alignments of the PCGs were the following: GTR + I + G for *atp6*, *cox1*, *cox2*, *cox3*, *cytb*, *nad1*, *nad2*, *nad4*, and *nad5*; HKY + I + G for *nad3* and *nad6*; and HKY + G for *nad4L*. Trees were graphically edited using PhyloWidget^61^ and Dendroscope 3.3.2^62^.

## Electronic supplementary material

Supplementary Information 1

Supplementary Information 2

Supplementary Information 3

Supplementary Information 4

Supplementary Information 5
