# Supplementary material for: Evolution of sex-dependent mtDNA transmission in freshwater mussels (Bivalvia: Unionida)
Source: Sci Rep. 2017 May 8;7:1551. doi: 10.1038/s41598-017-01708-1 (PMC5431520; doi:10.1038/s41598-017-01708-1)
Supplement: Supplementary file 1 — Supplementary Information 1 [file 41598_2017_1708_MOESM1_ESM.pdf]

Evolution of sex-dependent mtDNA transmission in freshwater mussels (Bivalvia: Unionida)

Davide Guerra[1], Federico Plazzi[2], Donald T. Stewart[3], Arthur E. Bogan[4], Walter R. Hoeh[5] & Sophie Breton[1]

[1]Département de Sciences Biologiques, Université de Montréal, Montréal H2V 2S9, Quebéc, Canada. [2]Dipartimento di Scienze Biologiche, Geologiche ed Ambientali (BiGeA), Università di Bologna, Bologna 40126, Italy. [3]Department of Biology, Acadia University, Wolfville B4P 2R6, Nova Scotia, Canada. [4]North Carolina Museum of Natural Sciences, Raleigh, NC 27607, USA. [5]Department of Biological Sciences, Kent State University, Kent, OH 44242, USA.

Supplementary Information 1

Descriptive and comparative analyses

(Supplementary Figures S1, S2, S3; Supplementary Tables S1, S2, S3)

	Unionida					
	Etherioidea		F mtDNAs		M mtDNAs	
Nmar	***Atra***	***Mdub***	***Hmen* F**	***Cmon* F**	***Hmen* M**	***Cmon* M**
- cox1	- cox1	- cox1	- cox1	- cox1	- cox1	- cox1
- nad3	- cox2	- cox2	- cox2	- cox2	- cox2	- cox2
- cox2	- nad3	- nad3	- nad3	- nad3	- nad3	- nad3
	- H	- H	- H	- H	C	
	A	A	A	A	A	A
S2	S2	S2	S2	S2	S2	S2
					I	
S1	S1	S1	S1	S1	S1	
N						
E	E	E	E	E	E	E
			F-orf	F-orf		
nad2	nad2	nad2	nad2	nad2	nad2	nad2
	M	M	M	M	M	M
W	W	W	W	W	W	W
R	R	R	R	R	R	R
12S	12S	12S	12S	12S	12S	12S
K	K	K	K	K	K	K
T	T	T	T	T	T	T
						S1
Y	Y	Y	Y	Y	Y	Y
16S	16S	16S	16S	16S	16S	16S
L1	L1	L1	L1	L1	L1	L1
	N	N	N	N	N	N
				E		
P	P	P	P	P	P	P
cytb	cytb	cytb	cytb	cytb	cytb	cytb
F	F	F	F	F	F	F
- nad5	- nad5	- nad5	- nad5	- nad5	- nad5	- nad5
- H					- H	- H
A						
Q	Q	Q	C	Q		Q
C	C	C	Q	C		C
M						
I	I	I	I	I		I
V	V	V	V	V	V	V
L2	L2	L2	L2	L2	L2	L2
nad1	nad1	nad1	nad1	nad1	nad1	nad1
					Q	
G	G	G	G	G	G	G
nad6	nad6	nad6	nad6	nad6	nad6	nad6
- nad4	- nad4	- nad4	- nad4	- nad4	- nad4	- nad4
- nad4L	- nad4L	- nad4L	- nad4L	- nad4L	- nad4L	- nad4L
					- M-orf	- M-orf
- atp8	- D	- atp8	- atp8	- atp8	- D	- D
- D	- atp8	- D	- D	- D	- atp8	- atp8
- atp6	- atp6	- atp6	- atp6	- atp6	- atp6	- atp6
- cox3	- cox3	- cox3	- cox3	- cox3	- cox3	- cox3

Supplementary Figure S1. Comparison of gene order among the seven new mitochondrial genomes. Empty spaces between cells are virtual and used only for the purpose of gene alignment. tRNA genes are indicated with the amino acid they encode (for tRNAs encoding serine and leucine the first and second gene are specified). A dash (-) before a gene name specifies it is encoded in the reverse direction (see also Fig. 1). Abbreviations: Nmar, *N. margaritacea*; Atra, *A. trapesialis*; Mdub, *M. dubia*; HmenF and HmenM, *H. menziesii* F and M mtDNA respectively; CmonF and CmonM, *C. monodonta* F and M mtDNA respectively. Color code of cells: grey, positions conserved among all seven mt genomes; yellow, positions specific to Unionida and not found in *N. margaritacea*; green, positions conserved among Etherioidea and F mtDNAs of Unionida; orange, positions shared between *N. margaritacea* and other mtDNAs; red, position of F-*orf*; light blue, position of M-*orf*; white, positions private to only one mtDNA or that do not fit the criteria described above. *N. margaritacea* gene order is largely comparable to those of Unionida, the main differences that distinguish it from Unionida being an inversion of *cox2* and *nad3* and the positions of tRNA-Asn and tRNA-Met. *N. margaritacea* mtDNA also has intermediate features shared with *M. dubia* and the F genome of *H. menziesii* and *C. monodonta*, such as the tRNA-Ser2+tRNA-Ser1 and the *atp8*+tRNA-Asp blocks, and with the M genome of *H. menziesii* and *C. monodonta*, such as the position of tRNA-His upstream of *nad5*. The mt genomes of *A. trapesialis* and *M. dubia* have practically the same gene order as *H. menziesii* and *C. monodonta* F mtDNAs, the only exceptions being the absence of an F-*orf* and the inversion in *A. trapesialis* of *atp8* and tRNA-Asp as in *H. menziesii* and *C. monodonta* M mtDNAs. F mtDNAs of *H. menziesii* and *C. monodonta* have the same gene order, with the exception of an inversion of tRNA-Cys and tRNA-Gln in *H. menziesii* (unique to this genome), and the putative duplication of tRNA-Glu in *C. monodonta* between tRNA-Asn and tRNA-Pro. *H. menziesii* M mtDNA has its own peculiarities in the positions of tRNA-Cys, tRNA-Ile, and tRNA-Gln, which are exclusive to it. The position of tRNA-Ser1 in *C. monodonta* M mtDNA is also unique.

Supplementary Table S1 [pages 3-4]. Nucleotide content statistics of *Neotrigonia margaritacea* and all unionid mt genomes in Table 1. Average values for Margaritiferidae and Unionidae F and Unionidae M mtDNAs (avg margFH, avg unioFH, and avg unioM, respectively), with the respective standard deviation, are also indicated. AT and GC skews are calculated as follows: AT-skew = (A-T)/(A+T), GC-skew = (G-C)/(G+C). mtDNA type is indicated only for DUI unionids or the secondarily hermaphroditic species. All these statistics are summarized in Supplementary Fig. S2. Since *Anodontites trapesialis* mt genome is missing two short segments, the statistics we present for it may slightly deviate from its true nucleotide composition. All unionid mtDNAs are rich in A (between 34.5% and 40.0%) and poor in G (between 10.8% and 13.7%), while the relative proportions of T and C are variable (from 20.4% to 28.9% and from 21.3% to 30.3%, respectively).

Family	Species	mtDNA type	Accession number	A	T	C	G	AT	CG	AG	CT	AT/CG	AG/CT	AT skew	GC skew
Trigonidae	*Neotrigonia margaritacea*	-	KU873118	0.364	0.275	0.232	0.130	0.639	0.362	0.494	0.507	1.765	0.974	0.139	-0.282
Iridinidae	*Mutela dubia*	-	KU873120	0.389	0.248	0.241	0.122	0.637	0.363	0.511	0.489	1.755	1.045	0.221	-0.328
Mycetopodidae	*Anodontites trapesialis*	-	KU873119	0.398	0.242	0.251	0.108	0.640	0.359	0.506	0.493	1.783	1.026	0.244	-0.398
Hyriidae	*Hyridella menziesii*	F	KU873121	0.386	0.214	0.282	0.118	0.600	0.400	0.504	0.496	1.500	1.016	0.287	-0.410
	Hyridella menziesii	M	KU873122	0.375	0.208	0.287	0.130	0.583	0.417	0.505	0.495	1.398	1.020	0.286	-0.376
Margaritiferidae	*Cumberlandia monodonta*	F	KU873123	0.382	0.213	0.278	0.128	0.595	0.406	0.510	0.491	1.466	1.039	0.284	-0.369
	Margaritifera falcata	H	HM856634	0.378	0.239	0.252	0.131	0.617	0.383	0.509	0.491	1.611	1.037	0.225	-0.316
	Cumberlandia monodonta	M	KU873124	0.373	0.204	0.303	0.120	0.577	0.423	0.493	0.507	1.364	0.972	0.293	-0.433
Unionidae	*Anodonta anatina*	F	KF030964	0.381	0.279	0.221	0.119	0.660	0.340	0.500	0.500	1.941	1.000	0.155	-0.300
	Anodonta woodiana	F	HQ283344	0.378	0.280	0.223	0.118	0.658	0.341	0.496	0.503	1.930	0.986	0.149	-0.308
	Cristaria plicata	F	FJ986302	0.365	0.272	0.232	0.130	0.637	0.362	0.495	0.504	1.760	0.982	0.146	-0.282
	Hyriopsis cumingii	F	FJ529186	0.371	0.231	0.270	0.127	0.602	0.397	0.498	0.501	1.516	0.994	0.233	-0.360
	Hyriopsis schlegelii	F	HQ641406	0.371	0.232	0.268	0.129	0.603	0.397	0.500	0.500	1.519	1.000	0.231	-0.350
	Inversidens japanensis	F	AB055625	0.345	0.227	0.291	0.137	0.572	0.428	0.482	0.518	1.336	0.931	0.206	-0.360
	Lamprotula tortuosa	F	KC109779	0.376	0.261	0.236	0.126	0.637	0.362	0.502	0.497	1.760	1.010	0.181	-0.304
	Lampsilis ornata	F	AY365193	0.382	0.242	0.259	0.117	0.624	0.376	0.499	0.501	1.660	0.996	0.224	-0.378
	Pyganodon grandis	F	FJ809754	0.368	0.275	0.229	0.129	0.643	0.358	0.497	0.504	1.796	0.986	0.145	-0.279
	Quadrula quadrula	F	FJ809750	0.392	0.234	0.259	0.115	0.626	0.374	0.507	0.493	1.674	1.028	0.252	-0.385
	Solenaia carinatus	F	KC848654	0.370	0.239	0.271	0.120	0.609	0.391	0.490	0.510	1.558	0.961	0.215	-0.386
	Solenaia oleivora	F	KF296320	0.365	0.234	0.275	0.126	0.599	0.401	0.491	0.509	1.494	0.965	0.219	-0.372
	Unio pictorum	F	HM014130	0.386	0.265	0.231	0.118	0.651	0.349	0.504	0.496	1.865	1.016	0.186	-0.324

Family	Species	mtDNA type	Accession number	A	T	C	G	AT	CG	AG	CT	AT/CG	AG/CT	AT skew	GC skew
Unionidae	*Utterbackia peninsularis*	F	HM856636	0.370	0.275	0.225	0.129	0.645	0.354	0.499	0.500	1.822	0.998	0.147	-0.271
	Venustaconcha ellipsiformis	F	FJ809753	0.381	0.244	0.257	0.117	0.625	0.374	0.498	0.501	1.671	0.994	0.219	-0.374
	Lasmigona compressa	H	HM856638	0.380	0.289	0.213	0.118	0.669	0.331	0.498	0.502	2.021	0.992	0.136	-0.287
	Lasmigona subviridis	H	HM856640	0.385	0.280	0.213	0.121	0.665	0.334	0.506	0.493	1.991	1.026	0.158	-0.275
	Toxolasma parvus	H	HM856639	0.377	0.241	0.262	0.121	0.618	0.383	0.498	0.503	1.614	0.990	0.220	-0.368
	Utterbackia imbecillis	H	HM856637	0.379	0.280	0.218	0.122	0.659	0.340	0.501	0.498	1.938	1.006	0.150	-0.282
	Inversidens japanensis	M	AB055624	0.357	0.214	0.293	0.136	0.571	0.429	0.493	0.507	1.331	0.972	0.250	-0.366
	Pyganodon grandis	M	FJ809755	0.361	0.287	0.225	0.128	0.648	0.353	0.489	0.512	1.836	0.955	0.114	-0.275
	Quadrula quadrula	M	FJ809751	0.400	0.221	0.269	0.110	0.621	0.379	0.510	0.490	1.639	1.041	0.288	-0.420
	Solenaia carinatus	M	KC848655	0.387	0.223	0.269	0.121	0.610	0.390	0.508	0.492	1.564	1.033	0.269	-0.379
	Utterbackia peninsularis	M	HM856635	0.368	0.282	0.220	0.129	0.650	0.349	0.497	0.502	1.862	0.990	0.132	-0.261
	Venustaconcha ellipsiformis	M	FJ809752	0.392	0.243	0.252	0.113	0.635	0.365	0.505	0.495	1.740	1.020	0.235	-0.381
avg margFH				0.380 ± 0.003	0.226 ± 0.018	0.265 ± 0.018	0.130 ± 0.002	0.606 ± 0.016	0.395 ± 0.016	0.510 ± 0.001	0.491 ± 0.000	1.538 ± 0.103	1.038 ± 0.001	0.255 ± 0.042	-0.343 ± 0.038
avg unioFH				0.375 ± 0.010	0.257 ± 0.021	0.245 ± 0.024	0.123 ± 0.006	0.632 ± 0.027	0.368 ± 0.027	0.498 ± 0.006	0.502 ± 0.006	1.730 ± 0.193	0.993 ± 0.023	0.188 ± 0.038	-0.329 ± 0.043
avg unioM				0.378 ± 0.018	0.245 ± 0.032	0.255 ± 0.028	0.123 ± 0.010	0.623 ± 0.030	0.378 ± 0.030	0.500 ± 0.009	0.500 ± 0.009	1.662 ± 0.198	1.002 ± 0.035	0.215 ± 0.073	-0.347 ± 0.064

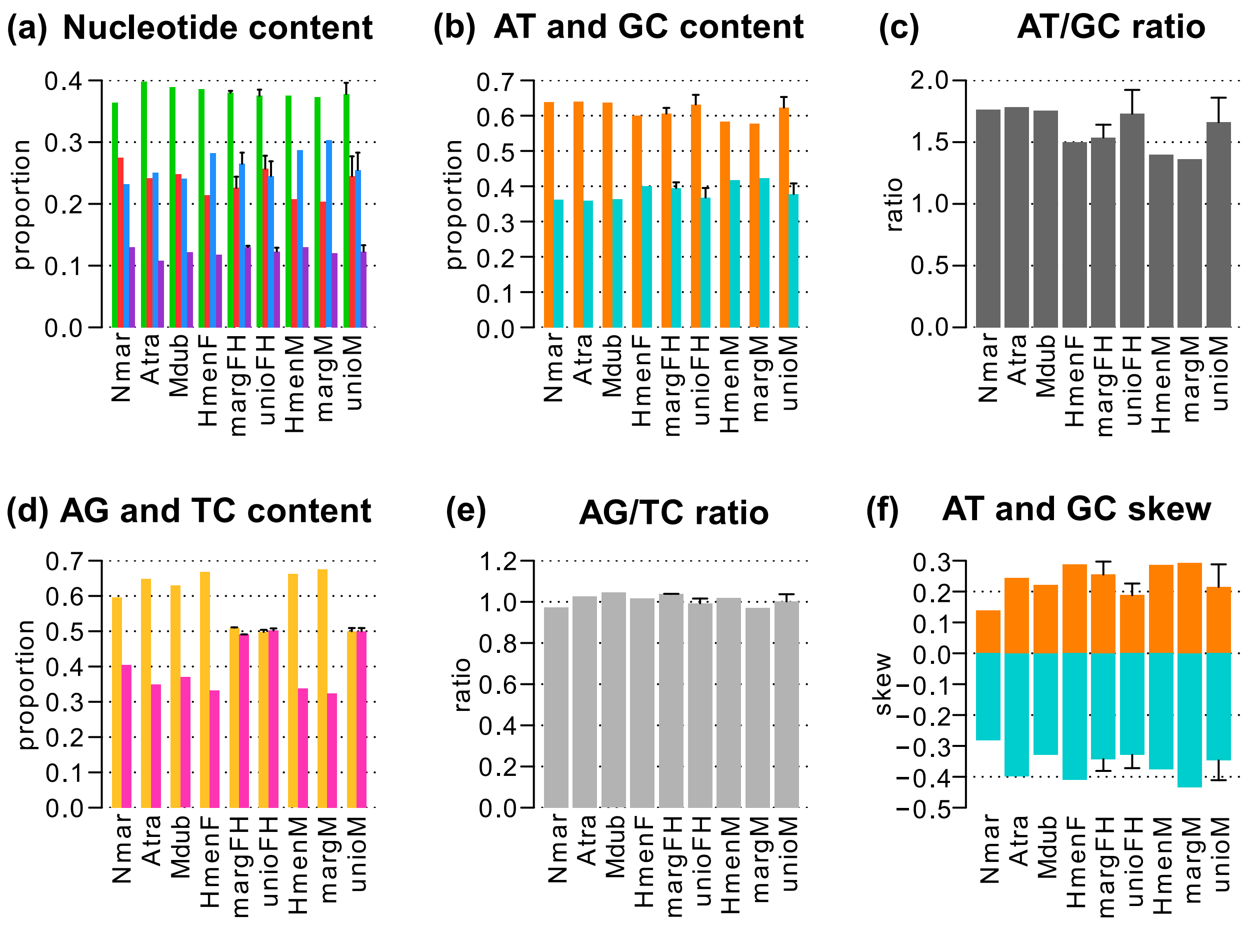

Supplementary Figure S2. Statistics of nucleotide content in Supplementary Table S1. Abbreviations: Nmar, *N. margaritacea*; Atra, *A. trapesialis*; Mdub, *M. dubia*; HmenF and HmenM, *H. menziesii* F and M mtDNA respectively; margFH and margM, average values for Margaritiferidae F plus H and M mtDNA, respectively; unioFH and unioM, average value for Unionidae F plus H and M mtDNAs, respectively. Standard deviation for margFH, unioFH, and unioM values is indicated as whiskers on the respective bars in each panel. (a) Single nucleotide contents expressed as proportion: green, A; red, T; blue, C; purple, G. (b) AT (orange bars) and GC (light blue bars) content. (c) Ratios between AT and GC. (d) Purine (AG, yellow) and pyrimidine (TC, pink) content. (e) Ratios between purine (AG) and pyrimidine (TC) content. (f) AT- and GC-skew values (orange and light blue, respectively). Comparing the simple nucleotide contents in panel (a), *N.*

margaritacea profile is clearly distinct from those of Unionida, but its C content is more similar to those of the etherioid *A. trapesialis* and *M. dubia* (respectively 23.2%, 25.1%, and 24.8%; Supplementary Table S1). T and C contents in Etherioidea differ only by 0.7-0.9%, which distinguishes them from the other freshwater mussels, where the difference is more pronounced. The M and F mt genomes of *H. menziesii* and margaritiferids (this last group comprises also the H mtDNA of *Margaritifera falcata*; see Table 1) are more similar between them than to those of Unionidae, as the latter have more T and less C on average. Using AT and CG contents (panel (b)) and their ratios (panel (c)), the profiles of *N. margaritacea*, *A. trapesialis*, and *M. dubia* are more AT-rich and almost identical to one another, and they can be clearly distinguished from the nucleotide composition patterns of the other groups, especially from *H. menziesii* and margaritiferids. Slight differences between F and M mtDNAs of Hyrioidea and Unionoidea (the latter group comprising also H mtDNAs among their F mt genomes; see Table 1) are evident, with the egg-transmitted mtDNAs being more AT-rich than the sperm-transmitted ones. The averages in the ratio profiles for Unionidae mtDNAs are however more similar to *N. margaritacea*, *A. trapesialis*, and *M. dubia* than to the other two groups. An excess of purines (A and G) over pyrimidines (T and C) in the heavy strand is observed in all considered mitochondrial genomes (panels (d) and (e)), with the exception of Unionidae mtDNAs where the two quantities are overall almost perfectly balanced (average AG/TC ratio $\pm$ SD in F+H and M: 0.993 $\pm$ 0.023 and 1.002 $\pm$ 0.035, respectively; Supplementary Table S1). Finally, given the aforementioned characteristics, *N. margaritacea* shows the lowest absolute AT- and GC-skew values compared to freshwater mussels (panel (f)). *H. menziesii* and margaritiferid mtDNAs are somewhat different from all other Unionida mitochondrial genomes, as they have the highest AT-skew and very low GC-skew values. However, *A. trapesialis* AT-skew is comparable to F+H Margaritiferidae mtDNAs, and its GC-skew is similar to *H. menziesii* F.

Supplementary Table S2 [pages 7-9]. Codon usage statistics. Codon usage is expressed as relative synonymous codon usage (RSCU) for the standard 13 protein coding genes in the seven mt genomes sequenced in this study. RSCU values were calculated with MEGA5 using the invertebrate mitochondrial genetic code. Amino acids encoded by the codons are indicated with the single-letter IUPAC nomenclature. These statistics are graphically summarized in Supplementary Fig. S3.

amino acid	codon	*N. margaritacea*	*A. trapesialis*	*M. dubia*	*H. menziesii* F	*C. monodonta* F	*H. menziesii* M	*C. monodonta* M
stop	UAA	1.38	1.17	0.92	0.77	0.92	0.92	1.00
	UAG	0.62	0.83	1.08	1.23	1.08	1.08	1.00
A	GCA	1.07	0.78	1.05	0.58	0.90	0.76	0.83
	GCC	0.63	0.69	0.68	0.94	0.89	1.08	1.09
	GCG	0.32	0.34	0.45	0.42	0.33	0.47	0.54
	GCU	1.98	2.19	1.83	2.07	1.88	1.69	1.54
C	UGC	0.35	0.46	0.36	0.45	0.47	0.42	0.30
	UGU	1.65	1.54	1.64	1.55	1.53	1.58	1.70
D	GAC	0.34	0.21	0.41	0.63	0.52	0.66	0.61
	GAU	1.66	1.79	1.59	1.38	1.48	1.34	1.39
E	GAA	1.27	1.04	0.87	0.55	0.66	0.70	0.42
	GAG	0.73	0.96	1.13	1.45	1.34	1.30	1.58
F	UUC	0.32	0.42	0.37	0.42	0.41	0.36	0.42
	UUU	1.68	1.58	1.63	1.58	1.59	1.64	1.58
G	GGA	1.39	0.85	0.99	0.44	0.51	0.42	0.52
	GGC	0.16	0.15	0.31	0.38	0.49	0.53	0.25
	GGG	1.14	1.35	1.50	1.92	1.67	1.90	2.02
	GGU	1.32	1.65	1.19	1.26	1.33	1.15	1.21
H	CAC	0.33	0.34	0.52	0.52	0.63	0.79	0.79
	CAU	1.67	1.66	1.48	1.48	1.37	1.21	1.21
I	AUC	0.31	0.62	0.44	0.57	0.56	0.62	0.66
	AUU	1.69	1.38	1.56	1.43	1.44	1.38	1.34
K	AAA	1.38	1.22	1.18	0.97	1.13	0.94	1.17

amino acid	codon	***N. margaritacea***	***A. trapesialis***	***M. dubia***	***H. menziesii* F**	***C. monodonta* F**	***H. menziesii* M**	***C. monodonta* M**
K	AAG	0.62	0.78	0.82	1.03	0.87	1.06	0.83
L	CUA	0.65	0.71	0.80	0.88	1.01	1.03	0.89
	CUC	0.24	0.34	0.27	0.37	0.33	0.41	0.61
	CUG	0.15	0.13	0.15	0.27	0.34	0.49	0.25
	CUU	0.97	0.79	0.96	0.87	0.85	0.91	0.88
	UUA	2.46	2.44	2.20	1.60	1.22	1.38	1.27
	UUG	1.53	1.59	1.61	2.00	2.24	1.79	2.09
M	AUA	1.06	1.41	1.31	1.02	0.95	1.31	0.99
	AUG	0.94	0.59	0.69	0.98	1.05	0.69	1.01
N	AAC	0.49	0.71	0.70	1.16	0.76	1.26	0.86
	AAU	1.51	1.29	1.30	0.84	1.24	0.74	1.14
P	CCA	1.45	1.47	2.11	1.06	1.59	1.09	1.43
	CCC	0.42	0.40	0.49	0.47	0.46	0.87	0.61
	CCG	0.53	0.46	0.34	0.29	0.55	0.54	0.48
	CCU	1.61	1.68	1.06	2.18	1.39	1.50	1.48
Q	CAA	1.35	1.45	1.27	1.18	1.06	1.19	1.07
	CAG	0.65	0.55	0.73	0.82	0.94	0.81	0.93
R	CGA	1.06	1.40	1.60	1.27	1.23	0.74	1.09
	CGC	0.18	0.21	0.27	0.48	0.74	0.31	0.16
	CGG	0.94	1.12	0.80	0.97	0.68	0.92	1.25
	CGU	1.82	1.26	1.33	1.27	1.35	2.03	1.51
S	AGA	1.82	0.81	1.21	0.68	0.82	0.46	0.69
	AGC	0.17	0.19	0.25	0.52	0.38	0.76	0.50
	AGG	0.86	1.44	1.01	1.77	1.38	1.38	1.38
	AGU	0.92	1.00	0.91	0.88	1.11	1.06	1.23
	UCA	0.86	1.30	1.78	0.73	1.01	0.62	1.02
	UCC	0.39	0.58	0.55	0.63	0.80	0.84	0.78
	UCG	0.26	0.32	0.43	0.36	0.38	0.52	0.35

amino acid	codon	***N. margaritacea***	***A. trapesialis***	***M. dubia***	***H. menziesii* F**	***C. monodonta* F**	***H. menziesii* M**	***C. monodonta* M**
S	UCU	2.72	2.37	1.85	2.42	2.14	2.35	2.05
T	ACA	0.89	1.15	1.36	1.28	1.24	1.41	1.12
	ACC	0.70	0.96	1.11	1.13	0.88	1.19	1.06
	ACG	0.29	0.37	0.25	0.21	0.41	0.38	0.45
	ACU	2.12	1.52	1.27	1.38	1.46	1.01	1.38
V	GUA	1.22	0.92	1.06	0.56	0.72	0.44	0.59
	GUC	0.15	0.23	0.21	0.37	0.32	0.36	0.22
	GUG	0.86	0.87	0.62	0.98	0.94	1.11	1.26
	GUU	1.76	1.98	2.12	2.08	2.02	2.09	1.92
W	UGA	1.06	1.02	0.87	0.60	0.83	0.65	0.64
	UGG	0.94	0.98	1.13	1.40	1.17	1.35	1.36
Y	UAC	0.56	0.55	0.57	0.65	0.82	0.77	0.64
	UAU	1.44	1.45	1.43	1.35	1.18	1.23	1.36

Supplementary Figure S3 [pages 10-11]. Codon usage. Codon usage is expressed as RSCU (relative synonymous codon usage). Y axis proportions vary in each panel. Boxplots describe the overall distribution of values for each codon. Symbols legend: ■, *Neotrigonia margaritacea*; ▲, *Anodontites trapesialis*; ▼, *Mutela dubia*; ▲, *Hyridella menziesii* F; ▲, *Hyridella menziesii* M; ▼, *Cumberlandia mondonta* F; ▼, *Cumberlandia mondonta* M. In 2-fold redundant codons, median RSCU values tend to be higher for codons ending in A or T, although in some cases a species may contain C or G in third position for this kind of codons (e.g., codons for glutamic acid, lysine, TTR leucine, methionine, asparagine, and tryptophan), and this tendency to use those two nucleotides is more evident for *H. menziesii* and *C. monodonta* F and M genomes. In 4-fold redundant codons, all species are more likely to use T in the third codon position, as for alanine, valine, and TCN serine, but in other cases the overall preference is mixed, usually with C and/or G with the lowest usage (as for alanine, glycine, CTN leucine, proline, arginine, and AGN serine). *N. margaritacea* RSCU values tend to be placed at the lower or higher extremes of the distributions, most times being very similar to those of *A. trapesialis* and/or *M. dubia* (GCV alanine, cysteine, aspartic acid, glutamic acid, GGV glycine, histidine, leucine, glutamine, AGV and TCK serine, GTR valine, tryptophane, and tyrosine).

Supplementary Figure S3 [part 1 of 2]

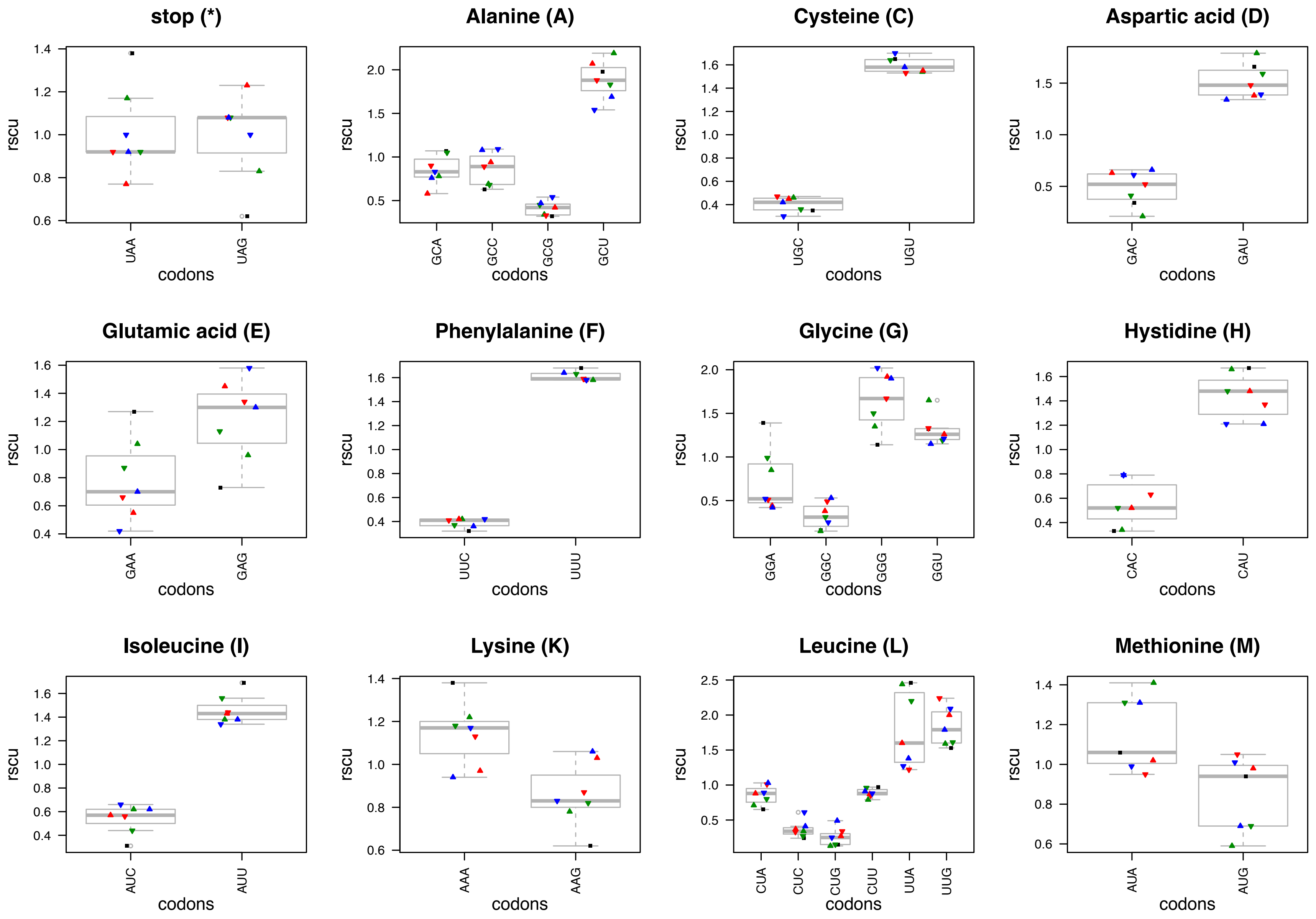

Supplementary Figure S3 [part 2 of 2]

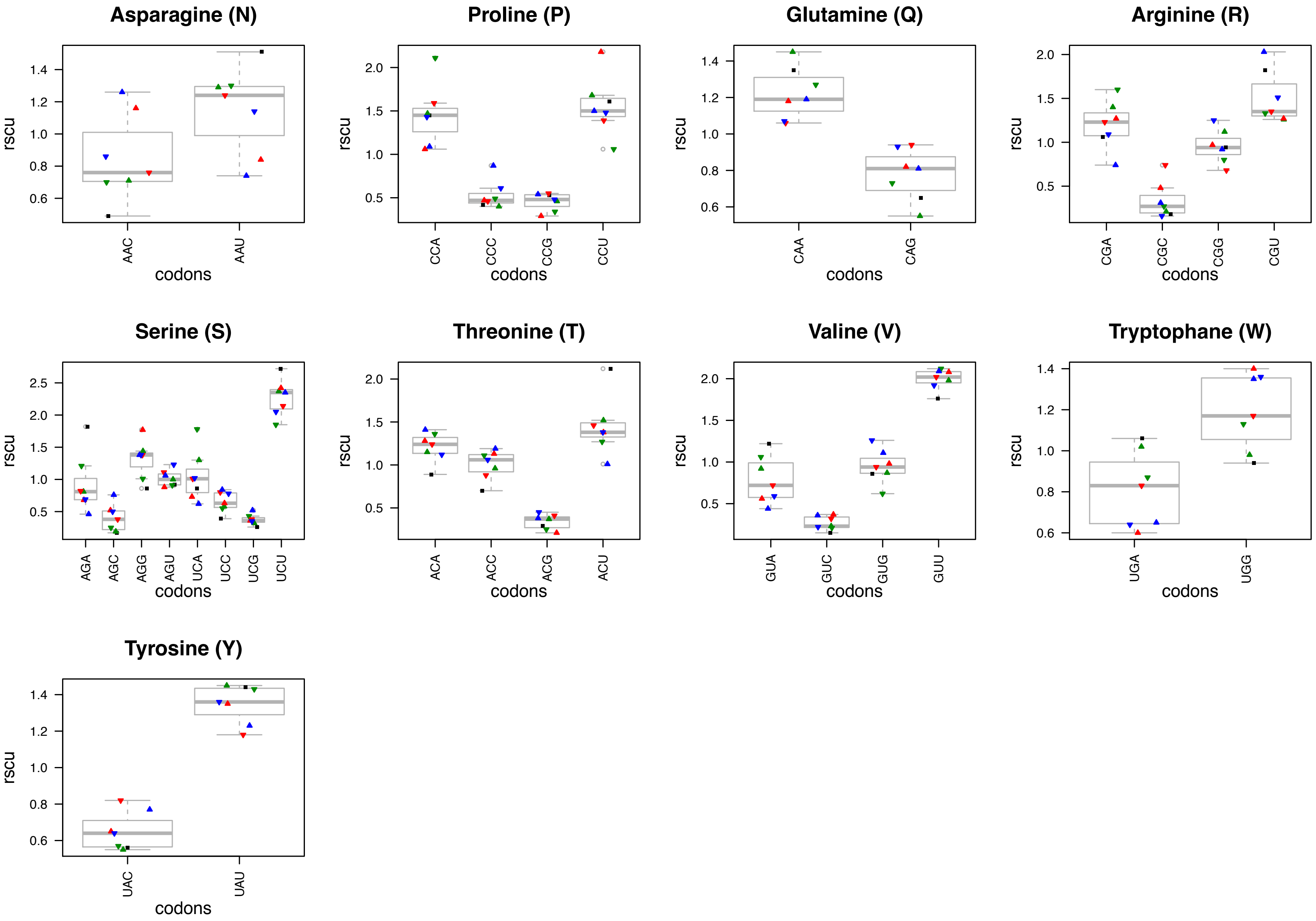

Supplementary Table S3. Usage of start and stop codons in the 13 standard protein coding genes and lineage-specific F-*orf* and M-*orf* of the seven mt genomes sequenced in this study. Codons used as start and stop for F-*orf* and M-*orf* in *H. menziesii* and *C. monodonta* are indicated with the superscripts "F-orf" and "M-orf", respectively; because of these additional genes, the sum of start and stop codons is 14 in the mtDNAs of these two species. Because of a sequencing gap in *A. trapesialis* mt genome, start of *cox2* and end of *nad3* are unknown (see also Fig. 1), therefore only 12 start and stop codons are enlisted for this species. The most widely used start codon is ATG, while use of alternative start codons is variable among species. *A. trapesialis* uses the two standard start codons (ATG and ATA) and the alternative ATT in equal proportions. There is a strong preference in *N. margaritacea* for the TAA stop codon over TAG compared to freshwater mussels, which are more balanced between the two. The only incomplete stop codon (T) is annotated in *C. monodonta* M mtDNA for *nad1*.

Codon type	Codon	*N. margaritacea*	*A. trapesialis*	*M. dubia*	*H. menziesii* F	*C. monodonta* F	*H. menziesii* M	*C. monodonta* M
Start	ATA	1	4	0	1	0	5	1
	ATG	7	4	8	8	10	$7^{M\text{-}orf}$	$5^{M\text{-}orf}$
	ATC	1	0	2	1	0	0	0
	ATT	2	4	1	2	$3^{F\text{-}orf}$	0	3
	GTG	2	0	0	$1^{F\text{-}orf}$	0	0	2
	TTG	0	0	2	1	1	2	3
Stop	TAA	9	7	6	5	$7^{F\text{-}orf}$	6	6
	TAG	4	5	7	$9^{F\text{-}orf}$	7	$8^{M\text{-}orf}$	$7^{M\text{-}orf}$
	T**	0	0	0	0	0	0	1
