# Supplementary material for: Evolution of sex-dependent mtDNA transmission in freshwater mussels (Bivalvia: Unionida)
Source: Sci Rep. 2017 May 8;7:1551. doi: 10.1038/s41598-017-01708-1 (PMC5431520; doi:10.1038/s41598-017-01708-1)
Supplement: Supplementary file 2 — Supplementary Information 2 [file 41598_2017_1708_MOESM2_ESM.pdf]

Evolution of sex-dependent mtDNA transmission in freshwater mussels (Bivalvia: Unionida)

Davide Guerra[1], Federico Plazzi[2], Donald T. Stewart[3], Arthur E. Bogan[4], Walter R. Hoeh[5] & Sophie Breton[1]

[1]Département de Sciences Biologiques, Université de Montréal, Montréal H2V 2S9, Quebéc, Canada. [2]Dipartimento di Scienze Biologiche, Geologiche ed Ambientali (BiGeA), Università di Bologna, Bologna 40126, Italy. [3]Department of Biology, Acadia University, Wolfville B4P 2R6, Nova Scotia, Canada. [4]North Carolina Museum of Natural Sciences, Raleigh, NC 27607, USA. [5]Department of Biological Sciences, Kent State University, Kent, OH 44242, USA.

Supplementary Information 2

Alignments of repeated sequences found in the mt genomes

Symbols legend: -, alignment gap; *, column with only identical nucleotides.

• *Anodontites trapesialis*

Alignment of the three large (310-331bp) tandem repeats between UR21 and *atp6*. R1 contains the start of **Atra_UR_21_9** (bold, blue) and the complete **tRNA-Asp** (bold, purple), R2 contains the whole **Atra_UR_22_18** (bold, red), R3 contains the full ***atp8*** (bold, green) and the end of ***atp6*** (bold, orange). Pseudogenized copies of tRNA-Asp are present in R2 and R3 (highlighted in purple), while pseudogenized fragments of *atp8* and *atp6* (highlighted in green and yellow, respectively) are present in R1 and R2. Fragments of the *atp8* gene are conserved inside Atra_UR_21_9 and Atra_UR_22_18 sequences. Coordinates of repeats on the mtDNA: R1, 12491-12820; R2, 12821-13130; R3, 13131-13461.

```
R1          GGTAAAACAACTCTCAAAAGATTATAAATCCTCCTGACCACCTTCTCCCTAACTAAATTA
R2          -------------------------ATCTTATC-------------CCTTACCTAGATTA
R3          GGTAAAACAACTCTTCAAAGACCATATCCTGTCTTAACTCTTTCCTTCCTACCTAAACTA
                                     *     **              * ** *** * **

R1          AAAAATCCATATTTATACCTATCT--CTACTAAATAAAATTAAACATAAAAAGAATAAT-
R2          CAAAA-------TTATACCTACCTACCTCACAAATAAACTCAAACACACAAAGAATAA--
R3          GAAGAATTATACTTACACCCACC---CCACCAAGTAAACCTAAGCATCAACAGAAAAATA
             ** *       *** *** * *   *    ** ****   ** **   * **** **

R1          AAAAACAAACCACAAC-TGGTACCCATAACCCTAATACAAAATTCAATTGAGTAATAAGA
R2          ACAAACATACCAAGATAAAACACCAACAATTATAACACAAAACCTAAATAAAAATTA--A
R3          AAAAACATACCCTAAT-AAATACCAATAACCATGACATAGGACTCAACTGAGGAATAAGA
            * ***** ***   *      *** * **   * * * *  *   ** * *  * **  *

R1          AAACGCTTTGGTAAGCAATATGAACTCGACAAGCTCAAGTTATATATTTAACTAGTCTTC
R2          AAACACTTTGGTTAACAATACCAACCCAACAATTTTAAGCTATATATTTACTTAATCTTC
R3          AGACGCTTTTACAAGCAATATGAACACAACAAACTCAAATTATACACCTAACTAGTCTTC
            * ** ****    * *****  *** * ****  * **  **** *  **  ** *****

R1          TTCCACAAATATCCATACCATCACTAATATCCAAAAATCGAATTACTACAAAAACACAAC
R2          CTCCATAAATGCTTATACTACCAC------CTAGAAATCAATTTACCACAAAAATACAAC
R3          CTTCACAGTTATCCACACCACTTCTAATGCTTAGAAAGTGAATGATCAGAAGAATACAAC
             * ** *  *    * ** *   *        * ***   * * *  * ** ** *****

R1          CCAAACAAC-AAACGAATACGTAAACTTATAATAA-------------------------
R2          CCAAACAAC-AAACGAACACATAAACCTATAATAAACCTTACTTCTTAATTTTAACACCT
R3          CTCAGCAACAAAACAAACACATAAGCTTGTAAAAA-------------------------
            *  * **** **** ** ** *** * * *** **

R1          ------
R2          TTTGAT
R3          ------
```

• ***Mutela dubia***

Alignment of four short (7-8bp) tandem repeats between the beginning of *nad4* and UR24. R1 and R2 are inside *nad4*, R3 is between *nad4* and UR24, and R4 is completely inside UR24. ***nad4*** parts are in bold and green. Coordinates of repeats on the mtDNA: R1, 13986-13993; R2, 13994-14001; R3, 14002-14009; R4, 14010-14016.

```
R1              AACAAAAA
R2              AAGAAAAA
R3              AACAAAAA
R4              AACAAAA-
                ** ****
```

• ***Cumberlandia monodonta*** **F**

Alignment of two tandem repeats (17bp) found inside *nad3*. Coordinates of repeats on the mtDNA: R1, 2362-2378; R2, 2379-2395.

```
R1              CCAAAGCACAATAACCG
R2              CCAAACCACAATCAACC
                ***** ****** * *
```

• ***Hyridella menziesii*** **M**

Alignment of the two longest repeats (270bp) found inside the M-*orf*. They have an overlap of 171 residues between the end of R1a and the beginning of R1b (underlined nucleotides). Coordinates of repeats on the mtDNA: R1a, 15399-15668; R1b, 15498-15767.

```
R1a_mORF        TCACTTTTTCAGGCTTATTTATAGCCTCTTTTTTTACCTTCTTAGGCTTACCCACAGACT
R1b_mORF        TCACTTTTTCAGGCTCATCTGTAGCCCTCTTCTTTACCTTCTTGGGTTTATCCGCAGGCT
                *************** ** * *****   ** *********** ** *** ** *** **

R1a             CTATCTTTGCCTTTTTAGGCTTACTTACGGCCTCTTTCCTCACTTTTTCAGGCTCATCTG
R1b             CTTTCTTTACCTTCTTAGGCTTATCACCGGCCTCTTTTTTCACCTTTTCAGGCTCATCCG
                ** ***** **** *********    **********  **** ************** *

R1a             TAGCCCTCTTCTTTACCTTCTTGGGTTTATCCGCAGGCTCTTTCTTTACCTTCTTAGGCT
R1b             CAGTTTCCTTCTTTACCTTCTTAGCTTTATTTATAACCTTTTTCTCTACCTCTTCAGGTT
                 **    *************** * *****    *  ** ***** *****  * *** *

R1a             TATCACCGGCCTCTTTTTTCACCTTTTCAGGCTCATCCGCAGTTTCCTTCTTTACCTTCT
R1b             CACCTGTAACCTCCTTCTTTACCTTTTTAGGCTTATCCTTAGTCTCCTTTTTCACTTTCT
                 * *     **** ** ** ******* ***** ****  *** ***** ** ** ****

R1a             TAGCTTTATTTATAACCTTTTTCTCTACCT
R1b             TAGGGCTATCTGTACCTTCTTTATTTACCT
                ***   *** * ** * * *** * *****
```

Alignment of two 47bp repeated sequences found at the beginning and at the end of the M-*orf* repeat region. Coordinates of repeats on the mtDNA: R2a, 15397-15443; R2b, 15727-15773.

```
R2a             TTTCACTTTTTCAGGCTTATTTATAGCCTCTTTTTTTACCTTCTTAG
R2b             TTTCACTTTCTTAGGGCTATCTGTACCTTCTTTATTTACCTTCTTAG
                ********* * ***  *** * ** * ***** *************
```

Alignment of two 99bp tandem repeats found inside the M-*orf* repeat region. Coordinates of repeats on the mtDNA: R3a, 15399-15497; R3b, 15498-15596.

```
R3a           TCACTTTTTCAGGCTTATTTATAGCCTCTTTTTTTACCTTCTTAGGCTTACCCACAGACT
R3b           TCACTTTTTCAGGCTCATCTGTAGCCCTCTTCTTTACCTTCTTGGGTTTATCCGCAGGCT
              *************** ** * *****   ** *********** ** *** ** *** **

R3a           CTATCTTTGCCTTTTTAGGCTTACTTACGGCCTCTTTCC
R3b           CTTTCTTTACCTTCTTAGGCTTATCACCGGCCTCTTTTT
              ** ***** **** *********    **********
```

Alignments of six tandem repeats (34-25bp) found inside the M-*orf* repeat region. Coordinates of repeats on the mtDNA: R4a, 15422-15454; R4b, 15455-15487; R4c, 15488-15520; R4d, 15521-15554; R4e, 15555-15586; R4f, 15587-15611.

```
R4a           GCCTCTTTTTTTACCTTCTTAGGCTTACCCACA-
R4b           GACTCTATCTTTGCCTTTTTAGGCTTACTTACG-
R4c           GCCTCTTTCCTCACTTTTTCAGGCTCATCTGTA-
R4d           GCCCTCTTCTTTACCTTCTTGGGTTTATCCGCAG
R4e           G-CTCTTTCTTTACCTTCTTAGGCTTATCACCG-
R4f           GCCTCTTTTTTCACCTTTTCAGGCT---------
              * *    *  *  * ** *  ** *
```

Alignment of two 99bp tandem repeats found inside the M-*orf* repeat region. Coordinates of repeats on the mtDNA: R5a, 15431-15529; R5b, 15530-15628.

```
R5a           TTTACCTTCTTAGGCTTACCCACAGACTCTATCTTTGCCTTTTTAGGCTTACTTACGGCC
R5b           TTTACCTTCTTGGGTTTATCCGCAGGCTCTTTCTTTACCTTCTTAGGCTTATCACCGGCC
              *********** ** *** ** *** **** ***** **** *********    *****

R5a           TCTTTCCTCACTTTTTCAGGCTCATCTGTAGCCCTCTTC
R5b           TCTTTTTTCACCTTTTCAGGCTCATCCGCAGTTTCCTTC
              *****  **** ************** * **    ****
```

Alignment of two tandem repeats (22 and 25bp) inside *nad3*. Coordinates of repeats on the mtDNA: R1_nad3, 3096-3120; R2_nad3, 3121-3142.

```
R1_nad3       ACCACCACAAACCCGATAACCCAGT
R2_nad3       ACCACC-CAAACCCACACACCCA--
              ****** *******    *****
```

Alignment of the two identical tandem repeats (34bp each) inside 16S rRNA gene. Coordinates of repeats on the mtDNA: R1_16S, 7858-7891; R2_16S, 7892-7925.

```
R1_16S        AAACTGGGTTTGCGACCTCGATGTTGGCTTAGGG
R2_16S        AAACTGGGTTTGCGACCTCGATGTTGGCTTAGGG
              **********************************
```

Alignment of two regions shared between UR3 and UR17 (100bp and 98bp, respectively). Coordinates of the shared regions on the mtDNA: S1, 3902-3999; S2, 11540-11639.

```
S1                GAGCCCGCGGGAATCCCGCCTCTACAGGGCAGCCCACGACCGCCAGCCCATTGTCTGCCA
S2                GAGCCCGCGGGAATCCCGCCTCCACAGGGCAGCCCACGACCGCCAGCCCATTGTCTGCCA
                  ********************** *************************************

S1                GCTCC-A-CCACCCTATTTTATATAGTAGTCCTATTTAAT
S2                GCTCCCACCCCCCCTATTTTTTAGCGTAAACCTATTTAAT
                  ***** * ** ********* **  ***  **********
```

Alignment of an imperfect palindrome sequence (153bp) found inside UR3 with its reverse complement. Given the palindromic nature of this segment, it might possibly form a hairpin 2D structure. The palindrome midpoint is in bold. Coordinates of the palindrome in the mtDNA: 3556-3708.

```
palindrome            AGGGAAGTAGAGATACCAGAGG--GGCCCCCCGGGAGGG-TCATTATGAGAAGCCTTAAA
palindrome_revcomp    AGTGAAGTAGAGATAGTGGAGGAAGGCCTTTAGGGAGGGATTATTA-GAGAAGCC-CAGA
                      ** ************   ****  ****    ******* * **** ********  * *

palindrome            CACTCTCGGGGGCTCCCTAAAGAAGGGCCCCCGGAGTGTCT-GGGCTTCTC-TAATAATC
palindrome_revcomp    CACTCCGGGGGCCCTTCTTTAGGGAGCCCCCGAGAGTGTTTAAGGCTTCTCATAATGA-C
                      *****  **** *   **  **   * ****  ****** *  ******** **** * *

palindrome            CCTCCCTAAAGGCCTTCCTCCACTATCTCTACTTCACT
palindrome_revcomp    CCTCCCGGGGGGCC--CCTCTGGTATCTCTACTTCCCT
                      ******    ****  ****   ************ **
```

• ***Cumberlandia monodonta* M**

Alignment of the two 210bp repeats between tRNA-Asp and UR16. R1 contains the beginning of **tRNA-Asp** (bold, purple). Both repeats contain pseudogenized fragments of tRNA-Asp (highlighted in purple). The 16 nucleotides overlap between the end of R1 and the beginning of R2 is underlined. Coordinates of repeats on the mtDNA: R1, 10755-10964; R2, 10949-11158.

```
R1                TCTTTTTCCCATCTAAACTACCTTGCAAAGAAACCCTTAATAGGCTATAACAAGCGCTTA
R2                TCTTTTTATACTTTAAACTACCTTGCAAAGAAACCCTTAATAGGCTATAACAAGCGCTTA
                  *******    * ***********************************************

R1                ATCTTGCCCACATAAAACTAATGGGTTAATAACCTAAAGTCCTCATAAATACTCTAACTT
R2                ATCTTGCCCACATAAAACTAATGGGTTAATAACCTAAAGTCCTCATAAATACTCTAACTT
                  ************************************************************

R1                ATCTACGCTTGCTTTATGTATGACAGTTACTCTAACTGATTTAGTTACTCTTTATAGGGT
R2                ATCTACGCTTGCTTTATGTATGACAGTTACTCTAACTGATTTAGTTACTCTTTATAGGGT
                  ************************************************************

R1                TCTCCATCCTTCTCTCTTTTTATACTTTAA
R2                TCTCCATCCTTCTCTCTTTTTATACTTTAA
                  ******************************
```

Alignment of the two repeats inside 16S rRNA gene. Coordinates of repeats on the mtDNA: R1, 7121-7155; R2, 7156-7189.

```
R1                GAAAGCTGGGTTTGCGACCTCGATGTTGGCTTAGG
R2                GAAA-CTGGGTTTGCGACCTCGATGTTGGCTTAGG
                  **** ******************************
```
