# Supplementary material for: Evolution of sex-dependent mtDNA transmission in freshwater mussels (Bivalvia: Unionida)
Source: Sci Rep. 2017 May 8;7:1551. doi: 10.1038/s41598-017-01708-1 (PMC5431520; doi:10.1038/s41598-017-01708-1)
Supplement: Supplementary file 3 — Supplementary Information 3 [file 41598_2017_1708_MOESM3_ESM.pdf]

Evolution of sex-dependent mtDNA transmission in freshwater mussels (Bivalvia: Unionida)

Davide Guerra[1], Federico Plazzi[2], Donald T. Stewart[3], Arthur E. Bogan[4], Walter R. Hoeh[5] & Sophie Breton[1]

[1]Département de Sciences Biologiques, Université de Montréal, Montréal H2V 2S9, Quebéc, Canada. [2]Dipartimento di Scienze Biologiche, Geologiche ed Ambientali (BiGeA), Università di Bologna, Bologna 40126, Italy. [3]Department of Biology, Acadia University, Wolfville B4P 2R6, Nova Scotia, Canada. [4]North Carolina Museum of Natural Sciences, Raleigh, NC 27607, USA. [5]Department of Biological Sciences, Kent State University, Kent, OH 44242, USA.

Supplementary Information 3

Pairwise distances, d_N and d_S analyses

(Supplementary Tables S4, S5)

Supplementary Table S4. Pairwise distances and statistics for rates of synonymous and non-synonymous substitutions in the comparison between F and M mtDNA-encoded genes for *H. menziesii* and *C. monodonta*. Pairwise distances (p-D) are calculated both for protein coding genes (PCGs) and their respective proteins. Synonymous (d_S) and non-synonymous (d_N) substitution statistics are calculated only for PCGs. Average values of the statistics for PCGs are accompanied by the respective standard deviations (SD). For 12S and 16S rRNA genes, only nucleotide level p-D are calculated. d_S values are higher than d_N ones in every comparison, resulting in d_N/d_S ratios always <1.

Species	Statistic	*atp6*	*atp8*	*cox1*	*cox2*	*cox3*	*cytb*	*nad1*	*nad2*	*nad3*	*nad4*	*nad4L*	*nad5*	*nad6*	average ± SD	12S	16S
H. menziesii	amino acid p-D	0.522	0.432	0.307	0.442	0.418	0.360	0.458	0.596	0.409	0.497	0.589	0.538	0.663	0.479 ± 0.101		
	nucleotide p-D	0.434	0.351	0.296	0.402	0.345	0.351	0.400	0.484	0.307	0.411	0.434	0.427	0.528	0.398 ± 0.067	0.306	0.270
	d_N	0.495	0.334	0.244	0.424	0.350	0.302	0.377	0.609	0.309	0.466	0.535	0.501	0.762	0.439 ± 0.143		
	d_S	1.389	0.923	1.084	1.384	1.029	1.508	2.083	1.780	0.800	1.253	1.198	1.277	1.792	1.346 ± 0.370		
	d_N/d_S	0.356	0.362	0.225	0.306	0.340	0.200	0.181	0.342	0.386	0.372	0.447	0.392	0.425	0.333 ± 0.084		
C. monodonta	amino acid p-D	0.498	0.649	0.298	0.425	0.424	0.295	0.451	0.586	0.508	0.497	0.609	0.523	0.604	0.490 ± 0.111		
	nucleotide p-D	0.420	0.540	0.278	0.370	0.364	0.293	0.413	0.474	0.392	0.409	0.401	0.436	0.502	0.407 ± 0.074	0.273	0.262
	d_N	0.475	0.900	0.222	0.377	0.373	0.209	0.407	0.628	0.464	0.461	0.521	0.544	0.681	0.482 ± 0.185		
	d_S	1.276	1.265	0.968	1.226	1.161	1.352	1.923	1.258	1.008	1.202	0.817	1.144	1.596	1.246 ± 0.279		
	d_N/d_S	0.372	0.711	0.229	0.308	0.321	0.155	0.212	0.499	0.460	0.384	0.638	0.476	0.427	0.399 ± 0.162		

Supplementary Table S5 [pages 3-7]. Pairwise distance, rates of synonymous and non-synonymous substitutions, and their ratios, for interspecific gene and protein comparisons among the seven mt genomes sequenced in this study. Abbreviations: p-D, pairwise distance; d_N, synonymous substitutions; d_S, non-synonymous substitutions. Values were calculated with MEGA5 (using the invertebrate mitochondrial genetic code for d_N and d_S). p-D were calculated both for PCGs and their respective proteins. Average values of the statistics are accompanied by the respective standard deviations (SD). When a value of d_N or d_S could not be calculated, this is indicated as n/c; when a d_N/d_S ratio could not be calculated because of this, it is indicated with na ("not applicable"). Values in blue and red are respectively the lowest and highest in a row (average and SD are excluded). Light blue and orange cells highlight respectively the lowest and highest values in a column (not applied to SD). The genomes having the lowest p-D in their respective pairwise comparisons are the F mtDNAs of *H. menziesii* and *C. monodonta* (overall nucleotide and protein p-distances ± SD: 0.291 ± 0.076 and 0.293 ± 0.138, respectively), followed by those of *A. trapesialis* and *M. dubia* (overall nucleotide and protein p-distances ± SD: 0.298 ± 0.125 and 0.306 ± 0.065, respectively); this is visible also in single gene/protein comparisons, where these two couples of mtDNAs always have the lowest p-D value. The M mt genomes of *H. menziesii* and *C. monodonta* have an overall nucleotide p-D ± SD of 0.390 ± 0.055 between them, and an overall protein p-D ± SD of 0.479 ± 0.098. Despite the structural similarities of *A. trapesialis* mtDNA with the M mtDNAs of unionids (see below), the overall divergences between it and *H. menziesii* and *C. monodonta* M mt genomes are respectively 41.1% and 48.3% for nucleotide sequences, and 40.7% and 49.0% for amino acids. The high similarity observable between *A. trapesialis cox2* with those of *H. menziesii* and *C. monodonta* M mtDNAs can be an effect of the short segment available for the analyses in this species; similarly, the highest p-D values in the comparison between *H. menziesii* M*cox2* and *C. monodonta* M*cox2* is the outcome of the original alignments of the two sequences, which comprise the long, highly variable, terminal elongation. The lowest overall d_N value belongs to the comparison between the F mtDNAs of *H. menziesii* and *C. monodonta* (overall d_N ± SD: 0.237 ± 0.135), which also has the lowest value for 9 out of 13 genes, while the highest to that of *M. dubia* and *C. monodonta* M (overall d_N ± SD: 0.499 ± 0.178). The comparison between the M of *H. menziesii* and *C. monodonta* has the lowest overall d_S value (overall d_S ± SD: 1.183 ± 0.265), and the highest belongs to the comparison between *N. margaritacea* and *H. menziesii* M (overall d_S ± SD: 1.782 ± 0.499). The comparison between *A. trapesialis* and *M. dubia* has the lowest overall d_N/d_S value (d_N/d_S ± SD: 0.195 ± 0.100), while that between *H. menziesii* M and *C. monodonta* F has the highest (overall d_N/d_S ± SD: 0.434 ± 0.278).

mtDNAs	Statistic	*atp6*	*atp8*	*cox1*	*cox2*	*cox3*	*cytb*	*nad1*	*nad2*	*nad3*	*nad4*	*nad4L*	*nad5*	*nad6*	average	SD
N. margaritacea vs *A. trapesialis*	amino acid p-D	0.481	0.641	0.150	0.268	0.229	0.358	0.371	0.555	0.333	0.433	0.516	0.448	0.500	0.406	0.139
	nucleotide p-D	0.428	0.542	0.233	0.269	0.264	0.356	0.377	0.484	0.292	0.375	0.403	0.386	0.417	0.371	0.089
	d_N	0.479	0.784	0.118	0.204	0.172	0.296	0.328	0.582	0.243	0.397	0.442	0.393	0.441	0.375	0.181
	d_S	1.492	2.408	1.311	0.953	1.305	2.147	2.228	3.011	1.082	1.125	1.363	1.422	1.904	1.673	0.615
	d_N/d_S	0.321	0.326	0.090	0.214	0.132	0.138	0.147	0.193	0.225	0.353	0.324	0.276	0.232	0.229	0.086

mtDNAs	Statistic	*atp6*	*atp8*	*cox1*	*cox2*	*cox3*	*cytb*	*nad1*	*nad2*	*nad3*	*nad4*	*nad4L*	*nad5*	*nad6*	average	SD
N. margaritacea vs *M. dubia*	amino acid p-D	0.470	0.593	0.158	0.280	0.208	0.311	0.388	0.607	0.431	0.475	0.490	0.451	0.512	0.413	0.139
	nucleotide p-D	0.412	0.455	0.242	0.311	0.258	0.337	0.359	0.474	0.410	0.407	0.414	0.392	0.461	0.379	0.074
	d_N	0.460	0.617	0.124	0.226	0.156	0.244	0.310	0.579	0.406	0.456	0.470	0.397	0.496	0.380	0.156
	d_S	1.230	0.999	1.518	1.755	1.384	2.939	1.769	2.030	n/c	1.270	1.352	1.538	n/c	1.617	0.525
	d_N/d_S	0.374	0.618	0.082	0.129	0.113	0.083	0.175	0.285	na	0.359	0.348	0.258	na	0.257	0.164
N. margaritacea vs *H. menziesii* F	amino acid p-D	0.457	0.480	0.115	0.281	0.178	0.258	0.301	0.522	0.368	0.406	0.411	0.396	0.543	0.363	0.130
	nucleotide p-D	0.399	0.458	0.211	0.296	0.256	0.323	0.330	0.453	0.357	0.361	0.333	0.365	0.469	0.355	0.077
	d_N	0.419	0.528	0.088	0.215	0.131	0.194	0.236	0.486	0.312	0.364	0.314	0.335	0.543	0.320	0.146
	d_S	1.379	1.677	1.263	1.352	2.011	n/c	2.169	n/c	1.845	1.106	1.108	1.605	2.586	1.646	0.471
	d_N/d_S	0.304	0.315	0.070	0.159	0.065	na	0.109	na	0.169	0.329	0.283	0.209	0.210	0.202	0.097
N. margaritacea vs *C. monodonta* F	amino acid p-D	0.435	0.564	0.132	0.280	0.162	0.274	0.301	0.500	0.373	0.422	0.368	0.411	0.500	0.363	0.130
	nucleotide p-D	0.398	0.470	0.216	0.294	0.237	0.328	0.350	0.445	0.347	0.372	0.323	0.377	0.484	0.357	0.081
	d_N	0.404	0.504	0.105	0.224	0.129	0.207	0.248	0.470	0.329	0.354	0.302	0.352	0.525	0.319	0.135
	d_S	1.453	n/c	1.158	1.239	1.319	n/c	n/c	2.811	1.259	1.465	1.017	1.709	n/c	1.492	0.533
	d_N/d_S	0.278	na	0.091	0.181	0.098	na	na	0.167	0.261	0.242	0.297	0.206	na	0.202	0.075
N. margaritacea vs *H. menziesii* M	amino acid p-D	0.518	0.415	0.318	0.427	0.426	0.359	0.468	0.561	0.525	0.526	0.594	0.505	0.696	0.488	0.101
	nucleotide p-D	0.447	0.381	0.321	0.417	0.385	0.365	0.433	0.485	0.412	0.443	0.423	0.453	0.574	0.426	0.062
	d_N	0.495	0.388	0.253	0.421	0.370	0.306	0.429	0.538	0.452	0.509	0.547	0.493	0.831	0.464	0.141
	d_S	1.948	1.451	1.520	2.072	1.849	2.121	n/c	n/c	1.543	1.604	0.926	2.789	n/c	1.782	0.499
	d_N/d_S	0.254	0.267	0.166	0.203	0.200	0.144	na	na	0.293	0.317	0.591	0.177	na	0.261	0.129
N. margaritacea vs *C. monodonta* M	amino acid p-D	0.518	0.648	0.311	0.436	0.428	0.324	0.413	0.575	0.508	0.505	0.587	0.566	0.634	0.496	0.108
	nucleotide p-D	0.420	0.515	0.310	0.369	0.388	0.353	0.404	0.501	0.437	0.413	0.427	0.455	0.515	0.424	0.062
	d_N	0.491	0.725	0.231	0.372	0.387	0.249	0.379	0.600	0.499	0.472	0.538	0.571	0.701	0.478	0.153
	d_S	1.154	1.735	1.570	1.194	1.549	n/c	2.130	n/c	1.784	1.193	0.927	1.379	2.207	1.529	0.410
	d_N/d_S	0.425	0.418	0.147	0.312	0.250	na	0.178	na	0.280	0.396	0.580	0.414	0.318	0.338	0.125
A. trapesialis vs *M. dubia*	amino acid p-D	0.316	0.474	0.063	0.161	0.143	0.242	0.271	0.461	0.262	0.376	0.306	0.386	0.415	0.298	0.125
	nucleotide p-D	0.316	0.368	0.187	0.240	0.224	0.262	0.302	0.385	0.278	0.356	0.313	0.374	0.378	0.306	0.065
	d_N	0.286	0.384	0.047	0.136	0.106	0.170	0.220	0.386	0.205	0.327	0.253	0.345	0.392	0.251	0.114

mtDNAs	Statistic	*atp6*	*atp8*	*cox1*	*cox2*	*cox3*	*cytb*	*nad1*	*nad2*	*nad3*	*nad4*	*nad4L*	*nad5*	*nad6*	average	SD
A. trapesialis vs *M. dubia*	d_S	1.029	0.989	1.382	1.352	1.401	1.208	1.466	1.617	1.085	1.495	1.330	1.733	1.182	1.328	0.224
	d_N/d_S	0.278	0.388	0.034	0.101	0.076	0.141	0.150	0.239	0.189	0.219	0.190	0.199	0.332	0.195	0.100
A. trapesialis vs *H. menziesii* F	amino acid p-D	0.429	0.538	0.100	0.211	0.190	0.353	0.341	0.519	0.310	0.421	0.400	0.426	0.497	0.364	0.133
	nucleotide p-D	0.362	0.442	0.191	0.239	0.239	0.356	0.346	0.420	0.255	0.369	0.319	0.359	0.425	0.332	0.079
	d_N	0.382	0.560	0.072	0.153	0.136	0.286	0.283	0.478	0.218	0.371	0.317	0.358	0.487	0.315	0.146
	d_S	1.012	0.930	1.092	0.941	1.272	2.331	1.694	1.389	0.638	1.243	0.875	1.154	1.405	1.229	0.429
	d_N/d_S	0.377	0.602	0.066	0.163	0.107	0.123	0.167	0.344	0.342	0.298	0.362	0.310	0.347	0.278	0.147
A. trapesialis vs *C. monodonta* F	amino acid p-D	0.410	0.526	0.102	0.218	0.202	0.342	0.365	0.522	0.341	0.395	0.388	0.416	0.513	0.365	0.128
	nucleotide p-D	0.357	0.368	0.203	0.267	0.242	0.353	0.369	0.415	0.310	0.355	0.320	0.366	0.454	0.337	0.069
	d_N	0.340	0.396	0.071	0.175	0.143	0.278	0.324	0.481	0.258	0.334	0.318	0.367	0.504	0.307	0.125
	d_S	1.330	1.034	1.343	1.358	1.223	2.427	1.819	1.234	1.224	1.333	0.866	1.231	2.455	1.452	0.488
	d_N/d_S	0.256	0.383	0.053	0.129	0.117	0.115	0.178	0.390	0.211	0.251	0.367	0.298	0.205	0.227	0.110
A. trapesialis vs *H. menziesii* M	amino acid p-D	0.543	0.590	0.311	0.282	0.416	0.401	0.465	0.646	0.452	0.505	0.565	0.515	0.590	0.483	0.109
	nucleotide p-D	0.434	0.483	0.299	0.307	0.366	0.382	0.417	0.496	0.357	0.410	0.448	0.428	0.510	0.411	0.067
	d_N	0.528	0.719	0.244	0.235	0.360	0.355	0.411	0.690	0.386	0.470	0.539	0.494	0.672	0.469	0.159
	d_S	1.178	1.050	1.128	1.325	1.327	1.741	2.165	1.427	0.960	1.204	1.501	1.379	1.972	1.412	0.357
	d_N/d_S	0.448	0.685	0.216	0.177	0.271	0.204	0.190	0.484	0.402	0.390	0.359	0.358	0.341	0.348	0.143
A. trapesialis vs *C. monodonta* M	amino acid p-D	0.556	0.513	0.297	0.355	0.430	0.426	0.443	0.567	0.435	0.507	0.630	0.568	0.639	0.490	0.103
	nucleotide p-D	0.440	0.450	0.297	0.299	0.367	0.362	0.411	0.474	0.353	0.406	0.459	0.450	0.517	0.407	0.067
	d_N	0.548	0.458	0.223	0.272	0.370	0.322	0.414	0.615	0.415	0.443	0.669	0.599	0.720	0.467	0.154
	d_S	1.154	n/c	1.322	0.847	1.201	1.688	1.748	1.423	0.720	1.299	0.778	1.068	1.699	1.246	0.355
	d_N/d_S	0.475	na	0.169	0.321	0.308	0.191	0.237	0.432	0.576	0.341	0.860	0.561	0.424	0.408	0.195
M. dubia vs *H.menziesii* F	amino acid p-D	0.421	0.544	0.109	0.236	0.181	0.315	0.338	0.513	0.308	0.418	0.400	0.436	0.536	0.366	0.135
	nucleotide p-D	0.365	0.425	0.208	0.282	0.238	0.331	0.323	0.432	0.299	0.383	0.351	0.380	0.433	0.342	0.072
	d_N	0.365	0.543	0.080	0.175	0.128	0.241	0.258	0.470	0.221	0.390	0.372	0.374	0.485	0.316	0.144
	d_S	1.185	0.997	1.341	1.676	1.354	2.180	1.432	1.979	1.630	1.357	0.956	1.486	1.662	1.480	0.351
	d_N/d_S	0.308	0.545	0.060	0.104	0.095	0.111	0.180	0.237	0.136	0.287	0.389	0.252	0.292	0.230	0.137
M. dubia vs *C. monodonta* F	amino acid p-D	0.366	0.472	0.113	0.199	0.193	0.284	0.358	0.527	0.333	0.406	0.378	0.406	0.453	0.345	0.120

mtDNAs	Statistic	*atp6*	*atp8*	*cox1*	*cox2*	*cox3*	*cytb*	*nad1*	*nad2*	*nad3*	*nad4*	*nad4L*	*nad5*	*nad6*	average	SD
M. dubia vs *C. monodonta* F	nucleotide p-D	0.331	0.407	0.207	0.261	0.251	0.312	0.354	0.424	0.319	0.364	0.377	0.372	0.428	0.339	0.068
	d_N	0.308	0.524	0.077	0.157	0.133	0.216	0.282	0.493	0.245	0.358	0.396	0.340	0.448	0.306	0.139
	d_S	1.077	0.784	1.309	1.514	1.645	1.856	2.216	1.340	1.934	1.279	1.203	1.768	2.089	1.540	0.422
	d_N/d_S	0.286	0.668	0.059	0.104	0.081	0.116	0.127	0.368	0.127	0.280	0.329	0.192	0.214	0.227	0.166
M. dubia vs *H.menziesii* M	amino acid p-D	0.518	0.581	0.318	0.416	0.430	0.391	0.458	0.581	0.443	0.496	0.400	0.426	0.497	0.458	0.075
	nucleotide p-D	0.441	0.447	0.316	0.374	0.361	0.365	0.410	0.476	0.374	0.433	0.478	0.359	0.425	0.405	0.050
	d_N	0.512	0.637	0.251	0.363	0.372	0.331	0.417	0.598	0.343	0.480	0.695	0.358	0.487	0.450	0.133
	d_S	1.480	0.989	1.437	1.477	1.093	1.535	1.726	1.714	2.363	1.726	1.026	1.154	1.405	1.471	0.373
	d_N/d_S	0.346	0.644	0.175	0.246	0.340	0.216	0.242	0.349	0.145	0.278	0.677	0.310	0.347	0.332	0.161
M. dubia vs *C. monodonta* M	amino acid p-D	0.529	0.691	0.307	0.429	0.412	0.360	0.434	0.594	0.462	0.508	0.620	0.546	0.586	0.498	0.110
	nucleotide p-D	0.440	0.536	0.314	0.360	0.364	0.324	0.402	0.461	0.401	0.439	0.491	0.461	0.491	0.422	0.068
	d_N	0.525	0.786	0.236	0.361	0.352	0.267	0.388	0.615	0.452	0.516	0.773	0.563	0.654	0.499	0.178
	d_S	1.314	1.919	1.618	1.161	1.328	1.365	1.780	1.156	1.330	1.434	0.854	1.640	1.519	1.417	0.283
	d_N/d_S	0.400	0.410	0.146	0.311	0.265	0.196	0.218	0.532	0.340	0.360	0.905	0.343	0.431	0.373	0.191
H. menziesii F vs *C. monodonta* F	amino acid p-D	0.352	0.519	0.066	0.164	0.116	0.218	0.200	0.413	0.263	0.370	0.316	0.319	0.487	0.293	0.138
	nucleotide p-D	0.312	0.377	0.165	0.215	0.188	0.283	0.274	0.375	0.252	0.333	0.267	0.312	0.424	0.291	0.076
	d_N	0.270	0.503	0.050	0.108	0.078	0.172	0.141	0.335	0.192	0.303	0.220	0.266	0.439	0.237	0.135
	d_S	1.075	0.605	0.921	1.026	1.035	1.687	1.925	1.907	0.891	1.150	0.838	1.123	1.859	1.234	0.449
	d_N/d_S	0.251	0.831	0.054	0.105	0.075	0.102	0.073	0.176	0.215	0.263	0.263	0.237	0.236	0.222	0.200
H. menziesii F vs *C. monodonta* M	amino acid p-D	0.495	0.569	0.293	0.447	0.428	0.333	0.468	0.601	0.460	0.489	0.573	0.538	0.653	0.488	0.102
	nucleotide p-D	0.407	0.429	0.283	0.360	0.342	0.320	0.397	0.488	0.336	0.404	0.411	0.436	0.534	0.396	0.069
	d_N	0.469	0.559	0.218	0.390	0.365	0.254	0.382	0.611	0.378	0.453	0.530	0.526	0.797	0.456	0.154
	d_S	1.068	1.119	1.081	0.900	0.849	1.474	1.724	1.908	0.753	1.178	0.905	1.273	1.601	1.218	0.360
	d_N/d_S	0.439	0.500	0.202	0.433	0.430	0.172	0.222	0.320	0.502	0.385	0.586	0.413	0.498	0.392	0.128
H. menziesii M vs *C. monodonta* F	amino acid p-D	0.507	0.581	0.307	0.438	0.410	0.368	0.478	0.621	0.487	0.509	0.598	0.505	0.641	0.496	0.099
	nucleotide p-D	0.432	0.424	0.305	0.373	0.379	0.375	0.440	0.477	0.353	0.423	0.442	0.409	0.516	0.411	0.055
	d_N	0.480	0.645	0.251	0.389	0.377	0.338	0.434	0.664	0.369	0.486	0.570	0.476	0.710	0.476	0.138
	d_S	1.585	0.520	1.132	1.123	1.496	1.685	n/c	1.149	1.049	1.358	1.094	1.124	1.743	1.255	0.340

**mtDNAs**	**Statistic**	***atp6***	***atp8***	***cox1***	***cox2***	***cox3***	***cytb***	***nad1***	***nad2***	***nad3***	***nad4***	***nad4L***	***nad5***	***nad6***	**average**	**SD**
H. menziesii M vs *C. monodonta* F	d_N/d_S	0.303	1.240	0.222	0.346	0.252	0.201	na	0.578	0.352	0.358	0.521	0.423	0.407	0.434	0.278
H. menziesii M vs *C. monodonta* M	amino acid p-D	0.485	0.605	0.258	0.539	0.434	0.373	0.425	0.582	0.500	0.486	0.451	0.478	0.615	0.479	0.098
	nucleotide p-D	0.396	0.424	0.269	0.421	0.357	0.358	0.399	0.466	0.373	0.391	0.341	0.389	0.481	0.390	0.055
	d_N	0.446	0.551	0.184	0.543	0.355	0.326	0.377	0.594	0.442	0.413	0.317	0.416	0.671	0.433	0.131
	d_S	1.066	0.849	1.207	0.932	1.140	1.421	1.846	1.333	0.861	1.265	1.057	1.249	1.150	1.183	0.265
	d_N/d_S	0.418	0.649	0.152	0.583	0.311	0.229	0.204	0.446	0.513	0.326	0.300	0.333	0.583	0.388	0.158
