# Supplementary material for: Evolution of sex-dependent mtDNA transmission in freshwater mussels (Bivalvia: Unionida)
Source: Sci Rep. 2017 May 8;7:1551. doi: 10.1038/s41598-017-01708-1 (PMC5431520; doi:10.1038/s41598-017-01708-1)
Supplement: Supplementary file 4 — Supplementary Information 4 [file 41598_2017_1708_MOESM4_ESM.pdf]

Evolution of sex-dependent mtDNA transmission in freshwater mussels (Bivalvia: Unionida)

Davide Guerra[1], Federico Plazzi[2], Donald T. Stewart[3], Arthur E. Bogan[4], Walter R. Hoeh[5] & Sophie Breton[1]

[1]Département de Sciences Biologiques, Université de Montréal, Montréal H2V 2S9, Quebéc, Canada. [2]Dipartimento di Scienze Biologiche, Geologiche ed Ambientali (BiGeA), Università di Bologna, Bologna 40126, Italy. [3]Department of Biology, Acadia University, Wolfville B4P 2R6, Nova Scotia, Canada. [4]North Carolina Museum of Natural Sciences, Raleigh, NC 27607, USA. [5]Department of Biological Sciences, Kent State University, Kent, OH 44242, USA.

Supplementary Information 4

Nucleotide sequences of the new ORFs and their translation

Conserved domains (Supplementary Table S6)

`hmmer` functional characterization of F-*orf*, M-*orf*, and new ORFs translated protein sequences

SEQUENCES OF THE NEW ORFs

New additional open reading frames (ORFs) were searched in the unassigned regions (URs) of the seven mitochondrial genomes using EMBOSS `getorf`. Here we display first the nucleotide sequences obtained and then their respective translation. The name of each sequence, written as `name_UR_x_y`, is composed of three identifiers: `name` is the acronym of the mtDNA (Nmar = *Neotrigonia margaritacea*, Atra = *Anodontites trapesialis*, Mdub = *Mutela dubia*, HmenF and HmenM = *Hyridella menziesii* F and M, CmonF and CmonM = *Cumberlandia monodonta* F and M); `UR_x` identifies the unassigned region in the genome (UR1 being between *cox1* and *nad3* in *N. margaritacea*, and between *cox1* and *cox2* in the remaining six mtDNAs; the remaining URs are numbered clockwise starting from this one); and `y` identifies the ORF in the UR. In square brackets are the coordinates of the ORF inside the UR (ORFs can be overlapping among each other). When an ORF was predicted on the reverse strand, this is indicated.

Nucleotide Sequences

Standard and alternative start codons were considered for the search. `getorf` does not include the stop codon in the output nucleotide sequences.

• ***Neotrigonia margaritacea***

```
>Nmar_UR_2_1 [1 - 30]
ATTCATCTGACCAGTTTACCTAGACCCAGA
>Nmar_UR_2_2 [30 - 59]
ATAAAAACAAAACATTATTTATTATTACCC
>Nmar_UR_2_3 [5 - 61]
ATCTGACCAGTTTACCTAGACCCAGATAAAAACAAAACATTATTTATTATTACCCCT
>Nmar_UR_2_4 [54 - 19] (REVERSE SENSE)
ATAATAAATAATGTTTTGTTTTTATCTGGGTCTAGG
>Nmar_UR_2_5 [47 - 3] (REVERSE SENSE)
ATAATGTTTTGTTTTTATCTGGGTCTAGGTAAACTGGTCAGATGA
>Nmar_UR_3_1 [6 - 35]
ATTACTTTAATTCATTTAAACCAACACTTA
>Nmar_UR_3_2 [57 - 92]
ATTTCTTCACTTATCCTTTTACCAAAATTTTCTGAT
>Nmar_UR_3_3 [91 - 120]
ATTAAAAAAAAATATAAAAAACAACACATA
>Nmar_UR_3_4 [35 - 154]
ATAATAACTTACCAACAAGTTAATTTCTTCACTTATCCTTTTACCAAAATTTTCTGATTA
AAAAAAAATATAAAAAACAACACATATAATTTTATACCTCTCCTTATTCACCCAAACCAA
>Nmar_UR_3_5 [167 - 220]
ATTTTTCAACCCTTCATTTCTTACCCTCAATTTTATACTTTCTTCCCCACAGAA
>Nmar_UR_3_6 [120 - 254]
ATAATTTTATACCTCTCCTTATTCACCCAAACCAATAATTTCACCACATTTTTCAACCCT
TCATTTCTTACCCTCAATTTTATACTTTCTTCCCCACAGAATAATAACAATTATCATAAC
TTTCAATTACCCAGA
```

```
>Nmar_UR_3_7 [154 - 357]
ATAATTTCACCACATTTTTCAACCCTTCATTTCTTACCCTCAATTTTATACTTTCTTCCC
CACAGAATAATAACAATTATCATAACTTTCAATTACCCAGATAAAAACCTCCCACTAAGG
GGTTTACATGCAAACCCCAAAATAATCTTTATTTTCGTCATTTTATATACCTTAAAATAT
AATCCTTACTCCAAAACTTTTCTA
>Nmar_UR_3_8 [357 - 407]
ATAATTCTAAAAATCATATTCTTAAACACCCGAAGAAAAAAACTCGAAATT
>Nmar_UR_3_9 [332 - 487]
ATAATCCTTACTCCAAAACTTTTCTATAATTCTAAAAATCATATTCTTAAACACCCGAAG
AAAAAAACTCGAAATTTAACCCCCCCCCCTTTGAACTTTTACGCCATGGGGGCTCGAAAA
ATAGTATCACCACGGAAAACACTATATAGACAATTT
>Nmar_UR_3_10 [484 - 537]
ATTTTAACTAAACATTTCTTTTTCGAGCCCCCATGAATAAATCTATCAGTGCAA
>Nmar_UR_3_11 [516 - 554]
ATGAATAAATCTATCAGTGCAATAGAATCCATGCAACTC
>Nmar_UR_3_12 [524 - 607]
ATCTATCAGTGCAATAGAATCCATGCAACTCTAATACAGAAATACCTTCTAGGTGTGATT
TTTCACTCGGCGAACCGCTGACCA
>Nmar_UR_3_13 [576 - 623]
GTGTGATTTTTCACTCGGCGAACCGCTGACCATAATTCTAGCACTATT
>Nmar_UR_3_14 [565 - 654]
ATACCTTCTAGGTGTGATTTTTCACTCGGCGAACCGCTGACCATAATTCTAGCACTATTT
AGAATGATTTTCCTATTCGGAAACATAATT
>Nmar_UR_3_15 [639 - 713]
ATTCGGAAACATAATTTAGTCTTCTTTTTTCTCTTTTTTTTTCATCTACGACAACAACTC
CTAAAACAACAATGA
>Nmar_UR_3_16 [710 - 766]
ATGATAAAAAAGAACCAAAAAAAAGGGACCCAGCCCAAATCACATTGCCAAGAATTA
>Nmar_UR_3_17 [776 - 814]
ATGGGGTGGATTCTCGTATCAAGTTGCTTCACTCCGAAG
>Nmar_UR_3_18 [753 - 830]
ATTGCCAAGAATTATAAACGTCAATGGGGTGGATTCTCGTATCAAGTTGCTTCACTCCGA
AGTAAAGGGATGCCCAAT
>Nmar_UR_3_19 [748 - 840]
ATCACATTGCCAAGAATTATAAACGTCAATGGGGTGGATTCTCGTATCAAGTTGCTTCAC
TCCGAAGTAAAGGGATGCCCAATTAGGGCTCTA
>Nmar_UR_3_20 [848 - 1006]
ATCTTTACCGTTCGTTCCTCAACCGTCGCTCCCCCGACTCGCTGCTCGTCATTACAGTTC
GAGGCCGGGGGCTCTTCCTTTATTCCTCAACGGTCGCTCGCTCCCCCGGCTGGTTCGTCT
CTCACTTTTGTTCTCCCTTCGCCCACTTGAGTCATCCCT
>Nmar_UR_3_21 [898 - 1050]
ATTACAGTTCGAGGCCGGGGGCTCTTCCTTTATTCCTCAACGGTCGCTCGCTCCCCCGGC
TGGTTCGTCTCTCACTTTTGTTCTCCCTTCGCCCACTTGAGTCATCCCTTAACTCCCCGT
TCCACATGCTCCCTTGAGAATCCACCCCCACAA
>Nmar_UR_3_22 [1031 - 1060]
TTGAGAATCCACCCCCACAATAGAGCTGCA
>Nmar_UR_3_23 [840 - 1088]
ATAGCTCAATCTTTACCGTTCGTTCCTCAACCGTCGCTCCCCCGACTCGCTGCTCGTCAT
TACAGTTCGAGGCCGGGGGCTCTTCCTTTATTCCTCAACGGTCGCTCGCTCCCCCGGCTG
GTTCGTCTCTCACTTTTGTTCTCCCTTCGCCCACTTGAGTCATCCCTTAACTCCCCGTTC
CACATGCTCCCTTGAGAATCCACCCCCACAATAGAGCTGCATAAAAGCAATATACTCTAT
TCAATTCTA
>Nmar_UR_3_24 [1146 - 1181]
ATATTGATTTTATCATTTTCAATTAATATGGTTAAA
```

```
>Nmar_UR_3_25 [1060 - 1206]
ATAAAAGCAATATACTCTATTCAATTCTATAATAAAAAAGAAGATTTCATAATTACTAGT
ACAGGTATCTTTACATGTTTTCATAAATATTGATTTTATCATTTTCAATTAATATGGTTA
AATAAAGAAATAATTTTGATGAAATTA
>Nmar_UR_3_26 [1328 - 1381]
ATGAAATTTAATAACAAAAATTTCCCAATCAAACATCACCTCAACTTTACATAC
>Nmar_UR_3_27 [1203 - 1391]
ATTATAGAATTATTTTGTGTTAATATAAAAGAAAGTCTTTTTACAAAGAAAAGGAAAACT
TTGAGTTTTCCTTTTCCTTTAACTATTAATAATTATTTCTTAAAAAAATATTTAAATTCA
ATTAAATGAAATTTAATAACAAAAATTTCCCAATCAAACATCACCTCAACTTTACATACT
AGAAAACTA
>Nmar_UR_3_28 [1378 - 1416]
ATACTAGAAAACTATAAACTATATTCATTAAATAACATT
>Nmar_UR_3_29 [1371 - 1327] (REVERSE SENSE)
TTGAGGTGATGTTTGATTGGGAAATTTTTGTTATTAAATTTCATT
>Nmar_UR_3_30 [1405 - 1298] (REVERSE SENSE)
ATGAATATAGTTTATAGTTTTCTAGTATGTAAAGTTGAGGTGATGTTTGATTGGGAAATT
TTTGTTATTAAATTTCATTTAATTGAATTTAAATATTTTTTTAAGAAA
>Nmar_UR_3_31 [1323 - 1291] (REVERSE SENSE)
TTGAATTTAAATATTTTTTTAAGAAATAATTAT
>Nmar_UR_3_32 [1199 - 1158] (REVERSE SENSE)
ATCAAAATTATTTCTTTATTTAACCATATTAATTGAAAATGA
>Nmar_UR_3_33 [1288 - 1133] (REVERSE SENSE)
ATAGTTAAAGGAAAAGGAAAACTCAAAGTTTTCCTTTTCTTTGTAAAAAGACTTTCTTTT
ATATTAACACAAAATAATTCTATAATTTCATCAAAATTATTTCTTTATTTAACCATATTA
ATTGAAAATGATAAAATCAATATTTATGAAAACATG
>Nmar_UR_3_34 [1127 - 1089] (REVERSE SENSE)
ATACCTGTACTAGTAATTATGAAATCTTCTTTTTTATTA
>Nmar_UR_3_35 [1167 - 1009] (REVERSE SENSE)
TTGAAAATGATAAAATCAATATTTATGAAAACATGTAAAGATACCTGTACTAGTAATTAT
GAAATCTTCTTTTTTATTATAGAATTGAATAGAGTATATTGCTTTTATGCAGCTCTATTG
TGGGGGTGGATTCTCAAGGGAGCATGTGGAACGGGGAGT
>Nmar_UR_3_36 [1079 - 930] (REVERSE SENSE)
ATAGAGTATATTGCTTTTATGCAGCTCTATTGTGGGGGTGGATTCTCAAGGGAGCATGTG
GAACGGGGAGTTAAGGGATGACTCAAGTGGGCGAAGGGAGAACAAAAGTGAGAGACGAAC
CAGCCGGGGGAGCGAGCGACCGTTGAGGAA
>Nmar_UR_3_37 [1072 - 902] (REVERSE SENSE)
ATATTGCTTTTATGCAGCTCTATTGTGGGGGTGGATTCTCAAGGGAGCATGTGGAACGGG
GAGTTAAGGGATGACTCAAGTGGGCGAAGGGAGAACAAAAGTGAGAGACGAACCAGCCGG
GGGAGCGAGCGACCGTTGAGGAATAAAGGAAGAGCCCCCGGCCTCGAACTG
>Nmar_UR_3_38 [899 - 855] (REVERSE SENSE)
ATGACGAGCAGCGAGTCGGGGGAGCGACGGTTGAGGAACGAACGG
>Nmar_UR_3_39 [1002 - 841] (REVERSE SENSE)
ATGACTCAAGTGGGCGAAGGGAGAACAAAAGTGAGAGACGAACCAGCCGGGGGAGCGAGC
GACCGTTGAGGAATAAAGGAAGAGCCCCCGGCCTCGAACTGTAATGACGAGCAGCGAGTC
GGGGGAGCGACGGTTGAGGAACGAACGGTAAAGATTGAGCTA
>Nmar_UR_3_40 [829 - 767] (REVERSE SENSE)
TTGGGCATCCCTTTACTTCGGAGTGAAGCAACTTGATACGAGAATCCACCCCATTGACGT
TTA
>Nmar_UR_3_41 [848 - 702] (REVERSE SENSE)
TTGAGCTATAGAGCCCTAATTGGGCATCCCTTTACTTCGGAGTGAAGCAACTTGATACGA
GAATCCACCCCATTGACGTTTATAATTCTTGGCAATGTGATTTGGGCTGGGTCCCTTTTT
TTTGGTTCTTTTTTATCATTGTTGTTT
```

```
>Nmar_UR_3_42 [807 - 640] (REVERSE SENSE)
GTGAAGCAACTTGATACGAGAATCCACCCCATTGACGTTTATAATTCTTGGCAATGTGAT
TTGGGCTGGGTCCCTTTTTTTTGGTTCTTTTTTATCATTGTTGTTTTAGGAGTTGTTGTC
GTAGATGAAAAAAAAAGAGAAAAAAGAAGACTAAATTATGTTTCCGAA
>Nmar_UR_3_43 [760 - 626] (REVERSE SENSE)
TTGGCAATGTGATTTGGGCTGGGTCCCTTTTTTTTGGTTCTTTTTTATCATTGTTGTTTT
AGGAGTTGTTGTCGTAGATGAAAAAAAAAGAGAAAAAAGAAGACTAAATTATGTTTCCGA
ATAGGAAAATCATTC
>Nmar_UR_3_44 [622 - 575] (REVERSE SENSE)
ATAGTGCTAGAATTATGGTCAGCGGTTCGCCGAGTGAAAAATCACACC
>Nmar_UR_3_45 [582 - 529] (REVERSE SENSE)
ATCACACCTAGAAGGTATTTCTGTATTAGAGTTGCATGGATTCTATTGCACTGA
>Nmar_UR_3_46 [547 - 494] (REVERSE SENSE)
ATGGATTCTATTGCACTGATAGATTTATTCATGGGGGCTCGAAAAAGAAATGTT
>Nmar_UR_3_47 [525 - 490] (REVERSE SENSE)
ATTTATTCATGGGGGCTCGAAAAAGAAATGTTTAGT
>Nmar_UR_3_48 [653 - 381] (REVERSE SENSE)
ATTATGTTTCCGAATAGGAAAATCATTCTAAATAGTGCTAGAATTATGGTCAGCGGTTCG
CCGAGTGAAAAATCACACCTAGAAGGTATTTCTGTATTAGAGTTGCATGGATTCTATTGC
ACTGATAGATTTATTCATGGGGGCTCGAAAAAGAAATGTTTAGTTAAAATTGTCTATATA
GTGTTTTCCGTGGTGATACTATTTTTCGAGCCCCCATGGCGTAAAAGTTCAAAGGGGGGG
GGGTTAAATTTCGAGTTTTTTTCTTCGGGTGTT
>Nmar_UR_3_49 [406 - 305] (REVERSE SENSE)
ATTTCGAGTTTTTTTCTTCGGGTGTTTAAGAATATGATTTTTAGAATTATAGAAAAGTTT
TGGAGTAAGGATTATATTTTAAGGTATATAAAATGACGAAAA
>Nmar_UR_3_50 [314 - 249] (REVERSE SENSE)
ATGACGAAAATAAAGATTATTTTGGGGTTTGCATGTAAACCCCTTAGTGGGAGGTTTTTA
TCTGGG
>Nmar_UR_3_51 [286 - 233] (REVERSE SENSE)
TTGCATGTAAACCCCTTAGTGGGAGGTTTTTATCTGGGTAATTGAAAGTTATGA
>Nmar_UR_3_52 [255 - 202] (REVERSE SENSE)
ATCTGGGTAATTGAAAGTTATGATAATTGTTATTATTCTGTGGGGAAGAAAGTA
>Nmar_UR_3_53 [245 - 141] (REVERSE SENSE)
TTGAAAGTTATGATAATTGTTATTATTCTGTGGGGAAGAAAGTATAAAATTGAGGGTAAG
AAATGAAGGGTTGAAAAATGTGGTGAAATTATTGGTTTGGGTGAA
>Nmar_UR_3_54 [229 - 95] (REVERSE SENSE)
TTGTTATTATTCTGTGGGGAAGAAAGTATAAAATTGAGGGTAAGAAATGAAGGGTTGAAA
AATGTGGTGAAATTATTGGTTTGGGTGAATAAGGAGAGGTATAAAATTATATGTGTTGTT
TTTTATATTTTTTTT
>Nmar_UR_3_55 [119 - 78] (REVERSE SENSE)
ATGTGTTGTTTTTTATATTTTTTTTTAATCAGAAAATTTTGG
>Nmar_UR_3_56 [183 - 70] (REVERSE SENSE)
ATGAAGGGTTGAAAAATGTGGTGAAATTATTGGTTTGGGTGAATAAGGAGAGGTATAAAA
TTATATGTGTTGTTTTTTATATTTTTTTTTAATCAGAAAATTTTGGTAAAAGGA
>Nmar_UR_3_57 [39 - 10] (REVERSE SENSE)
ATTATAAGTGTTGGTTTAAATGAATTAAAG
>Nmar_UR_3_58 [70 - 2] (REVERSE SENSE)
ATAAGTGAAGAAATTAACTTGTTGGTAAGTTATTATAAGTGTTGGTTTAAATGAATTAAA
GTAATTTTA
>Nmar_UR_14_1 [9 - 47]
ATAATTATCAATTTTATACAAATTCATTGTTTAATATGC
>Nmar_UR_14_2 [5 - 79]
ATAGATAATTATCAATTTTATACAAATTCATTGTTTAATATGCTAATCATTATAATTAAA
CAAATAATAATGATA
```

```
>Nmar_UR_14_3 [78 - 25] (REVERSE SENSE)
ATCATTATTATTTGTTTAATTATAATGATTAGCATATTAAACAATGAATTTGTA
>Nmar_UR_14_4 [35 - 3] (REVERSE SENSE)
ATGAATTTGTATAAAATTGATAATTATCTATAT
>Nmar_UR_14_5 [43 - 2] (REVERSE SENSE)
ATTAAACAATGAATTTGTATAAAATTGATAATTATCTATATT
>Nmar_UR_20_1 [14 - 58]
ATTACATCATCAAAAAATAAAACATTCAGTATAAGAATCATCAAC
>Nmar_UR_20_2 [57 - 1] (REVERSE SENSE)
TTGATGATTCTTATACTGAATGTTTTATTTTTTGATGATGTAATTGATTATAATTGA
>Nmar_UR_21_1 [10 - 39]
ATCAGCCAAATTAAATACAAAAGCTCTGCC
>Nmar_UR_22_1 [6 - 35]
ATATTAATAACCATATATTTAACTTTGACC
>Nmar_UR_22_2 [8 - 40]
ATTAATAACCATATATTTAACTTTGACCTAAAA
>Nmar_UR_23_1 [11 - 52]
ATATTTCAATTAATATTGCAATTCTTTAATATTAATAACCCT
>Nmar_UR_23_2 [39 - 95]
ATATTAATAACCCTTAGACCCCAACAACACTATGTAAACAAACACATCTACAATAAT
>Nmar_UR_23_3 [95 - 45] (REVERSE SENSE)
ATTATTGTAGATGTGTTTGTTTACATAGTGTTGTTGGGGTCTAAGGGTTAT
>Nmar_UR_23_4 [42 - 1] (REVERSE SENSE)
ATATTAAAGAATTGCAATATTAATTGAAATATTAGTTTAAAA
>Nmar_UR_24_1 [24 - 56]
ATTTATAGTAAATTTTCTATCAACCTCAACTTA
>Nmar_UR_24_2 [28 - 63]
ATAGTAAATTTTCTATCAACCTCAACTTATAACTTA
```

• *Anodontites trapesialis*

```
>Atra_UR_2_1 [1 - 33]
ATCTCACCTAATATAGGCCCCACCCAACCTATA
>Atra_UR_2_2 [32 - 3] (REVERSE SENSE)
ATAGGTTGGGTGGGGCCTATATTAGGTGAG
>Atra_UR_3_1 [68 - 24] (REVERSE SENSE)
TTGTTTATTAGGGAAGATTATTTAATTATTATAGATGTTAATTAT
>Atra_UR_13_1 [2 - 31]
ATCTTTGAATCTAACAAAAAAATAAATTTT
>Atra_UR_13_2 [37 - 78]
ATACATTTTCACATACACCTGCCTACACCGTATTTATATTCC
>Atra_UR_13_3 [27 - 104]
ATTTTTAACTATACATTTTCACATACACCTGCCTACACCGTATTTATATTCCTAATACTG
ATGACTCTATTACCCTGG
>Atra_UR_13_4 [115 - 150]
ATATTTTGATTTTTTCAATGTTATAGTATTTATCTT
>Atra_UR_13_5 [41 - 154]
ATTTTCACATACACCTGCCTACACCGTATTTATATTCCTAATACTGATGACTCTATTACC
CTGGTAAGTCTCATATATTTTGATTTTTTCAATGTTATAGTATTTATCTTTAAT
>Atra_UR_13_6 [153 - 200]
ATTAAGTTTTTTTTAAAAAAAAAATAAATAGTCTAATCTTTAACACA
>Atra_UR_13_7 [242 - 189] (REVERSE SENSE)
GTGTTATGTTCTAGAATATATTGTGTGTTTTTATTTAGATTATGTGTTAAAGAT
```

>Atra_UR_13_8 [182 - 153] (REVERSE SENSE)
ATTTATTTTTTTTTAAAAAAAAACTTAAT
>Atra_UR_13_9 [147 - 73] (REVERSE SENSE)
ATAAATACTATAACATTGAAAAAATCAAAATATATGAGACTTACCAGGGTAATAGAGTCA
TCAGTATTAGGAATA
>Atra_UR_13_10 [223 - 62] (REVERSE SENSE)
ATTGTGTGTTTTTATTTAGATTATGTGTTAAAGATTAGACTATTTATTTTTTTTTAAAA
AAAAACTTAATTAAAGATAAATACTATAACATTGAAAAAATCAAAATATATGAGACTTAC
CAGGGTAATAGAGTCATCAGTATTAGGAATATAAATACGGTG
>Atra_UR_13_11 [69 - 34] (REVERSE SENSE)
ATACGGTGTAGGCAGGTGTATGTGAAAATGTATAGT
>Atra_UR_13_12 [50 - 15] (REVERSE SENSE)
ATGTGAAAATGTATAGTTAAAAATTTATTTTTTTGT
>Atra_UR_14_1 [11 - 64]
ATTATCCCAACTAACTATTTACAGTCATTAAATTACTTAACACACAAAAACTTC
>Atra_UR_14_2 [37 - 84]
ATTAAATTACTTAACACACAAAAACTTCTAGTAAATAAAAGTAAAAAT
>Atra_UR_14_3 [96 - 67] (REVERSE SENSE)
GTGTGTTGTTTAATTTTTACTTTTATTTAC
>Atra_UR_14_4 [94 - 50] (REVERSE SENSE)
GTGTTGTTTAATTTTTACTTTTATTTACTAGAAGTTTTTGTGTGT
>Atra_UR_14_5 [53 - 24] (REVERSE SENSE)
GTGTTAAGTAATTTAATGACTGTAAATAGT
>Atra_UR_21_1 [16 - 69]
ATCTTAGGCTTACCCTTTTTAGGCCTTGGAGGTAAAACAACTCTCAAAAGATTA
>Atra_UR_21_2 [41 - 94]
TTGGAGGTAAAACAACTCTCAAAAGATTATAAATCCTCCTGACCACCTTCTCCC
>Atra_UR_21_3 [66 - 98]
ATTATAAATCCTCCTGACCACCTTCTCCCTAAC
>Atra_UR_21_4 [102 - 137]
ATTAAAAAATCCATATTTATACCTATCTCTACTAAA
>Atra_UR_21_5 [137 - 187]
ATAAAATTAAACATAAAAAGAATAATAAAAACAAACCACAACTGGTACCCA
>Atra_UR_21_6 [73 - 213]
ATCCTCCTGACCACCTTCTCCCTAACTAAATTAAAAAATCCATATTTATACCTATCTCTA
CTAAATAAAATTAAACATAAAAAGAATAATAAAAACAAACCACAACTGGTACCCATAACC
CTAATACAAAATTCAATTGAG
>Atra_UR_21_7 [208 - 146] (REVERSE SENSE)
TTGAATTTTGTATTAGGGTTATGGGTACCAGTTGTGGTTTGTTTTTATTATTCTTTTTAT
GTT
>Atra_UR_21_8 [177 - 136] (REVERSE SENSE)
TTGTGGTTTGTTTTTATTATTCTTTTTATGTTTAATTTTATT
>Atra_UR_21_9 [209 - 9] (REVERSE SENSE)
ATTGAATTTTGTATTAGGGTTATGGGTACCAGTTGTGGTTTGTTTTTATTATTCTTTTTA
TGTTTAATTTTATTTAGTAGAGATAGGTATAAATATGGATTTTTTAATTTAGTTAGGGAG
AAGGTGGTCAGGAGGATTTATAATCTTTTGAGAGTTGTTTTACCTCCAAGGCCTAAAAAG
GGTAAGCCTAAGATTAAAAAG
>Atra_UR_21_10 [70 - 2] (REVERSE SENSE)
ATAATCTTTTGAGAGTTGTTTTACCTCCAAGGCCTAAAAAGGGTAAGCCTAAGATTAAAA
AGTAAGGTA
>Atra_UR_22_1 [7 - 78]
ATATCCATACCATCACTAATATCCAAAAATCGAATTACTACAAAAACACAACCCAAACAA
CAAACGAATACG

>Atra_UR_22_2 [9 - 86]
ATCCATACCATCACTAATATCCAAAAATCGAATTACTACAAAAACACAACCCAAACAACA
AACGAATACGTAAACTTA
>Atra_UR_22_3 [111 - 197]
ATTACAAAATTATACCTACCTACCTCACAAATAAACTCAAACACACAAAGAATAAACAAA
CATACCAAGATAAAACACCAACAATTA
>Atra_UR_22_4 [35 - 220]
ATCGAATTACTACAAAAACACAACCCAAACAACAAACGAATACGTAAACTTATAATAAAT
CTTATCCCTTACCTAGATTACAAAATTATACCTACCTACCTCACAAATAAACTCAAACAC
ACAAAGAATAAACAAACATACCAAGATAAAACACCAACAATTATAACACAAAACCTAAAT
AAAAAT
>Atra_UR_22_5 [213 - 275]
ATAAAAATTAAAAACACTTTGGTTAACAATACCAACCCAACAATTTTAAGCTATATATTT
ACT
>Atra_UR_22_6 [292 - 357]
ATGCTTATACTACCACCTAGAAATCAATTTACCACAAAAATACAACCCAAACAACAAACG
AACACA
>Atra_UR_22_7 [318 - 365]
ATTTACCACAAAAATACAACCCAAACAACAAACGAACACATAAACCTA
>Atra_UR_22_8 [269 - 382]
ATTTACTTAATCTTCCTCCATAAATGCTTATACTACCACCTAGAAATCAATTTACCACAA
AAATACAACCCAAACAACAAACGAACACATAAACCTATAATAAACCTTACTTCT
>Atra_UR_22_9 [398 - 436]
TTGATGGTAAAACAACTCTTCAAAGACCATATCCTGTCT
>Atra_UR_22_10 [385 - 453]
ATTTTAACACCTTTTGATGGTAAAACAACTCTTCAAAGACCATATCCTGTCTTAACTCTT
TCCTTCCTA
>Atra_UR_22_11 [429 - 391] (REVERSE SENSE)
ATATGGTCTTTGAAGAGTTGTTTTACCATCAAAAGGTGT
>Atra_UR_22_12 [427 - 323] (REVERSE SENSE)
ATGGTCTTTGAAGAGTTGTTTTACCATCAAAAGGTGTTAAAATTAAGAAGTAAGGTTTAT
TATAGGTTTATGTGTTCGTTTGTTGTTTGGGTTGTATTTTTGTGG
>Atra_UR_22_13 [369 - 304] (REVERSE SENSE)
ATTATAGGTTTATGTGTTCGTTTGTTGTTTGGGTTGTATTTTTGTGGTAAATTGATTTCT
AGGTGG
>Atra_UR_22_14 [356 - 261] (REVERSE SENSE)
GTGTTCGTTTGTTGTTTGGGTTGTATTTTTGTGGTAAATTGATTTCTAGGTGGTAGTATA
AGCATTTATGGAGGAAGATTAAGTAAATATATAGCT
>Atra_UR_22_15 [270 - 238] (REVERSE SENSE)
ATATATAGCTTAAAATTGTTGGGTTGGTATTGT
>Atra_UR_22_16 [256 - 212] (REVERSE SENSE)
ATTGTTGGGTTGGTATTGTTAACCAAAGTGTTTTTAATTTTTATT
>Atra_UR_22_17 [242 - 198] (REVERSE SENSE)
ATTGTTAACCAAAGTGTTTTTAATTTTTATTTAGGTTTTGTGTTA
>Atra_UR_22_18 [198 - 133] (REVERSE SENSE)
ATAATTGTTGGTGTTTTATCTTGGTATGTTTGTTTATTCTTTGTGTGTTTGAGTTTATTT
GTGAGG
>Atra_UR_22_19 [194 - 129] (REVERSE SENSE)
TTGTTGGTGTTTTATCTTGGTATGTTTGTTTATTCTTTGTGTGTTTGAGTTTATTTGTGA
GGTAGG
>Atra_UR_22_20 [205 - 110] (REVERSE SENSE)
TTGTGTTATAATTGTTGGTGTTTTATCTTGGTATGTTTGTTTATTCTTTGTGTGTTTGAG
TTTATTTGTGAGGTAGGTAGGTATAATTTTGTAATC

>Atra_UR_22_21 [94 - 47] (REVERSE SENSE)
ATTTATTATAAGTTTACGTATTCGTTTGTTGTTTGGGTTGTGTTTTTG
>Atra_UR_22_22 [99 - 25] (REVERSE SENSE)
ATAAGATTTATTATAAGTTTACGTATTCGTTTGTTGTTTGGGTTGTGTTTTTGTAGTAAT
TCGATTTTTGGATAT
>Atra_UR_22_23 [62 - 3] (REVERSE SENSE)
TTGGGTTGTGTTTTTGTAGTAATTCGATTTTTGGATATTAGTGATGGTATGGATATTTGT
>Atra_UR_23_1 [24 - 56]
ATGAACACAACAAACTCAAATTATACACCTAAC
>Atra_UR_23_2 [22 - 87]
ATATGAACACAACAAACTCAAATTATACACCTAACTAGTCTTCCTTCACAGTTATCCACA
CCACTT
>Atra_UR_23_3 [51 - 16] (REVERSE SENSE)
GTGTATAATTTGAGTTTGTTGTGTTCATATTGCTTG
>Atra_UR_23_4 [80 - 3] (REVERSE SENSE)
GTGGATAACTGTGAAGGAAGACTAGTTAGGTGTATAATTTGAGTTTGTTGTGTTCATATT
GCTTGTAAAAGCGTCTTC
>Atra_UR_23_5 [31 - 2] (REVERSE SENSE)
GTGTTCATATTGCTTGTAAAAGCGTCTTCT

• ***Mutela dubia***

>Mdub_UR_4_1 [16 - 45]
ATTTATTTTTGCTTAGTATTACTTATAAAC
>Mdub_UR_4_2 [9 - 116]
ATTTCTTATTTATTTTTGCTTAGTATTACTTATAAACTAATTTCTCAAAAACAACAAATT
TTTAAAAATTTAATAATAAAATTTTTAACCAATTTGTTTAATACAATA
>Mdub_UR_4_3 [77 - 48] (REVERSE SENSE)
ATTTTTAAAAATTTGTTGTTTTTGAGAAAT
>Mdub_UR_4_4 [115 - 41] (REVERSE SENSE)
ATTGTATTAAACAAATTGGTTAAAAATTTTATTATTAAATTTTTAAAAATTTGTTGTTTT
TGAGAAATTAGTTTA
>Mdub_UR_15_1 [26 - 64]
ATTATAATCTCTACTATAAAGCTACTATTATCAATCATT
>Mdub_UR_15_2 [75 - 110]
ATTATAAACACAAATGCTCTATATCAGTCTACCAAA
>Mdub_UR_15_3 [95 - 130]
ATATCAGTCTACCAAATAATTTCAATGTTACGCCTC
>Mdub_UR_15_4 [134 - 163]
ATTATACTAACACTATTAGCCTTATGCCCA
>Mdub_UR_15_5 [88 - 231]
ATGCTCTATATCAGTCTACCAAATAATTTCAATGTTACGCCTCTAAATTATACTAACACT
ATTAGCCTTATGCCCATAATCAACTACATGCAACAGAAGCTAGCGGCAGAGGACTGGATA
AAGAGGGGGCTCTTGTTTAATTTT
>Mdub_UR_15_6 [227 - 274]
ATTTTTAATTTTTGGGTTTTGGTTCATATGAGCGCGCAAGCGGTGCAA
>Mdub_UR_15_7 [238 - 318]
TTGGGTTTTGGTTCATATGAGCGCGCAAGCGGTGCAATAGAGACATCAATCATTATAGTG
AATGATTGACGGGATGTGTAC
>Mdub_UR_15_8 [358 - 444]
GTGGTGGGGAGTCCGCAAGCGGAGTTATTGGGAGTCACTGGTATGGTTGATTGGTGGGTG
AGGCGGCTCCCAGGGGGTATACTTTCC

>Mdub_UR_15_9 [299 - 466]
ATGATTGACGGGATGTGTACTAACCCACTTGACTTTAATTTGGGGATGGAGGCGAGGGAG
TGGTGGGGAGTCCGCAAGCGGAGTTATTGGGAGTCACTGGTATGGTTGATTGGTGGGTGA
GGCGGCTCCCAGGGGGTATACTTTCCTAAACCAAATTTTAGCTTCAAC
>Mdub_UR_15_10 [234 - 485]
ATTTTTGGGTTTTGGTTCATATGAGCGCGCAAGCGGTGCAATAGAGACATCAATCATTAT
AGTGAATGATTGACGGGATGTGTACTAACCCACTTGACTTTAATTTGGGGATGGAGGCGA
GGGAGTGGTGGGGAGTCCGCAAGCGGAGTTATTGGGAGTCACTGGTATGGTTGATTGGTG
GGTGAGGCGGCTCCCAGGGGGTATACTTTCCTAAACCAAATTTTAGCTTCAACTAGAGTG
CAGCAGGTAACA
>Mdub_UR_15_11 [539 - 625]
GTGAATTGGGGGTGGGAGACGGTTGGTTGAACTGTCCAAGGGCACCTCTACAGAACCAAG
GGTACTTATGAGAACCTGCCTTTGAAT
>Mdub_UR_15_12 [514 - 669]
ATACTCTCCCTACCTCCCATCATGTGTGAATTGGGGGTGGGAGACGGTTGGTTGAACTGT
CCAAGGGCACCTCTACAGAACCAAGGGTACTTATGAGAACCTGCCTTTGAATTAGAGCTT
CCGAGCACAGCACAATACGGTGGACCGGAGCCATCG
>Mdub_UR_15_13 [716 - 745]
ATGGTCCTATGGAGTGATTGCAGAGCATTG
>Mdub_UR_15_14 [752 - 790]
ATTATGAGAATGCATCAGGTGTGATTGACTGTGGAACTT
>Mdub_UR_15_15 [537 - 797]
GTGTGAATTGGGGGTGGGAGACGGTTGGTTGAACTGTCCAAGGGCACCTCTACAGAACCA
AGGGTACTTATGAGAACCTGCCTTTGAATTAGAGCTTCCGAGCACAGCACAATACGGTGG
ACCGGAGCCATCGTAGACCTTAACTTTAGCTACCTTGGCTCTGGGCTCTACGGCCTCCTA
TGGTCCTATGGAGTGATTGCAGAGCATTGTAAAATATTATGAGAATGCATCAGGTGTGAT
TGACTGTGGAACTTTAAACTT
>Mdub_UR_15_16 [807 - 839]
ATTTACAGGATGGATGATTCCTTTTTTTTCATT
>Mdub_UR_15_17 [691 - 897]
TTGGCTCTGGGCTCTACGGCCTCCTATGGTCCTATGGAGTGATTGCAGAGCATTGTAAAA
TATTATGAGAATGCATCAGGTGTGATTGACTGTGGAACTTTAAACTTTAAAACGAGATTT
ACAGGATGGATGATTCCTTTTTTTTCATTTAATTATAAATTTTTTAAATTAATAACCTTT
TGTCTACACCTACACATACAAATTTTT
>Mdub_UR_15_18 [858 - 905]
ATTAATAACCTTTTGTCTACACCTACACATACAAATTTTTTAAAAAAT
>Mdub_UR_15_19 [893 - 846] (REVERSE SENSE)
ATTTGTATGTGTAGGTGTAGACAAAAGGTTATTAATTTAAAAAATTTA
>Mdub_UR_15_20 [846 - 793] (REVERSE SENSE)
ATAATTAAATGAAAAAAAAGGAATCATCCATCCTGTAAATCTCGTTTTAAAGTT
>Mdub_UR_15_21 [824 - 756] (REVERSE SENSE)
ATCATCCATCCTGTAAATCTCGTTTTAAAGTTTAAAGTTCCACAGTCAATCACACCTGAT
GCATTCTCA
>Mdub_UR_15_22 [766 - 725] (REVERSE SENSE)
ATGCATTCTCATAATATTTTACAATGCTCTGCAATCACTCCA
>Mdub_UR_15_23 [762 - 685] (REVERSE SENSE)
ATTCTCATAATATTTTACAATGCTCTGCAATCACTCCATAGGACCATAGGAGGCCGTAGA
GCCCAGAGCCAAGGTAGC
>Mdub_UR_15_24 [641 - 510] (REVERSE SENSE)
GTGCTCGGAAGCTCTAATTCAAAGGCAGGTTCTCATAAGTACCCTTGGTTCTGTAGAGGT
GCCCTTGGACAGTTCAACCAACCGTCTCCCACCCCCAATTCACACATGATGGGAGGTAGG
GAGAGTATCGGT

```
>Mdub_UR_15_25 [465 - 385] (REVERSE SENSE)
TTGAAGCTAAAATTTGGTTTAGGAAAGTATACCCCCTGGGAGCCGCCTCACCCACCAATC
AACCATACCAGTGACTCCCAA
>Mdub_UR_15_26 [437 - 336] (REVERSE SENSE)
ATACCCCCTGGGAGCCGCCTCACCCACCAATCAACCATACCAGTGACTCCCAATAACTCC
GCTTGCGGACTCCCCACCACTCCCTCGCCTCCATCCCCAAAT
>Mdub_UR_15_27 [667 - 293] (REVERSE SENSE)
ATGGCTCCGGTCCACCGTATTGTGCTGTGCTCGGAAGCTCTAATTCAAAGGCAGGTTCTC
ATAAGTACCCTTGGTTCTGTAGAGGTGCCCTTGGACAGTTCAACCAACCGTCTCCCACCC
CCAATTCACACATGATGGGAGGTAGGGAGAGTATCGGTTAACAGGGGGTTCGTGTTAAGT
TATGTTACCTGCTGCACTCTAGTTGAAGCTAAAATTTGGTTTAGGAAAGTATACCCCCTG
GGAGCCGCCTCACCCACCAATCAACCATACCAGTGACTCCCAATAACTCCGCTTGCGGAC
TCCCCACCACTCCCTCGCCTCCATCCCCAAATTAAAGTCAAGTGGGTTAGTACACATCCC
GTCAATCATTCACTA
>Mdub_UR_15_28 [293 - 234] (REVERSE SENSE)
ATAATGATTGATGTCTCTATTGCACCGCTTGCGCGCTCATATGAACCAAAACCCAAAAAT
>Mdub_UR_15_29 [286 - 227] (REVERSE SENSE)
TTGATGTCTCTATTGCACCGCTTGCGCGCTCATATGAACCAAAACCCAAAAATTAAAAAT
>Mdub_UR_15_30 [375 - 190] (REVERSE SENSE)
TTGCGGACTCCCCACCACTCCCTCGCCTCCATCCCCAAATTAAAGTCAAGTGGGTTAGTA
CACATCCCGTCAATCATTCACTATAATGATTGATGTCTCTATTGCACCGCTTGCGCGCTC
ATATGAACCAAAACCCAAAAATTAAAAATTAAACAAGAGCCCCCTCTTTATCCAGTCCTC
TGCCGC
>Mdub_UR_15_31 [206 - 174] (REVERSE SENSE)
ATCCAGTCCTCTGCCGCTAGCTTCTGTTGCATG
>Mdub_UR_15_32 [170 - 138] (REVERSE SENSE)
TTGATTATGGGCATAAGGCTAATAGTGTTAGTA
>Mdub_UR_15_33 [180 - 133] (REVERSE SENSE)
TTGCATGTAGTTGATTATGGGCATAAGGCTAATAGTGTTAGTATAATT
>Mdub_UR_15_34 [109 - 74] (REVERSE SENSE)
TTGGTAGACTGATATAGAGCATTTGTGTTTATAATC
>Mdub_UR_15_35 [89 - 42] (REVERSE SENSE)
ATTTGTGTTTATAATCTAATAATTAAATGATTGATAATAGTAGCTTTA
>Mdub_UR_15_36 [63 - 22] (REVERSE SENSE)
ATGATTGATAATAGTAGCTTTATAGTAGAGATTATAATCTTA
>Mdub_UR_16_1 [45 - 74]
ATTAATATTCCTAACCATCCCAAGTCTACT
>Mdub_UR_16_2 [40 - 90]
ATCCTATTAATATTCCTAACCATCCCAAGTCTACTTAACATATATTTAATT
>Mdub_UR_16_3 [81 - 110]
ATATTTAATTTAAAAAAATACTATATTTAT
>Mdub_UR_16_4 [98 - 136]
ATACTATATTTATTAATAAATAAAAACTTATTAATAACA
>Mdub_UR_16_5 [137 - 93] (REVERSE SENSE)
TTGTTATTAATAAGTTTTTATTTATTAATAAATATAGTATTTTTT
>Mdub_UR_16_6 [132 - 88] (REVERSE SENSE)
ATTAATAAGTTTTTATTTATTAATAAATATAGTATTTTTTTAAAT
>Mdub_UR_16_7 [89 - 12] (REVERSE SENSE)
ATTAAATATATGTTAAGTAGACTTGGGATGGTTAGGAATATTAATAGGATTAGTTTTTTA
TTTGGGGAAAAGAAGTTA
>Mdub_UR_16_8 [67 - 8] (REVERSE SENSE)
TTGGGATGGTTAGGAATATTAATAGGATTAGTTTTTTATTTGGGGAAAAGAAGTTATAGT
>Mdub_UR_16_9 [30 - 1] (REVERSE SENSE)
ATTTGGGGAAAAGAAGTTATAGTTAGTAAA
```

>Mdub_UR_23_1 [35 - 3] (REVERSE SENSE)
TTGGTAATTATTTATTTAGTTTTTGATTATA

• *Hyridella menziesii* F

>HmenF_UR_1_1 [34 - 69]
ATATTAACTCCACACTTCCCATACCACAATTACCAC
>HmenF_UR_1_2 [36 - 71]
ATTAACTCCACACTTCCCATACCACAATTACCACAC
>HmenF_UR_1_3 [69 - 40] (REVERSE SENSE)
GTGGTAATTGTGGTATGGGAAGTGTGGAGT
>HmenF_UR_1_4 [62 - 3] (REVERSE SENSE)
TTGTGGTATGGGAAGTGTGGAGTTAATATGTGCTTGTGGGTATTTAGGTTGGTGGTTTTG
>HmenF_UR_1_5 [55 - 2] (REVERSE SENSE)
ATGGGAAGTGTGGAGTTAATATGTGCTTGTGGGTATTTAGGTTGGTGGTTTTGA
>HmenF_UR_2_1 [36 - 1] (REVERSE SENSE)
TTGAGTTTAGTGGGTGATAATAATGGAGGACAGTAT
>HmenF_UR_5_1 [11 - 43]
ATAGCTACACAACCAACCATACCCTCACATCAC
>HmenF_UR_5_2 [43 - 2] (REVERSE SENSE)
GTGATGTGAGGGTATGGTTGGTTGTGTAGCTATTGTTGAGAG
>HmenF_UR_5_3 [30 - 1] (REVERSE SENSE)
ATGGTTGGTTGTGTAGCTATTGTTGAGAGT
>HmenF_UR_8_1 [3 - 32]
ATTCCTTCAAATACACCCCAAAGTACACAC
>HmenF_UR_8_2 [32 - 3] (REVERSE SENSE)
GTGTGTACTTTGGGGTGTATTTGAAGGAAT
>HmenF_UR_10_1 [41 - 3] (REVERSE SENSE)
TTGATATTGTGTTGTAGAGGGTTATTTGTTTATTTTATT
>HmenF_UR_13_1 [3 - 44]
GTGGTCGTAGCCCCACGAACCACAGCCATTAAACAAAACTTT
>HmenF_UR_13_2 [38 - 3] (REVERSE SENSE)
TTGTTTAATGGCTGTGGTTCGTGGGGCTACGACCAC
>HmenF_UR_13_3 [31 - 2] (REVERSE SENSE)
ATGGCTGTGGTTCGTGGGGCTACGACCACA
>HmenF_UR_16_1 [39 - 1] (REVERSE SENSE)
ATCTTTAGATTTTTTTGTGGATTAGTTAAGTGGTTGTTG
>HmenF_UR_17_1 [35 - 64]
ATATCTTTGCTCAAACCAACCTTTACAGGC
>HmenF_UR_17_2 [69 - 122]
ATCAAGCCAAACCAAGCTACCATATCCTCCAACCCTGTATTTACAACTTATAAA
>HmenF_UR_17_3 [37 - 129]
ATCTTTGCTCAAACCAACCTTTACAGGCTAAAATCAAGCCAAACCAAGCTACCATATCCT
CCAACCCTGTATTTACAACTTATAAATAAATCT
>HmenF_UR_17_4 [119 - 60] (REVERSE SENSE)
ATAAGTTGTAAATACAGGGTTGGAGGATATGGTAGCTTGGTTTGGCTTGATTTTAGCCTG
>HmenF_UR_17_5 [100 - 26] (REVERSE SENSE)
TTGGAGGATATGGTAGCTTGGTTTGGCTTGATTTTAGCCTGTAAAGGTTGGTTTGAGCAA
AGATATGCTAGGCGT
>HmenF_UR_17_6 [48 - 10] (REVERSE SENSE)
TTGAGCAAAGATATGCTAGGCGTTAGGGGCTTGGTTTGG
>HmenF_UR_21_1 [39 - 4] (REVERSE SENSE)
TTGATACTTGGATGTTTAGTGTTAGATGATGTGGGT

```
>HmenF_UR_24_1 [6 - 62]
ATACAATTAACATACACTAATCCTCCACTACCGCCAACCCAACCCACATCAACCACC
>HmenF_UR_24_2 [25 - 75]
ATCCTCCACTACCGCCAACCCAACCCACATCAACCACCTAACAATCCCGTC
>HmenF_UR_24_3 [53 - 88]
ATCAACCACCTAACAATCCCGTCTAACCTCACCCAC
>HmenF_UR_24_4 [68 - 36] (REVERSE SENSE)
TTGTTAGGTGGTTGATGTGGGTTGGGTTGGCGG
>HmenF_UR_24_5 [84 - 25] (REVERSE SENSE)
GTGAGGTTAGACGGGATTGTTAGGTGGTTGATGTGGGTTGGGTTGGCGGTAGTGGAGGAT
>HmenF_UR_24_6 [61 - 2] (REVERSE SENSE)
GTGGTTGATGTGGGTTGGGTTGGCGGTAGTGGAGGATTAGTGTATGTTAATTGTATATCT
>HmenF_UR_25_1 [74 - 103]
ATTACCCTTCTTAACCACTATTACAAACCT
>HmenF_UR_25_2 [94 - 53] (REVERSE SENSE)
ATAGTGGTTAAGAAGGGTAATGGTGTGGTGCCAGGTGCCCTT
>HmenF_UR_25_3 [75 - 34] (REVERSE SENSE)
ATGGTGTGGTGCCAGGTGCCCTTTAGAGTGTTGGGTTGTAGG
>HmenF_UR_26_1 [1 - 30]
ATAACACTCAAAAGAAACTCTAACCTCACC
>HmenF_UR_26_2 [36 - 1] (REVERSE SENSE)
TTGTTAGGTGAGGTTAGAGTTTCTTTTGAGTGTTAT
```

• *Cumberlandia monodonta* F

```
>CmonF_UR_1_1 [31 - 2] (REVERSE SENSE)
ATGTTTGTGAGGGTTTGTTACGTGTTAAGG
>CmonF_UR_3_1 [31 - 2] (REVERSE SENSE)
TTGTTTTTATTCGTCGTAGACGAGTGGAAA
>CmonF_UR_4_1 [32 - 91]
ATTTACCAGGTAAAACCTTATACAAAAAAATCAATTTACCACTTAAACAAAATATTATGA
>CmonF_UR_4_2 [61 - 138]
ATCAATTTACCACTTAAACAAAATATTATGATAATTAACTTTTTTCAAGCAGCTAGTCAG
CTAGCAGCAAACAGGAAA
>CmonF_UR_4_3 [154 - 183]
ATGAACCAACTAATGAACAGATCAATAAAC
>CmonF_UR_4_4 [138 - 215]
ATAAACAAACCGGTAAATGAACCAACTAATGAACAGATCAATAAACTAACCAATAACAAG
AATACCCATCACCTAAAC
>CmonF_UR_4_5 [162 - 124] (REVERSE SENSE)
TTGGTTCATTTACCGGTTTGTTTATTTCCTGTTTGCTGC
>CmonF_UR_4_6 [196 - 116] (REVERSE SENSE)
TTGTTATTGGTTAGTTTATTGATCTGTTCATTAGTTGGTTCATTTACCGGTTTGTTTATT
TCCTGTTTGCTGCTAGCTGAC
>CmonF_UR_4_7 [108 - 70] (REVERSE SENSE)
TTGAAAAAAGTTAATTATCATAATATTTTGTTTAAGTGG
>CmonF_UR_4_8 [179 - 3] (REVERSE SENSE)
ATTGATCTGTTCATTAGTTGGTTCATTTACCGGTTTGTTTATTTCCTGTTTGCTGCTAGC
TGACTAGCTGCTTGAAAAAAGTTAATTATCATAATATTTTGTTTAAGTGGTAAATTGATT
TTTTTGTATAAGGTTTTACCTGGTAAATTGGTTCTTCTTTGAAGAGGTTTTGGGTGC
>CmonF_UR_4_9 [73 - 2] (REVERSE SENSE)
GTGGTAAATTGATTTTTTTGTATAAGGTTTTACCTGGTAAATTGGTTCTTCTTTGAAGAG
GTTTTGGGTGCG
```

```
>CmonF_UR_4_10 [33 - 1] (REVERSE SENSE)
ATTGGTTCTTCTTTGAAGAGGTTTTGGGTGCGG
>CmonF_UR_7_1 [6 - 44]
ATTCACTCACCTACACAAGGGAAGGCAAATCAATCACCA
>CmonF_UR_7_2 [1 - 45]
ATACAATTCACTCACCTACACAAGGGAAGGCAAATCAATCACCAG
>CmonF_UR_7_3 [38 - 3] (REVERSE SENSE)
TTGATTTGCCTTCCCTTGTGTAGGTGAGTGAATTGT
>CmonF_UR_8_1 [7 - 36]
ATTCAACCCACCAAAATCCCTCTCCACCCT
>CmonF_UR_8_2 [33 - 1] (REVERSE SENSE)
GTGGAGAGGGATTTTGGTGGGTTGAATTTCGTT
>CmonF_UR_10_1 [24 - 62]
ATTCAAGACAACTTAAATATCTACAAACCCCACATGACT
>CmonF_UR_10_2 [43 - 2] (REVERSE SENSE)
ATATTTAAGTTGTCTTGAATAAGAAGGTTTTGTTTGTTAAAA
>CmonF_UR_17_1 [9 - 71]
ATAATCACCCATCAAGGAAATACCCATCAAGACTTCCCAAAAACATCAACATGCCGACAA
AAT
>CmonF_UR_17_2 [19 - 75]
ATCAAGGAAATACCCATCAAGACTTCCCAAAAACATCAACATGCCGACAAAATTAAT
>CmonF_UR_17_3 [71 - 3] (REVERSE SENSE)
ATTTTGTCGGCATGTTGATGTTTTTGGGAAGTCTTGATGGGTATTTCCTTGATGGGTGAT
TATTTCTGG
>CmonF_UR_17_4 [75 - 1] (REVERSE SENSE)
ATTAATTTTGTCGGCATGTTGATGTTTTTGGGAAGTCTTGATGGGTATTTCCTTGATGGG
TGATTATTTCTGGTT
>CmonF_UR_18_1 [2 - 37]
ATACTCCATCTAAACCCCCACCAACACGCATTAACA
>CmonF_UR_18_2 [38 - 3] (REVERSE SENSE)
ATGTTAATGCGTGTTGGTGGGGGTTTAGATGGAGTA
>CmonF_UR_19_1 [1 - 30]
ATAGCTTTCTTTAATCATCTGTGCAAAATA
>CmonF_UR_19_2 [14 - 52]
ATCATCTGTGCAAAATATAAATACAGAATTATCTGCCTT
>CmonF_UR_19_3 [21 - 53]
GTGCAAAATATAAATACAGAATTATCTGCCTTG
>CmonF_UR_19_4 [35 - 3] (REVERSE SENSE)
ATTTATATTTTGCACAGATGATTAAAGAAAGCT
>CmonF_UR_19_5 [45 - 1] (REVERSE SENSE)
ATAATTCTGTATTTATATTTTGCACAGATGATTAAAGAAAGCTAT
>CmonF_UR_20_1 [5 - 43]
ATAACGTCAACGCTTATTGACACGAGTTTTCAACACAAT
>CmonF_UR_20_2 [21 - 56]
TTGACACGAGTTTTCAACACAATTAATATCTATCAA
>CmonF_UR_20_3 [62 - 21] (REVERSE SENSE)
ATTTTATTGATAGATATTAATTGTGTTGAAAACTCGTGTCAA
>CmonF_UR_20_4 [49 - 2] (REVERSE SENSE)
ATATTAATTGTGTTGAAAACTCGTGTCAATAAGCGTTGACGTTATTAC
>CmonF_UR_20_5 [42 - 1] (REVERSE SENSE)
TTGTGTTGAAAACTCGTGTCAATAAGCGTTGACGTTATTACA
>CmonF_UR_25_1 [2 - 46]
GTGCAACAAGCCTTCACGGTCATCACCCTATTCCTGCTATCACCT
```

```
>CmonF_UR_25_2 [44 - 3] (REVERSE SENSE)
GTGATAGCAGGAATAGGGTGATGACCGTGAAGGCTTGTTGCA
>CmonF_UR_26_1 [1 - 33]
ATACTCACCCAAGTAAATTTCTCTTATACGAAC
>CmonF_UR_28_1 [51 - 13] (REVERSE SENSE)
GTGTGGGAAGAGGTTGGGTTGTCTAGGTTGTGGGGGGCG
>CmonF_UR_28_2 [38 - 3] (REVERSE SENSE)
TTGGGTTGTCTAGGTTGTGGGGGGCGTAGTTATACT
>CmonF_UR_29_1 [46 - 17] (REVERSE SENSE)
ATGGACTGAATGTGGTGGGGTTGTAGTGGT
```

• ***Hyridella menziesii* M**

```
>HmenM_UR_2_1 [30 - 59]
ATAACAACTCCACCTTTAATCTGGGCCTTA
>HmenM_UR_2_2 [60 - 31] (REVERSE SENSE)
ATAAGGCCCAGATTAAAGGTGGAGTTGTTA
>HmenM_UR_2_3 [49 - 2] (REVERSE SENSE)
ATTAAAGGTGGAGTTGTTATAGGAGGACGTGGAGGATTGGCTTGTGTA
>HmenM_UR_3_1 [8 - 64]
ATTAAACGCACAACAACTACAACCACAAACAACCGACCAGATTACTCAAACAAACAA
>HmenM_UR_3_2 [94 - 177]
ATTACTTGACCATACCCTTCCCCCCCTTACACCCCCCCATCCCTACCTATCTTCTTCTTT
TTGTTACTTTCATCGGAAGGGAAG
>HmenM_UR_3_3 [310 - 339]
ATCTCTACTTCACTAGTAATACAGCTTGAA
>HmenM_UR_3_4 [386 - 460]
ATACTTACTTCTACACACCTTACCTACACACCACCCAAGTGGGGCCCCCCCCTCCCCCCC
TGCACACCTCTACGC
>HmenM_UR_3_5 [424 - 597]
GTGGGGCCCCCCCCTCCCCCCCTGCACACCTCTACGCTAACGAGAAGTGCGCAAGGGAAA
AGTCTACGCCGACTAAAGACTCATCCGCTCGGGGAGCCCGCGGGAATCCCGCCTCTACAG
GGCAGCCCACGACCGCCAGCCCATTGTCTGCCAGCTCCACCACCCTATTTTATA
>HmenM_UR_3_6 [506 - 610]
ATCCGCTCGGGGAGCCCGCGGGAATCCCGCCTCTACAGGGCAGCCCACGACCGCCAGCCC
ATTGTCTGCCAGCTCCACCACCCTATTTTATATAGTAGTCCTATT
>HmenM_UR_3_7 [75 - 638]
ATTAATATAAACACCAACAATTACTTGACCATACCCTTCCCCCCCTTACACCCCCCCATC
CCTACCTATCTTCTTCTTTTTGTTACTTTCATCGGAAGGGAAGTAGAGATACCAGAGGGG
CCCCCCGGGAGGGTCATTATGAGAAGCCTTAAACACTCTCGGGGGCTCCCTAAAGAAGGG
CCCCCGGAGTGTCTGGGCTTCTCTAATAATCCCTCCCTAAAGGCCTTCCTCCACTATCTC
TACTTCACTAGTAATACAGCTTGAATAACTTTCAAAACTCTTAGTAAAGACGAGGCTAGT
AAGCCTTACCTATACTTACTTCTACACACCTTACCTACACACCACCCAAGTGGGGCCCCC
CCCTCCCCCCCTGCACACCTCTACGCTAACGAGAAGTGCGCAAGGGAAAAGTCTACGCCG
ACTAAAGACTCATCCGCTCGGGGAGCCCGCGGGAATCCCGCCTCTACAGGGCAGCCCACG
ACCGCCAGCCCATTGTCTGCCAGCTCCACCACCCTATTTTATATAGTAGTCCTATTTAAT
CATCTACAGACAGAGACCACAAAA
>HmenM_UR_3_8 [613 - 666]
ATCATCTACAGACAGAGACCACAAAATAACCAACCACCACTCAAAGCCTACGCT
>HmenM_UR_3_9 [638 - 667]
ATAACCAACCACCACTCAAAGCCTACGCTT
>HmenM_UR_3_10 [656 - 621] (REVERSE SENSE)
TTGAGTGGTGGTTGGTTATTTTGTGGTCTCTGTCTG
>HmenM_UR_3_11 [645 - 613] (REVERSE SENSE)
TTGGTTATTTTGTGGTCTCTGTCTGTAGATGAT
```

```
>HmenM_UR_3_12 [652 - 596] (REVERSE SENSE)
GTGGTGGTTGGTTATTTTGTGGTCTCTGTCTGTAGATGATTAAATAGGACTACTATA
>HmenM_UR_3_13 [609 - 541] (REVERSE SENSE)
ATAGGACTACTATATAAAATAGGGTGGTGGAGCTGGCAGACAATGGGCTGGCGGTCGTGG
GCTGCCCTG
>HmenM_UR_3_14 [586 - 458] (REVERSE SENSE)
GTGGTGGAGCTGGCAGACAATGGGCTGGCGGTCGTGGGCTGCCCTGTAGAGGCGGGATTC
CCGCGGGCTCCCCGAGCGGATGAGTCTTTAGTCGGCGTAGACTTTTCCCTTGCGCACTTC
TCGTTAGCG
>HmenM_UR_3_15 [450 - 412] (REVERSE SENSE)
GTGCAGGGGGGGAGGGGGGGGCCCCACTTGGGTGGTGTG
>HmenM_UR_3_16 [530 - 408] (REVERSE SENSE)
ATTCCCGCGGGCTCCCCGAGCGGATGAGTCTTTAGTCGGCGTAGACTTTTCCCTTGCGCA
CTTCTCGTTAGCGTAGAGGTGTGCAGGGGGGGAGGGGGGGGCCCCACTTGGGTGGTGTGT
AGG
>HmenM_UR_3_17 [349 - 317] (REVERSE SENSE)
TTGAAAGTTATTCAAGCTGTATTACTAGTGAAG
>HmenM_UR_3_18 [329 - 294] (REVERSE SENSE)
ATTACTAGTGAAGTAGAGATAGTGGAGGAAGGCCTT
>HmenM_UR_3_19 [214 - 143] (REVERSE SENSE)
ATAATGACCCTCCCGGGGGGCCCCTCTGGTATCTCTACTTCCCTTCCGATGAAAGTAACA
AAAAGAAGAAGA
>HmenM_UR_3_20 [284 - 123] (REVERSE SENSE)
ATTATTAGAGAAGCCCAGACACTCCGGGGGCCCTTCTTTAGGGAGCCCCCGAGAGTGTTT
AAGGCTTCTCATAATGACCCTCCCGGGGGGCCCCTCTGGTATCTCTACTTCCCTTCCGAT
GAAAGTAACAAAAAGAAGAAGATAGGTAGGGATGGGGGGGTG
>HmenM_UR_3_21 [133 - 98] (REVERSE SENSE)
ATGGGGGGGTGTAAGGGGGGGAAGGGTATGGTCAAG
>HmenM_UR_3_22 [95 - 27] (REVERSE SENSE)
ATTGTTGGTGTTTATATTAATTTATCTATTATTGTTTGTTTGAGTAATCTGGTCGGTTGT
TTGTGGTTG
>HmenM_UR_3_23 [94 - 2] (REVERSE SENSE)
TTGTTGGTGTTTATATTAATTTATCTATTATTGTTTGTTTGAGTAATCTGGTCGGTTGTT
TGTGGTTGTAGTTGTTGTGCGTTTAATTACGTA
>HmenM_UR_3_24 [39 - 1] (REVERSE SENSE)
TTGTTTGTGGTTGTAGTTGTTGTGCGTTTAATTACGTAT
>HmenM_UR_4_1 [38 - 3] (REVERSE SENSE)
TTGGTGGGCGTGGTTAGAGGAAGGCTTAGGCGTTAC
>HmenM_UR_6_1 [31 - 2] (REVERSE SENSE)
GTGTGTGTTTGTGTAGCCTATTTGGTTGGG
>HmenM_UR_14_1 [12 - 68]
ATAACCCAAAAACCAGTTAAACCACAAGCTTACACCTGTACGCCAACAACACAAAAC
>HmenM_UR_14_2 [63 - 31] (REVERSE SENSE)
GTGTTGTTGGCGTACAGGTGTAAGCTTGTGGTT
>HmenM_UR_14_3 [65 - 3] (REVERSE SENSE)
TTGTGTTGTTGGCGTACAGGTGTAAGCTTGTGGTTTAACTGGTTTTTGGGTTATGAAATA
GAA
>HmenM_UR_17_1 [10 - 45]
ATAATCCAACCAAAATTTAAACAAGAGAAGATAAAT
>HmenM_UR_17_2 [64 - 132]
ATCCCGCCTCCACAGGGCAGCCCACGACCGCCAGCCCATTGTCTGCCAGCTCCCACCCCC
CCTATTTTT
```

>HmenM_UR_17_3 [48 - 137]
ATAAGAGCCCGCGGGAATCCCGCCTCCACAGGGCAGCCCACGACCGCCAGCCCATTGTCT
GCCAGCTCCCACCCCCCCTATTTTTTAGCG
>HmenM_UR_17_4 [44 - 172]
ATTAATAAGAGCCCGCGGGAATCCCGCCTCCACAGGGCAGCCCACGACCGCCAGCCCATT
GTCTGCCAGCTCCCACCCCCCCTATTTTTTAGCGTAAACCTATTTAATAACCATAGCCAA
ACAAGCTAT
>HmenM_UR_17_5 [190 - 222]
ATAACAAAACAAAAACAAAAGTACAAGCTTACC
>HmenM_UR_17_6 [225 - 272]
ATAATAAACACAAACAACACTCAAGTAAATCCTACACAACACACAGAC
>HmenM_UR_17_7 [266 - 225] (REVERSE SENSE)
GTGTTGTGTAGGATTTACTTGAGTGTTGTTTGTGTTTATTAT
>HmenM_UR_17_8 [268 - 221] (REVERSE SENSE)
GTGTGTTGTGTAGGATTTACTTGAGTGTTGTTTGTGTTTATTATTAGG
>HmenM_UR_17_9 [237 - 175] (REVERSE SENSE)
TTGTGTTTATTATTAGGTAAGCTTGTACTTTTGTTTTTGTTTTGTTATAGGCAGCCTCTT
ATT
>HmenM_UR_17_10 [215 - 150] (REVERSE SENSE)
TTGTACTTTTGTTTTTGTTTTGTTATAGGCAGCCTCTTATTTAATAGCTTGTTTGGCTAT
GGTTAT
>HmenM_UR_17_11 [128 - 48] (REVERSE SENSE)
ATAGGGGGGGTGGGAGCTGGCAGACAATGGGCTGGCGGTCGTGGGCTGCCCTGTGGAGGC
GGGATTCCCGCGGGCTCTTAT
>HmenM_UR_17_12 [88 - 29] (REVERSE SENSE)
GTGGGCTGCCCTGTGGAGGCGGGATTCCCGCGGGCTCTTATTAATTTATCTTCTCTTGTT
>HmenM_UR_17_13 [41 - 3] (REVERSE SENSE)
ATCTTCTCTTGTTTAAATTTTGGTTGGATTATTGAGTTA
>HmenM_UR_17_14 [102 - 1] (REVERSE SENSE)
ATGGGCTGGCGGTCGTGGGCTGCCCTGTGGAGGCGGGATTCCCGCGGGCTCTTATTAATT
TATCTTCTCTTGTTTAAATTTTGGTTGGATTATTGAGTTAGG
>HmenM_UR_20_1 [50 - 3] (REVERSE SENSE)
TTGAGGGTAGTTAACCGCTTAGGGGTCGGGTTTAGTGGTGCGGTGGGT
>HmenM_UR_21_1 [1 - 30]
ATACACAGCAACGCAAACTCATCAAAAATA
>HmenM_UR_21_2 [52 - 96]
GTGAGGGCCCACACCTACCTGAACCCCACCACGCACAAAGCACCT
>HmenM_UR_21_3 [109 - 174]
ATAATAAATCACAACTATACAATAAACATGCCCCTGCCCCCGGTGAACAACCCACCCAAT
TGCCAA
>HmenM_UR_21_4 [21 - 176]
ATCAAAAATATAAAAATCTTAAAACTTTACGGTGAGGGCCCACACCTACCTGAACCCCAC
CACGCACAAAGCACCTTAAGCTCATCCAATAATAAATCACAACTATACAATAAACATGCC
CCTGCCCCCGGTGAACAACCCACCCAATTGCCAACC
>HmenM_UR_21_5 [167 - 126] (REVERSE SENSE)
TTGGGTGGGTTGTTCACCGGGGGCAGGGGCATGTTTATTGTA
>HmenM_UR_21_6 [163 - 50] (REVERSE SENSE)
GTGGGTTGTTCACCGGGGGCAGGGGCATGTTTATTGTATAGTTGTGATTTATTATTGGAT
GAGCTTAAGGTGCTTTGTGCGTGGTGGGGTTCAGGTAGGTGTGGGCCCTCACCG
>HmenM_UR_21_7 [37 - 2] (REVERSE SENSE)
ATTTTTATATTTTTGATGAGTTTGCGTTGCTGTGTA

>HmenM_UR_21_8 [174 - 1] (REVERSE SENSE)
TTGGCAATTGGGTGGGTTGTTCACCGGGGGCAGGGGCATGTTTATTGTATAGTTGTGATT
TATTATTGGATGAGCTTAAGGTGCTTTGTGCGTGGTGGGGTTCAGGTAGGTGTGGGCCCT
CACCGTAAAGTTTTAAGATTTTTATATTTTTGATGAGTTTGCGTTGCTGTGTAT
>HmenM_UR_25_1 [3 - 41]
ATTAACTGAACCACAACATTACCGAACCCTATAACCACT
>HmenM_UR_25_2 [40 - 2] (REVERSE SENSE)
GTGGTTATAGGGTTCGGTAATGTTGTGGTTCAGTTAATG
>HmenM_UR_26_1 [34 - 2] (REVERSE SENSE)
TTGTTGCCTGTTGTTGCTGTATTTGCGTCTGCT
>HmenM_UR_26_2 [78 - 1] (REVERSE SENSE)
ATTGAGGTTTGGTTAGGTAGTGCGGGCAGGTTTAGGTTAGCAGGTTGTTGCCTGTTGTTG
CTGTATTTGCGTCTGCTT
>HmenM_UR_28_1 [2 - 40]
ATTGCACACAACCTCGGTGTGTTAAACAGCTATAATCAA

• ***Cumberlandia monodonta* M**

>CmonM_UR_1_1 [20 - 55]
ATTAAAAGCACCTACCACAAACGCCTCACCAACCTC
>CmonM_UR_1_2 [12 - 56]
ATCTTTCAATTAAAAGCACCTACCACAAACGCCTCACCAACCTCC
>CmonM_UR_1_3 [37 - 2] (REVERSE SENSE)
GTGGTAGGTGCTTTTAATTGAAAGATGCAGTGTGTG
>CmonM_UR_1_4 [30 - 1] (REVERSE SENSE)
GTGCTTTTAATTGAAAGATGCAGTGTGTGA
>CmonM_UR_2_1 [4 - 144]
ATGCCTCCAAACCCCGGAAACAACATTACCGCCTTCCCTTATTATTACTTAATAGTAACT
TTATTCACTATAAAACCGACAATATTTAATAAATTTACTCTTTTCATAAAGCCCCCTAAA
CCGGGGGCTCAAATTAAAAAC
>CmonM_UR_2_2 [135 - 67] (REVERSE SENSE)
TTGAGCCCCCGGTTTAGGGGGCTTTATGAAAAGAGTAAATTTATTAAATATTGTCGGTTT
TATAGTGAA
>CmonM_UR_2_3 [137 - 51] (REVERSE SENSE)
ATTTGAGCCCCCGGTTTAGGGGGCTTTATGAAAAGAGTAAATTTATTAAATATTGTCGGT
TTTATAGTGAATAAAGTTACTATTAAG
>CmonM_UR_2_4 [67 - 32] (REVERSE SENSE)
ATAAAGTTACTATTAAGTAATAATAAGGGAAGGCGG
>CmonM_UR_2_5 [48 - 1] (REVERSE SENSE)
ATAATAAGGGAAGGCGGTAATGTTGTTTCCGGGGTTTGGAGGCATTTT
>CmonM_UR_15_1 [9 - 38]
ATCCCCAAAAAAACTAAAACAATACGTGTA
>CmonM_UR_15_2 [31 - 2] (REVERSE SENSE)
ATTGTTTTAGTTTTTTTGGGGATGTGTTAT
>CmonM_UR_16_1 [13 - 45]
ATAGGCTATAACAAGCGCTTAATCTTGCCCACA
>CmonM_UR_16_2 [78 - 125]
ATAAATACTCTAACTTATCTACGCTTGCTTTATGTATGACAGTTACTC
>CmonM_UR_16_3 [94 - 147]
ATCTACGCTTGCTTTATGTATGACAGTTACTCTAACTGATTTAGTTACTCTTTA
>CmonM_UR_16_4 [113 - 214]
ATGACAGTTACTCTAACTGATTTAGTTACTCTTTATAGGGTTCTCCATCCTTCTCTCTTT
TTATACTTTAAACTACCTTGCAAAGAAACCCTTAATAGGCTA

>CmonM_UR_16_5 [147 - 239]
ATAGGGTTCTCCATCCTTCTCTCTTTTTATACTTTAAACTACCTTGCAAAGAAACCCTTA
ATAGGCTATAACAAGCGCTTAATCTTGCCCACA
>CmonM_UR_16_6 [272 - 319]
ATAAATACTCTAACTTATCTACGCTTGCTTTATGTATGACAGTTACTC
>CmonM_UR_16_7 [288 - 341]
ATCTACGCTTGCTTTATGTATGACAGTTACTCTAACTGATTTAGTTACTCTTTA
>CmonM_UR_16_8 [307 - 378]
ATGACAGTTACTCTAACTGATTTAGTTACTCTTTATAGGGTTCTCCATCCTTCTCTCTTT
TTATACTTTAAC
>CmonM_UR_16_9 [341 - 439]
ATAGGGTTCTCCATCCTTCTCTCTTTTTATACTTTAACTAAACAACTTCCCTGCCAAACC
CTTCCTCCCACCTCAATTGTAAGAATCTTAATTACTAGT
>CmonM_UR_16_10 [450 - 497]
ATTATTGTAACTAAAAATTCCTGATCAGGGCAATATAGGCGCTCACCA
>CmonM_UR_16_11 [466 - 519]
ATTCCTGATCAGGGCAATATAGGCGCTCACCATAACGTAAATAATGCGGCCATG
>CmonM_UR_16_12 [510 - 463] (REVERSE SENSE)
ATTATTTACGTTATGGTGAGCGCCTATATTGCCCTGATCAGGAATTTT
>CmonM_UR_16_13 [518 - 438] (REVERSE SENSE)
ATGGCCGCATTATTTACGTTATGGTGAGCGCCTATATTGCCCTGATCAGGAATTTTTAGT
TACAATAATGTTTTGTTTAAC
>CmonM_UR_16_14 [416 - 381] (REVERSE SENSE)
TTGAGGTGGGAGGAAGGGTTTGGCAGGGAAGTTGTT
>CmonM_UR_16_15 [432 - 370] (REVERSE SENSE)
ATTAAGATTCTTACAATTGAGGTGGGAGGAAGGGTTTGGCAGGGAAGTTGTTTAGTTAAA
GTA
>CmonM_UR_16_16 [370 - 335] (REVERSE SENSE)
ATAAAAAGAGAGAAGGATGGAGAACCCTATAAAGAG
>CmonM_UR_16_17 [308 - 264] (REVERSE SENSE)
ATACATAAAGCAAGCGTAGATAAGTTAGAGTATTTATGAGGACTT
>CmonM_UR_16_18 [277 - 248] (REVERSE SENSE)
ATTTATGAGGACTTTAGGTTATTAACCCAT
>CmonM_UR_16_19 [257 - 228] (REVERSE SENSE)
ATTAACCCATTAGTTTTATGTGGGCAAGAT
>CmonM_UR_16_20 [249 - 148] (REVERSE SENSE)
ATTAGTTTTATGTGGGCAAGATTAAGCGCTTGTTATAGCCTATTAAGGGTTTCTTTGCAA
GGTAGTTTAAAGTATAAAAAGAGAGAAGGATGGAGAACCCTA
>CmonM_UR_16_21 [176 - 141] (REVERSE SENSE)
ATAAAAAGAGAGAAGGATGGAGAACCCTATAAAGAG
>CmonM_UR_16_22 [114 - 70] (REVERSE SENSE)
ATACATAAAGCAAGCGTAGATAAGTTAGAGTATTTATGAGGACTT
>CmonM_UR_16_23 [83 - 54] (REVERSE SENSE)
ATTTATGAGGACTTTAGGTTATTAACCCAT
>CmonM_UR_16_24 [63 - 34] (REVERSE SENSE)
ATTAACCCATTAGTTTTATGTGGGCAAGAT
>CmonM_UR_16_25 [55 - 2] (REVERSE SENSE)
ATTAGTTTTATGTGGGCAAGATTAAGCGCTTGTTATAGCCTATTAAGGGTTTCT
>CmonM_UR_24_1 [63 - 131]
ATTACCCTAAAAAAACCGAAAGAAGAACAAATAGGGTGACAAGCCAAAGCACACGAACAC
AAAACCGAC

>CmonM_UR_24_2 [98 - 271]
GTGACAAGCCAAAGCACACGAACACAAAACCGACTAAATCACTTCAACACTTTTATTGAC
ATAGCCCCTAACCGCACCCAACCACTCCCCCCCACTCTAAAGTCAACCTTAATGCTCTTC
CACAATCCCTTAAGCCCTCGCTTAACTTTCTTAACAAAAACCTTCCGCCGCTCA
>CmonM_UR_24_3 [271 - 324]
ATAAACTCACATAAACGCACTCACACAAATACAAACCAAAACTGCAATAATAAG
>CmonM_UR_24_4 [281 - 382]
ATAAACGCACTCACACAAATACAAACCAAAACTGCAATAATAAGTAAAAATAAGCTGTTT
TCGTGAAAGAACTCACGAATTGCCCCAAAAAGAGCTTTCAAA
>CmonM_UR_24_5 [343 - 396]
GTGAAAGAACTCACGAATTGCCCCAAAAAGAGCTTTCAAATAAAGGGCACCATC
>CmonM_UR_24_6 [330 - 458]
ATAAGCTGTTTTCGTGAAAGAACTCACGAATTGCCCCAAAAAGAGCTTTCAAATAAAGGG
CACCATCTAGCACAAGCGGGACCTCAACCCCACAATCTACCCTTCTTAAGCACACCACTA
AACCAGCCC
>CmonM_UR_24_7 [442 - 251] (REVERSE SENSE)
GTGCTTAAGAAGGGTAGATTGTGGGGTTGAGGTCCCGCTTGTGCTAGATGGTGCCCTTTA
TTTGAAAGCTCTTTTTGGGGCAATTCGTGAGTTCTTTCACGAAAACAGCTTATTTTTACT
TATTATTGCAGTTTTGGTTTGTATTTGTGTGAGTGCGTTTATGTGAGTTTATGAGCGGCG
GAAGGTTTTTGT
>CmonM_UR_24_8 [395 - 168] (REVERSE SENSE)
ATGGTGCCCTTTATTTGAAAGCTCTTTTTGGGGCAATTCGTGAGTTCTTTCACGAAAACA
GCTTATTTTTACTTATTATTGCAGTTTTGGTTTGTATTTGTGTGAGTGCGTTTATGTGAG
TTTATGAGCGGCGGAAGGTTTTTGTTAAGAAAGTTAAGCGAGGGCTTAAGGGATTGTGGA
AGAGCATTAAGGTTGACTTTAGAGTGGGGGGGAGTGGTTGGGTGCGGT
>CmonM_UR_24_9 [178 - 134] (REVERSE SENSE)
TTGGGTGCGGTTAGGGGCTATGTCAATAAAAGTGTTGAAGTGATT
>CmonM_UR_24_10 [122 - 72] (REVERSE SENSE)
GTGTTCGTGTGCTTTGGCTTGTCACCCTATTTGTTCTTCTTTCGGTTTTTT
>CmonM_UR_24_11 [447 - 67] (REVERSE SENSE)
GTGGTGTGCTTAAGAAGGGTAGATTGTGGGGTTGAGGTCCCGCTTGTGCTAGATGGTGCC
CTTTATTTGAAAGCTCTTTTTGGGGCAATTCGTGAGTTCTTTCACGAAAACAGCTTATTT
TTACTTATTATTGCAGTTTTGGTTTGTATTTGTGTGAGTGCGTTTATGTGAGTTTATGAG
CGGCGGAAGGTTTTTGTTAAGAAAGTTAAGCGAGGGCTTAAGGGATTGTGGAAGAGCATT
AAGGTTGACTTTAGAGTGGGGGGGAGTGGTTGGGTGCGGTTAGGGGCTATGTCAATAAAA
GTGTTGAAGTGATTTAGTCGGTTTTGTGTTCGTGTGCTTTGGCTTGTCACCCTATTTGTT
CTTCTTTCGGTTTTTTTAGGG
>CmonM_UR_24_12 [124 - 32] (REVERSE SENSE)
TTGTGTTCGTGTGCTTTGGCTTGTCACCCTATTTGTTCTTCTTTCGGTTTTTTTAGGGTA
ATTGTAGTGGTGGTTTGGCAGTTGTTTAGTGCC
>CmonM_UR_27_1 [2 - 181]
ATTTATTTGCTCTTCTTACCAAATTTAACCCTACCCACACCCCGCACAAAAAACCCACCC
ACCCGAGACTTAACCAAAAGTGGGTTCTTATCTTTGATAACCCCCCCCAAATTCGAACTC
TTAACAACCATCTTTACCGCCTTCTTACACTCCGCAACCCAACGACTAAGCTTACGCTCA
>CmonM_UR_27_2 [91 - 195]
ATCTTTGATAACCCCCCCCAAATTCGAACTCTTAACAACCATCTTTACCGCCTTCTTACA
CTCCGCAACCCAACGACTAAGCTTACGCTCATAATCAGCCCGATA
>CmonM_UR_27_3 [194 - 138] (REVERSE SENSE)
ATCGGGCTGATTATGAGCGTAAGCTTAGTCGTTGGGTTGCGGAGTGTAAGAAGGCGG
>CmonM_UR_27_4 [132 - 7] (REVERSE SENSE)
ATGGTTGTTAAGAGTTCGAATTTGGGGGGGGTTATCAAAGATAAGAACCCACTTTTGGTT
AAGTCTCGGGTGGGTGGGTTTTTTGTGCGGGGTGTGGGTAGGGTTAAATTTGGTAAGAAG
AGCAAA

>CmonM_UR_27_5 [128 - 3] (REVERSE SENSE)
TTGTTAAGAGTTCGAATTTGGGGGGGGTTATCAAAGATAAGAACCCACTTTTGGTTAAGT
CTCGGGTGGGTGGGTTTTTTGTGCGGGGTGTGGGTAGGGTTAAATTTGGTAAGAAGAGCA
AATAAA

PROTEIN SEQUENCES

These sequences have been obtained by translating the preceding nucleotide sequences using the mitochondrial genetic code. Here we show only the translation made using the option `-methionine no` in `getorf` to translate the start codon as the corresponding amino acid and not as a methionine.

• *Neotrigonia margaritacea*

```
>Nmar_UR_2_1 [1 - 30]
IHLTSLPSPS
>Nmar_UR_2_2 [30 - 59]
MKTKHYLLLP
>Nmar_UR_2_3 [5 - 61]
IWPVYLDPDKNKTLFIITP
>Nmar_UR_2_4 [54 - 19] (REVERSE SENSE)
MMNNVLFLSGSS
>Nmar_UR_2_5 [47 - 3] (REVERSE SENSE)
MMFCFYLGLGKLVSW
>Nmar_UR_3_1 [6 - 35]
ITLIHLNQHL
>Nmar_UR_3_2 [57 - 92]
ISSLILLPKFSD
>Nmar_UR_3_3 [91 - 120]
IKKKYKKQHM
>Nmar_UR_3_4 [35 - 154]
MMTYQQVNFFTYPFTKIFWLKKNMKNNTYNFMPLLIHPNQ
>Nmar_UR_3_5 [167 - 220]
IFQPFISYPQFYTFFPTE
>Nmar_UR_3_6 [120 - 254]
MILYLSLFTQTNNFTTFFNPSFLTLNFMLSSPQNNNNYHNFQLPS
>Nmar_UR_3_7 [154 - 357]
MISPHFSTLHFLPSILYFLPHSMMTIIMTFNYPDKNLPLSGLHANPKMIFIFVILYTLKY
NPYSKTFL
>Nmar_UR_3_8 [357 - 407]
MILKIMFLNTRSKKLEI
>Nmar_UR_3_9 [332 - 487]
MILTPKLFYNSKNHILKHPKKKTRNLTPPPLNFYAMGARKMVSPRKTLYSQF
>Nmar_UR_3_10 [484 - 537]
ILTKHFFFEPPWMNLSVQ
>Nmar_UR_3_11 [516 - 554]
MNKSISAMESMQL
>Nmar_UR_3_12 [524 - 607]
IYQCNSIHATLMQKYLLGVIFHSANRWP
>Nmar_UR_3_13 [576 - 623]
VWFFTRRTADHNSSTI
>Nmar_UR_3_14 [565 - 654]
MPSSCDFSLGEPLTMILALFSMIFLFGNMI
>Nmar_UR_3_15 [639 - 713]
IRKHNLVFFFLFFFHLRQQLLKQQW
>Nmar_UR_3_16 [710 - 766]
MMKKNQKKGTQPKSHCQEL
```

```
>Nmar_UR_3_17 [776 - 814]
MGWILVSSCFTPK
>Nmar_UR_3_18 [753 - 830]
IAKNYKRQWGGFSYQVASLRSKGMPN
>Nmar_UR_3_19 [748 - 840]
ITLPSIMNVNGVDSRIKLLHSEVKGCPISAL
>Nmar_UR_3_20 [848 - 1006]
IFTVRSSTVAPPTRCSSLQFEAGGSSFIPQRSLAPPAGSSLTFVLPSPTWVIP
>Nmar_UR_3_21 [898 - 1050]
ITVRGRGLFLYSSTVARSPGWFVSHFCSPFAHLSHPLTPRSTCSLENPPPQ
>Nmar_UR_3_22 [1031 - 1060]
LSIHPHNSAA
>Nmar_UR_3_23 [840 - 1088]
MAQSLPFVPQPSLPRLAARHYSSSPGALPLFLNGRSLPRLVRLSLLFSLRPLESSLNSPF
HMLPWESTPTMELHKSNMLYSIL
>Nmar_UR_3_24 [1146 - 1181]
MLILSFSINMVK
>Nmar_UR_3_25 [1060 - 1206]
MKAMYSIQFYNKKEDFMITSTGIFTCFHKYWFYHFQLMWLNKEMILMKL
>Nmar_UR_3_26 [1328 - 1381]
MKFNNKNFPIKHHLNFTY
>Nmar_UR_3_27 [1203 - 1391]
IMELFCVNMKESLFTKKSKTLSFPFPLTINNYFLKKYLNSIKWNLMTKISQSNITSTLHT
SKL
>Nmar_UR_3_28 [1378 - 1416]
MLENYKLYSLNNI
>Nmar_UR_3_29 [1371 - 1327] (REVERSE SENSE)
LSWCLIGKFLLLNFI
>Nmar_UR_3_30 [1405 - 1298] (REVERSE SENSE)
MNMVYSFLVCKVEVMFDWEIFVIKFHLIEFKYFFKK
>Nmar_UR_3_31 [1323 - 1291] (REVERSE SENSE)
LNLNIFLSNNY
>Nmar_UR_3_32 [1199 - 1158] (REVERSE SENSE)
IKIISLFNHINWKW
>Nmar_UR_3_33 [1288 - 1133] (REVERSE SENSE)
MVKGKGKLKVFLFFVKSLSFMLTQNNSMISSKLFLYLTMLIENDKINIYENM
>Nmar_UR_3_34 [1127 - 1089] (REVERSE SENSE)
MPVLVIMKSSFLL
>Nmar_UR_3_35 [1167 - 1009] (REVERSE SENSE)
LKMMKSMFMKTCKDTCTSNYEIFFFIMELNSVYCFYAALLWGWILKGACGTGS
>Nmar_UR_3_36 [1079 - 930] (REVERSE SENSE)
MEYIAFMQLYCGGGFSSEHVERGVKGWLKWAKGEQKWETNQPGERATVEE
>Nmar_UR_3_37 [1072 - 902] (REVERSE SENSE)
MLLLCSSIVGVDSQGSMWNGELSDDSSGRSENKSESRTSRGSERPLSNKGSAPGLEL
>Nmar_UR_3_38 [899 - 855] (REVERSE SENSE)
MTSSESGERRLSNER
>Nmar_UR_3_39 [1002 - 841] (REVERSE SENSE)
MTQVGEGSTKVSDEPAGGASDRWGMKEEPPASNCNDEQRVGGATVEERTVKIEL
>Nmar_UR_3_40 [829 - 767] (REVERSE SENSE)
LGIPLLRSEATWYENPPHWRL
>Nmar_UR_3_41 [848 - 702] (REVERSE SENSE)
LSYSALIGHPFTSEWSNLMRESTPLTFMILGNVIWAGSLFFGSFLSLLF
>Nmar_UR_3_42 [807 - 640] (REVERSE SENSE)
VKQLDTSIHPIDVYNSWQCDLGWVPFFWFFFIIVVLGVVVVDEKKSEKSSLNYVSE
```

>Nmar_UR_3_43 [760 - 626] (REVERSE SENSE)
LAMWFGLGPFFLVLFYHCCFSSCCRSWKKKSKKKTKLCFRMGKSF
>Nmar_UR_3_44 [622 - 575] (REVERSE SENSE)
MVLELWSAVRRVKNHT
>Nmar_UR_3_45 [582 - 529] (REVERSE SENSE)
ITPSSYFCISVAWILLHW
>Nmar_UR_3_46 [547 - 494] (REVERSE SENSE)
MDSIALMDLFMGARKSNV
>Nmar_UR_3_47 [525 - 490] (REVERSE SENSE)
IYSWGLEKEMFS
>Nmar_UR_3_48 [653 - 381] (REVERSE SENSE)
IMFPNSKIILNSASIMVSGSPSEKSHLEGISVLELHGFYCTDSFIHGGSKKKCLVKIVYM
VFSVVMLFFEPPWRKSSKGGGLNFEFFSSGV
>Nmar_UR_3_49 [406 - 305] (REVERSE SENSE)
ISSFFLRVFKNMIFSIMEKFWSKDYILSYMKWRK
>Nmar_UR_3_50 [314 - 249] (REVERSE SENSE)
MTKMKIILGFACKPLSGSFLSG
>Nmar_UR_3_51 [286 - 233] (REVERSE SENSE)
LHVNPLVGGFYLGNWKLW
>Nmar_UR_3_52 [255 - 202] (REVERSE SENSE)
IWVIESYDNCYYSVGKKV
>Nmar_UR_3_53 [245 - 141] (REVERSE SENSE)
LKVMMIVIILWGSKYKIEGKKWSVEKCGEIIGLGE
>Nmar_UR_3_54 [229 - 95] (REVERSE SENSE)
LLLFCGEESMKLSVSNEGLKNVVKLLVWVNKESYKIMCVVFYIFF
>Nmar_UR_3_55 [119 - 78] (REVERSE SENSE)
MCCFLYFFLISKFW
>Nmar_UR_3_56 [183 - 70] (REVERSE SENSE)
MKGWKMWWNYWFGWMSSGMKLYVLFFMFFFNQKILVKG
>Nmar_UR_3_57 [39 - 10] (REVERSE SENSE)
IMSVGLNELK
>Nmar_UR_3_58 [70 - 2] (REVERSE SENSE)
MSEEINLLVSYYKCWFKWIKVIL
>Nmar_UR_14_1 [9 - 47]
MIINFMQIHCLMC
>Nmar_UR_14_2 [5 - 79]
MDNYQFYTNSLFNMLIIMIKQMMMM
>Nmar_UR_14_3 [78 - 25] (REVERSE SENSE)
IIIICLIMMISMLNNEFV
>Nmar_UR_14_4 [35 - 3] (REVERSE SENSE)
MNLYKIDNYLY
>Nmar_UR_14_5 [43 - 2] (REVERSE SENSE)
IKQWICMKLMIIYI
>Nmar_UR_20_1 [14 - 58]
ITSSKNKTFSMSIIN
>Nmar_UR_20_2 [57 - 1] (REVERSE SENSE)
LMILMLNVLFFDDVIDYNW
>Nmar_UR_21_1 [10 - 39]
ISQIKYKSSA
>Nmar_UR_22_1 [6 - 35]
MLMTMYLTLT
>Nmar_UR_22_2 [8 - 40]
INNHMFNFDLK

```
>Nmar_UR_23_1 [11 - 52]
MFQLMLQFFNINNP
>Nmar_UR_23_2 [39 - 95]
MLMTLSPQQHYVNKHIYNN
>Nmar_UR_23_3 [95 - 45] (REVERSE SENSE)
IIVDVFVYMVLLGSKGY
>Nmar_UR_23_4 [42 - 1] (REVERSE SENSE)
MLKNCNINWNISLK
>Nmar_UR_24_1 [24 - 56]
IYSKFSINLNL
>Nmar_UR_24_2 [28 - 63]
MVNFLSTSTYNL
```

• *Anodontites trapesialis*

```
>Atra_UR_2_1 [1 - 33]
ISPNMGPTQPM
>Atra_UR_2_2 [32 - 3] (REVERSE SENSE)
MGWVGPMLGE
>Atra_UR_3_1 [68 - 24] (REVERSE SENSE)
LFISEDYLIIMDVNY
>Atra_UR_13_1 [2 - 31]
IFESNKKMNF
>Atra_UR_13_2 [37 - 78]
MHFHMHLPTPYLYS
>Atra_UR_13_3 [27 - 104]
IFNYTFSHTPAYTVFMFLMLMTLLPW
>Atra_UR_13_4 [115 - 150]
MFWFFQCYSIYL
>Atra_UR_13_5 [41 - 154]
IFTYTCLHRIYIPNTDDSITLVSLMYFDFFNVMVFIFN
>Atra_UR_13_6 [153 - 200]
IKFFFKKKMNSLIFNT
>Atra_UR_13_7 [242 - 189] (REVERSE SENSE)
VLCSSMYCVFLFSLCVKD
>Atra_UR_13_8 [182 - 153] (REVERSE SENSE)
IYFFFKKKLN
>Atra_UR_13_9 [147 - 73] (REVERSE SENSE)
MNTMTLKKSKYMSLTSVMESSVLGM
>Atra_UR_13_10 [223 - 62] (REVERSE SENSE)
IVCFYLDYVLKISLFIFFLKKNLIKDKYYNIEKIKMYETYQGNSVISISNMNTV
>Atra_UR_13_11 [69 - 34] (REVERSE SENSE)
MRCSQVYVKMYS
>Atra_UR_13_12 [50 - 15] (REVERSE SENSE)
MWKCMVKNLFFC
>Atra_UR_14_1 [11 - 64]
IIPTNYLQSLNYLTHKNF
>Atra_UR_14_2 [37 - 84]
IKLLNTQKLLVNKSKN
>Atra_UR_14_3 [96 - 67] (REVERSE SENSE)
VCCLIFTFIY
>Atra_UR_14_4 [94 - 50] (REVERSE SENSE)
VLFNFYFYLLEVFVC
```

>Atra_UR_14_5 [53 - 24] (REVERSE SENSE)
VLSNLMTVNS
>Atra_UR_21_1 [16 - 69]
ILGLPFLGLGGKTTLKSL
>Atra_UR_21_2 [41 - 94]
LEVKQLSKDYKSSWPPSP
>Atra_UR_21_3 [66 - 98]
IMNPPDHLLPN
>Atra_UR_21_4 [102 - 137]
IKKSMFMPISTK
>Atra_UR_21_5 [137 - 187]
MKLNMKSMMKTNHNWYP
>Atra_UR_21_6 [73 - 213]
ILLTTFSLTKLKNPYLYLSLLNKIKHKKNNKNKPQLVPMTLMQNSIE
>Atra_UR_21_7 [208 - 146] (REVERSE SENSE)
LNFVLGLWVPVVVCFYYSFYV
>Atra_UR_21_8 [177 - 136] (REVERSE SENSE)
LWFVFIILFMFNFI
>Atra_UR_21_9 [209 - 9] (REVERSE SENSE)
IEFCISVMGTSCGLFLLFFLCLILFSSDSYKYGFFNLVSEKVVSSIYNLLSVVLPPSPKK
GKPKIKK
>Atra_UR_21_10 [70 - 2] (REVERSE SENSE)
MIFWELFYLQGLKSVSLSLKSKV
>Atra_UR_22_1 [7 - 78]
MSMPSLMSKNRITTKTQPKQQTNT
>Atra_UR_22_2 [9 - 86]
IHTITNIQKSNYYKNTTQTTNEYVNL
>Atra_UR_22_3 [111 - 197]
ITKLYLPTSQMNSNTQSMNKHTKMKHQQL
>Atra_UR_22_4 [35 - 220]
IELLQKHNPNNKRMRKLMMNLIPYLDYKIMPTYLTNKLKHTKNKQTYQDKTPTIMTQNLN
KN
>Atra_UR_22_5 [213 - 275]
MKIKNTLVNNTNPTILSYMFT
>Atra_UR_22_6 [292 - 357]
MLMLPPSNQFTTKMQPKQQTNT
>Atra_UR_22_7 [318 - 365]
IYHKNTTQTTNEHMNL
>Atra_UR_22_8 [269 - 382]
IYLIFLHKCLYYHLEINLPQKYNPNNKRTHKPMMNLTS
>Atra_UR_22_9 [398 - 436]
LMVKQLFKDHILS
>Atra_UR_22_10 [385 - 453]
ILTPFDGKTTLQSPYPVLTLSFL
>Atra_UR_22_11 [429 - 391] (REVERSE SENSE)
MWSLKSCFTIKSC
>Atra_UR_22_12 [427 - 323] (REVERSE SENSE)
MVFEELFYHQKVLKLSSKVYYSFMCSFVVWVVFLW
>Atra_UR_22_13 [369 - 304] (REVERSE SENSE)
IMGLCVRLLFGLYFCGKLISSW
>Atra_UR_22_14 [356 - 261] (REVERSE SENSE)
VFVCCLGCIFVVNWFLGGSMSIYGGSLSKYMA
>Atra_UR_22_15 [270 - 238] (REVERSE SENSE)
MYSLKLLGWYC

>Atra_UR_22_16 [256 - 212] (REVERSE SENSE)
IVGLVLLTKVFLIFI
>Atra_UR_22_17 [242 - 198] (REVERSE SENSE)
IVNQSVFNFYLGFVL
>Atra_UR_22_18 [198 - 133] (REVERSE SENSE)
MIVGVLSWYVCLFFVCLSLFVS
>Atra_UR_22_19 [194 - 129] (REVERSE SENSE)
LLVFYLGMFVYSLCVWVYLWGS
>Atra_UR_22_20 [205 - 110] (REVERSE SENSE)
LCYNCWCFILVCLFILCVFEFICEVGSYNFVI
>Atra_UR_22_21 [94 - 47] (REVERSE SENSE)
IYYKFTYSFVVWVVFL
>Atra_UR_22_22 [99 - 25] (REVERSE SENSE)
MSFIMSLRIRLLFGLCFCSNSIFGY
>Atra_UR_22_23 [62 - 3] (REVERSE SENSE)
LGCVFVVIRFLDISDGMDIC
>Atra_UR_23_1 [24 - 56]
MNTTNSNYTPN
>Atra_UR_23_2 [22 - 87]
MWTQQTQIMHLTSLPSQLSTPL
>Atra_UR_23_3 [51 - 16] (REVERSE SENSE)
VYNLSLLCSYCL
>Atra_UR_23_4 [80 - 3] (REVERSE SENSE)
VDNCEGSLVSCMIWVCCVHIACKSVF
>Atra_UR_23_5 [31 - 2] (REVERSE SENSE)
VFMLLVKASS

• ***Mutela dubia***

>Mdub_UR_4_1 [16 - 45]
IYFCLVLLMN
>Mdub_UR_4_2 [9 - 116]
ISYLFLLSITYKLISQKQQIFKNLMMKFLTNLFNTM
>Mdub_UR_4_3 [77 - 48] (REVERSE SENSE)
IFKNLLFLSN
>Mdub_UR_4_4 [115 - 41] (REVERSE SENSE)
IVLNKLVKNFIIKFLKICCFWEISL
>Mdub_UR_15_1 [26 - 64]
IMISTMKLLLSII
>Mdub_UR_15_2 [75 - 110]
IMNTNALYQSTK
>Mdub_UR_15_3 [95 - 130]
MSVYQMISMLRL
>Mdub_UR_15_4 [134 - 163]
IMLTLLALCP
>Mdub_UR_15_5 [88 - 231]
MLYISLPNNFNVTPLNYTNTISLMPMINYMQQKLAAEDWMKSGLLFNF
>Mdub_UR_15_6 [227 - 274]
IFNFWVLVHMSAQAVQ
>Mdub_UR_15_7 [238 - 318]
LGFGSYERASGAMETSIIMVNDWRDVY
>Mdub_UR_15_8 [358 - 444]
VVGSPQAELLGVTGMVDWWVSRLPGGMLS

```
>Mdub_UR_15_9 [299 - 466]
MIDGMCTNPLDFNLGMEASEWWGVRKRSYWESLVWLIGGWGGSQGVYFPKPNFSFN
>Mdub_UR_15_10 [234 - 485]
IFGFWFMWARKRCNSDINHYSEWLTGCVLTHLTLIWGWSRGSGGESASGVIGSHWYGWLV
GEAAPSGYTFLNQILASTSVQQVT
>Mdub_UR_15_11 [539 - 625]
VNWGWETVGWTVQGHLYSTKGTYENLPLN
>Mdub_UR_15_12 [514 - 669]
MLSLPPIMCELGVGDGWLNCPSAPLQNQGYLWEPAFELELPSTAQYGGPEPS
>Mdub_UR_15_13 [716 - 745]
MVLWSDCSAL
>Mdub_UR_15_14 [752 - 790]
IMSMHQVWLTVEL
>Mdub_UR_15_15 [537 - 797]
VWIGGGSRLVELSKGTSTEPSVLMSTCLWISASEHSTMRWTGAIVDLNFSYLGSGLYGLL
WSYGVIAEHCKMLWECISCDWLWNFKL
>Mdub_UR_15_16 [807 - 839]
IYSMDDSFFFI
>Mdub_UR_15_17 [691 - 897]
LALGSTASYGPMEWLQSIVKYYENASGVIDCGTLNFKTSFTGWMIPFFSFNYKFFKLMTF
CLHLHMQIF
>Mdub_UR_15_18 [858 - 905]
INNLLSTPTHTNFLKN
>Mdub_UR_15_19 [893 - 846] (REVERSE SENSE)
ICMCSCSQKVINLKNL
>Mdub_UR_15_20 [846 - 793] (REVERSE SENSE)
MIKWKKSNHPSCKSRFKV
>Mdub_UR_15_21 [824 - 756] (REVERSE SENSE)
IIHPVNLVLKFKVPQSITPDAFS
>Mdub_UR_15_22 [766 - 725] (REVERSE SENSE)
MHSHNILQCSAITP
>Mdub_UR_15_23 [762 - 685] (REVERSE SENSE)
ILMMFYNALQSLHSTMGGRSAQSQGS
>Mdub_UR_15_24 [641 - 510] (REVERSE SENSE)
VLGSSNSKAGSHKYPWFCSGALGQFNQPSPTPNSHMMGGSESIG
>Mdub_UR_15_25 [465 - 385] (REVERSE SENSE)
LKLKFGLGKYTPWEPPHPPINHTSDSQ
>Mdub_UR_15_26 [437 - 336] (REVERSE SENSE)
MPPGSRLTHQSTMPVTPNNSACGLPTTPSPPSPN
>Mdub_UR_15_27 [667 - 293] (REVERSE SENSE)
MAPVHRIVLCSEALIQSQVLMSTLGSVEVPLDSSTNRLPPPIHTWWEVGSVSVNSGFVLS
YVTCCTLVEAKIWFSKVYPLGAASPTNQPYQWLPMTPLADSPPLPRLHPQIKVKWVSTHP
VNHSL
>Mdub_UR_15_28 [293 - 234] (REVERSE SENSE)
MMIDVSIAPLARSYEPKPKN
>Mdub_UR_15_29 [286 - 227] (REVERSE SENSE)
LMSLLHRLRAHMNQNPKIKN
>Mdub_UR_15_30 [375 - 190] (REVERSE SENSE)
LRTPHHSLASIPKLKSSGLVHIPSIIHYNDWCLYCTACALMWTKTQKLKIKQEPPLYPVL
CR
>Mdub_UR_15_31 [206 - 174] (REVERSE SENSE)
IQSSAASFCCM
>Mdub_UR_15_32 [170 - 138] (REVERSE SENSE)
LIMGMSLMVLV
```

```
>Mdub_UR_15_33 [180 - 133] (REVERSE SENSE)
LHVVDYGHKANSVSMI
>Mdub_UR_15_34 [109 - 74] (REVERSE SENSE)
LVDWYSAFVFMI
>Mdub_UR_15_35 [89 - 42] (REVERSE SENSE)
ICVYNLMIKWLMMVAL
>Mdub_UR_15_36 [63 - 22] (REVERSE SENSE)
MIDNSSFMVEIMIL
>Mdub_UR_16_1 [45 - 74]
INIPNHPKST
>Mdub_UR_16_2 [40 - 90]
ILLMFLTIPSLLNMYLI
>Mdub_UR_16_3 [81 - 110]
MFNLKKYYIY
>Mdub_UR_16_4 [98 - 136]
MLYLLMNKNLLMT
>Mdub_UR_16_5 [137 - 93] (REVERSE SENSE)
LLLMSFYLLMNMVFF
>Mdub_UR_16_6 [132 - 88] (REVERSE SENSE)
INKFLFINKYSIFLN
>Mdub_UR_16_7 [89 - 12] (REVERSE SENSE)
IKYMLSSLGMVSNINSISFLFGEKKL
>Mdub_UR_16_8 [67 - 8] (REVERSE SENSE)
LGWLGMLMGLVFYLGKSSYS
>Mdub_UR_16_9 [30 - 1] (REVERSE SENSE)
IWGKEVMVSK
>Mdub_UR_23_1 [35 - 3] (REVERSE SENSE)
LVIIYFSFLIM
```

• ***Hyridella menziesii* F**

```
>HmenF_UR_1_1 [34 - 69]
MLTPHFPYHNYH
>HmenF_UR_1_2 [36 - 71]
INSTLPMPQLPH
>HmenF_UR_1_3 [69 - 40] (REVERSE SENSE)
VVIVVWEVWS
>HmenF_UR_1_4 [62 - 3] (REVERSE SENSE)
LWYGKCGVNMCLWVFSLVVL
>HmenF_UR_1_5 [55 - 2] (REVERSE SENSE)
MGSVELMCACGYLGWWFW
>HmenF_UR_2_1 [36 - 1] (REVERSE SENSE)
LSLVGDNNGGQY
>HmenF_UR_5_1 [11 - 43]
MATQPTMPSHH
>HmenF_UR_5_2 [43 - 2] (REVERSE SENSE)
VMWGYGWLCSYCWE
>HmenF_UR_5_3 [30 - 1] (REVERSE SENSE)
MVGCVAIVES
>HmenF_UR_8_1 [3 - 32]
IPSNTPQSTH
>HmenF_UR_8_2 [32 - 3] (REVERSE SENSE)
VCTLGCIWSN
```

```
>HmenF_UR_10_1 [41 - 3] (REVERSE SENSE)
LMLCCSGLFVYFI
>HmenF_UR_13_1 [3 - 44]
VVVAPRTTAIKQNF
>HmenF_UR_13_2 [38 - 3] (REVERSE SENSE)
LFNGCGSWGYDH
>HmenF_UR_13_3 [31 - 2] (REVERSE SENSE)
MAVVRGATTT
>HmenF_UR_16_1 [39 - 1] (REVERSE SENSE)
IFSFFCGLVKWLL
>HmenF_UR_17_1 [35 - 64]
MSLLKPTFTG
>HmenF_UR_17_2 [69 - 122]
IKPNQATMSSNPVFTTYK
>HmenF_UR_17_3 [37 - 129]
IFAQTNLYSLKSSQTKLPYPPTLYLQLMNKS
>HmenF_UR_17_4 [119 - 60] (REVERSE SENSE)
MSCKYSVGGYGSLVWLDFSL
>HmenF_UR_17_5 [100 - 26] (REVERSE SENSE)
LEDMVAWFGLILACKGWFEQSYASR
>HmenF_UR_17_6 [48 - 10] (REVERSE SENSE)
LSKDMLGVSGLVW
>HmenF_UR_21_1 [39 - 4] (REVERSE SENSE)
LMLGCLVLDDVG
>HmenF_UR_24_1 [6 - 62]
MQLTYTNPPLPPTQPTSTT
>HmenF_UR_24_2 [25 - 75]
ILHYRQPNPHQPPNNPV
>HmenF_UR_24_3 [53 - 88]
INHLTIPSNLTH
>HmenF_UR_24_4 [68 - 36] (REVERSE SENSE)
LLGGWCGLGWR
>HmenF_UR_24_5 [84 - 25] (REVERSE SENSE)
VSLDGIVSWLMWVGLAVVED
>HmenF_UR_24_6 [61 - 2] (REVERSE SENSE)
VVDVGWVGGSGGLVYVNCMS
>HmenF_UR_25_1 [74 - 103]
ITLLNHYYKP
>HmenF_UR_25_2 [94 - 53] (REVERSE SENSE)
MVVKKGNGVVPGAL
>HmenF_UR_25_3 [75 - 34] (REVERSE SENSE)
MVWCQVPFSVLGCS
>HmenF_UR_26_1 [1 - 30]
MTLKSNSNLT
>HmenF_UR_26_2 [36 - 1] (REVERSE SENSE)
LLGEVSVSFECY
```

- ***Cumberlandia monodonta* F**

```
>CmonF_UR_1_1 [31 - 2] (REVERSE SENSE)
MFVSVCYVLS
>CmonF_UR_3_1 [31 - 2] (REVERSE SENSE)
LFLFVVDEWK
```

>CmonF_UR_4_1 [32 - 91]
IYQVKPYTKKSIYHLNKMLW
>CmonF_UR_4_2 [61 - 138]
INLPLKQNIMMINFFQAASQLAANSK
>CmonF_UR_4_3 [154 - 183]
MNQLMNSSMN
>CmonF_UR_4_4 [138 - 215]
MNKPVNEPTNEQINKLTNNKNTHHLN
>CmonF_UR_4_5 [162 - 124] (REVERSE SENSE)
LVHLPVCLFPVCC
>CmonF_UR_4_6 [196 - 116] (REVERSE SENSE)
LLLVSLLICSLVGSFTGLFISCLLLAD
>CmonF_UR_4_7 [108 - 70] (REVERSE SENSE)
LKKVNYHNILFKW
>CmonF_UR_4_8 [179 - 3] (REVERSE SENSE)
IDLFISWFIYRFVYFLFAASWLAAWKKLIIMMFCLSGKLIFLYKVLPGKLVLLWSGFGC
>CmonF_UR_4_9 [73 - 2] (REVERSE SENSE)
VVNWFFCMSFYLVNWFFFEEVLGA
>CmonF_UR_4_10 [33 - 1] (REVERSE SENSE)
IGSSLKSFWVR
>CmonF_UR_7_1 [6 - 44]
IHSPTQGKANQSP
>CmonF_UR_7_2 [1 - 45]
MQFTHLHKGSQINHQ
>CmonF_UR_7_3 [38 - 3] (REVERSE SENSE)
LICLPLCSWVNC
>CmonF_UR_8_1 [7 - 36]
IQPTKIPLHP
>CmonF_UR_8_2 [33 - 1] (REVERSE SENSE)
VESDFGGLNFV
>CmonF_UR_10_1 [24 - 62]
IQDNLNIYKPHMT
>CmonF_UR_10_2 [43 - 2] (REVERSE SENSE)
MFKLSWMSSFCLLK
>CmonF_UR_17_1 [9 - 71]
MITHQGNTHQDFPKTSTCRQN
>CmonF_UR_17_2 [19 - 75]
IKEMPIKTSQKHQHADKIN
>CmonF_UR_17_3 [71 - 3] (REVERSE SENSE)
ILSACWCFWEVLMGISLMGDYFW
>CmonF_UR_17_4 [75 - 1] (REVERSE SENSE)
INFVGMLMFLGSLDGYFLDGWLFLV
>CmonF_UR_18_1 [2 - 37]
MLHLNPHQHALT
>CmonF_UR_18_2 [38 - 3] (REVERSE SENSE)
MLMRVGGGLDGV
>CmonF_UR_19_1 [1 - 30]
MAFFNHLCKM
>CmonF_UR_19_2 [14 - 52]
IICAKYKYSIICL
>CmonF_UR_19_3 [21 - 53]
VQNMNTELSAL
>CmonF_UR_19_4 [35 - 3] (REVERSE SENSE)
IYILHSWLKKA

```
>CmonF_UR_19_5 [45 - 1] (REVERSE SENSE)
MILYLYFAQMIKESY
>CmonF_UR_20_1 [5 - 43]
MTSTLIDTSFQHN
>CmonF_UR_20_2 [21 - 56]
LTRVFNTINIYQ
>CmonF_UR_20_3 [62 - 21] (REVERSE SENSE)
ILLMDINCVENSCQ
>CmonF_UR_20_4 [49 - 2] (REVERSE SENSE)
MLIVLKTRVNKRWRYY
>CmonF_UR_20_5 [42 - 1] (REVERSE SENSE)
LCWKLVSMSVDVIT
>CmonF_UR_25_1 [2 - 46]
VQQAFTVITLFLLSP
>CmonF_UR_25_2 [44 - 3] (REVERSE SENSE)
VMAGMGWWPWSLVA
>CmonF_UR_26_1 [1 - 33]
MLTQVNFSYTN
>CmonF_UR_28_1 [51 - 13] (REVERSE SENSE)
VWEEVGLSSLWGA
>CmonF_UR_28_2 [38 - 3] (REVERSE SENSE)
LGCLGCGGRSYT
>CmonF_UR_29_1 [46 - 17] (REVERSE SENSE)
MDWMWWGCSG
```

- ***Hyridella menziesii* M**

```
>HmenM_UR_2_1 [30 - 59]
MTTPPLIWAL
>HmenM_UR_2_2 [60 - 31] (REVERSE SENSE)
MSPSLKVELL
>HmenM_UR_2_3 [49 - 2] (REVERSE SENSE)
IKGGVVMGGRGGLACV
>HmenM_UR_3_1 [8 - 64]
IKRTTTTTTNNRPDYSNKQ
>HmenM_UR_3_2 [94 - 177]
ITWPYPSPPYTPPSLPIFFFLLLSSEGK
>HmenM_UR_3_3 [310 - 339]
ISTSLVMQLE
>HmenM_UR_3_4 [386 - 460]
MLTSTHLTYTPPKWGPPLPPCTPLR
>HmenM_UR_3_5 [424 - 597]
VGPPPPPLHTSTLTSSAQGKSLRRLKTHPLGEPAGIPPLQGSPRPPAHCLPAPPPYFM
>HmenM_UR_3_6 [506 - 610]
IRSGSPRESRLYSAAHDRQPIVCQLHHPILYSSPI
>HmenM_UR_3_7 [75 - 638]
INMNTNNYLTMPFPPLHPPIPTYLLLFVTFIGSEVEMPEGPPGSVIMSSLKHSRGLPKEG
PPECLGFSNNPSLKAFLHYLYFTSNTAWMTFKTLSKDEASKPYLYLLLHTLPTHHPSGAP
PSPPAHLYANEKCASEKSTPTKDSSARGARGNPASTGQPTTASPLSASSTTLFYMVVLFN
HLQTETTK
>HmenM_UR_3_8 [613 - 666]
IIYSQSPQNNQPPLKAYA
```

>HmenM_UR_3_9 [638 - 667]
MTNHHSKPTL
>HmenM_UR_3_10 [656 - 621] (REVERSE SENSE)
LSGGWLFCGLCL
>HmenM_UR_3_11 [645 - 613] (REVERSE SENSE)
LVILWSLSVDD
>HmenM_UR_3_12 [652 - 596] (REVERSE SENSE)
VVVGYFVVSVCSWLNSTTM
>HmenM_UR_3_13 [609 - 541] (REVERSE SENSE)
MGLLYKMGWWSWQTMGWRSWAAL
>HmenM_UR_3_14 [586 - 458] (REVERSE SENSE)
VVELADNGLAVVGCPVEAGFPRAPRADESLVGVDFSLAHFSLA
>HmenM_UR_3_15 [450 - 412] (REVERSE SENSE)
VQGGSGGPHLGGV
>HmenM_UR_3_16 [530 - 408] (REVERSE SENSE)
IPAGSPSGWVFSRRSLFPCALLVSVEVCSGGGGGPTWVVCS
>HmenM_UR_3_17 [349 - 317] (REVERSE SENSE)
LKVIQAVLLVK
>HmenM_UR_3_18 [329 - 294] (REVERSE SENSE)
ITSEVEMVEEGL
>HmenM_UR_3_19 [214 - 143] (REVERSE SENSE)
MMTLPGGPSGISTSLPMKVTKSSS
>HmenM_UR_3_20 [284 - 123] (REVERSE SENSE)
IISEAQTLRGPFFSEPPSVFKASHNDPPGGPLWYLYFPSDESNKKKKMGSDGGV
>HmenM_UR_3_21 [133 - 98] (REVERSE SENSE)
MGGCKGGKGMVK
>HmenM_UR_3_22 [95 - 27] (REVERSE SENSE)
IVGVYINLSIIVCLSNLVGCLWL
>HmenM_UR_3_23 [94 - 2] (REVERSE SENSE)
LLVFMLIYLLLFVWVIWSVVCGCSCCAFNYV
>HmenM_UR_3_24 [39 - 1] (REVERSE SENSE)
LFVVVVVVRLITY
>HmenM_UR_4_1 [38 - 3] (REVERSE SENSE)
LVGVVSGSLSRY
>HmenM_UR_6_1 [31 - 2] (REVERSE SENSE)
VCVCVAYLVG
>HmenM_UR_14_1 [12 - 68]
MTQKPVKPQAYTCTPTTQN
>HmenM_UR_14_2 [63 - 31] (REVERSE SENSE)
VLLAYSCKLVV
>HmenM_UR_14_3 [65 - 3] (REVERSE SENSE)
LCCWRTGVSLWFNWFLGYEME
>HmenM_UR_17_1 [10 - 45]
MIQPKFKQEKMN
>HmenM_UR_17_2 [64 - 132]
IPPPQGSPRPPAHCLPAPTPPIF
>HmenM_UR_17_3 [48 - 137]
MSARGNPASTGQPTTASPLSASSHPPYFLA
>HmenM_UR_17_4 [44 - 172]
INKSPRESRLHSAAHDRQPIVCQLPPPLFFSVNLFNNHSQTSY
>HmenM_UR_17_5 [190 - 222]
MTKQKQKYKLT
>HmenM_UR_17_6 [225 - 272]
MMNTNNTQVNPTQHTD

>HmenM_UR_17_7 [266 - 225] (REVERSE SENSE)
VLCSIYLSVVCVYY
>HmenM_UR_17_8 [268 - 221] (REVERSE SENSE)
VCCVGFTWVLFVFIIS
>HmenM_UR_17_9 [237 - 175] (REVERSE SENSE)
LCLLLGKLVLLFLFCYSQPLI
>HmenM_UR_17_10 [215 - 150] (REVERSE SENSE)
LYFCFCFVMGSLLFNSLFGYGY
>HmenM_UR_17_11 [128 - 48] (REVERSE SENSE)
MGGVGAGSQWAGGRGLPCGGGIPAGSY
>HmenM_UR_17_12 [88 - 29] (REVERSE SENSE)
VGCPVEAGFPRALINLSSLV
>HmenM_UR_17_13 [41 - 3] (REVERSE SENSE)
IFSCLNFGWIIEL
>HmenM_UR_17_14 [102 - 1] (REVERSE SENSE)
MGWRSWAALWSRDSRGLLLIYLLLFKFWLDYWVS
>HmenM_UR_20_1 [50 - 3] (REVERSE SENSE)
LSVVNRLGVGFSGAVG
>HmenM_UR_21_1 [1 - 30]
MHSNANSSKM
>HmenM_UR_21_2 [52 - 96]
VSAHTYLNPTTHKAP
>HmenM_UR_21_3 [109 - 174]
MMNHNYTMNMPLPPVNNPPNCQ
>HmenM_UR_21_4 [21 - 176]
IKNMKILKLYGEGPHLPEPHHAQSTLSSSNNKSQLYNKHAPAPGEQPTQLPT
>HmenM_UR_21_5 [167 - 126] (REVERSE SENSE)
LGGLFTGGSGMFIV
>HmenM_UR_21_6 [163 - 50] (REVERSE SENSE)
VGCSPGAGACLLYSCDLLLDELKVLCAWWGSGSCGPSP
>HmenM_UR_21_7 [37 - 2] (REVERSE SENSE)
IFMFLMSLRCCV
>HmenM_UR_21_8 [174 - 1] (REVERSE SENSE)
LAIGWVVHRGQGHVYCMVVIYYWMSLSCFVRGGVQVGVGPHRKVLSFLYFWWVCVAVY
>HmenM_UR_25_1 [3 - 41]
INWTTTLPNPMTT
>HmenM_UR_25_2 [40 - 2] (REVERSE SENSE)
VVMGFGNVVVQLM
>HmenM_UR_26_1 [34 - 2] (REVERSE SENSE)
LLPVVAVFASA
>HmenM_UR_26_2 [78 - 1] (REVERSE SENSE)
IEVWLGSAGSFSLAGCCLLLLYLRLL
>HmenM_UR_28_1 [2 - 40]
IAHNLGVLNSYNQ

- ***Cumberlandia monodonta* M**

>CmonM_UR_1_1 [20 - 55]
IKSTYHKRLTNL
>CmonM_UR_1_2 [12 - 56]
IFQLKAPTTNASPTS
>CmonM_UR_1_3 [37 - 2] (REVERSE SENSE)
VVGAFNWKMQCV

>CmonM_UR_1_4 [30 - 1] (REVERSE SENSE)
VLLIESCSVW
>CmonM_UR_2_1 [4 - 144]
MPPNPGNNITAFPYYYLMVTLFTMKPTMFNKFTLFMKPPKPGAQIKN
>CmonM_UR_2_2 [135 - 67] (REVERSE SENSE)
LSPRFSGLYEKSKFIKYCRFYSE
>CmonM_UR_2_3 [137 - 51] (REVERSE SENSE)
IWAPGLGGFMKSVNLLNIVGFMVNKVTIK
>CmonM_UR_2_4 [67 - 32] (REVERSE SENSE)
MKLLLSNNKGSR
>CmonM_UR_2_5 [48 - 1] (REVERSE SENSE)
MMSEGGNVVSGVWSHF
>CmonM_UR_15_1 [9 - 38]
IPKKTKTMRV
>CmonM_UR_15_2 [31 - 2] (REVERSE SENSE)
IVLVFLGMCY
>CmonM_UR_16_1 [13 - 45]
MGYNKRLILPT
>CmonM_UR_16_2 [78 - 125]
MNTLTYLRLLYVWQLL
>CmonM_UR_16_3 [94 - 147]
IYACFMYDSYSNWFSYSL
>CmonM_UR_16_4 [113 - 214]
MTVTLTDLVTLYSVLHPSLFLYFKLPCKETLNSL
>CmonM_UR_16_5 [147 - 239]
MGFSILLSFYTLNYLAKKPLMGYNKRLILPT
>CmonM_UR_16_6 [272 - 319]
MNTLTYLRLLYVWQLL
>CmonM_UR_16_7 [288 - 341]
IYACFMYDSYSNWFSYSL
>CmonM_UR_16_8 [307 - 378]
MTVTLTDLVTLYSVLHPSLFLYFN
>CmonM_UR_16_9 [341 - 439]
MGFSILLSFYTLTKQLPCQTLPPTSIVSILITS
>CmonM_UR_16_10 [450 - 497]
IIVTKNSWSGQYSRSP
>CmonM_UR_16_11 [466 - 519]
IPDQGNMGAHHNVNNAAM
>CmonM_UR_16_12 [510 - 463] (REVERSE SENSE)
IIYVMVSAYIALISNF
>CmonM_UR_16_13 [518 - 438] (REVERSE SENSE)
MAALFTLWWAPMLPWSGIFSYNNVLFN
>CmonM_UR_16_14 [416 - 381] (REVERSE SENSE)
LSWEEGFGSEVV
>CmonM_UR_16_15 [432 - 370] (REVERSE SENSE)
IKILTIEVGGSVWQGSCLVKV
>CmonM_UR_16_16 [370 - 335] (REVERSE SENSE)
MKSEKDGEPYKE
>CmonM_UR_16_17 [308 - 264] (REVERSE SENSE)
MHKASVDKLEYLWGL
>CmonM_UR_16_18 [277 - 248] (REVERSE SENSE)
IYEDFSLLTH
>CmonM_UR_16_19 [257 - 228] (REVERSE SENSE)
INPLVLCGQD

>CmonM_UR_16_20 [249 - 148] (REVERSE SENSE)
ISFMWASLSACYSLLSVSLQGSLKYKKSEGWSTL
>CmonM_UR_16_21 [176 - 141] (REVERSE SENSE)
MKSEKDGEPYKE
>CmonM_UR_16_22 [114 - 70] (REVERSE SENSE)
MHKASVDKLEYLWGL
>CmonM_UR_16_23 [83 - 54] (REVERSE SENSE)
IYEDFSLLTH
>CmonM_UR_16_24 [63 - 34] (REVERSE SENSE)
INPLVLCGQD
>CmonM_UR_16_25 [55 - 2] (REVERSE SENSE)
ISFMWASLSACYSLLSVS
>CmonM_UR_24_1 [63 - 131]
ITLKKPKEEQMGWQAKAHEHKTD
>CmonM_UR_24_2 [98 - 271]
VTSQSTRTQNRLNHFNTFIDMAPNRTQPLPPTLKSTLMLFHNPLSPRLTFLTKTFRRS
>CmonM_UR_24_3 [271 - 324]
MNSHKRTHTNTNQNCNNK
>CmonM_UR_24_4 [281 - 382]
MNALTQMQTKTAMMSKNKLFSWKNSRIAPKSAFK
>CmonM_UR_24_5 [343 - 396]
VKELTNCPKKSFQMKGTI
>CmonM_UR_24_6 [330 - 458]
MSCFRESTHELPQKELSNKGHHLAQAGPQPHNLPFLSTPLNQP
>CmonM_UR_24_7 [442 - 251] (REVERSE SENSE)
VLKKGSLWGWGPACASWCPLFESSFWGNSWVLSRKQLIFTYYCSFGLYLCECVYVSLWAA
EGFC
>CmonM_UR_24_8 [395 - 168] (REVERSE SENSE)
MVPFIWKLFLGQFVSSFTKTAYFYLLLQFWFVFVWVRLCEFMSGGSFLLSKLSEGLSDCG
SALSLTLEWGGVVGCG
>CmonM_UR_24_9 [178 - 134] (REVERSE SENSE)
LGAVSGYVNKSVEVI
>CmonM_UR_24_10 [122 - 72] (REVERSE SENSE)
VFVCFGLSPYLFFFRFF
>CmonM_UR_24_11 [447 - 67] (REVERSE SENSE)
VVCLSSVDCGVEVPLVLDGALYLKALFGAIREFFHENSLFLLIIAVLVCICVSAFMWVYE
RRKVFVKKVKRGLKGLWKSIKVDFSVGGSGWVRLGAMSMKVLKWFSRFCVRVLWLVTLFV
LLSVFLG
>CmonM_UR_24_12 [124 - 32] (REVERSE SENSE)
LCSCALACHPICSSFGFFSVIVVVVWQLFSA
>CmonM_UR_27_1 [2 - 181]
IYLLFLPNLTLPTPRTKNPPTRDLTKSGFLSLMTPPKFELLTTIFTAFLHSATQRLSLRS
>CmonM_UR_27_2 [91 - 195]
IFDNPPQIRTLNNHLYRLLTLRNPTTKLTLMISPM
>CmonM_UR_27_3 [194 - 138] (REVERSE SENSE)
IGLIMSVSLVVGLRSVSSR
>CmonM_UR_27_4 [132 - 7] (REVERSE SENSE)
MVVKSSNLGGVIKDKNPLLVKSRVGGFFVRGVGSVKFGKKSK
>CmonM_UR_27_5 [128 - 3] (REVERSE SENSE)
LLSVRIWGGLSKMSTHFWLSLGWVGFLCGVWVGLNLVSSANK

RESULTS OF THE SEARCH FOR CONSERVED DOMAINS

Supplementary Table S6 [pages 37-38]. Results for *Hyridella menziesii* M-*orf*. Conserved domains were found only in *H. menziesii* M-*ORF*. No hits were found in *H. menziesii* F-*ORF*, *C. monodonta* F-*ORF* and M-*ORF*, or any other protein putatively encoded by the new ORFs found in the seven newly sequenced mt genomes. Domains were predicted with Batch Web CD-search tool on the CDD database. In the table is indicated the location of the hits on the *H. menziesii* M-*ORF* (From, To), their statistical support (E-value, Bitscore), and information on the hits (Accession, Short Name, Incomplete, Superfamily, Hit type, PSSM-ID, Description). SLAC1 and TDT proteins are transmembrane transporters of various molecules. MAEBL proteins are involved in *Plasmodium* spp. attachment to erythrocytes. MIP-T3 is a microtubule-binding protein. ProP expression regulator and FinO bacterial conjugation repressor are involved in RNA binding. Vfa1 and replication factor C large subunit are AAA-ATPases related proteins. TonB is a bacterial membrane transporter. *H. menziesii* M-*ORF* C-terminus (positions 147-305) is partly encoded by the complex repeat region found in its respective gene (see also Fig. 1 and Supplementary Information 2).

From	To	E-Value	Bitscore	Accession	Short name	Incomplete	Superfamily	Hit type	PSSM-ID	Definition
20	118	7.93E-04	39.04	pfam01757	Acyl_transf_3	NC	cl21495	non-specific	250841	Acyltransferase family
20	118	7.93E-04	39.04	cl21495	Acyl_transf_3 superfamily	NC	-	superfamily	277542	Acyltransferase family
31	117	1.05E-04	41.80	pfam03595	SLAC1	C	cl04176	non-specific	252046	Voltage-dependent anion channel
31	117	1.05E-04	41.80	cl04176	TDT superfamily	C	-	superfamily	275802	Tellurite-resistance/Dicarboxylate Transporter (TDT) family
147	288	3.78E-04	40.13	pfam06459	RR_TM4-6	C	-	multi-dom	253742	Ryanodine Receptor TM 4-6
161	304	7.89E-04	39.74	PTZ00121	PTZ00121	NC	-	multi-dom	173412	MAEBL
166	313	8.12E-06	45.66	pfam10243	MIP-T3	C	-	multi-dom	255846	Microtubule-binding protein MIP-T3
168	315	5.82E-04	40.12	PTZ00121	PTZ00121	N	-	multi-dom	173412	MAEBL
192	310	1.89E-04	41.67	PTZ00121	PTZ00121	NC	-	multi-dom	173412	MAEBL
197	293	1.87E-04	41.03	PRK13808	PRK13808	N	-	multi-dom	172341	adenylate kinase
198	270	1.67E-04	41.44	PRK04195	PRK04195	N	-	multi-dom	235250	replication factor C large subunit
198	292	3.76E-04	40.75	PRK14900	valS	N	-	multi-dom	237855	valyl-tRNA synthetase
201	288	1.62E-04	40.68	PRK04950	PRK04950	N	cl15270	non-specific	235322	ProP expression regulator
201	288	1.62E-04	40.68	cl15270	FinO_conjug_rep superfamily	N	-	superfamily	277488	FinO bacterial conjugation repressor domain
201	300	7.59E-04	39.00	COG0810	TonB	NC	-	multi-dom	223880	Periplasmic protein TonB
203	306	5.01E-06	46.67	PTZ00121	PTZ00121	NC	-	multi-dom	173412	MAEBL
203	304	1.04E-04	42.82	PTZ00121	PTZ00121	NC	-	multi-dom	173412	MAEBL

From	To	E-Value	Bitscore	Accession	Short name	Incomplete	Superfamily	Hit type	PSSM-ID	Definition
203	315	9.31E-04	39.74	PTZ00121	PTZ00121	N	-	multi-dom	173412	MAEBL
204	304	2.58E-04	41.28	PTZ00121	PTZ00121	NC	-	multi-dom	173412	MAEBL
205	313	2.74E-04	40.64	PRK13808	PRK13808	N	-	multi-dom	172341	adenylate kinase
207	308	1.63E-05	44.49	PRK13808	PRK13808	N	-	multi-dom	172341	adenylate kinase
213	305	4.03E-05	42.40	pfam08432	Vfa1	NC	-	multi-dom	254797	AAA-ATPase Vps4-associated protein 1

COMPLETE RESULTS OF `hmmer` ANALYSES ON NEW ORF PROTEINS

The suffix `m` or `a` before a ORF protein name indicates that the protein sequence starts with the amino acid methionine regardless of the start codon, or the corresponding amino acid in presence of alternative, non-methionine, start codons. Both kinds of translation were used for the analyses.

`phmmer` ANALYSIS RESULTS

The "per-target" output table is displayed for successful analyses only (when no results were obtained, no table is shown). Analyses are presented following the order used in the Results section of the main text. Notable hits cited in the paper are highlighted in yellow for reader's convenience.

• vs standard mtDNA PCGs

No results

• vs SwissProt database

No results

• vs TrEMBL database

```
#                                                               --- full sequence ---- --- best 1 domain ---- --- domain number estimation ----
# target name          accession  query name           accession   E-value  score  bias   E-value  score  bias   exp reg clu  ov env dom rep inc description of target
#  ------------------- ---------- -------------------- ---------- --------- ------ ----- --------- ------ -----   --- --- --- --- --- --- --- --- ---------------------
tr|F4ZFE6|F4ZFE6_CUMMO -          CmonF_FORF           -            3.1e-52  185.5  13.3   3.4e-52  185.3  13.3   1.0   1   0   0   1   1   1   1 Female-specific orf protein OS=Cumberlandia monodonta GN=forf PE=4 SV=1
tr|F4ZFE8|F4ZFE8_CUMMO -          CmonF_FORF           -            2.3e-48  173.1  13.3   2.6e-48  173.0  13.3   1.0   1   0   0   1   1   1   1 Female-specific orf protein OS=Cumberlandia monodonta GN=forf PE=4 SV=1
tr|F4ZFH7|F4ZFH7_9BIVA -          CmonF_FORF           -              1e-08   46.5  11.4   1.5e-08   46.0  11.4   1.4   1   1   0   1   1   1   1 Female-specific orf protein OS=Margaritifera marrianae GN=forf PE=4 SV=1
tr|X2CT99|X2CT99_9BIVA -          CmonF_FORF           -              4e-08   44.7  11.8   5.4e-08   44.3  11.8   1.3   1   1   0   1   1   1   1 H open reading frame OS=Dahurinaia dahurica GN=HORF PE=4 SV=1
tr|A0A0B3XTV8|A0A0B3XTV8_PECCC -          CmonM_MORF           -     8.2e-18   75.3  17.5  2.1e+05    3.5   0.0 27.4  34   0   0  34  34  34   0 Hemagglutinin OS=Pectobacterium carotovorum subsp. carotovorum GN=RD01_08090 PE=4 SV=1
tr|J7KVC2|J7KVC2_PECCC         -          CmonM_MORF           -     8.9e-09   46.3   8.8  1.6e+05    3.9   0.0 22.3  36   0   0  36  36  36   1 Putative hemagglutinin/hemolysin-related protein OS=Pectobacterium carotovorum subsp. carotovorum PCC21 GN=PCC21_030980 PE=4 SV=1
tr|A0A0B3XCD8|A0A0B3XCD8_PECCC -          CmonM_MORF           -     4.5e-08   44.1   3.0  1.7e+05    3.8   0.0 15.6  20   0   0  20  20  20   0 Hemagglutinin OS=Pectobacterium carotovorum subsp. carotovorum GN=RD02_13410 PE=4 SV=1
tr|F4ZFF9|F4ZFF9_9BIVA -          HmenF_FORF           -            3.7e-51  181.4  10.0   4.1e-51  181.3  10.0   1.0   1   0   0   1   1   1   1 Female-specific orf protein OS=Echyridella menziesii GN=forf PE=4 SV=1
tr|A0A077TN52|A0A077TN52_PLACH -          HmenM_MORF           -     5.2e-23   92.9  94.9  5.1e-06   37.1  17.5  5.1   1   1   4   5   5   5   5 Fam-a protein OS=Plasmodium chabaudi chabaudi GN=PCHAS_130320 PE=4 SV=1
tr|A0A077TQ82|A0A077TQ82_PLACH -          HmenM_MORF           -     4.7e-09   47.1  59.5  4.6e-05   34.0  43.6  2.5   2   1   0   2   2   2   2 Fam-a protein OS=Plasmodium chabaudi chabaudi GN=PCHAS_130250 PE=4 SV=1
tr|Q4YH81|Q4YH81_PLABA         -          HmenM_MORF           -     5.6e-08   43.6  17.4  6.3e-08   43.4  17.4  1.0   1   0   0   1   1   1   1 Putative uncharacterized protein (Fragment) OS=Plasmodium berghei (strain Anka) GN=PB300859.00.0 PE=4 SV=1
tr|A0A0C1T723|A0A0C1T723_9ACTN -          HmenM_MORF           -     1.2e-06   39.2   6.5  0.00027   31.5   0.8  7.6   1   1   7   9   9   9   3 Large Ala/Glu-rich protein OS=Streptomyces pluripotens GN=LK07_24015 PE=4 SV=1
tr|Q7RLJ1|Q7RLJ1_PLAYO         -          HmenM_MORF           -       2e-06   38.5  28.2  0.00015   32.3  21.1  2.1   1   1   1   2   2   2   2 Uncharacterized protein OS=Plasmodium yoelii yoelii GN=PY02550 PE=4 SV=1
tr|V7PLV2|V7PLV2_9APIC         -          HmenM_MORF           -     2.2e-06   38.3  21.9  2.2e-06   38.3  21.9  2.6   2   0   0   2   2   2   1 Uncharacterized protein OS=Plasmodium yoelii 17X GN=YYC_02202 PE=4 SV=1
tr|Q4X405|Q4X405_PLACH         -          HmenM_MORF           -     9.6e-06   36.2  43.7  2.9e-05   34.6  43.8  1.6   1   1   0   1   1   1   1 Cyclin related protein, putative (Fragment) OS=Plasmodium chabaudi GN=PC101437.00.0 PE=4 SV=1
tr|A0A067L7M7|A0A067L7M7_JATCU -          HmenM_MORF           -     1.3e-05   35.8  14.1  1.4e-05   35.6  14.1  1.0   1   0   0   1   1   1   1 Uncharacterized protein OS=Jatropha curcas GN=JCGZ_02097 PE=4 SV=1
tr|S4R676|S4R676_PETMA         -          HmenM_MORF           -     1.9e-05   35.2   0.0  8.7e+02   10.1   0.0  3.9   1   1   3   4   4   4   4 Uncharacterized protein (Fragment) OS=Petromyzon marinus PE=4 SV=1
tr|Q7RG09|Q7RG09_PLAYO         -          HmenM_MORF           -     2.1e-05   35.1  14.9  2.1e-05   35.1  14.9  3.0   2   1   1   3   3   2   1 Late embryogenesis abundant protein, putative OS=Plasmodium yoelii yoelii GN=PY04542 PE=4 SV=1
tr|A0A078K3T9|A0A078K3T9_9APIC -          HmenM_MORF           -     2.1e-05   35.1  14.9  2.1e-05   35.1  14.9  2.9   2   1   0   2   2   2   1 Uncharacterized protein OS=Plasmodium yoelii GN=PY17X_0216300 PE=4 SV=1
tr|A0A058ZGS7|A0A058ZGS7_9EUKA -          HmenM_MORF           -     0.00011   32.8  18.8      1.2   19.5   6.3  2.5   1   1   0   2   2   2   2 Uncharacterized protein OS=Fonticula alba GN=H696_00714 PE=4 SV=1
tr|Q4XPU8|Q4XPU8_PLACH         -          HmenM_MORF           -     0.00024   31.6  21.1     0.52   20.7   9.5  1.7   1   1   0   1   1   1   1 Cyclin related protein, putative (Fragment) OS=Plasmodium chabaudi GN=PC000395.04.0 PE=4 SV=1
tr|A7T819|A7T819_NEMVE -          a_Nmar_UR_2_3        -            0.00053   29.1   0.2   4.6e+04    6.3   0.0   5.1   1   1   4   5   5   5   5 Predicted protein (Fragment) OS=Nematostella vectensis GN=v1g149618 PE=4 SV=1
tr|F4JGI0|F4JGI0_ARATH -          a_Nmar_UR_3_16       -            1.1e-07   38.6  24.5   2.9e+05    4.5   0.0  13.9  15   0   0  15  15  15   0 Uncharacterized protein OS=Arabidopsis thaliana GN=At4g05612 PE=4 SV=1
tr|F4JGH9|F4JGH9_ARATH -          a_Nmar_UR_3_16       -              1e-05   33.3  27.3   3.3e+05    4.3   0.0  14.4  16   0   0  16  16  16   0 Uncharacterized protein OS=Arabidopsis thaliana GN=At4g05612 PE=4 SV=1
tr|V4AN35|V4AN35_LOTGI -          a_Nmar_UR_3_26       -            4.5e-09   42.9  83.3   3.2e+03    9.8   0.8  18.1   1   1  19  20  20  20  19 Uncharacterized protein (Fragment) OS=Lottia gigantea GN=LOTGIDRAFT_116067 PE=4 SV=1
tr|T1I4N5|T1I4N5_RHOPR -          a_Nmar_UR_3_29       -            2.1e-08   40.5   7.7   2.7e+05    4.3   0.0   9.0   9   0   0   9   9   9   0 Uncharacterized protein OS=Rhodnius prolixus PE=4 SV=1
tr|J3MTQ6|J3MTQ6_ORYBR -          a_Nmar_UR_3_29       -              2e-07   37.9  10.3   4.3e+05    3.8   0.0   9.5   9   0   0   9   9   9   0 Uncharacterized protein OS=Oryza brachyantha GN=OB08G24870 PE=4 SV=1
tr|A7T4Z8|A7T4Z8_NEMVE -          a_Nmar_UR_3_30       -            0.00069   30.4  42.0   3.6e+05    4.0   0.7  18.6   1   1  19  20  20  20   1 Predicted protein OS=Nematostella vectensis GN=v1g146147 PE=4 SV=1
tr|A7S3P9|A7S3P9_NEMVE -          a_Nmar_UR_3_37       -            0.00091   30.5   1.6   7.4e+04    5.9   0.0   6.4   2   2   9  11  11  11   5 Predicted protein OS=Nematostella vectensis GN=v1g103253 PE=4 SV=1
tr|V4B8A3|V4B8A3_LOTGI -          a_Nmar_UR_3_41       -            0.00097   30.2   6.7   1.2e+03   11.3   0.1   4.7   2   1   3   5   5   5   4 Uncharacterized protein (Fragment) OS=Lottia gigantea GN=LOTGIDRAFT_131302 PE=4 SV=1
tr|S4RHK8|S4RHK8_PETMA -          a_Nmar_UR_23_2       -            3.2e-07   37.0  25.1   1.6e+04    7.3   0.1  11.2   9   1   1  10  10  10   6 Uncharacterized protein (Fragment) OS=Petromyzon marinus PE=4 SV=1
tr|T1P979|T1P979_MUSDO -          a_Atra_UR_13_3       -            0.00017   31.3   5.0     4e+05    3.8   0.0   9.2   9   0   0   9   9   9   0 Sperm-tail PG-rich repeat protein OS=Musca domestica PE=2 SV=1
tr|T1PK79|T1PK79_MUSDO -          a_Atra_UR_13_3       -            0.00061   29.7   6.1   7.1e+05    3.0   0.0   9.2   9   0   0   9   9   9   0 Sperm-tail PG-rich repeat protein OS=Musca domestica PE=2 SV=1
tr|R7UYE0|R7UYE0_CAPTE -          a_Atra_UR_13_11      -            8.2e-05   27.2   1.2   4.2e+05    3.3   0.0   7.3   7   0   0   7   7   7   3 Uncharacterized protein (Fragment) OS=Capitella teleta GN=CAPTEDRAFT_55497 PE=4 SV=1
tr|V4A249|V4A249_LOTGI -          a_Atra_UR_14_1       -            7.2e-08   38.6  23.3   8.3e+04    5.3   0.0  12.7   1   1  12  13  13  13   9 Uncharacterized protein (Fragment) OS=Lottia gigantea GN=LOTGIDRAFT_106986 PE=4 SV=1
tr|H2Y9P9|H2Y9P9_CIOSA -          a_Atra_UR_22_1       -            0.00022   29.5  19.6   1.4e+06    2.2   0.0  13.1   1   1  17  19  19  19   0 Uncharacterized protein (Fragment) OS=Ciona savignyi PE=4 SV=1
tr|H2Y9P9|H2Y9P9_CIOSA -          a_Atra_UR_22_6       -            8.3e-09   42.0  30.6   1.8e+05    4.8   0.2  15.6   1   1  19  20  20  20  15 Uncharacterized protein (Fragment) OS=Ciona savignyi PE=4 SV=1
tr|G0UTM7|G0UTM7_TRYCI -          a_Atra_UR_22_14      -            2.1e-12   55.8  55.0   6.6e+05    2.9   0.0  26.8   1   1  28  29  29  29   0 Putative uncharacterized protein OS=Trypanosoma congolense (strain IL3000) GN=TCIL3000_9_1380 PE=4 SV=1
```

tr|B7Q9L7|B7Q9L7_IXOSC	-	a_Atra_UR_22_22	-	0.00091	28.8	18.3	4.2e+05	3.6	0.0	10.3	1	1	10	11	11	11	2	Putative uncharacterized protein (Fragment) OS=Ixodes scapularis GN=IscW_ISCW011693 PE=4 SV=1
tr|M5J7E6|M5J7E6_9LACO	-	a_Mdub_UR_15_7	-	0.00026	30.4	3.5	1.5e+05	4.7	0.0	9.3	10	0	0	10	10	10	2	Uncharacterized protein OS=Lactobacillus saerimneri 30a GN=D271_00543 PE=4 SV=1
tr|M5J5H4|M5J5H4_9LACO	-	a_Mdub_UR_15_7	-	0.00057	29.4	0.5	2e+05	4.3	0.0	9.6	12	0	0	12	12	12	1	N-acetylmuramoyl-L-alanine amidase OS=Lactobacillus saerimneri 30a GN=D271_00538 PE=4 SV=1
tr|A5KLS0|A5KLS0_9FIRM	-	a_Mdub_UR_15_11	-	8.9e-10	48.0	10.9	7.1e+02	12.0	0.1	6.2	5	0	0	5	5	5	4	Repeat protein OS=Ruminococcus torques ATCC 27756 GN=RUMTOR_01174 PE=4 SV=1
tr|E5XHY6|E5XHY6_9FIRM	-	a_Mdub_UR_15_11	-	5.2e-06	36.6	1.3	93	14.6	0.1	3.7	3	0	0	3	3	3	3	Uncharacterized protein (Fragment) OS=Lachnospiraceae bacterium 8_1_57FAA GN=HMPREF1026_01741 PE=4 SV=1
tr|E5XHY5|E5XHY5_9FIRM	-	a_Mdub_UR_15_11	-	7e-06	36.2	4.1	7.5e+02	11.9	0.1	4.2	3	0	0	3	3	3	3	Uncharacterized protein (Fragment) OS=Lachnospiraceae bacterium 8_1_57FAA GN=HMPREF1026_01740 PE=4 SV=1
tr|F4LTD8|F4LTD8_TEPAE	-	a_Mdub_UR_15_11	-	4.8e-05	33.6	1.2	4.8e+03	9.5	0.0	4.9	4	0	0	4	4	4	3	Uncharacterized protein OS=Tepidanaerobacter acetatoxydans (strain DSM 21804 / JCM 16047 / Re1) GN=TEPIRE1_0286 PE=4 SV=1
tr|R5QDA7|R5QDA7_9FIRM	-	a_Mdub_UR_15_11	-	0.0002	31.8	2.6	7.9e+02	11.8	0.1	4.3	3	0	0	3	3	3	2	Uncharacterized protein OS=Ruminococcus torques CAG:61 GN=BN734_00456 PE=4 SV=1
tr|H8XVV7|H8XVV7_FLAIG	-	a_Mdub_UR_15_11	-	0.00071	30.1	33.8	2.9e+04	7.1	0.3	9.0	7	0	0	7	7	7	7	Flavobacterium indicum GPTSA100-9 complete genome OS=Flavobacterium indicum (strain DSM 17447 / CIP 109464 / GPTSA100-9) GN=KQS_10725 PE=4 SV=1
tr|L0MB85|L0MB85_ENTBF	-	a_Mdub_UR_15_12	-	4.9e-08	43.7	0.0	1.7e+05	4.4	0.0	8.5	8	0	0	8	8	8	6	Type 1 secretion C-terminal target domain (VC_A0849 subclass) OS=Enterobacteriaceae bacterium (strain FGI 57) GN=D782_4133 PE=4 SV=1
tr|Q10YX3|Q10YX3_TRIEI	-	a_Mdub_UR_15_17	-	0.00093	30.3	0.2	3.9e+04	6.2	0.0	7.0	7	0	0	7	7	7	3	YD repeat protein OS=Trichodesmium erythraeum (strain IMS101) GN=Tery_3459 PE=4 SV=1
tr|B0CQE1|B0CQE1_LACBS	-	a_Mdub_UR_15_21	-	1.6e-08	42.0	7.0	9.1e+04	5.5	0.0	12.1	2	2	13	15	15	15	0	Predicted protein OS=Laccaria bicolor (strain S238N-H82 / ATCC MYA-4686) GN=LACBIDRAFT_242697 PE=4 SV=1
tr|B0D5G4|B0D5G4_LACBS	-	a_Mdub_UR_15_21	-	2e-07	38.9	2.8	1.6e+05	4.8	0.0	9.3	1	1	10	11	11	11	0	Predicted protein (Fragment) OS=Laccaria bicolor (strain S238N-H82 / ATCC MYA-4686) GN=LACBIDRAFT_161519 PE=4 SV=1
tr|B0DHT5|B0DHT5_LACBS	-	a_Mdub_UR_15_21	-	2.4e-06	35.8	3.3	3.7e+04	6.6	0.0	8.6	2	2	8	10	10	10	2	Predicted protein OS=Laccaria bicolor (strain S238N-H82 / ATCC MYA-4686) GN=LACBIDRAFT_251592 PE=4 SV=1
tr|B0D9C1|B0D9C1_LACBS	-	a_Mdub_UR_15_21	-	4.9e-06	34.9	3.1	1.5e+05	4.8	0.0	8.9	1	1	9	10	10	10	0	Predicted protein (Fragment) OS=Laccaria bicolor (strain S238N-H82 / ATCC MYA-4686) GN=LACBIDRAFT_160710 PE=4 SV=1
tr|B0CT34|B0CT34_LACBS	-	a_Mdub_UR_15_21	-	1.4e-05	33.6	4.9	1e+05	5.3	0.0	9.2	1	1	9	10	10	10	0	Predicted protein OS=Laccaria bicolor (strain S238N-H82 / ATCC MYA-4686) GN=LACBIDRAFT_244568 PE=4 SV=1
tr|B0E477|B0E477_LACBS	-	a_Mdub_UR_15_21	-	4.2e-05	32.2	1.1	2.1e+04	7.3	0.0	6.7	2	2	5	7	7	7	1	Predicted protein (Fragment) OS=Laccaria bicolor (strain S238N-H82 / ATCC MYA-4686) GN=LACBIDRAFT_164190 PE=4 SV=1
tr|B0E4G9|B0E4G9_LACBS	-	a_Mdub_UR_15_21	-	6.6e-05	31.7	1.8	1.6e+05	4.8	0.0	7.4	2	2	6	8	8	8	0	Predicted protein (Fragment) OS=Laccaria bicolor (strain S238N-H82 / ATCC MYA-4686) GN=LACBIDRAFT_163230 PE=4 SV=1
tr|B0DC92|B0DC92_LACBS	-	a_Mdub_UR_15_21	-	0.00026	29.9	3.1	3.4e+05	3.8	0.0	10.4	4	4	9	13	13	13	0	Predicted protein OS=Laccaria bicolor (strain S238N-H82 / ATCC MYA-4686) GN=LACBIDRAFT_235562 PE=4 SV=1
tr|B0DSH6|B0DSH6_LACBS	-	a_Mdub_UR_15_21	-	0.00079	28.6	1.2	3.1e+04	6.8	0.0	7.8	2	2	8	10	10	10	1	Predicted protein (Fragment) OS=Laccaria bicolor (strain S238N-H82 / ATCC MYA-4686) GN=LACBIDRAFT_146775 PE=4 SV=1
tr|A7S2A4|A7S2A4_NEMVE	-	a_Mdub_UR_15_22	-	0.00017	28.0	8.7	2.2e+06	1.4	0.0	11.6	13	0	0	13	13	13	0	Predicted protein OS=Nematostella vectensis GN=v1g242342 PE=4 SV=1
tr|C3ZQY2|C3ZQY2_BRAFL	-	a_Mdub_UR_15_24	-	2.3e-10	50.6	20.0	3.9e+03	9.7	0.0	9.6	2	2	5	9	9	9	9	Putative uncharacterized protein OS=Branchiostoma floridae GN=BRAFLDRAFT_87485 PE=4 SV=1
tr|L8ILL1|L8ILL1_9CETA	-	a_HmenF_UR_1_1	-	0.00071	27.3	23.5	8.4e+04	5.6	0.2	9.4	9	0	0	9	9	9	8	Uncharacterized protein OS=Bos mutus GN=M91_06813 PE=4 SV=1
tr|C3ZQ11|C3ZQ11_BRAFL	-	a_HmenF_UR_26_2	-	8.4e-07	33.1	12.7	3.3e+05	3.4	0.0	10.7	11	0	0	11	11	11	8	Putative uncharacterized protein (Fragment) OS=Branchiostoma floridae GN=BRAFLDRAFT_217974 PE=4 SV=1
tr|A7SVX3|A7SVX3_NEMVE	-	a_CmonF_UR_10_1	-	1.6e-05	30.5	4.8	8.5e+03	7.8	0.0	5.2	1	1	4	5	5	5	5	Predicted protein (Fragment) OS=Nematostella vectensis GN=v1g63670 PE=4 SV=1
tr|V4BNX3|V4BNX3_LOTGI	-	a_CmonF_UR_10_1	-	9.5e-05	28.5	2.3	9.3e+04	5.1	0.0	6.0	6	0	0	6	6	6	1	Uncharacterized protein (Fragment) OS=Lottia gigantea GN=LOTGIDRAFT_78339 PE=4 SV=1
tr|A7SSW3|A7SSW3_NEMVE	-	a_CmonF_UR_10_1	-	0.00053	26.6	14.4	9.6e+05	2.4	0.0	11.7	11	0	0	11	11	11	0	Predicted protein OS=Nematostella vectensis GN=v1g216953 PE=4 SV=1
tr|A2HU56|A2HU56_TRIVA	-	a_CmonF_UR_17_1	-	1.7e-05	33.0	15.2	2.1e+05	4.4	0.0	9.1	9	0	0	9	9	9	0	Putative uncharacterized protein (Fragment) OS=Trichomonas vaginalis GN=TVAG_593970 PE=4 SV=1
tr|A0A033UL83|A0A033UL83_STAAU	-	a_CmonF_UR_17_1	-	3.4e-05	32.1	14.9	2.6e+05	4.1	0.0	9.4	2	1	8	10	10	10	0	Uncharacterized protein OS=Staphylococcus aureus C0673 GN=V070_02125 PE=4 SV=1
tr|A2HFQ8|A2HFQ8_TRIVA	-	a_CmonF_UR_17_1	-	9.1e-05	30.9	9.5	1.7e+05	4.6	0.0	7.2	7	0	0	7	7	7	0	Putative uncharacterized protein OS=Trichomonas vaginalis GN=TVAG_141710 PE=4 SV=1
tr|A2H7W9|A2H7W9_TRIVA	-	a_CmonF_UR_17_1	-	0.00036	29.2	10.0	1.6e+05	4.7	0.0	7.1	7	0	0	7	7	7	0	Putative uncharacterized protein (Fragment) OS=Trichomonas vaginalis GN=TVAG_420670 PE=4 SV=1
tr|A0A010V2T1|A0A010V2T1_ACIBA	-	a_CmonF_UR_19_1	-	5e-06	30.1	28.5	1.1e+05	4.7	0.1	11.5	11	0	0	11	11	11	0	Uncharacterized protein OS=Acinetobacter baumannii 121738 GN=J717_1250 PE=4 SV=1
tr|A0A0A8TTV8|A0A0A8TTV8_ACIBZ	-	a_CmonF_UR_19_1	-	2.3e-05	28.5	23.9	5.7e+04	5.4	0.0	9.5	9	0	0	9	9	9	2	Fragment of Orfc273-4 from Vibrio metschnikovii OS=Acinetobacter bereziniae PE=4 SV=1
tr|D0C9W2|D0C9W2_ACIBA	-	a_CmonF_UR_19_1	-	8.6e-05	27.1	23.6	8.4e+04	5.0	0.1	9.6	9	1	0	9	9	9	0	Uncharacterized protein OS=Acinetobacter baumannii ATCC 19606 = CIP 70.34 GN=HMPREF0010_01425 PE=4 SV=1
tr|M2YDJ0|M2YDJ0_ACIBA	-	a_CmonF_UR_19_1	-	8.6e-05	27.1	23.6	8.4e+04	5.0	0.1	9.6	9	1	0	9	9	9	0	Uncharacterized protein OS=Acinetobacter baumannii MSP4-16 GN=G347_03045 PE=4 SV=1
tr|A0A010V2R5|A0A010V2R5_ACIBA	-	a_CmonF_UR_19_1	-	9.4e-05	27.0	17.8	4.1e+04	5.8	0.1	7.7	7	0	0	7	7	7	2	Uncharacterized protein OS=Acinetobacter baumannii 121738 GN=J717_1235 PE=4 SV=1
tr|L9NZF6|L9NZF6_ACIBA	-	a_CmonF_UR_19_1	-	0.00018	26.3	8.4	2.7e+04	6.2	0.1	6.4	6	0	0	6	6	6	1	Uncharacterized protein OS=Acinetobacter baumannii Naval-57 GN=ACINNAV57_0947 PE=4 SV=1
tr|N9JS27|N9JS27_ACIBA	-	a_CmonF_UR_19_1	-	0.0004	25.4	21.1	5.1e+04	5.5	0.1	8.6	8	1	0	8	8	8	1	Uncharacterized protein OS=Acinetobacter baumannii ATCC 19606 = CIP 70.34 GN=F911_03042 PE=4 SV=1
tr|M2ZH54|M2ZH54_ACIBA	-	a_CmonF_UR_19_1	-	0.00085	24.6	21.4	6.9e+04	5.2	0.0	8.3	8	0	0	8	8	8	0	Uncharacterized protein OS=Acinetobacter baumannii MSP4-16 GN=G347_03040 PE=4 SV=1
tr|A9THW6|A9THW6_PHYPA	-	a_CmonF_UR_20_5	-	0.00033	27.4	5.5	1.9e+05	4.2	0.0	8.2	7	0	0	7	7	7	3	Predicted protein (Fragment) OS=Physcomitrella patens subsp. patens GN=PHYPADRAFT_145827 PE=4 SV=1
tr|H9GSU9|H9GSU9_ANOCA	-	a_HmenM_UR_3_5	-	5.2e-07	41.0	28.2	3.5e+04	6.8	0.1	10.9	1	1	12	13	13	13	11	Uncharacterized protein (Fragment) OS=Anolis carolinensis PE=4 SV=1
tr|H9GUI7|H9GUI7_ANOCA	-	a_HmenM_UR_3_5	-	0.00019	32.9	32.9	1.2e+04	8.2	0.5	8.9	1	1	11	12	12	12	11	Uncharacterized protein (Fragment) OS=Anolis carolinensis PE=4 SV=1
tr|A0A0E0B8G3|A0A0E0B8G3_9ORYZ	-	a_HmenM_UR_3_16	-	1.2e-82	274.5	414.4	4.1e+05	3.7	0.1	125.1	128	0	0	128	128	128	127	Uncharacterized protein (Fragment) OS=Oryza glumipatula PE=4 SV=1
tr|V4C094|V4C094_LOTGI	-	a_HmenM_UR_20_1	-	3.8e-09	41.4	33.8	4.4e+05	3.4	0.1	20.2	3	2	17	23	23	23	1	Uncharacterized protein (Fragment) OS=Lottia gigantea GN=LOTGIDRAFT_117769 PE=4 SV=1
tr|T0S4S0|T0S4S0_9STRA	-	a_HmenM_UR_21_4	-	0.00023	32.5	0.9	4e+05	3.7	0.0	11.5	15	0	0	15	15	15	0	Uncharacterized protein OS=Saprolegnia diclina VS20 GN=SDRG_02703 PE=4 SV=1
tr|T0SBD4|T0SBD4_9STRA	-	a_HmenM_UR_21_4	-	0.00026	32.3	1.1	4e+05	3.7	0.0	11.5	15	0	0	15	15	15	0	Uncharacterized protein OS=Saprolegnia diclina VS20 GN=SDRG_02703 PE=4 SV=1
tr|A7S409|A7S409_NEMVE	-	a_CmonM_UR_1_1	-	0.00027	26.0	16.0	1.8e+05	4.1	0.1	9.1	2	2	7	9	9	9	9	Predicted protein (Fragment) OS=Nematostella vectensis GN=v1g23937 PE=4 SV=1
tr|F0X6V5|F0X6V5_GROCL	-	a_CmonM_UR_16_17	-	2.9e-15	58.3	14.8	5.7e+04	5.7	0.0	12.2	12	0	0	12	12	12	9	Putative uncharacterized protein OS=Grosmannia clavigera (strain kw1407 / UAMH 11150) GN=CMQ_6895 PE=4 SV=1
tr|B8KPF0|B8KPF0_9GAMM	-	a_CmonM_UR_16_17	-	0.00027	28.4	9.9	7.8e+05	2.6	0.0	10.0	11	1	0	11	11	11	0	Putative uncharacterized protein OS=gamma proteobacterium NOR5-3 GN=NOR53_192 PE=4 SV=1
tr|A4A5P6|A4A5P6_9GAMM	-	a_CmonM_UR_16_17	-	0.00062	27.4	3.6	1.3e+06	2.0	0.0	8.9	9	0	0	9	9	9	0	Uncharacterized protein OS=Congregibacter litoralis KT71 GN=KT71_00160 PE=4 SV=1
tr|F0X6V5|F0X6V5_GROCL	-	a_CmonM_UR_16_22	-	2.9e-15	58.3	14.8	5.7e+04	5.7	0.0	12.2	12	0	0	12	12	12	9	Putative uncharacterized protein OS=Grosmannia clavigera (strain kw1407 / UAMH 11150) GN=CMQ_6895 PE=4 SV=1
tr|B8KPF0|B8KPF0_9GAMM	-	a_CmonM_UR_16_22	-	0.00027	28.4	9.9	7.8e+05	2.6	0.0	10.0	11	1	0	11	11	11	0	Putative uncharacterized protein OS=gamma proteobacterium NOR5-3 GN=NOR53_192 PE=4 SV=1
tr|A4A5P6|A4A5P6_9GAMM	-	a_CmonM_UR_16_22	-	0.00062	27.4	3.6	1.3e+06	2.0	0.0	8.9	9	0	0	9	9	9	0	Uncharacterized protein OS=Congregibacter litoralis KT71 GN=KT71_00160 PE=4 SV=1
tr|F6RIH0|F6RIH0_MACMU	-	a_CmonM_UR_24_1	-	3.5e-05	32.9	25.6	2.5e+05	4.5	0.0	11.3	11	0	0	11	11	11	7	Uncharacterized protein (Fragment) OS=Macaca mulatta PE=4 SV=1
tr|A0A093J0F2|A0A093J0F2_EURHL	-	a_CmonM_UR_24_3	-	1.9e-09	42.9	28.8	2.9e+04	6.9	0.1	11.1	11	0	0	11	11	11	0	Zinc finger protein 41 (Fragment) OS=Eurypyga helias GN=N326_13237 PE=4 SV=1
tr|J9K4V2|J9K4V2_ACYPI	-	a_CmonM_UR_24_3	-	7.7e-06	33.1	31.1	5.7e+05	3.3	0.0	14.3	14	0	0	14	14	14	0	Uncharacterized protein (Fragment) OS=Acyrthosiphon pisum GN=LOC100166945 PE=4 SV=2
tr|A0A075M1N1|A0A075M1N1_PANTR	-	a_CmonM_UR_24_3	-	8.3e-06	33.0	32.8	8.7e+05	2.8	0.0	15.5	16	0	0	16	16	16	0	PRDM9 zinc finger domain protein (Fragment) OS=Pan troglodytes GN=PRDM9 PE=4 SV=1
tr|A0A075M1T5|A0A075M1T5_PANTR	-	a_CmonM_UR_24_3	-	2.1e-05	31.9	35.4	9.5e+05	2.7	0.0	16.3	17	0	0	17	17	17	0	PRDM9 zinc finger domain protein (Fragment) OS=Pan troglodytes GN=PRDM9 PE=4 SV=1
tr|I6W7U9|I6W7U9_PANTR	-	a_CmonM_UR_24_3	-	2.3e-05	31.8	35.4	9.5e+05	2.7	0.0	16.3	17	0	0	17	17	17	0	PR domain zinc finger protein 9 (Fragment) OS=Pan troglodytes schweinfurthii GN=PRDM9 PE=4 SV=1
tr|H9GV96|H9GV96_ANOCA	-	a_CmonM_UR_24_3	-	3e-05	31.5	43.9	1.6e+05	4.9	0.1	13.9	14	0	0	14	14	13	0	Uncharacterized protein (Fragment) OS=Anolis carolinensis PE=4 SV=1
tr|R4GC05|R4GC05_ANOCA	-	a_CmonM_UR_24_3	-	3e-05	31.4	52.8	1e+05	5.4	0.1	15.3	15	0	0	15	15	15	0	Uncharacterized protein OS=Anolis carolinensis PE=4 SV=1
tr|L7MZY5|L7MZY5_ANOCA	-	a_CmonM_UR_24_3	-	6.2e-05	30.6	39.6	3.5e+05	3.9	0.1	14.8	15	0	0	15	15	14	0	Uncharacterized protein OS=Anolis carolinensis PE=4 SV=2
tr|A0A075M0D2|A0A075M0D2_PANTR	-	a_CmonM_UR_24_3	-	6.2e-05	30.6	28.1	7.3e+05	3.0	0.0	13.6	14	0	0	14	14	14	0	PRDM9 zinc finger domain protein (Fragment) OS=Pan troglodytes GN=PRDM9 PE=4 SV=1
tr|H9GVJ5|H9GVJ5_ANOCA	-	a_CmonM_UR_24_3	-	6.4e-05	30.6	44.2	1.6e+05	4.8	0.1	14.0	14	0	0	14	14	13	0	Uncharacterized protein (Fragment) OS=Anolis carolinensis PE=4 SV=1
tr|A0A075M1F7|A0A075M1F7_PANTR	-	a_CmonM_UR_24_3	-	6.5e-05	30.5	41.0	7.5e+05	3.0	0.0	17.9	19	0	0	19	19	19	0	PRDM9 zinc finger domain protein (Fragment) OS=Pan troglodytes GN=PRDM9 PE=4 SV=1
tr|A0A075M1P7|A0A075M1P7_PANTR	-	a_CmonM_UR_24_3	-	7e-05	30.4	33.2	8.7e+05	2.8	0.0	15.3	16	0	0	16	16	16	0	PRDM9 zinc finger domain protein (Fragment) OS=Pan troglodytes GN=PRDM9 PE=4 SV=1
tr|J9K2Z9|J9K2Z9_ACYPI	-	a_CmonM_UR_24_3	-	7.4e-05	30.4	33.4	3.6e+05	3.9	0.0	15.4	16	0	0	16	16	15	0	Uncharacterized protein OS=Acyrthosiphon pisum PE=4 SV=2
tr|A0A075M5Z3|A0A075M5Z3_PANTR	-	a_CmonM_UR_24_3	-	7.6e-05	30.3	40.8	1.1e+06	2.6	0.0	17.9	19	0	0	19	19	19	0	PRDM9 zinc finger domain protein (Fragment) OS=Pan troglodytes GN=PRDM9 PE=4 SV=1
tr|A0A075M5Y8|A0A075M5Y8_PANTR	-	a_CmonM_UR_24_3	-	7.8e-05	30.3	40.8	1.1e+06	2.6	0.0	17.9	19	0	0	19	19	19	0	PRDM9 zinc finger domain protein (Fragment) OS=Pan troglodytes GN=PRDM9 PE=4 SV=1
tr|A0A075M1L8|A0A075M1L8_PANTR	-	a_CmonM_UR_24_3	-	8.6e-05	30.2	35.7	9.5e+05	2.7	0.0	16.2	17	0	0	17	17	17	0	PRDM9 zinc finger domain protein (Fragment) OS=Pan troglodytes GN=PRDM9 PE=4 SV=1
tr|I6VCR1|I6VCR1_PANTR	-	a_CmonM_UR_24_3	-	9.3e-05	30.1	35.7	9.5e+05	2.7	0.0	16.2	17	0	0	17	17	17	0	PR domain zinc finger protein 9 (Fragment) OS=Pan troglodytes verus GN=PRDM9 PE=4 SV=1
tr|A0A075M5Z8|A0A075M5Z8_PANTR	-	a_CmonM_UR_24_3	-	9.6e-05	30.1	38.6	7e+05	3.1	0.0	17.0	18	0	0	18	18	18	0	PRDM9 zinc finger domain protein (Fragment) OS=Pan troglodytes GN=PRDM9 PE=4 SV=1
tr|H9G9B5|H9G9B5_ANOCA	-	a_CmonM_UR_24_3	-	0.00011	29.9	50.9	2e+05	4.6	0.1	15.4	15	0	0	15	15	15	0	Uncharacterized protein (Fragment) OS=Anolis carolinensis PE=4 SV=1
tr|L7MZG7|L7MZG7_ANOCA	-	a_CmonM_UR_24_3	-	0.00013	29.7	30.0	2.7e+05	4.2	0.1	12.3	12	0	0	12	12	12	0	Uncharacterized protein OS=Anolis carolinensis PE=4 SV=1
tr|J9JY95|J9JY95_ACYPI	-	a_CmonM_UR_24_3	-	0.00014	29.6	9.0	1.2e+05	5.2	0.1	7.9	7	0	0	7	7	7	0	Uncharacterized protein OS=Acyrthosiphon pisum PE=4 SV=2
tr|A0A075M1K8|A0A075M1K8_PAPCY	-	a_CmonM_UR_24_3	-	0.00016	29.5	20.7	5.3e+05	3.4	0.0	11.1	11	0	0	11	11	11	0	PRDM9 zinc finger domain protein (Fragment) OS=Papio cynocephalus GN=PRDM9 PE=4 SV=1
tr|A0A075M0B2|A0A075M0B2_PANTR	-	a_CmonM_UR_24_3	-	0.00016	29.4	25.1	6.6e+05	3.2	0.0	12.7	13	0	0	13	13	13	0	PRDM9 zinc finger domain protein (Fragment) OS=Pan troglodytes GN=PRDM9 PE=4 SV=1
tr|X1XDB1|X1XDB1_ACYPI	-	a_CmonM_UR_24_3	-	0.00017	29.4	35.6	2.8e+05	4.2	0.0	13.7	14	0	0	14	14	14	0	Uncharacterized protein OS=Acyrthosiphon pisum PE=4 SV=1
tr|A0A075M0C6|A0A075M0C6_PANTR	-	a_CmonM_UR_24_3	-	0.00018	29.3	35.9	9.5e+05	2.7	0.0	16.1	17	0	0	17	17	17	0	PRDM9 zinc finger domain protein (Fragment) OS=Pan troglodytes GN=PRDM9 PE=4 SV=1
tr|A0A075M5Y4|A0A075M5Y4_PANTR	-	a_CmonM_UR_24_3	-	0.00018	29.3	35.9	9.5e+05	2.7	0.0	16.1	17	0	0	17	17	17	0	PRDM9 zinc finger domain protein (Fragment) OS=Pan troglodytes GN=PRDM9 PE=4 SV=1
tr|A0A075M1G1|A0A075M1G1_PANTR	-	a_CmonM_UR_24_3	-	0.00018	29.3	35.9	9.5e+05	2.7	0.0	16.1	17	0	0	17	17	17	0	PRDM9 zinc finger domain protein (Fragment) OS=Pan troglodytes GN=PRDM9 PE=4 SV=1
tr|A0A075M1T0|A0A075M1T0_PANTR	-	a_CmonM_UR_24_3	-	0.00018	29.3	36.3	6.8e+05	3.1	0.0	16.1	17	0	0	17	17	17	0	PRDM9 zinc finger domain protein (Fragment) OS=Pan troglodytes GN=PRDM9 PE=4 SV=1
tr|A0A075M0A5|A0A075M0A5_PANTR	-	a_CmonM_UR_24_3	-	0.00019	29.2	36.3	6.8e+05	3.1	0.0	16.1	17	0	0	17	17	17	0	PRDM9 zinc finger domain protein (Fragment) OS=Pan troglodytes GN=PRDM9 PE=4 SV=1
tr|A0A075M1H5|A0A075M1H5_PANTR	-	a_CmonM_UR_24_3	-	0.00024	29.0	28.3	7.3e+05	3.0	0.0	13.6	14	0	0	14	14	14	0	PRDM9 zinc finger domain protein (Fragment) OS=Pan troglodytes GN=PRDM9 PE=4 SV=1
tr|A0A075M621|A0A075M621_PANTR	-	a_CmonM_UR_24_3	-	0.00025	28.9	28.3	7.3e+05	3.0	0.0	13.5	14	0	0	14	14	14	0	PRDM9 zinc finger domain protein (Fragment) OS=Pan troglodytes GN=PRDM9 PE=4 SV=1
tr|J9JVC4|J9JVC4_ACYPI	-	a_CmonM_UR_24_3	-	0.00026	28.9	17.2	5.6e+04	6.1	0.0	9.9	10	0	0	10	10	8	0	Uncharacterized protein OS=Acyrthosiphon pisum PE=4 SV=1
tr|A0A075M615|A0A075M615_PANTR	-	a_CmonM_UR_24_3	-	0.00026	28.9	28.3	7.3e+05	3.0	0.0	13.5	14	0	0	14	14	14	0	PRDM9 zinc finger domain protein (Fragment) OS=Pan troglodytes GN=PRDM9 PE=4 SV=1
tr|I6W5C4|I6W5C4_PANTR	-	a_CmonM_UR_24_3	-	0.00026	28.9	28.3	7.4e+05	3.0	0.0	13.5	14	0	0	14	14	14	0	PR domain zinc finger protein 9 (Fragment) OS=Pan troglodytes schweinfurthii GN=PRDM9 PE=4 SV=1
tr|A0A075M1Q1|A0A075M1Q1_PANTR	-	a_CmonM_UR_24_3	-	0.00027	28.8	28.4	6.4e+05	3.2	0.0	13.5	14	0	0	14	14	14	0	PRDM9 zinc finger domain protein (Fragment) OS=Pan troglodytes GN=PRDM9 PE=4 SV=1
tr|A0A075M1F2|A0A075M1F2_PANTR	-	a_CmonM_UR_24_3	-	0.00031	28.7	33.8	6.2e+05	3.2	0.0	15.3	16	0	0	16	16	16	0	PRDM9 zinc finger domain protein (Fragment) OS=Pan troglodytes GN=PRDM9 PE=4 SV=1
tr|I6VVM1|I6VVM1_PANTR	-	a_CmonM_UR_24_3	-	0.00034	28.6	33.8	6.3e+05	3.2	0.0	15.3	16	0	0	16	16	16	0	PR domain zinc finger protein 9 (Fragment) OS=Pan troglodytes troglodytes GN=PRDM9 PE=4 SV=1
tr|I6VCQ8|I6VCQ8_PANTR	-	a_CmonM_UR_24_3	-	0.00041	28.3	23.1	4.3e+05	3.7	0.0	11.8	12	0	0	12	12	12	0	PR domain zinc finger protein 9 (Fragment) OS=Pan troglodytes troglodytes GN=PRDM9 PE=4 SV=1
tr|H9GQ53|H9GQ53_ANOCA	-	a_CmonM_UR_24_3	-	0.00042	28.3	29.9	2.6e+05	4.3	0.1	12.2	12	0	0	12	12	12	0	Uncharacterized protein (Fragment) OS=Anolis carolinensis PE=4 SV=1
tr|A0A075M1M8|A0A075M1M8_PANTR	-	a_CmonM_UR_24_3	-	0.00046	28.2	38.6	1e+06	2.6	0.0	16.9	18	0	0	18	18	18	0	PRDM9 zinc finger domain protein (Fragment) OS=Pan troglodytes GN=PRDM9 PE=4 SV=1
tr|X1XPN5|X1XPN5_ACYPI	-	a_CmonM_UR_24_3	-	0.00052	28.0	22.0	6.3e+04	5.9	0.1	9.6	9	1	0	9	9	8	0	Uncharacterized protein OS=Acyrthosiphon pisum PE=4 SV=1
tr|G1KXE3|G1KXE3_ANOCA	-	a_CmonM_UR_24_3	-	0.00057	28.0	60.4	9.4e+04	5.5	0.1	19.1	20	0	0	20	20	19	0	Uncharacterized protein (Fragment) OS=Anolis carolinensis PE=4 SV=1
tr|H9GTR3|H9GTR3_ANOCA	-	a_CmonM_UR_24_3	-	0.0006	27.9	39.4	1.8e+05	4.7	0.1	14.7	15	0	0	15	15	14	0	Uncharacterized protein OS=Anolis carolinensis PE=4 SV=1
tr|A0A075M1M3|A0A075M1M3_PANTR	-	a_CmonM_UR_24_3	-	0.00061	27.9	41.3	1.1e+06	2.6	0.0	17.7	19	0	0	19	19	19	0	PRDM9 zinc finger domain protein (Fragment) OS=Pan troglodytes GN=PRDM9 PE=4 SV=1
tr|X1XP18|X1XP18_ACYPI	-	a_CmonM_UR_24_3	-	0.00069	27.7	30.1	2.4e+05	4.3	0.0	13.2	13	0	0	13	13	13	0	Uncharacterized protein OS=Acyrthosiphon pisum GN=pdp1 PE=4 SV=1
tr|H2R5H8|H2R5H8_PANTR	-	a_CmonM_UR_24_3	-	0.00082	27.5	41.7	9e+05	2.8	0.1	18.1	19	0	0	19	19	19	0	Uncharacterized protein (Fragment) OS=Pan troglodytes PE=4 SV=1
tr|A0A075M0H4|A0A075M0H4_CALJA	-	a_CmonM_UR_24_3	-	0.00087	27.4	19.0	4.7e+05	3.6	0.0	10.1	10	0	0	10	10	10	0	PRDM9 zinc finger domain protein (Fragment) OS=Callithrix jacchus GN=PRDM9 PE=4 SV=1
tr|A0A075M1G6|A0A075M1G6_PANTR	-	a_CmonM_UR_24_3	-	0.00092	27.4	38.8	1e+06	2.6	0.0	16.9	18	0	0	18	18	18	0	PRDM9 zinc finger domain protein (Fragment) OS=Pan troglodytes GN=PRDM9 PE=4 SV=1
tr|A0A075M0B9|A0A075M0B9_PANTR	-	a_CmonM_UR_24_3	-	0.00092	27.4	38.8	1e+06	2.6	0.0	16.9	18	0	0	18	18	18	0	PRDM9 zinc finger domain protein (Fragment) OS=Pan troglodytes GN=PRDM9 PE=4 SV=1
tr|T1HAU4|T1HAU4_RHOPR	-	a_CmonM_UR_24_5	-	0.00061	27.5	6.8	5.6e+05	2.9	0.0	10.9	12	0	0	12	12	12	0	Uncharacterized protein OS=Rhodnius prolixus PE=4 SV=1
tr|A7S1D4|A7S1D4_NEMVE	-	a_CmonM_UR_27_2	-	4.4e-10	48.5	46.9	1.7e+05	4.6	0.2	20.7	1	1	28	30	30	30	0	Predicted protein (Fragment) OS=Nematostella vectensis GN=v1g100921 PE=4 SV=1
tr|A7T938|A7T938_NEMVE	-	a_CmonM_UR_27_2	-	6.9e-10	47.9	29.7	1.2e+05	5.1	0.2	15.5	1	1	24	25	25	25	2	Predicted protein (Fragment) OS=Nematostella vectensis GN=v1g224033 PE=4 SV=1
tr|G3X2E1|G3X2E1_SARHA	-	a_CmonM_UR_27_2	-	3.3e-05	33.8	77.1	1.3e+04	8.0	1.3	14.2	1	1	19	20	20	20	17	Uncharacterized protein (Fragment) OS=Sarcophilus harrisii PE=4 SV=1
tr|A7T819|A7T819_NEMVE	-	m_Nmar_UR_2_3	-	0.0004	29.4	0.2	4.2e+04	6.4	0.0	5.1	1	1	4	5	5	5	5	Predicted protein (Fragment) OS=Nematostella vectensis GN=v1g149618 PE=4 SV=1
tr|F4JGI0|F4JGI0_ARATH	-	m_Nmar_UR_3_16	-	1.1e-07	38.6	24.5	2.9e+05	4.5	0.0	13.9	15	0	0	15	15	15	0	Uncharacterized protein OS=Arabidopsis thaliana GN=At4g05612 PE=4 SV=1

tr|F4JGH9|F4JGH9_ARATH	-	m_Nmar_UR_3_16	-	1e-05	33.3	27.3	3.3e+05	4.3	0.0	14.4	16	0	0	16	16	16	0	Uncharacterized protein OS=Arabidopsis thaliana GN=At4g05612 PE=4 SV=1
tr|V4AN35|V4AN35_LOTGI	-	m_Nmar_UR_3_26	-	4.5e-09	42.9	83.3	3.2e+03	9.8	0.8	18.1	1	1	19	20	20	20	19	Uncharacterized protein (Fragment) OS=Lottia gigantea GN=LOTGIDRAFT_116067 PE=4 SV=1
tr|J3MTQ6|J3MTQ6_ORYBR	-	m_Nmar_UR_3_29	-	1.2e-09	43.9	12.0	2.1e+05	4.6	0.0	9.6	9	0	0	9	9	9	9	Uncharacterized protein OS=Oryza brachyantha GN=OB08G24870 PE=4 SV=1
tr|T1I4N5|T1I4N5_RHOPR	-	m_Nmar_UR_3_29	-	2.3e-07	37.5	6.4	4.1e+05	3.8	0.0	8.9	9	0	0	9	9	9	0	Uncharacterized protein OS=Rhodnius prolixus PE=4 SV=1
tr|A7T4Z8|A7T4Z8_NEMVE	-	m_Nmar_UR_3_30	-	0.00069	30.4	42.0	3.6e+05	4.0	0.7	18.6	1	1	19	20	20	20	1	Predicted protein OS=Nematostella vectensis GN=v1g146147 PE=4 SV=1
tr|A7S3P9|A7S3P9_NEMVE	-	m_Nmar_UR_3_37	-	0.00091	30.5	1.6	7.4e+04	5.9	0.0	6.4	2	2	9	11	11	11	5	Predicted protein OS=Nematostella vectensis GN=v1g103253 PE=4 SV=1
tr|V4B8A3|V4B8A3_LOTGI	-	m_Nmar_UR_3_41	-	0.00096	30.2	6.7	1.2e+03	11.3	0.1	4.7	2	1	3	5	5	5	4	Uncharacterized protein (Fragment) OS=Lottia gigantea GN=LOTGIDRAFT_131302 PE=4 SV=1
tr|S4RHK8|S4RHK8_PETMA	-	m_Nmar_UR_23_2	-	3.2e-07	37.0	25.1	1.6e+04	7.3	0.1	11.2	9	1	1	10	10	10	6	Uncharacterized protein (Fragment) OS=Petromyzon marinus PE=4 SV=1
tr|T1P979|T1P979_MUSDO	-	m_Atra_UR_13_3	-	0.00024	30.8	5.7	3.5e+05	3.9	0.0	9.2	9	0	0	9	9	9	0	Sperm-tail PG-rich repeat protein OS=Musca domestica PE=2 SV=1
tr|T1PK79|T1PK79_MUSDO	-	m_Atra_UR_13_3	-	0.00058	29.7	6.3	7.2e+05	3.0	0.0	9.2	9	0	0	9	9	9	0	Sperm-tail PG-rich repeat protein OS=Musca domestica PE=2 SV=1
tr|R7UYE0|R7UYE0_CAPTE	-	m_Atra_UR_13_11	-	8.2e-05	27.2	1.2	4.2e+05	3.3	0.0	7.3	7	0	0	7	7	7	3	Uncharacterized protein (Fragment) OS=Capitella teleta GN=CAPTEDRAFT_55497 PE=4 SV=1
tr|V4A249|V4A249_LOTGI	-	m_Atra_UR_14_1	-	4.7e-09	41.7	22.5	6.6e+04	5.5	0.0	12.7	2	1	11	13	13	13	9	Uncharacterized protein (Fragment) OS=Lottia gigantea GN=LOTGIDRAFT_106986 PE=4 SV=1
tr|H2Y9P9|H2Y9P9_CIOSA	-	m_Atra_UR_22_1	-	0.00022	29.5	19.6	1.4e+06	2.2	0.0	13.1	1	1	17	19	19	19	0	Uncharacterized protein (Fragment) OS=Ciona savignyi PE=4 SV=1
tr|H2Y9P9|H2Y9P9_CIOSA	-	m_Atra_UR_22_6	-	8.3e-09	42.0	30.6	1.8e+05	4.8	0.2	15.6	1	1	19	20	20	20	15	Uncharacterized protein (Fragment) OS=Ciona savignyi PE=4 SV=1
tr|G0UTM7|G0UTM7_TRYCI	-	m_Atra_UR_22_14	-	1.7e-12	56.1	54.9	6.7e+05	2.9	0.0	26.7	1	1	28	29	29	29	0	Putative uncharacterized protein OS=Trypanosoma congolense (strain IL3000) GN=TCIL3000_9_1380 PE=4 SV=1
tr|B7Q9L7|B7Q9L7_IXOSC	-	m_Atra_UR_22_22	-	0.00091	28.8	18.3	4.2e+05	3.6	0.0	10.3	1	1	10	11	11	11	2	Putative uncharacterized protein (Fragment) OS=Ixodes scapularis GN=IscW_ISCW011693 PE=4 SV=1
tr|M5J7E6|M5J7E6_9LACO	-	m_Mdub_UR_15_7	-	0.00047	29.6	4.5	1.5e+05	4.7	0.0	9.3	10	0	0	10	10	10	2	Uncharacterized protein OS=Lactobacillus saerimneri 30a GN=D271_00543 PE=4 SV=1
tr|A5KLS0|A5KLS0_9FIRM	-	m_Mdub_UR_15_11	-	8.8e-10	48.0	10.7	7.4e+02	11.9	0.1	6.2	5	0	0	5	5	5	4	Repeat protein OS=Ruminococcus torques ATCC 27756 GN=RUMTOR_01174 PE=4 SV=1
tr|E5XHY6|E5XHY6_9FIRM	-	m_Mdub_UR_15_11	-	5.4e-06	36.5	1.2	97	14.6	0.1	3.7	3	0	0	3	3	3	3	Uncharacterized protein (Fragment) OS=Lachnospiraceae bacterium 8_1_57FAA GN=HMPREF1026_01741 PE=4 SV=1
tr|E5XHY5|E5XHY5_9FIRM	-	m_Mdub_UR_15_11	-	6.9e-06	36.2	3.9	7.8e+02	11.9	0.1	4.2	3	0	0	3	3	3	3	Uncharacterized protein (Fragment) OS=Lachnospiraceae bacterium 8_1_57FAA GN=HMPREF1026_01740 PE=4 SV=1
tr|F4LTD8|F4LTD8_TEPAE	-	m_Mdub_UR_15_11	-	6.1e-05	33.3	1.1	5.3e+03	9.3	0.0	4.9	4	0	0	4	4	4	3	Uncharacterized protein OS=Tepidanaerobacter acetatoxydans (strain DSM 21804 / JCM 16047 / Re1) GN=TEPIRE1_0286 PE=4 SV=1
tr|R5QDA7|R5QDA7_9FIRM	-	m_Mdub_UR_15_11	-	0.0002	31.8	2.4	8.2e+02	11.8	0.1	4.3	3	0	0	3	3	3	2	Uncharacterized protein OS=Ruminococcus torques CAG:61 GN=BN734_00456 PE=4 SV=1
tr|L0MB85|L0MB85_ENTBF	-	m_Mdub_UR_15_12	-	4.9e-08	43.7	0.0	1.7e+05	4.4	0.0	8.5	8	0	0	8	8	8	6	Type 1 secretion C-terminal target domain (VC_A0849 subclass) OS=Enterobacteriaceae bacterium (strain FGI 57) GN=D782_4133 PE=4 SV=1
tr|Q10YX3|Q10YX3_TRIEI	-	m_Mdub_UR_15_17	-	0.00093	30.3	0.2	3.9e+04	6.2	0.0	7.0	7	0	0	7	7	7	3	YD repeat protein OS=Trichodesmium erythraeum (strain IMS101) GN=Tery_3459 PE=4 SV=1
tr|B0CQE1|B0CQE1_LACBS	-	m_Mdub_UR_15_21	-	8e-09	42.8	6.5	9.8e+04	5.4	0.0	12.1	2	2	13	15	15	15	0	Predicted protein OS=Laccaria bicolor (strain S238N-H82 / ATCC MYA-4686) GN=LACBIDRAFT_242697 PE=4 SV=1
tr|B0D5G4|B0D5G4_LACBS	-	m_Mdub_UR_15_21	-	1.6e-07	39.1	2.7	1.5e+05	4.9	0.0	9.3	1	1	10	11	11	11	0	Predicted protein (Fragment) OS=Laccaria bicolor (strain S238N-H82 / ATCC MYA-4686) GN=LACBIDRAFT_161519 PE=4 SV=1
tr|B0DHT5|B0DHT5_LACBS	-	m_Mdub_UR_15_21	-	2.2e-06	35.8	3.4	4.4e+04	6.4	0.0	8.6	2	2	8	10	10	10	2	Predicted protein OS=Laccaria bicolor (strain S238N-H82 / ATCC MYA-4686) GN=LACBIDRAFT_251592 PE=4 SV=1
tr|B0D9C1|B0D9C1_LACBS	-	m_Mdub_UR_15_21	-	3e-06	35.5	2.8	1.5e+05	4.9	0.0	8.9	1	1	9	10	10	10	0	Predicted protein (Fragment) OS=Laccaria bicolor (strain S238N-H82 / ATCC MYA-4686) GN=LACBIDRAFT_160710 PE=4 SV=1
tr|B0CT34|B0CT34_LACBS	-	m_Mdub_UR_15_21	-	9.3e-06	34.0	4.6	9.4e+04	5.4	0.0	9.2	1	1	9	10	10	10	0	Predicted protein OS=Laccaria bicolor (strain S238N-H82 / ATCC MYA-4686) GN=LACBIDRAFT_244568 PE=4 SV=1
tr|B0E477|B0E477_LACBS	-	m_Mdub_UR_15_21	-	3.1e-05	32.5	0.9	2e+04	7.3	0.0	6.7	2	2	5	7	7	7	1	Predicted protein (Fragment) OS=Laccaria bicolor (strain S238N-H82 / ATCC MYA-4686) GN=LACBIDRAFT_164190 PE=4 SV=1
tr|B0E4G9|B0E4G9_LACBS	-	m_Mdub_UR_15_21	-	5.8e-05	31.8	1.9	1.6e+05	4.8	0.0	7.4	2	2	7	9	9	9	0	Predicted protein (Fragment) OS=Laccaria bicolor (strain S238N-H82 / ATCC MYA-4686) GN=LACBIDRAFT_163230 PE=4 SV=1
tr|B0DC92|B0DC92_LACBS	-	m_Mdub_UR_15_21	-	0.00011	31.0	2.4	3.9e+05	3.7	0.0	10.4	3	3	9	13	13	13	0	Predicted protein OS=Laccaria bicolor (strain S238N-H82 / ATCC MYA-4686) GN=LACBIDRAFT_235562 PE=4 SV=1
tr|B0DSH6|B0DSH6_LACBS	-	m_Mdub_UR_15_21	-	0.0007	28.7	1.2	3e+04	6.8	0.0	7.8	2	2	8	10	10	10	1	Predicted protein (Fragment) OS=Laccaria bicolor (strain S238N-H82 / ATCC MYA-4686) GN=LACBIDRAFT_146775 PE=4 SV=1
tr|A7S2A4|A7S2A4_NEMVE	-	m_Mdub_UR_15_22	-	0.00017	28.0	8.7	2.2e+06	1.4	0.0	11.6	13	0	0	13	13	13	0	Predicted protein OS=Nematostella vectensis GN=v1g242342 PE=4 SV=1
tr|C3ZQY2|C3ZQY2_BRAFL	-	m_Mdub_UR_15_24	-	2.7e-09	47.3	21.3	5.2e+03	9.3	0.0	9.6	2	2	5	9	9	9	9	Putative uncharacterized protein OS=Branchiostoma floridae GN=BRAFLDRAFT_87485 PE=4 SV=1
tr|L8ILL1|L8ILL1_9CETA	-	m_HmenF_UR_1_1	-	0.00071	27.3	23.5	8.4e+04	5.6	0.2	9.4	9	0	0	9	9	9	8	Uncharacterized protein OS=Bos mutus GN=M91_06813 PE=4 SV=1
tr|C3ZQ11|C3ZQ11_BRAFL	-	m_HmenF_UR_26_2	-	4.6e-08	36.1	12.6	2.4e+05	3.7	0.0	10.8	11	0	0	11	11	11	8	Putative uncharacterized protein (Fragment) OS=Branchiostoma floridae GN=BRAFLDRAFT_217974 PE=4 SV=1
tr|A7SVX3|A7SVX3_NEMVE	-	m_CmonF_UR_10_1	-	1.2e-05	30.6	4.6	8.3e+03	7.8	0.0	5.2	1	1	4	5	5	5	5	Predicted protein (Fragment) OS=Nematostella vectensis GN=v1g63670 PE=4 SV=1
tr|V4BNX3|V4BNX3_LOTGI	-	m_CmonF_UR_10_1	-	6.5e-05	28.8	2.2	8.8e+04	5.1	0.0	6.0	6	0	0	6	6	6	1	Uncharacterized protein (Fragment) OS=Lottia gigantea GN=LOTGIDRAFT_78339 PE=4 SV=1
tr|A7SSW3|A7SSW3_NEMVE	-	m_CmonF_UR_10_1	-	0.0005	26.5	14.2	9.8e+05	2.4	0.0	11.7	11	0	0	11	11	11	0	Predicted protein OS=Nematostella vectensis GN=v1g216953 PE=4 SV=1
tr|A2HU56|A2HU56_TRIVA	-	m_CmonF_UR_17_1	-	1.7e-05	33.0	15.2	2.1e+05	4.4	0.0	9.1	9	0	0	9	9	9	0	Putative uncharacterized protein (Fragment) OS=Trichomonas vaginalis GN=TVAG_593970 PE=4 SV=1
tr|A0A033UL83|A0A033UL83_STAAU	-	m_CmonF_UR_17_1	-	3.4e-05	32.1	14.9	2.6e+05	4.1	0.0	9.4	2	1	8	10	10	10	0	Uncharacterized protein OS=Staphylococcus aureus C0673 GN=V070_02125 PE=4 SV=1
tr|A2HFQ8|A2HFQ8_TRIVA	-	m_CmonF_UR_17_1	-	9.1e-05	30.9	9.5	1.7e+05	4.6	0.0	7.2	7	0	0	7	7	7	0	Putative uncharacterized protein OS=Trichomonas vaginalis GN=TVAG_141710 PE=4 SV=1
tr|A2H7W9|A2H7W9_TRIVA	-	m_CmonF_UR_17_1	-	0.00036	29.2	10.0	1.6e+05	4.7	0.0	7.1	7	0	0	7	7	7	0	Putative uncharacterized protein (Fragment) OS=Trichomonas vaginalis GN=TVAG_420670 PE=4 SV=1
tr|A0A010V2T1|A0A010V2T1_ACIBA	-	m_CmonF_UR_19_1	-	5e-06	30.1	28.5	1.1e+05	4.7	0.1	11.5	11	0	0	11	11	11	0	Uncharacterized protein OS=Acinetobacter baumannii 121738 GN=J717_1250 PE=4 SV=1
tr|A0A0A8TTV8|A0A0A8TTV8_ACIBZ	-	m_CmonF_UR_19_1	-	2.3e-05	28.5	23.9	5.7e+04	5.4	0.0	9.5	9	0	0	9	9	9	2	Fragment of Orfc273-4 from Vibrio metschnikovii OS=Acinetobacter bereziniae PE=4 SV=1
tr|D0C9W2|D0C9W2_ACIBA	-	m_CmonF_UR_19_1	-	8.6e-05	27.1	23.6	8.4e+04	5.0	0.1	9.6	9	1	0	9	9	9	0	Uncharacterized protein OS=Acinetobacter baumannii ATCC 19606 = CIP 70.34 GN=HMPREF0010_01425 PE=4 SV=1
tr|M2YDJ0|M2YDJ0_ACIBA	-	m_CmonF_UR_19_1	-	8.6e-05	27.1	23.6	8.4e+04	5.0	0.1	9.6	9	1	0	9	9	9	0	Uncharacterized protein OS=Acinetobacter baumannii MSP4-16 GN=G347_03045 PE=4 SV=1
tr|A0A010V2R5|A0A010V2R5_ACIBA	-	m_CmonF_UR_19_1	-	9.4e-05	27.0	17.8	4.1e+04	5.8	0.1	7.7	7	0	0	7	7	7	2	Uncharacterized protein OS=Acinetobacter baumannii 121738 GN=J717_1235 PE=4 SV=1
tr|L9NZF6|L9NZF6_ACIBA	-	m_CmonF_UR_19_1	-	0.00018	26.3	8.4	2.7e+04	6.2	0.1	6.4	6	0	0	6	6	6	1	Uncharacterized protein OS=Acinetobacter baumannii Naval-57 GN=ACINNAV57_0947 PE=4 SV=1
tr|N9JS27|N9JS27_ACIBA	-	m_CmonF_UR_19_1	-	0.0004	25.4	21.1	5.1e+04	5.5	0.1	8.6	8	1	0	8	8	8	1	Uncharacterized protein OS=Acinetobacter baumannii ATCC 19606 = CIP 70.34 GN=F911_03042 PE=4 SV=1
tr|M2ZH54|M2ZH54_ACIBA	-	m_CmonF_UR_19_1	-	0.00085	24.6	21.4	6.9e+04	5.2	0.0	8.3	8	0	0	8	8	8	0	Uncharacterized protein OS=Acinetobacter baumannii MSP4-16 GN=G347_03040 PE=4 SV=1
tr|V4ACV4|V4ACV4_LOTGI	-	m_CmonF_UR_20_3	-	0.00057	26.5	7.9	2.4e+06	1.3	0.0	15.3	24	0	0	24	24	24	0	Uncharacterized protein OS=Lottia gigantea GN=LOTGIDRAFT_176751 PE=4 SV=1
tr|H9GSU9|H9GSU9_ANOCA	-	m_HmenM_UR_3_5	-	6e-07	40.8	28.4	3.2e+04	6.9	0.1	10.9	1	1	13	14	14	14	11	Uncharacterized protein (Fragment) OS=Anolis carolinensis PE=4 SV=1
tr|H9GUI7|H9GUI7_ANOCA	-	m_HmenM_UR_3_5	-	0.00022	32.7	33.2	1.2e+04	8.2	0.5	8.9	1	1	11	12	12	12	11	Uncharacterized protein (Fragment) OS=Anolis carolinensis PE=4 SV=1
tr|A0A0E0B8G3|A0A0E0B8G3_9ORYZ	-	m_HmenM_UR_3_16	-	1.1e-82	274.6	414.5	4.1e+05	3.7	0.1	125.1	128	0	0	128	128	128	127	Uncharacterized protein (Fragment) OS=Oryza glumipatula PE=4 SV=1
tr|V4C094|V4C094_LOTGI	-	m_HmenM_UR_20_1	-	1.9e-09	42.0	33.2	5.1e+05	3.2	0.0	20.2	3	2	20	24	24	24	0	Uncharacterized protein (Fragment) OS=Lottia gigantea GN=LOTGIDRAFT_117769 PE=4 SV=1
tr|T0S4S0|T0S4S0_9STRA	-	m_HmenM_UR_21_4	-	0.00014	33.1	1.8	3.5e+05	3.9	0.0	11.8	15	0	0	15	15	15	0	Uncharacterized protein OS=Saprolegnia diclina VS20 GN=SDRG_02703 PE=4 SV=1
tr|T0SBD4|T0SBD4_9STRA	-	m_HmenM_UR_21_4	-	0.00015	33.0	1.9	3.5e+05	3.9	0.0	11.8	15	0	0	15	15	15	0	Uncharacterized protein OS=Saprolegnia diclina VS20 GN=SDRG_02703 PE=4 SV=1
tr|F0X6V5|F0X6V5_GROCL	-	m_CmonM_UR_16_17	-	2.9e-15	58.3	14.8	5.7e+04	5.7	0.0	12.2	12	0	0	12	12	12	9	Putative uncharacterized protein OS=Grosmannia clavigera (strain kw1407 / UAMH 11150) GN=CMQ_6895 PE=4 SV=1
tr|B8KPF0|B8KPF0_9GAMM	-	m_CmonM_UR_16_17	-	0.00027	28.4	9.9	7.8e+05	2.6	0.0	10.0	11	1	0	11	11	11	0	Putative uncharacterized protein OS=gamma proteobacterium NOR5-3 GN=NOR53_192 PE=4 SV=1
tr|A4A5P6|A4A5P6_9GAMM	-	m_CmonM_UR_16_17	-	0.00062	27.4	3.6	1.3e+06	2.0	0.0	8.9	9	0	0	9	9	9	0	Uncharacterized protein OS=Congregibacter litoralis KT71 GN=KT71_00160 PE=4 SV=1
tr|F0X6V5|F0X6V5_GROCL	-	m_CmonM_UR_16_22	-	2.9e-15	58.3	14.8	5.7e+04	5.7	0.0	12.2	12	0	0	12	12	12	9	Putative uncharacterized protein OS=Grosmannia clavigera (strain kw1407 / UAMH 11150) GN=CMQ_6895 PE=4 SV=1
tr|B8KPF0|B8KPF0_9GAMM	-	m_CmonM_UR_16_22	-	0.00027	28.4	9.9	7.8e+05	2.6	0.0	10.0	11	1	0	11	11	11	0	Putative uncharacterized protein OS=gamma proteobacterium NOR5-3 GN=NOR53_192 PE=4 SV=1
tr|A4A5P6|A4A5P6_9GAMM	-	m_CmonM_UR_16_22	-	0.00062	27.4	3.6	1.3e+06	2.0	0.0	8.9	9	0	0	9	9	9	0	Uncharacterized protein OS=Congregibacter litoralis KT71 GN=KT71_00160 PE=4 SV=1
tr|F6RIH0|F6RIH0_MACMU	-	m_CmonM_UR_24_1	-	3.3e-05	32.9	25.6	2.5e+05	4.5	0.0	11.3	11	0	0	11	11	11	7	Uncharacterized protein (Fragment) OS=Macaca mulatta PE=4 SV=1
tr|A0A093J0F2|A0A093J0F2_EURHL	-	m_CmonM_UR_24_3	-	1.9e-09	42.9	28.8	2.9e+04	6.9	0.1	11.1	11	0	0	11	11	11	0	Zinc finger protein 41 (Fragment) OS=Eurypyga helias GN=N326_13237 PE=4 SV=1
tr|J9K4V2|J9K4V2_ACYPI	-	m_CmonM_UR_24_3	-	7.7e-06	33.1	31.1	5.7e+05	3.3	0.0	14.3	14	0	0	14	14	14	0	Uncharacterized protein (Fragment) OS=Acyrthosiphon pisum GN=LOC100166945 PE=4 SV=2
tr|A0A075M1N1|A0A075M1N1_PANTR	-	m_CmonM_UR_24_3	-	8.3e-06	33.0	32.8	8.7e+05	2.8	0.0	15.5	16	0	0	16	16	16	0	PRDM9 zinc finger domain protein (Fragment) OS=Pan troglodytes GN=PRDM9 PE=4 SV=1
tr|A0A075M1T5|A0A075M1T5_PANTR	-	m_CmonM_UR_24_3	-	2.1e-05	31.9	35.4	9.5e+05	2.7	0.0	16.3	17	0	0	17	17	17	0	PRDM9 zinc finger domain protein (Fragment) OS=Pan troglodytes GN=PRDM9 PE=4 SV=1
tr|I6W7U9|I6W7U9_PANTR	-	m_CmonM_UR_24_3	-	2.3e-05	31.8	35.4	9.5e+05	2.7	0.0	16.3	17	0	0	17	17	17	0	PR domain zinc finger protein 9 (Fragment) OS=Pan troglodytes schweinfurthii GN=PRDM9 PE=4 SV=1
tr|H9GV96|H9GV96_ANOCA	-	m_CmonM_UR_24_3	-	3e-05	31.5	43.9	1.6e+05	4.9	0.1	13.9	14	0	0	14	14	13	0	Uncharacterized protein (Fragment) OS=Anolis carolinensis PE=4 SV=1
tr|R4GC05|R4GC05_ANOCA	-	m_CmonM_UR_24_3	-	3e-05	31.4	52.8	1e+05	5.4	0.1	15.3	15	0	0	15	15	15	0	Uncharacterized protein OS=Anolis carolinensis PE=4 SV=1
tr|L7MZY5|L7MZY5_ANOCA	-	m_CmonM_UR_24_3	-	6.2e-05	30.6	39.6	3.5e+05	3.9	0.1	14.8	15	0	0	15	15	14	0	Uncharacterized protein OS=Anolis carolinensis PE=4 SV=2
tr|A0A075M0D2|A0A075M0D2_PANTR	-	m_CmonM_UR_24_3	-	6.2e-05	30.6	28.1	7.3e+05	3.0	0.0	13.6	14	0	0	14	14	14	0	PRDM9 zinc finger domain protein (Fragment) OS=Pan troglodytes GN=PRDM9 PE=4 SV=1
tr|H9GVJ5|H9GVJ5_ANOCA	-	m_CmonM_UR_24_3	-	6.4e-05	30.6	44.2	1.6e+05	4.8	0.1	14.0	14	0	0	14	14	13	0	Uncharacterized protein (Fragment) OS=Anolis carolinensis PE=4 SV=1
tr|A0A075M1F7|A0A075M1F7_PANTR	-	m_CmonM_UR_24_3	-	6.5e-05	30.5	41.0	7.5e+05	3.0	0.0	17.9	19	0	0	19	19	19	0	PRDM9 zinc finger domain protein (Fragment) OS=Pan troglodytes GN=PRDM9 PE=4 SV=1
tr|A0A075M1P7|A0A075M1P7_PANTR	-	m_CmonM_UR_24_3	-	7e-05	30.4	33.2	8.7e+05	2.8	0.0	15.3	16	0	0	16	16	16	0	PRDM9 zinc finger domain protein (Fragment) OS=Pan troglodytes GN=PRDM9 PE=4 SV=1
tr|J9K2Z9|J9K2Z9_ACYPI	-	m_CmonM_UR_24_3	-	7.4e-05	30.4	33.4	3.6e+05	3.9	0.0	15.4	16	0	0	16	16	15	0	Uncharacterized protein OS=Acyrthosiphon pisum PE=4 SV=2
tr|A0A075M5Z3|A0A075M5Z3_PANTR	-	m_CmonM_UR_24_3	-	7.6e-05	30.3	40.8	1.1e+06	2.6	0.0	17.9	19	0	0	19	19	19	0	PRDM9 zinc finger domain protein (Fragment) OS=Pan troglodytes GN=PRDM9 PE=4 SV=1
tr|A0A075M5Y8|A0A075M5Y8_PANTR	-	m_CmonM_UR_24_3	-	7.8e-05	30.3	40.8	1.1e+06	2.6	0.0	17.9	19	0	0	19	19	19	0	PRDM9 zinc finger domain protein (Fragment) OS=Pan troglodytes GN=PRDM9 PE=4 SV=1
tr|A0A075M1L8|A0A075M1L8_PANTR	-	m_CmonM_UR_24_3	-	8.6e-05	30.2	35.7	9.5e+05	2.7	0.0	16.2	17	0	0	17	17	17	0	PRDM9 zinc finger domain protein (Fragment) OS=Pan troglodytes GN=PRDM9 PE=4 SV=1
tr|I6VCR1|I6VCR1_PANTR	-	m_CmonM_UR_24_3	-	9.3e-05	30.1	35.7	9.5e+05	2.7	0.0	16.2	17	0	0	17	17	17	0	PR domain zinc finger protein 9 (Fragment) OS=Pan troglodytes verus GN=PRDM9 PE=4 SV=1
tr|A0A075M5Z8|A0A075M5Z8_PANTR	-	m_CmonM_UR_24_3	-	9.6e-05	30.1	38.6	7e+05	3.1	0.0	17.0	18	0	0	18	18	18	0	PRDM9 zinc finger domain protein (Fragment) OS=Pan troglodytes GN=PRDM9 PE=4 SV=1
tr|H9G9B5|H9G9B5_ANOCA	-	m_CmonM_UR_24_3	-	0.00011	29.9	50.9	2e+05	4.6	0.1	15.4	15	0	0	15	15	15	0	Uncharacterized protein (Fragment) OS=Anolis carolinensis PE=4 SV=1
tr|L7MZG7|L7MZG7_ANOCA	-	m_CmonM_UR_24_3	-	0.00013	29.7	30.0	2.7e+05	4.2	0.1	12.3	12	0	0	12	12	12	0	Uncharacterized protein OS=Anolis carolinensis PE=4 SV=1
tr|J9JY95|J9JY95_ACYPI	-	m_CmonM_UR_24_3	-	0.00014	29.6	9.0	1.2e+05	5.2	0.1	7.9	7	0	0	7	7	7	0	Uncharacterized protein OS=Acyrthosiphon pisum PE=4 SV=2
tr|A0A075M1K8|A0A075M1K8_PAPCY	-	m_CmonM_UR_24_3	-	0.00016	29.5	20.7	5.3e+05	3.4	0.0	11.1	11	0	0	11	11	11	0	PRDM9 zinc finger domain protein (Fragment) OS=Papio cynocephalus GN=PRDM9 PE=4 SV=1
tr|A0A075M0B2|A0A075M0B2_PANTR	-	m_CmonM_UR_24_3	-	0.00016	29.4	25.1	6.6e+05	3.2	0.0	12.7	13	0	0	13	13	13	0	PRDM9 zinc finger domain protein (Fragment) OS=Pan troglodytes GN=PRDM9 PE=4 SV=1
tr|X1XDB1|X1XDB1_ACYPI	-	m_CmonM_UR_24_3	-	0.00017	29.4	35.6	2.8e+05	4.2	0.0	13.7	14	0	0	14	14	14	0	Uncharacterized protein OS=Acyrthosiphon pisum PE=4 SV=1
tr|A0A075M0C6|A0A075M0C6_PANTR	-	m_CmonM_UR_24_3	-	0.00018	29.3	35.9	9.5e+05	2.7	0.0	16.1	17	0	0	17	17	17	0	PRDM9 zinc finger domain protein (Fragment) OS=Pan troglodytes GN=PRDM9 PE=4 SV=1
tr|A0A075M5Y4|A0A075M5Y4_PANTR	-	m_CmonM_UR_24_3	-	0.00018	29.3	35.9	9.5e+05	2.7	0.0	16.1	17	0	0	17	17	17	0	PRDM9 zinc finger domain protein (Fragment) OS=Pan troglodytes GN=PRDM9 PE=4 SV=1
tr|A0A075M1G1|A0A075M1G1_PANTR	-	m_CmonM_UR_24_3	-	0.00018	29.3	35.9	9.5e+05	2.7	0.0	16.1	17	0	0	17	17	17	0	PRDM9 zinc finger domain protein (Fragment) OS=Pan troglodytes GN=PRDM9 PE=4 SV=1
tr|A0A075M1T0|A0A075M1T0_PANTR	-	m_CmonM_UR_24_3	-	0.00018	29.3	36.3	6.8e+05	3.1	0.0	16.1	17	0	0	17	17	17	0	PRDM9 zinc finger domain protein (Fragment) OS=Pan troglodytes GN=PRDM9 PE=4 SV=1
tr|A0A075M0A5|A0A075M0A5_PANTR	-	m_CmonM_UR_24_3	-	0.00019	29.2	36.3	6.8e+05	3.1	0.0	16.1	17	0	0	17	17	17	0	PRDM9 zinc finger domain protein (Fragment) OS=Pan troglodytes GN=PRDM9 PE=4 SV=1
tr|A0A075M1H5|A0A075M1H5_PANTR	-	m_CmonM_UR_24_3	-	0.00024	29.0	28.3	7.3e+05	3.0	0.0	13.6	14	0	0	14	14	14	0	PRDM9 zinc finger domain protein (Fragment) OS=Pan troglodytes GN=PRDM9 PE=4 SV=1
tr|A0A075M621|A0A075M621_PANTR	-	m_CmonM_UR_24_3	-	0.00025	28.9	28.3	7.3e+05	3.0	0.0	13.5	14	0	0	14	14	14	0	PRDM9 zinc finger domain protein (Fragment) OS=Pan troglodytes GN=PRDM9 PE=4 SV=1
tr|J9JVC4|J9JVC4_ACYPI	-	m_CmonM_UR_24_3	-	0.00026	28.9	17.2	5.6e+04	6.1	0.0	9.9	10	0	0	10	10	8	0	Uncharacterized protein OS=Acyrthosiphon pisum PE=4 SV=1
tr|A0A075M615|A0A075M615_PANTR	-	m_CmonM_UR_24_3	-	0.00026	28.9	28.3	7.3e+05	3.0	0.0	13.5	14	0	0	14	14	14	0	PRDM9 zinc finger domain protein (Fragment) OS=Pan troglodytes GN=PRDM9 PE=4 SV=1
tr|I6W5C4|I6W5C4_PANTR	-	m_CmonM_UR_24_3	-	0.00026	28.9	28.3	7.4e+05	3.0	0.0	13.5	14	0	0	14	14	14	0	PR domain zinc finger protein 9 (Fragment) OS=Pan troglodytes schweinfurthii GN=PRDM9 PE=4 SV=1
tr|A0A075M1Q1|A0A075M1Q1_PANTR	-	m_CmonM_UR_24_3	-	0.00027	28.8	28.4	6.4e+05	3.2	0.0	13.5	14	0	0	14	14	14	0	PRDM9 zinc finger domain protein (Fragment) OS=Pan troglodytes GN=PRDM9 PE=4 SV=1
tr|A0A075M1F2|A0A075M1F2_PANTR	-	m_CmonM_UR_24_3	-	0.00031	28.7	33.8	6.2e+05	3.2	0.0	15.3	16	0	0	16	16	16	0	PRDM9 zinc finger domain protein (Fragment) OS=Pan troglodytes GN=PRDM9 PE=4 SV=1
tr|I6VVM1|I6VVM1_PANTR	-	m_CmonM_UR_24_3	-	0.00034	28.6	33.8	6.3e+05	3.2	0.0	15.3	16	0	0	16	16	16	0	PR domain zinc finger protein 9 (Fragment) OS=Pan troglodytes troglodytes GN=PRDM9 PE=4 SV=1
tr|I6VCQ8|I6VCQ8_PANTR	-	m_CmonM_UR_24_3	-	0.00041	28.3	23.1	4.3e+05	3.7	0.0	11.8	12	0	0	12	12	12	0	PR domain zinc finger protein 9 (Fragment) OS=Pan troglodytes troglodytes GN=PRDM9 PE=4 SV=1
tr|H9GQ53|H9GQ53_ANOCA	-	m_CmonM_UR_24_3	-	0.00042	28.3	29.9	2.6e+05	4.3	0.1	12.2	12	0	0	12	12	12	0	Uncharacterized protein (Fragment) OS=Anolis carolinensis PE=4 SV=1
tr|A0A075M1M8|A0A075M1M8_PANTR	-	m_CmonM_UR_24_3	-	0.00046	28.2	38.6	1e+06	2.6	0.0	16.9	18	0	0	18	18	18	0	PRDM9 zinc finger domain protein (Fragment) OS=Pan troglodytes GN=PRDM9 PE=4 SV=1
tr|X1XPN5|X1XPN5_ACYPI	-	m_CmonM_UR_24_3	-	0.00052	28.0	22.0	6.3e+04	5.9	0.1	9.6	9	1	0	9	9	8	0	Uncharacterized protein OS=Acyrthosiphon pisum PE=4 SV=1
tr|G1KXE3|G1KXE3_ANOCA	-	m_CmonM_UR_24_3	-	0.00057	28.0	60.4	9.4e+04	5.5	0.1	19.1	20	0	0	20	20	19	0	Uncharacterized protein (Fragment) OS=Anolis carolinensis PE=4 SV=1
tr|H9GTR3|H9GTR3_ANOCA	-	m_CmonM_UR_24_3	-	0.0006	27.9	39.4	1.8e+05	4.7	0.1	14.7	15	0	0	15	15	14	0	Uncharacterized protein OS=Anolis carolinensis PE=4 SV=1

```
tr|A0A075M1M3|A0A075M1M3_PANTR -          m_CmonM_UR_24_3      -           0.00061   27.9  41.3   1.1e+06    2.6   0.0  17.7  19    0    0  19  19  19    0 PRDM9 zinc finger domain protein (Fragment) OS=Pan troglodytes GN=PRDM9 PE=4 SV=1
tr|X1XP18|X1XP18_ACYPI          -          m_CmonM_UR_24_3      -           0.00069   27.7  30.1   2.4e+05    4.3   0.0  13.2  13    0    0  13  13  13    0 Uncharacterized protein OS=Acyrthosiphon pisum GN=pdp1 PE=4 SV=1
tr|H2R5H8|H2R5H8_PANTR          -          m_CmonM_UR_24_3      -           0.00082   27.5  41.7     9e+05    2.8   0.1  18.1  19    0    0  19  19  19    0 Uncharacterized protein (Fragment) OS=Pan troglodytes PE=4 SV=1
tr|A0A075M0H4|A0A075M0H4_CALJA -          m_CmonM_UR_24_3      -           0.00087   27.4  19.0   4.7e+05    3.6   0.0  10.1  10    0    0  10  10  10    0 PRDM9 zinc finger domain protein (Fragment) OS=Callithrix jacchus GN=PRDM9 PE=4 SV=1
tr|A0A075M1G6|A0A075M1G6_PANTR -          m_CmonM_UR_24_3      -           0.00092   27.4  38.8     1e+06    2.6   0.0  16.9  18    0    0  18  18  18    0 PRDM9 zinc finger domain protein (Fragment) OS=Pan troglodytes GN=PRDM9 PE=4 SV=1
tr|A0A075M0B9|A0A075M0B9_PANTR -          m_CmonM_UR_24_3      -           0.00092   27.4  38.8     1e+06    2.6   0.0  16.9  18    0    0  18  18  18    0 PRDM9 zinc finger domain protein (Fragment) OS=Pan troglodytes GN=PRDM9 PE=4 SV=1
tr|T1HAU4|T1HAU4_RHOPR          -          m_CmonM_UR_24_5      -           0.00068   27.3   7.0   5.6e+05    2.9   0.0  10.9  12    0    0  12  12  12    0 Uncharacterized protein OS=Rhodnius prolixus PE=4 SV=1
tr|A7S1D4|A7S1D4_NEMVE -        m_CmonM_UR_27_2      -          5.7e-10   48.1  46.9   2.1e+05    4.4   0.3  20.8   1    1  27  29  29  29    0 Predicted protein (Fragment) OS=Nematostella vectensis GN=v1g100921 PE=4 SV=1
tr|A7T938|A7T938_NEMVE -        m_CmonM_UR_27_2      -          9.9e-10   47.4  30.0   1.5e+05    4.8   0.2  15.6   1    1  23  24  24  24    1 Predicted protein (Fragment) OS=Nematostella vectensis GN=v1g224033 PE=4 SV=1
tr|G3X2E1|G3X2E1_SARHA -        m_CmonM_UR_27_2      -          4.6e-05   33.3  75.5   1.9e+04    7.5   0.6  14.3   1    1  19  21  21  21   19 Uncharacterized protein (Fragment) OS=Sarcophilus harrisii PE=4 SV=1
```

• vs lineage-specific mitochondrial ORFans of non-unionoid DUI species

No results

• vs themselves

```
#                                                               --- full sequence ---- --- best 1 domain ---- --- domain number estimation ----
# target name        accession  query name           accession    E-value  score  bias   E-value  score  bias   exp reg clu  ov env dom rep inc description of target
#------------------- ---------- -------------------- ---------- --------- ------ ----- --------- ------ -----   --- --- --- --- --- --- --- --- ---------------------
CmonF_FORF           -          CmonF_FORF           -            2.5e-57  186.3  13.3   2.8e-57  186.2  13.3   1.0   1   0   0   1   1   1   1 -
CmonM_MORF           -          CmonM_MORF           -            4.3e-61  198.5  11.3   4.8e-61  198.3  11.3   1.0   1   0   0   1   1   1   1 -
a_CmonM_UR_24_11     -          CmonM_MORF           -              3e-05   19.6   3.0     3e-05   19.6   3.0   1.7   1   1   1   2   2   2   1 [447 - 67] (REVERSE SENSE)
m_CmonM_UR_24_11     -          CmonM_MORF           -              3e-05   19.6   3.0     3e-05   19.6   3.0   1.7   1   1   1   2   2   2   1 [447 - 67] (REVERSE SENSE)
HmenF_FORF           -          HmenF_FORF           -            2.6e-56  182.4   9.8   2.9e-56  182.3   9.8   1.0   1   0   0   1   1   1   1 -
HmenM_MORF           -          HmenM_MORF           -           5.3e-206  678.1  29.2    6e-206  678.0  29.2   1.0   1   0   0   1   1   1   1 -
a_Nmar_UR_2_1        -          a_Nmar_UR_2_1        -            1.3e-05   17.0   0.3   1.3e-05   17.0   0.3   1.0   1   0   0   1   1   1   1 [1 - 30]
m_Nmar_UR_2_1        -          a_Nmar_UR_2_1        -            3.2e-05   16.1   0.3   3.2e-05   16.1   0.3   1.0   1   0   0   1   1   1   1 [1 - 30]
a_Nmar_UR_2_2        -          a_Nmar_UR_2_2        -            4.3e-06   18.4   0.3   4.3e-06   18.4   0.3   1.0   1   0   0   1   1   1   1 [30 - 59]
m_Nmar_UR_2_2        -          a_Nmar_UR_2_2        -            4.3e-06   18.4   0.3   4.3e-06   18.4   0.3   1.0   1   0   0   1   1   1   1 [30 - 59]
a_Nmar_UR_2_3        -          a_Nmar_UR_2_3        -            1.7e-12   39.7   0.6   1.8e-12   39.6   0.6   1.0   1   0   0   1   1   1   1 [5 - 61]
m_Nmar_UR_2_3        -          a_Nmar_UR_2_3        -            3.4e-12   38.8   0.5   3.4e-12   38.8   0.5   1.0   1   0   0   1   1   1   1 [5 - 61]
a_Nmar_UR_2_4        -          a_Nmar_UR_2_4        -              9e-07   19.6   0.3     9e-07   19.6   0.3   1.0   1   0   0   1   1   1   1 [54 - 19] (REVERSE SENSE)
m_Nmar_UR_2_4        -          a_Nmar_UR_2_4        -              9e-07   19.6   0.3     9e-07   19.6   0.3   1.0   1   0   0   1   1   1   1 [54 - 19] (REVERSE SENSE)
a_Nmar_UR_2_5        -          a_Nmar_UR_2_5        -            9.1e-10   31.0   1.3   9.2e-10   30.9   1.3   1.0   1   0   0   1   1   1   1 [47 - 3] (REVERSE SENSE)
m_Nmar_UR_2_5        -          a_Nmar_UR_2_5        -            9.1e-10   31.0   1.3   9.2e-10   30.9   1.3   1.0   1   0   0   1   1   1   1 [47 - 3] (REVERSE SENSE)
a_Nmar_UR_3_1        -          a_Nmar_UR_3_1        -            9.7e-06   17.4   0.7   9.7e-06   17.4   0.7   1.0   1   0   0   1   1   1   1 [6 - 35]
m_Nmar_UR_3_1        -          a_Nmar_UR_3_1        -            2.1e-05   16.6   0.5   2.1e-05   16.6   0.5   1.0   1   0   0   1   1   1   1 [6 - 35]
a_Nmar_UR_3_2        -          a_Nmar_UR_3_2        -            3.9e-06   18.7   0.1   3.9e-06   18.7   0.1   1.0   1   0   0   1   1   1   1 [57 - 92]
m_Nmar_UR_3_2        -          a_Nmar_UR_3_2        -            8.5e-06   17.9   0.1   8.5e-06   17.9   0.1   1.0   1   0   0   1   1   1   1 [57 - 92]
a_Nmar_UR_3_3        -          a_Nmar_UR_3_3        -            1.6e-05   16.7   1.9   1.6e-05   16.7   1.9   1.0   1   0   0   1   1   1   1 [91 - 120]
m_Nmar_UR_3_3        -          a_Nmar_UR_3_3        -            6.1e-05   15.4   2.3   6.1e-05   15.4   2.3   1.1   1   0   0   1   1   1   1 [91 - 120]
a_Nmar_UR_3_4        -          a_Nmar_UR_3_4        -            1.9e-26   84.9   6.6     2e-26   84.8   6.6   1.0   1   0   0   1   1   1   1 [35 - 154]
m_Nmar_UR_3_4        -          a_Nmar_UR_3_4        -            1.9e-26   84.9   6.6     2e-26   84.8   6.6   1.0   1   0   0   1   1   1   1 [35 - 154]
a_Nmar_UR_3_5        -          a_Nmar_UR_3_5        -            1.4e-10   33.8   5.1   1.5e-10   33.8   5.1   1.0   1   0   0   1   1   1   1 [167 - 220]
m_Nmar_UR_3_5        -          a_Nmar_UR_3_5        -            2.7e-10   33.1   4.9   2.7e-10   33.1   4.9   1.0   1   0   0   1   1   1   1 [167 - 220]
a_Nmar_UR_3_6        -          a_Nmar_UR_3_6        -            1.5e-26   85.2  12.3   1.6e-26   85.1  12.3   1.0   1   0   0   1   1   1   1 [120 - 254]
m_Nmar_UR_3_6        -          a_Nmar_UR_3_6        -            1.5e-26   85.2  12.3   1.6e-26   85.1  12.3   1.0   1   0   0   1   1   1   1 [120 - 254]
a_Nmar_UR_3_7        -          a_Nmar_UR_3_7        -            2.2e-43  140.5  11.0   2.4e-43  140.4  11.0   1.0   1   0   0   1   1   1   1 [154 - 357]
m_Nmar_UR_3_7        -          a_Nmar_UR_3_7        -            2.2e-43  140.5  11.0   2.4e-43  140.4  11.0   1.0   1   0   0   1   1   1   1 [154 - 357]
a_Nmar_UR_3_8        -          a_Nmar_UR_3_8        -            2.9e-09   28.0   0.4   2.9e-09   28.0   0.4   1.0   1   0   0   1   1   1   1 [357 - 407]
m_Nmar_UR_3_8        -          a_Nmar_UR_3_8        -            2.9e-09   28.0   0.4   2.9e-09   28.0   0.4   1.0   1   0   0   1   1   1   1 [357 - 407]
a_Nmar_UR_3_9        -          a_Nmar_UR_3_9        -              4e-34  109.7   3.1   4.3e-34  109.6   3.1   1.0   1   0   0   1   1   1   1 [332 - 487]
m_Nmar_UR_3_9        -          a_Nmar_UR_3_9        -              4e-34  109.7   3.1   4.3e-34  109.6   3.1   1.0   1   0   0   1   1   1   1 [332 - 487]
a_Nmar_UR_3_10       -          a_Nmar_UR_3_10       -            2.5e-12   38.4   0.3   2.5e-12   38.4   0.3   1.0   1   0   0   1   1   1   1 [484 - 537]
m_Nmar_UR_3_10       -          a_Nmar_UR_3_10       -            5.3e-12   37.5   0.4   5.3e-12   37.5   0.4   1.0   1   0   0   1   1   1   1 [484 - 537]
a_Nmar_UR_3_11       -          a_Nmar_UR_3_11       -            3.6e-07   19.9   0.2   3.6e-07   19.9   0.2   1.0   1   0   0   1   1   1   1 [516 - 554]
m_Nmar_UR_3_11       -          a_Nmar_UR_3_11       -            3.6e-07   19.9   0.2   3.6e-07   19.9   0.2   1.0   1   0   0   1   1   1   1 [516 - 554]
a_Nmar_UR_3_12       -          a_Nmar_UR_3_12       -            3.4e-19   60.6   0.1   3.6e-19   60.5   0.1   1.0   1   0   0   1   1   1   1 [524 - 607]
m_Nmar_UR_3_12       -          a_Nmar_UR_3_12       -              7e-19   59.6   0.1   7.3e-19   59.6   0.1   1.0   1   0   0   1   1   1   1 [524 - 607]
a_Nmar_UR_3_13       -          a_Nmar_UR_3_13       -            1.6e-10   32.3   0.4   1.6e-10   32.3   0.4   1.0   1   0   0   1   1   1   1 [576 - 623]
m_Nmar_UR_3_13       -          a_Nmar_UR_3_13       -            3.6e-10   31.4   0.4   3.6e-10   31.4   0.4   1.0   1   0   0   1   1   1   1 [576 - 623]
a_Nmar_UR_3_14       -          a_Nmar_UR_3_14       -            2.1e-18   58.2   1.4   2.2e-18   58.2   1.4   1.0   1   0   0   1   1   1   1 [565 - 654]
m_Nmar_UR_3_14       -          a_Nmar_UR_3_14       -            2.1e-18   58.2   1.4   2.2e-18   58.2   1.4   1.0   1   0   0   1   1   1   1 [565 - 654]
a_Nmar_UR_3_15       -          a_Nmar_UR_3_15       -            1.3e-14   46.7   6.9   1.4e-14   46.6   6.9   1.0   1   0   0   1   1   1   1 [639 - 713]
m_Nmar_UR_3_15       -          a_Nmar_UR_3_15       -            2.8e-14   45.7   7.0   2.9e-14   45.6   7.0   1.0   1   0   0   1   1   1   1 [639 - 713]
a_Nmar_UR_3_16       -          a_Nmar_UR_3_16       -            5.2e-12   37.3   1.3   5.3e-12   37.3   1.3   1.0   1   0   0   1   1   1   1 [710 - 766]
m_Nmar_UR_3_16       -          a_Nmar_UR_3_16       -            5.2e-12   37.3   1.3   5.3e-12   37.3   1.3   1.0   1   0   0   1   1   1   1 [710 - 766]
a_Nmar_UR_3_17       -          a_Nmar_UR_3_17       -            1.1e-08   27.1   0.1   1.1e-08   27.1   0.1   1.0   1   0   0   1   1   1   1 [776 - 814]
m_Nmar_UR_3_17       -          a_Nmar_UR_3_17       -            1.1e-08   27.1   0.1   1.1e-08   27.1   0.1   1.0   1   0   0   1   1   1   1 [776 - 814]
a_Nmar_UR_3_18       -          a_Nmar_UR_3_18       -              5e-17   53.6   0.2   5.2e-17   53.5   0.2   1.0   1   0   0   1   1   1   1 [753 - 830]
m_Nmar_UR_3_18       -          a_Nmar_UR_3_18       -              1e-16   52.6   0.3   1.1e-16   52.6   0.3   1.0   1   0   0   1   1   1   1 [753 - 830]
a_Nmar_UR_3_19       -          a_Nmar_UR_3_19       -            6.7e-19   59.6   0.1     7e-19   59.5   0.1   1.0   1   0   0   1   1   1   1 [748 - 840]
m_Nmar_UR_3_19       -          a_Nmar_UR_3_19       -            1.3e-18   58.7   0.1   1.4e-18   58.6   0.1   1.0   1   0   0   1   1   1   1 [748 - 840]
a_Nmar_UR_3_20       -          a_Nmar_UR_3_20       -            7.1e-33  106.0   4.6   7.7e-33  105.9   4.6   1.0   1   0   0   1   1   1   1 [848 - 1006]
m_Nmar_UR_3_20       -          a_Nmar_UR_3_20       -            1.2e-32  105.3   4.4   1.3e-32  105.2   4.4   1.0   1   0   0   1   1   1   1 [848 - 1006]
a_Nmar_UR_3_21       -          a_Nmar_UR_3_21       -            2.7e-35  113.9   2.4   2.9e-35  113.8   2.4   1.0   1   0   0   1   1   1   1 [898 - 1050]
m_Nmar_UR_3_21       -          a_Nmar_UR_3_21       -            5.3e-35  112.9   2.4   5.7e-35  112.8   2.4   1.0   1   0   0   1   1   1   1 [898 - 1050]
a_Nmar_UR_3_22       -          a_Nmar_UR_3_22       -            4.5e-06   18.1   0.3   4.5e-06   18.1   0.3   1.0   1   0   0   1   1   1   1 [1031 - 1060]
m_Nmar_UR_3_22       -          a_Nmar_UR_3_22       -              8e-06   17.5   0.3     8e-06   17.5   0.3   1.0   1   0   0   1   1   1   1 [1031 - 1060]
a_Nmar_UR_3_23       -          a_Nmar_UR_3_23       -            8.4e-52  167.9   9.4   9.2e-52  167.8   9.4   1.0   1   0   0   1   1   1   1 [840 - 1088]
m_Nmar_UR_3_23       -          a_Nmar_UR_3_23       -            8.4e-52  167.9   9.4   9.2e-52  167.8   9.4   1.0   1   0   0   1   1   1   1 [840 - 1088]
a_Nmar_UR_3_24       -          a_Nmar_UR_3_24       -            3.5e-06   18.3   0.3   3.5e-06   18.3   0.3   1.0   1   0   0   1   1   1   1 [1146 - 1181]
m_Nmar_UR_3_24       -          a_Nmar_UR_3_24       -            3.5e-06   18.3   0.3   3.5e-06   18.3   0.3   1.0   1   0   0   1   1   1   1 [1146 - 1181]
a_Nmar_UR_3_25       -          a_Nmar_UR_3_25       -            3.3e-33  106.6   6.2   3.6e-33  106.5   6.2   1.0   1   0   0   1   1   1   1 [1060 - 1206]
m_Nmar_UR_3_25       -          a_Nmar_UR_3_25       -            3.3e-33  106.6   6.2   3.6e-33  106.5   6.2   1.0   1   0   0   1   1   1   1 [1060 - 1206]
a_Nmar_UR_3_26       -          a_Nmar_UR_3_26       -            1.1e-11   36.7   3.0   1.2e-11   36.7   3.0   1.0   1   0   0   1   1   1   1 [1328 - 1381]
m_Nmar_UR_3_26       -          a_Nmar_UR_3_26       -            1.1e-11   36.7   3.0   1.2e-11   36.7   3.0   1.0   1   0   0   1   1   1   1 [1328 - 1381]
a_Nmar_UR_3_27       -          a_Nmar_UR_3_27       -            2.6e-39  126.5   6.1   2.9e-39  126.4   6.1   1.0   1   0   0   1   1   1   1 [1203 - 1391]
m_Nmar_UR_3_27       -          a_Nmar_UR_3_27       -            5.4e-39  125.6   6.2   5.8e-39  125.5   6.2   1.0   1   0   0   1   1   1   1 [1203 - 1391]
a_Nmar_UR_3_28       -          a_Nmar_UR_3_28       -            1.2e-07   22.9   0.6   1.2e-07   22.9   0.6   1.0   1   0   0   1   1   1   1 [1378 - 1416]
m_Nmar_UR_3_28       -          a_Nmar_UR_3_28       -            1.2e-07   22.9   0.6   1.2e-07   22.9   0.6   1.0   1   0   0   1   1   1   1 [1378 - 1416]
a_Nmar_UR_3_29       -          a_Nmar_UR_3_29       -            5.4e-09   28.9   1.4   5.4e-09   28.9   1.4   1.0   1   0   0   1   1   1   1 [1371 - 1327] (REVERSE SENSE)
m_Nmar_UR_3_29       -          a_Nmar_UR_3_29       -              7e-09   28.6   1.0     7e-09   28.6   1.0   1.0   1   0   0   1   1   1   1 [1371 - 1327] (REVERSE SENSE)
a_Nmar_UR_3_30       -          a_Nmar_UR_3_30       -            4.5e-22   71.0   7.3   4.7e-22   70.9   7.3   1.0   1   0   0   1   1   1   1 [1405 - 1298] (REVERSE SENSE)
m_Nmar_UR_3_30       -          a_Nmar_UR_3_30       -            4.5e-22   71.0   7.3   4.7e-22   70.9   7.3   1.0   1   0   0   1   1   1   1 [1405 - 1298] (REVERSE SENSE)
```

a_Nmar_UR_3_31	-	a_Nmar_UR_3_31	-	5.6e-06	18.1	1.6	5.6e-06	18.1	1.6	1.0	1	0	0	1	1	1	1	[1323 - 1291] (REVERSE SENSE)
m_Nmar_UR_3_31	-	a_Nmar_UR_3_31	-	7.5e-06	17.8	1.3	7.5e-06	17.8	1.3	1.0	1	0	0	1	1	1	1	[1323 - 1291] (REVERSE SENSE)
a_Nmar_UR_3_32	-	a_Nmar_UR_3_32	-	7.4e-09	28.1	2.3	7.4e-09	28.1	2.3	1.0	1	0	0	1	1	1	1	[1199 - 1158] (REVERSE SENSE)
m_Nmar_UR_3_32	-	a_Nmar_UR_3_32	-	1e-08	27.6	1.8	1e-08	27.6	1.8	1.0	1	0	0	1	1	1	1	[1199 - 1158] (REVERSE SENSE)
a_Nmar_UR_3_33	-	a_Nmar_UR_3_33	-	8.5e-31	98.6	4.5	9.3e-31	98.5	4.5	1.0	1	0	0	1	1	1	1	[1288 - 1133] (REVERSE SENSE)
m_Nmar_UR_3_33	-	a_Nmar_UR_3_33	-	8.5e-31	98.6	4.5	9.3e-31	98.5	4.5	1.0	1	0	0	1	1	1	1	[1288 - 1133] (REVERSE SENSE)
a_Nmar_UR_3_34	-	a_Nmar_UR_3_34	-	1.1e-06	20.2	0.4	1.1e-06	20.2	0.4	1.0	1	0	0	1	1	1	1	[1127 - 1089] (REVERSE SENSE)
m_Nmar_UR_3_34	-	a_Nmar_UR_3_34	-	1.1e-06	20.2	0.4	1.1e-06	20.2	0.4	1.0	1	0	0	1	1	1	1	[1127 - 1089] (REVERSE SENSE)
a_Nmar_UR_3_35	-	a_Nmar_UR_3_35	-	2.4e-36	117.5	4.9	2.6e-36	117.4	4.9	1.0	1	0	0	1	1	1	1	[1167 - 1009] (REVERSE SENSE)
m_Nmar_UR_3_35	-	a_Nmar_UR_3_35	-	4.3e-36	116.7	5.1	4.6e-36	116.6	5.1	1.0	1	0	0	1	1	1	1	[1167 - 1009] (REVERSE SENSE)
a_Nmar_UR_3_36	-	a_Nmar_UR_3_36	-	1e-34	111.9	2.0	1.1e-34	111.8	2.0	1.0	1	0	0	1	1	1	1	[1079 - 930] (REVERSE SENSE)
m_Nmar_UR_3_36	-	a_Nmar_UR_3_36	-	1e-34	111.9	2.0	1.1e-34	111.8	2.0	1.0	1	0	0	1	1	1	1	[1079 - 930] (REVERSE SENSE)
a_Nmar_UR_3_37	-	a_Nmar_UR_3_37	-	1.2e-34	111.7	3.4	1.3e-34	111.6	3.4	1.0	1	0	0	1	1	1	1	[1072 - 902] (REVERSE SENSE)
m_Nmar_UR_3_37	-	a_Nmar_UR_3_37	-	1.2e-34	111.7	3.4	1.3e-34	111.6	3.4	1.0	1	0	0	1	1	1	1	[1072 - 902] (REVERSE SENSE)
a_Nmar_UR_3_38	-	a_Nmar_UR_3_38	-	4.8e-08	24.1	0.6	4.8e-08	24.1	0.6	1.0	1	0	0	1	1	1	1	[899 - 855] (REVERSE SENSE)
m_Nmar_UR_3_38	-	a_Nmar_UR_3_38	-	4.8e-08	24.1	0.6	4.8e-08	24.1	0.6	1.0	1	0	0	1	1	1	1	[899 - 855] (REVERSE SENSE)
a_Nmar_UR_3_39	-	a_Nmar_UR_3_39	-	6.7e-35	112.5	1.2	7.3e-35	112.4	1.2	1.0	1	0	0	1	1	1	1	[1002 - 841] (REVERSE SENSE)
m_Nmar_UR_3_39	-	a_Nmar_UR_3_39	-	6.7e-35	112.5	1.2	7.3e-35	112.4	1.2	1.0	1	0	0	1	1	1	1	[1002 - 841] (REVERSE SENSE)
a_Nmar_UR_3_40	-	a_Nmar_UR_3_40	-	1e-14	46.8	0.6	1.1e-14	46.7	0.6	1.0	1	0	0	1	1	1	1	[829 - 767] (REVERSE SENSE)
m_Nmar_UR_3_40	-	a_Nmar_UR_3_40	-	1.6e-14	46.2	0.5	1.6e-14	46.2	0.5	1.0	1	0	0	1	1	1	1	[829 - 767] (REVERSE SENSE)
a_Nmar_UR_3_41	-	a_Nmar_UR_3_41	-	4.2e-31	100.1	3.3	4.6e-31	100.0	3.3	1.0	1	0	0	1	1	1	1	[848 - 702] (REVERSE SENSE)
m_Nmar_UR_3_41	-	a_Nmar_UR_3_41	-	5.8e-31	99.7	3.2	6.3e-31	99.6	3.2	1.0	1	0	0	1	1	1	1	[848 - 702] (REVERSE SENSE)
a_Nmar_UR_3_42	-	a_Nmar_UR_3_42	-	9.3e-38	121.8	2.2	1e-37	121.7	2.2	1.0	1	0	0	1	1	1	1	[807 - 640] (REVERSE SENSE)
m_Nmar_UR_3_42	-	a_Nmar_UR_3_42	-	1.4e-37	121.3	1.8	1.5e-37	121.2	1.8	1.0	1	0	0	1	1	1	1	[807 - 640] (REVERSE SENSE)
a_Nmar_UR_3_43	-	a_Nmar_UR_3_43	-	4.4e-30	97.3	11.5	4.8e-30	97.2	11.5	1.0	1	0	0	1	1	1	1	[760 - 626] (REVERSE SENSE)
m_Nmar_UR_3_43	-	a_Nmar_UR_3_43	-	7.3e-30	96.6	11.6	7.8e-30	96.5	11.6	1.0	1	0	0	1	1	1	1	[760 - 626] (REVERSE SENSE)
a_Nmar_UR_3_44	-	a_Nmar_UR_3_44	-	1e-09	29.8	0.1	1e-09	29.8	0.1	1.0	1	0	0	1	1	1	1	[622 - 575] (REVERSE SENSE)
m_Nmar_UR_3_44	-	a_Nmar_UR_3_44	-	1e-09	29.8	0.1	1e-09	29.8	0.1	1.0	1	0	0	1	1	1	1	[622 - 575] (REVERSE SENSE)
a_Nmar_UR_3_45	-	a_Nmar_UR_3_45	-	8.5e-12	37.7	1.9	8.7e-12	37.7	1.9	1.0	1	0	0	1	1	1	1	[582 - 529] (REVERSE SENSE)
m_Nmar_UR_3_45	-	a_Nmar_UR_3_45	-	1.4e-11	37.1	1.5	1.4e-11	37.1	1.5	1.0	1	0	0	1	1	1	1	[582 - 529] (REVERSE SENSE)
a_Nmar_UR_3_46	-	a_Nmar_UR_3_46	-	2.4e-10	31.2	0.1	2.4e-10	31.2	0.1	1.0	1	0	0	1	1	1	1	[547 - 494] (REVERSE SENSE)
m_Nmar_UR_3_46	-	a_Nmar_UR_3_46	-	2.4e-10	31.2	0.1	2.4e-10	31.2	0.1	1.0	1	0	0	1	1	1	1	[547 - 494] (REVERSE SENSE)
a_Nmar_UR_3_47	-	a_Nmar_UR_3_47	-	1.3e-07	23.5	0.1	1.3e-07	23.5	0.1	1.0	1	0	0	1	1	1	1	[525 - 490] (REVERSE SENSE)
m_Nmar_UR_3_47	-	a_Nmar_UR_3_47	-	3.1e-07	22.5	0.2	3.1e-07	22.5	0.2	1.0	1	0	0	1	1	1	1	[525 - 490] (REVERSE SENSE)
a_Nmar_UR_3_48	-	a_Nmar_UR_3_48	-	3.2e-60	195.7	3.2	3.6e-60	195.6	3.2	1.0	1	0	0	1	1	1	1	[653 - 381] (REVERSE SENSE)
m_Nmar_UR_3_48	-	a_Nmar_UR_3_48	-	5.9e-60	194.9	3.2	6.5e-60	194.8	3.2	1.0	1	0	0	1	1	1	1	[653 - 381] (REVERSE SENSE)
a_Nmar_UR_3_49	-	a_Nmar_UR_3_49	-	5.7e-21	66.8	5.1	6e-21	66.8	5.1	1.0	1	0	0	1	1	1	1	[406 - 305] (REVERSE SENSE)
m_Nmar_UR_3_49	-	a_Nmar_UR_3_49	-	1.2e-20	65.8	5.2	1.3e-20	65.8	5.2	1.0	1	0	0	1	1	1	1	[406 - 305] (REVERSE SENSE)
a_Nmar_UR_3_50	-	a_Nmar_UR_3_50	-	2.2e-13	41.7	0.3	2.3e-13	41.6	0.3	1.0	1	0	0	1	1	1	1	[314 - 249] (REVERSE SENSE)
m_Nmar_UR_3_50	-	a_Nmar_UR_3_50	-	2.2e-13	41.7	0.3	2.3e-13	41.6	0.3	1.0	1	0	0	1	1	1	1	[314 - 249] (REVERSE SENSE)
a_Nmar_UR_3_51	-	a_Nmar_UR_3_51	-	2.1e-12	39.7	1.9	2.2e-12	39.7	1.9	1.0	1	0	0	1	1	1	1	[286 - 233] (REVERSE SENSE)
m_Nmar_UR_3_51	-	a_Nmar_UR_3_51	-	2.9e-12	39.4	1.6	3e-12	39.3	1.6	1.0	1	0	0	1	1	1	1	[286 - 233] (REVERSE SENSE)
a_Nmar_UR_3_52	-	a_Nmar_UR_3_52	-	5.1e-12	37.8	0.7	5.2e-12	37.7	0.7	1.0	1	0	0	1	1	1	1	[255 - 202] (REVERSE SENSE)
m_Nmar_UR_3_52	-	a_Nmar_UR_3_52	-	9.9e-12	36.9	0.5	9.9e-12	36.9	0.5	1.0	1	0	0	1	1	1	1	[255 - 202] (REVERSE SENSE)
a_Nmar_UR_3_53	-	a_Nmar_UR_3_53	-	3.6e-21	67.9	4.3	3.8e-21	67.8	4.3	1.0	1	0	0	1	1	1	1	[245 - 141] (REVERSE SENSE)
m_Nmar_UR_3_53	-	a_Nmar_UR_3_53	-	5.8e-21	67.3	4.4	6.1e-21	67.2	4.4	1.0	1	0	0	1	1	1	1	[245 - 141] (REVERSE SENSE)
a_Nmar_UR_3_54	-	a_Nmar_UR_3_54	-	2.8e-28	90.3	2.9	3e-28	90.2	2.9	1.0	1	0	0	1	1	1	1	[229 - 95] (REVERSE SENSE)
m_Nmar_UR_3_54	-	a_Nmar_UR_3_54	-	4.1e-28	89.8	2.8	4.4e-28	89.7	2.8	1.0	1	0	0	1	1	1	1	[229 - 95] (REVERSE SENSE)
a_Nmar_UR_3_55	-	a_Nmar_UR_3_55	-	2.8e-08	27.1	6.0	2.8e-08	27.1	6.0	1.0	1	0	0	1	1	1	1	[119 - 78] (REVERSE SENSE)
m_Nmar_UR_3_55	-	a_Nmar_UR_3_55	-	2.8e-08	27.1	6.0	2.8e-08	27.1	6.0	1.0	1	0	0	1	1	1	1	[119 - 78] (REVERSE SENSE)
a_Nmar_UR_3_56	-	a_Nmar_UR_3_56	-	6.3e-24	77.7	15.9	6.8e-24	77.6	15.9	1.0	1	0	0	1	1	1	1	[183 - 70] (REVERSE SENSE)
m_Nmar_UR_3_56	-	a_Nmar_UR_3_56	-	6.3e-24	77.7	15.9	6.8e-24	77.6	15.9	1.0	1	0	0	1	1	1	1	[183 - 70] (REVERSE SENSE)
a_Nmar_UR_3_57	-	a_Nmar_UR_3_57	-	3.6e-05	15.4	0.0	3.6e-05	15.4	0.0	1.0	1	0	0	1	1	1	1	[39 - 10] (REVERSE SENSE)
m_Nmar_UR_3_57	-	a_Nmar_UR_3_57	-	9e-05	14.5	0.0	9e-05	14.5	0.0	1.0	1	0	0	1	1	1	1	[39 - 10] (REVERSE SENSE)
a_Nmar_UR_3_58	-	a_Nmar_UR_3_58	-	7.2e-15	47.0	2.0	7.4e-15	46.9	2.0	1.0	1	0	0	1	1	1	1	[70 - 2] (REVERSE SENSE)
m_Nmar_UR_3_58	-	a_Nmar_UR_3_58	-	7.2e-15	47.0	2.0	7.4e-15	46.9	2.0	1.0	1	0	0	1	1	1	1	[70 - 2] (REVERSE SENSE)
a_Nmar_UR_14_1	-	a_Nmar_UR_14_1	-	4.5e-08	24.7	2.8	4.5e-08	24.7	2.8	1.0	1	0	0	1	1	1	1	[9 - 47]
m_Nmar_UR_14_1	-	a_Nmar_UR_14_1	-	4.5e-08	24.7	2.8	4.5e-08	24.7	2.8	1.0	1	0	0	1	1	1	1	[9 - 47]
a_Nmar_UR_14_2	-	a_Nmar_UR_14_2	-	2.8e-14	44.4	4.9	2.9e-14	44.4	4.9	1.0	1	0	0	1	1	1	1	[5 - 79]
m_Nmar_UR_14_2	-	a_Nmar_UR_14_2	-	2.8e-14	44.4	4.9	2.9e-14	44.4	4.9	1.0	1	0	0	1	1	1	1	[5 - 79]
a_Nmar_UR_14_3	-	a_Nmar_UR_14_3	-	4.6e-09	28.3	3.6	4.7e-09	28.3	3.6	1.0	1	0	0	1	1	1	1	[78 - 25] (REVERSE SENSE)
m_Nmar_UR_14_3	-	a_Nmar_UR_14_3	-	8e-09	27.6	3.4	8.1e-09	27.6	3.4	1.0	1	0	0	1	1	1	1	[78 - 25] (REVERSE SENSE)
a_Nmar_UR_14_4	-	a_Nmar_UR_14_4	-	1.1e-06	20.5	1.0	1.1e-06	20.5	1.0	1.0	1	0	0	1	1	1	1	[35 - 3] (REVERSE SENSE)
m_Nmar_UR_14_4	-	a_Nmar_UR_14_4	-	1.1e-06	20.5	1.0	1.1e-06	20.5	1.0	1.0	1	0	0	1	1	1	1	[35 - 3] (REVERSE SENSE)
a_Nmar_UR_14_5	-	a_Nmar_UR_14_5	-	3.4e-08	25.8	2.4	3.4e-08	25.8	2.4	1.0	1	0	0	1	1	1	1	[43 - 2] (REVERSE SENSE)
m_Nmar_UR_14_5	-	a_Nmar_UR_14_5	-	6.5e-08	25.0	2.2	6.5e-08	25.0	2.2	1.0	1	0	0	1	1	1	1	[43 - 2] (REVERSE SENSE)
a_Nmar_UR_20_1	-	a_Nmar_UR_20_1	-	3.2e-08	24.0	0.5	3.2e-08	24.0	0.5	1.0	1	0	0	1	1	1	1	[14 - 58]
m_Nmar_UR_20_1	-	a_Nmar_UR_20_1	-	7.1e-08	23.1	0.5	7.1e-08	23.1	0.5	1.0	1	0	0	1	1	1	1	[14 - 58]
a_Nmar_UR_20_2	-	a_Nmar_UR_20_2	-	6.5e-11	34.9	3.3	6.7e-11	34.9	3.3	1.0	1	0	0	1	1	1	1	[57 - 1] (REVERSE SENSE)
m_Nmar_UR_20_2	-	a_Nmar_UR_20_2	-	1e-10	34.4	3.3	1e-10	34.3	3.3	1.0	1	0	0	1	1	1	1	[57 - 1] (REVERSE SENSE)
a_Nmar_UR_21_1	-	a_Nmar_UR_21_1	-	2.9e-05	14.9	0.1	2.9e-05	14.9	0.1	1.0	1	0	0	1	1	1	1	[10 - 39]
m_Nmar_UR_21_1	-	a_Nmar_UR_21_1	-	7.1e-05	14.0	0.1	7.1e-05	14.0	0.1	1.1	1	0	0	1	1	1	1	[10 - 39]
a_Nmar_UR_22_1	-	a_Nmar_UR_22_1	-	6.4e-05	14.4	2.2	6.4e-05	14.4	2.2	1.1	1	0	0	1	1	1	1	[6 - 35]
m_Nmar_UR_22_1	-	a_Nmar_UR_22_1	-	6.4e-05	14.4	2.2	6.4e-05	14.4	2.2	1.1	1	0	0	1	1	1	1	[6 - 35]
a_Nmar_UR_22_2	-	a_Nmar_UR_22_2	-	3.6e-07	21.6	0.7	3.6e-07	21.6	0.7	1.0	1	0	0	1	1	1	1	[8 - 40]
m_Nmar_UR_22_2	-	a_Nmar_UR_22_2	-	9.5e-07	20.6	0.8	9.5e-07	20.6	0.8	1.0	1	0	0	1	1	1	1	[8 - 40]
a_Nmar_UR_23_1	-	a_Nmar_UR_23_1	-	2.4e-08	25.5	1.7	2.4e-08	25.5	1.7	1.0	1	0	0	1	1	1	1	[11 - 52]
m_Nmar_UR_23_1	-	a_Nmar_UR_23_1	-	2.4e-08	25.5	1.7	2.4e-08	25.5	1.7	1.0	1	0	0	1	1	1	1	[11 - 52]
a_Nmar_UR_23_2	-	a_Nmar_UR_23_2	-	1.6e-12	38.3	0.8	1.7e-12	38.3	0.8	1.0	1	0	0	1	1	1	1	[39 - 95]
m_Nmar_UR_23_2	-	a_Nmar_UR_23_2	-	1.6e-12	38.3	0.8	1.7e-12	38.3	0.8	1.0	1	0	0	1	1	1	1	[39 - 95]
a_Nmar_UR_23_3	-	a_Nmar_UR_23_3	-	2e-09	29.7	0.6	2e-09	29.7	0.6	1.0	1	0	0	1	1	1	1	[95 - 45] (REVERSE SENSE)
m_Nmar_UR_23_3	-	a_Nmar_UR_23_3	-	3.9e-09	28.9	0.5	3.9e-09	28.9	0.5	1.0	1	0	0	1	1	1	1	[95 - 45] (REVERSE SENSE)
a_Nmar_UR_23_4	-	a_Nmar_UR_23_4	-	8.6e-09	27.0	2.0	8.6e-09	27.0	2.0	1.0	1	0	0	1	1	1	1	[42 - 1] (REVERSE SENSE)
m_Nmar_UR_23_4	-	a_Nmar_UR_23_4	-	8.6e-09	27.0	2.0	8.6e-09	27.0	2.0	1.0	1	0	0	1	1	1	1	[42 - 1] (REVERSE SENSE)
a_Nmar_UR_24_1	-	a_Nmar_UR_24_1	-	3.8e-06	18.3	0.2	3.8e-06	18.3	0.2	1.0	1	0	0	1	1	1	1	[24 - 56]
m_Nmar_UR_24_1	-	a_Nmar_UR_24_1	-	8.9e-06	17.4	0.2	8.9e-06	17.4	0.2	1.0	1	0	0	1	1	1	1	[24 - 56]
a_Nmar_UR_24_2	-	a_Nmar_UR_24_2	-	4.5e-07	20.5	0.3	4.5e-07	20.5	0.3	1.0	1	0	0	1	1	1	1	[28 - 63]
m_Nmar_UR_24_2	-	a_Nmar_UR_24_2	-	4.5e-07	20.5	0.3	4.5e-07	20.5	0.3	1.0	1	0	0	1	1	1	1	[28 - 63]
a_Atra_UR_2_1	-	a_Atra_UR_2_1	-	7.7e-07	21.1	0.5	7.7e-07	21.1	0.5	1.0	1	0	0	1	1	1	1	[1 - 33]
m_Atra_UR_2_1	-	a_Atra_UR_2_1	-	2.3e-06	19.9	0.8	2.3e-06	19.9	0.8	1.0	1	0	0	1	1	1	1	[1 - 33]
a_Atra_UR_2_2	-	a_Atra_UR_2_2	-	2.3e-06	20.4	0.8	2.3e-06	20.4	0.8	1.0	1	0	0	1	1	1	1	[32 - 3] (REVERSE SENSE)
m_Atra_UR_2_2	-	a_Atra_UR_2_2	-	2.3e-06	20.4	0.8	2.3e-06	20.4	0.8	1.0	1	0	0	1	1	1	1	[32 - 3] (REVERSE SENSE)
a_Atra_UR_3_1	-	a_Atra_UR_3_1	-	1.3e-08	26.5	0.5	1.3e-08	26.5	0.5	1.0	1	0	0	1	1	1	1	[68 - 24] (REVERSE SENSE)
m_Atra_UR_3_1	-	a_Atra_UR_3_1	-	2.2e-08	25.9	0.5	2.2e-08	25.9	0.5	1.0	1	0	0	1	1	1	1	[68 - 24] (REVERSE SENSE)
a_Atra_UR_13_1	-	a_Atra_UR_13_1	-	4.3e-06	17.5	0.3	4.3e-06	17.5	0.3	1.0	1	0	0	1	1	1	1	[2 - 31]
m_Atra_UR_13_1	-	a_Atra_UR_13_1	-	1.2e-05	16.5	0.3	1.2e-05	16.5	0.3	1.0	1	0	0	1	1	1	1	[2 - 31]
a_Atra_UR_13_2	-	a_Atra_UR_13_2	-	6.3e-09	28.4	3.6	6.3e-09	28.4	3.6	1.0	1	0	0	1	1	1	1	[37 - 78]
m_Atra_UR_13_2	-	a_Atra_UR_13_2	-	6.3e-09	28.4	3.6	6.3e-09	28.4	3.6	1.0	1	0	0	1	1	1	1	[37 - 78]
a_Atra_UR_13_3	-	a_Atra_UR_13_3	-	2.8e-16	51.8	4.6	2.9e-16	51.7	4.6	1.0	1	0	0	1	1	1	1	[27 - 104]
m_Atra_UR_13_3	-	a_Atra_UR_13_3	-	7.6e-16	50.5	4.9	7.9e-16	50.4	4.9	1.0	1	0	0	1	1	1	1	[27 - 104]
a_Atra_UR_13_4	-	a_Atra_UR_13_4	-	1e-07	24.6	3.6	1e-07	24.6	3.6	1.0	1	0	0	1	1	1	1	[115 - 150]
m_Atra_UR_13_4	-	a_Atra_UR_13_4	-	1e-07	24.6	3.6	1e-07	24.6	3.6	1.0	1	0	0	1	1	1	1	[115 - 150]
a_Atra_UR_13_5	-	a_Atra_UR_13_5	-	6.2e-24	76.8	5.4	6.7e-24	76.7	5.4	1.0	1	0	0	1	1	1	1	[41 - 154]
m_Atra_UR_13_5	-	a_Atra_UR_13_5	-	1e-23	76.1	5.2	1.1e-23	76.0	5.2	1.0	1	0	0	1	1	1	1	[41 - 154]
a_Atra_UR_13_6	-	a_Atra_UR_13_6	-	4e-09	27.9	2.0	4e-09	27.9	2.0	1.0	1	0	0	1	1	1	1	[153 - 200]
m_Atra_UR_13_6	-	a_Atra_UR_13_6	-	9.3e-09	26.9	2.1	9.3e-09	26.9	2.1	1.0	1	0	0	1	1	1	1	[153 - 200]
a_Atra_UR_13_7	-	a_Atra_UR_13_7	-	6.6e-11	34.3	3.0	6.7e-11	34.3	3.0	1.0	1	0	0	1	1	1	1	[242 - 189] (REVERSE SENSE)
m_Atra_UR_13_7	-	a_Atra_UR_13_7	-	1.4e-10	33.4	3.0	1.4e-10	33.4	3.0	1.0	1	0	0	1	1	1	1	[242 - 189] (REVERSE SENSE)
a_Atra_UR_13_8	-	a_Atra_UR_13_8	-	1e-05	17.7	1.5	1e-05	17.7	1.5	1.0	1	0	0	1	1	1	1	[182 - 153] (REVERSE SENSE)

m_Atra_UR_13_8	-	a_Atra_UR_13_8	-	2.4e-05	16.8	1.4	2.4e-05	16.8	1.4	1.0	1	0	0	1	1	1	1	[182 - 153] (REVERSE SENSE)
a_Atra_UR_13_9	-	a_Atra_UR_13_9	-	6.6e-14	42.1	1.6	6.9e-14	42.0	1.6	1.0	1	0	0	1	1	1	1	[147 - 73] (REVERSE SENSE)
m_Atra_UR_13_9	-	a_Atra_UR_13_9	-	6.6e-14	42.1	1.6	6.9e-14	42.0	1.6	1.0	1	0	0	1	1	1	1	[147 - 73] (REVERSE SENSE)
a_Atra_UR_13_10	-	a_Atra_UR_13_10	-	4e-33	106.5	6.0	4.4e-33	106.4	6.0	1.0	1	0	0	1	1	1	1	[223 - 62] (REVERSE SENSE)
m_Atra_UR_13_10	-	a_Atra_UR_13_10	-	6.3e-33	105.9	5.7	6.7e-33	105.8	5.7	1.0	1	0	0	1	1	1	1	[223 - 62] (REVERSE SENSE)
a_Atra_UR_13_11	-	a_Atra_UR_13_11	-	1e-07	22.5	0.4	1e-07	22.5	0.4	1.0	1	0	0	1	1	1	1	[69 - 34] (REVERSE SENSE)
m_Atra_UR_13_11	-	a_Atra_UR_13_11	-	1e-07	22.5	0.4	1e-07	22.5	0.4	1.0	1	0	0	1	1	1	1	[69 - 34] (REVERSE SENSE)
a_Atra_UR_13_12	-	a_Atra_UR_13_12	-	3.6e-08	25.7	2.6	3.6e-08	25.7	2.6	1.0	1	0	0	1	1	1	1	[50 - 15] (REVERSE SENSE)
m_Atra_UR_13_12	-	a_Atra_UR_13_12	-	3.6e-08	25.7	2.6	3.6e-08	25.7	2.6	1.0	1	0	0	1	1	1	1	[50 - 15] (REVERSE SENSE)
a_Atra_UR_14_1	-	a_Atra_UR_14_1	-	1.5e-11	35.5	0.4	1.6e-11	35.4	0.4	1.0	1	0	0	1	1	1	1	[11 - 64]
m_Atra_UR_14_1	-	a_Atra_UR_14_1	-	3.2e-11	34.6	0.4	3.2e-11	34.6	0.4	1.0	1	0	0	1	1	1	1	[11 - 64]
a_Atra_UR_14_2	-	a_Atra_UR_14_2	-	2.5e-08	25.2	1.1	2.5e-08	25.2	1.1	1.0	1	0	0	1	1	1	1	[37 - 84]
m_Atra_UR_14_2	-	a_Atra_UR_14_2	-	5.3e-08	24.4	1.0	5.3e-08	24.4	1.0	1.0	1	0	0	1	1	1	1	[37 - 84]
a_Atra_UR_14_3	-	a_Atra_UR_14_3	-	9.2e-06	18.5	3.0	9.2e-06	18.5	3.0	1.0	1	0	0	1	1	1	1	[96 - 67] (REVERSE SENSE)
m_Atra_UR_14_3	-	a_Atra_UR_14_3	-	1.6e-05	17.9	2.6	1.6e-05	17.9	2.6	1.0	1	0	0	1	1	1	1	[96 - 67] (REVERSE SENSE)
a_Atra_UR_14_4	-	a_Atra_UR_14_4	-	3.9e-08	26.4	4.3	3.9e-08	26.4	4.3	1.0	1	0	0	1	1	1	1	[94 - 50] (REVERSE SENSE)
m_Atra_UR_14_4	-	a_Atra_UR_14_4	-	6.5e-08	25.8	4.0	6.5e-08	25.8	4.0	1.0	1	0	0	1	1	1	1	[94 - 50] (REVERSE SENSE)
a_Atra_UR_14_5	-	a_Atra_UR_14_5	-	2.5e-05	14.9	0.2	2.5e-05	14.9	0.2	1.0	1	0	0	1	1	1	1	[53 - 24] (REVERSE SENSE)
m_Atra_UR_14_5	-	a_Atra_UR_14_5	-	6.4e-05	13.9	0.2	6.4e-05	13.9	0.2	1.1	1	0	0	1	1	1	1	[53 - 24] (REVERSE SENSE)
a_Atra_UR_21_1	-	a_Atra_UR_21_1	-	7.8e-10	31.3	1.1	7.8e-10	31.3	1.1	1.0	1	0	0	1	1	1	1	[16 - 69]
m_Atra_UR_21_1	-	a_Atra_UR_21_1	-	1.6e-09	30.4	1.1	1.6e-09	30.4	1.1	1.0	1	0	0	1	1	1	1	[16 - 69]
a_Atra_UR_21_2	-	a_Atra_UR_21_2	-	3.5e-11	35.1	0.8	3.5e-11	35.0	0.8	1.0	1	0	0	1	1	1	1	[41 - 94]
m_Atra_UR_21_2	-	a_Atra_UR_21_2	-	5.8e-11	34.4	0.8	5.9e-11	34.4	0.8	1.0	1	0	0	1	1	1	1	[41 - 94]
a_Atra_UR_21_3	-	a_Atra_UR_21_3	-	6.2e-07	21.8	1.1	6.2e-07	21.8	1.1	1.0	1	0	0	1	1	1	1	[66 - 98]
m_Atra_UR_21_3	-	a_Atra_UR_21_3	-	1.6e-06	20.7	1.3	1.6e-06	20.7	1.3	1.0	1	0	0	1	1	1	1	[66 - 98]
a_Atra_UR_21_4	-	a_Atra_UR_21_4	-	7e-07	20.1	0.2	7e-07	20.1	0.2	1.0	1	0	0	1	1	1	1	[102 - 137]
m_Atra_UR_21_4	-	a_Atra_UR_21_4	-	1.8e-06	19.1	0.3	1.8e-06	19.1	0.3	1.0	1	0	0	1	1	1	1	[102 - 137]
a_Atra_UR_21_5	-	a_Atra_UR_21_5	-	3.1e-11	34.7	2.2	3.1e-11	34.7	2.2	1.0	1	0	0	1	1	1	1	[137 - 187]
m_Atra_UR_21_5	-	a_Atra_UR_21_5	-	3.1e-11	34.7	2.2	3.1e-11	34.7	2.2	1.0	1	0	0	1	1	1	1	[137 - 187]
a_Atra_UR_21_6	-	a_Atra_UR_21_6	-	6.3e-28	89.2	5.4	6.8e-28	89.1	5.4	1.0	1	0	0	1	1	1	1	[73 - 213]
m_Atra_UR_21_6	-	a_Atra_UR_21_6	-	1.3e-27	88.2	5.6	1.4e-27	88.1	5.6	1.0	1	0	0	1	1	1	1	[73 - 213]
a_Atra_UR_21_7	-	a_Atra_UR_21_7	-	6.2e-13	41.4	4.5	6.3e-13	41.3	4.5	1.0	1	0	0	1	1	1	1	[208 - 146] (REVERSE SENSE)
m_Atra_UR_21_7	-	a_Atra_UR_21_7	-	8.6e-13	40.9	4.3	8.7e-13	40.9	4.3	1.0	1	0	0	1	1	1	1	[208 - 146] (REVERSE SENSE)
a_Atra_UR_21_8	-	a_Atra_UR_21_8	-	2.3e-06	21.4	7.2	2.3e-06	21.4	7.2	1.0	1	0	0	1	1	1	1	[177 - 136] (REVERSE SENSE)
m_Atra_UR_21_8	-	a_Atra_UR_21_8	-	3.7e-06	20.9	7.1	3.7e-06	20.9	7.1	1.0	1	0	0	1	1	1	1	[177 - 136] (REVERSE SENSE)
a_Atra_UR_21_9	-	a_Atra_UR_21_9	-	2.6e-42	137.0	5.1	2.8e-42	136.9	5.1	1.0	1	0	0	1	1	1	1	[209 - 9] (REVERSE SENSE)
m_Atra_UR_21_9	-	a_Atra_UR_21_9	-	4.3e-42	136.3	5.0	4.7e-42	136.2	5.0	1.0	1	0	0	1	1	1	1	[209 - 9] (REVERSE SENSE)
a_Atra_UR_21_10	-	a_Atra_UR_21_10	-	2.1e-13	42.0	0.6	2.1e-13	41.9	0.6	1.0	1	0	0	1	1	1	1	[70 - 2] (REVERSE SENSE)
m_Atra_UR_21_10	-	a_Atra_UR_21_10	-	2.1e-13	42.0	0.6	2.1e-13	41.9	0.6	1.0	1	0	0	1	1	1	1	[70 - 2] (REVERSE SENSE)
a_Atra_UR_22_1	-	a_Atra_UR_22_1	-	7.2e-14	42.5	2.5	7.5e-14	42.4	2.5	1.0	1	0	0	1	1	1	1	[7 - 78]
m_Atra_UR_22_1	-	a_Atra_UR_22_1	-	7.2e-14	42.5	2.5	7.5e-14	42.4	2.5	1.0	1	0	0	1	1	1	1	[7 - 78]
a_Atra_UR_22_6	-	a_Atra_UR_22_1	-	3.5e-06	21.1	0.7	3.5e-06	21.1	0.7	1.1	1	0	0	1	1	1	1	[292 - 357]
m_Atra_UR_22_6	-	a_Atra_UR_22_1	-	3.5e-06	21.1	0.7	3.5e-06	21.1	0.7	1.1	1	0	0	1	1	1	1	[292 - 357]
a_Atra_UR_22_2	-	a_Atra_UR_22_2	-	2.8e-15	47.7	4.8	2.9e-15	47.6	4.8	1.0	1	0	0	1	1	1	1	[9 - 86]
m_Atra_UR_22_2	-	a_Atra_UR_22_2	-	4.7e-15	47.0	4.5	4.9e-15	47.0	4.5	1.0	1	0	0	1	1	1	1	[9 - 86]
m_Atra_UR_22_7	-	a_Atra_UR_22_2	-	3e-06	21.8	0.6	3e-06	21.8	0.6	1.0	1	0	0	1	1	1	1	[318 - 365]
a_Atra_UR_22_7	-	a_Atra_UR_22_2	-	3.2e-06	21.7	0.6	3.2e-06	21.7	0.6	1.0	1	0	0	1	1	1	1	[318 - 365]
a_Atra_UR_22_3	-	a_Atra_UR_22_3	-	8e-18	55.2	2.7	8.4e-18	55.1	2.7	1.0	1	0	0	1	1	1	1	[111 - 197]
m_Atra_UR_22_3	-	a_Atra_UR_22_3	-	2.7e-17	53.7	3.3	2.8e-17	53.6	3.3	1.0	1	0	0	1	1	1	1	[111 - 197]
a_Atra_UR_22_4	-	a_Atra_UR_22_4	-	1.1e-38	124.6	10.6	1.2e-38	124.5	10.6	1.0	1	0	0	1	1	1	1	[35 - 220]
m_Atra_UR_22_4	-	a_Atra_UR_22_4	-	2.8e-38	123.3	11.0	3e-38	123.2	11.0	1.0	1	0	0	1	1	1	1	[35 - 220]
m_Atra_UR_22_8	-	a_Atra_UR_22_4	-	3.9e-05	19.6	0.7	4.9e-05	19.3	0.7	1.2	1	0	0	1	1	1	1	[269 - 382]
a_Atra_UR_22_8	-	a_Atra_UR_22_4	-	3.9e-05	19.6	0.7	4.9e-05	19.3	0.7	1.2	1	0	0	1	1	1	1	[269 - 382]
a_Atra_UR_22_5	-	a_Atra_UR_22_5	-	1.2e-12	38.9	1.1	1.2e-12	38.8	1.1	1.0	1	0	0	1	1	1	1	[213 - 275]
m_Atra_UR_22_5	-	a_Atra_UR_22_5	-	1.2e-12	38.9	1.1	1.2e-12	38.8	1.1	1.0	1	0	0	1	1	1	1	[213 - 275]
a_Atra_UR_22_6	-	a_Atra_UR_22_6	-	3.1e-13	40.9	2.5	3.2e-13	40.9	2.5	1.0	1	0	0	1	1	1	1	[292 - 357]
m_Atra_UR_22_6	-	a_Atra_UR_22_6	-	3.1e-13	40.9	2.5	3.2e-13	40.9	2.5	1.0	1	0	0	1	1	1	1	[292 - 357]
a_Atra_UR_22_1	-	a_Atra_UR_22_6	-	4.5e-06	20.9	1.1	4.6e-06	20.9	1.1	1.2	1	0	0	1	1	1	1	[7 - 78]
m_Atra_UR_22_1	-	a_Atra_UR_22_6	-	4.5e-06	20.9	1.1	4.6e-06	20.9	1.1	1.2	1	0	0	1	1	1	1	[7 - 78]
a_Atra_UR_22_7	-	a_Atra_UR_22_7	-	3.1e-10	31.0	1.7	3.1e-10	31.0	1.7	1.0	1	0	0	1	1	1	1	[318 - 365]
m_Atra_UR_22_7	-	a_Atra_UR_22_7	-	9.1e-10	29.7	1.9	9.1e-10	29.7	1.9	1.0	1	0	0	1	1	1	1	[318 - 365]
m_Atra_UR_22_2	-	a_Atra_UR_22_7	-	1.4e-06	21.2	1.5	2.4e-06	20.6	0.9	1.6	1	1	0	1	1	1	1	[9 - 86]
a_Atra_UR_22_2	-	a_Atra_UR_22_7	-	1.4e-06	21.2	1.5	2.4e-06	20.6	0.9	1.6	1	1	0	1	1	1	1	[9 - 86]
a_Atra_UR_22_8	-	a_Atra_UR_22_8	-	1.9e-25	81.5	4.0	2e-25	81.4	4.0	1.0	1	0	0	1	1	1	1	[269 - 382]
m_Atra_UR_22_8	-	a_Atra_UR_22_8	-	4.3e-25	80.4	4.2	4.5e-25	80.4	4.2	1.0	1	0	0	1	1	1	1	[269 - 382]
a_Atra_UR_22_4	-	a_Atra_UR_22_8	-	5.7e-05	19.1	0.7	5.7e-05	19.1	0.7	2.0	2	1	0	2	2	2	1	[35 - 220]
m_Atra_UR_22_4	-	a_Atra_UR_22_8	-	9.7e-05	18.4	0.8	9.7e-05	18.4	0.8	2.0	2	1	0	2	2	2	1	[35 - 220]
a_Atra_UR_22_9	-	a_Atra_UR_22_9	-	2.1e-07	22.3	0.1	2.1e-07	22.3	0.1	1.0	1	0	0	1	1	1	1	[398 - 436]
m_Atra_UR_22_9	-	a_Atra_UR_22_9	-	3.6e-07	21.7	0.1	3.6e-07	21.7	0.1	1.0	1	0	0	1	1	1	1	[398 - 436]
a_Atra_UR_22_10	-	a_Atra_UR_22_10	-	4.4e-14	44.0	0.5	4.5e-14	44.0	0.5	1.0	1	0	0	1	1	1	1	[385 - 453]
m_Atra_UR_22_10	-	a_Atra_UR_22_10	-	8.8e-14	43.2	0.5	8.9e-14	43.2	0.5	1.0	1	0	0	1	1	1	1	[385 - 453]
a_Atra_UR_22_11	-	a_Atra_UR_22_11	-	1.6e-08	26.2	1.4	1.6e-08	26.2	1.4	1.0	1	0	0	1	1	1	1	[429 - 391] (REVERSE SENSE)
m_Atra_UR_22_11	-	a_Atra_UR_22_11	-	1.6e-08	26.2	1.4	1.6e-08	26.2	1.4	1.0	1	0	0	1	1	1	1	[429 - 391] (REVERSE SENSE)
a_Atra_UR_22_12	-	a_Atra_UR_22_12	-	2.1e-21	68.9	6.8	2.2e-21	68.8	6.8	1.0	1	0	0	1	1	1	1	[427 - 323] (REVERSE SENSE)
m_Atra_UR_22_12	-	a_Atra_UR_22_12	-	2.1e-21	68.9	6.8	2.2e-21	68.8	6.8	1.0	1	0	0	1	1	1	1	[427 - 323] (REVERSE SENSE)
a_Atra_UR_22_21	-	a_Atra_UR_22_12	-	0.00013	18.1	3.6	0.00013	18.1	3.6	1.0	1	0	0	1	1	1	1	[94 - 47] (REVERSE SENSE)
m_Atra_UR_22_21	-	a_Atra_UR_22_12	-	0.00018	17.7	3.4	0.00018	17.7	3.4	1.0	1	0	0	1	1	1	1	[94 - 47] (REVERSE SENSE)
a_Atra_UR_22_13	-	a_Atra_UR_22_13	-	7.8e-14	44.5	2.3	8.1e-14	44.5	2.3	1.0	1	0	0	1	1	1	1	[369 - 304] (REVERSE SENSE)
m_Atra_UR_22_13	-	a_Atra_UR_22_13	-	1.5e-13	43.6	2.2	1.6e-13	43.6	2.2	1.0	1	0	0	1	1	1	1	[369 - 304] (REVERSE SENSE)
a_Atra_UR_22_22	-	a_Atra_UR_22_13	-	7.2e-05	18.4	0.8	8e-05	18.2	0.8	1.2	1	0	0	1	1	1	1	[99 - 25] (REVERSE SENSE)
m_Atra_UR_22_22	-	a_Atra_UR_22_13	-	7.2e-05	18.4	0.8	8e-05	18.2	0.8	1.2	1	0	0	1	1	1	1	[99 - 25] (REVERSE SENSE)
a_Atra_UR_22_14	-	a_Atra_UR_22_14	-	1.6e-20	65.8	5.3	1.7e-20	65.7	5.3	1.0	1	0	0	1	1	1	1	[356 - 261] (REVERSE SENSE)
m_Atra_UR_22_14	-	a_Atra_UR_22_14	-	3e-20	64.9	5.2	3.2e-20	64.9	5.2	1.0	1	0	0	1	1	1	1	[356 - 261] (REVERSE SENSE)
a_Atra_UR_22_15	-	a_Atra_UR_22_15	-	3e-07	23.1	1.0	3e-07	23.1	1.0	1.0	1	0	0	1	1	1	1	[270 - 238] (REVERSE SENSE)
m_Atra_UR_22_15	-	a_Atra_UR_22_15	-	3e-07	23.1	1.0	3e-07	23.1	1.0	1.0	1	0	0	1	1	1	1	[270 - 238] (REVERSE SENSE)
a_Atra_UR_22_16	-	a_Atra_UR_22_16	-	9.6e-07	21.8	2.2	9.6e-07	21.8	2.2	1.0	1	0	0	1	1	1	1	[256 - 212] (REVERSE SENSE)
m_Atra_UR_22_16	-	a_Atra_UR_22_16	-	1.4e-06	21.4	1.7	1.4e-06	21.4	1.7	1.0	1	0	0	1	1	1	1	[256 - 212] (REVERSE SENSE)
a_Atra_UR_22_17	-	a_Atra_UR_22_17	-	1e-08	27.0	0.7	1e-08	27.0	0.7	1.0	1	0	0	1	1	1	1	[242 - 198] (REVERSE SENSE)
m_Atra_UR_22_17	-	a_Atra_UR_22_17	-	2e-08	26.3	0.5	2e-08	26.3	0.5	1.0	1	0	0	1	1	1	1	[242 - 198] (REVERSE SENSE)
a_Atra_UR_22_18	-	a_Atra_UR_22_18	-	1.9e-12	40.0	5.3	1.9e-12	40.0	5.3	1.0	1	0	0	1	1	1	1	[198 - 133] (REVERSE SENSE)
m_Atra_UR_22_18	-	a_Atra_UR_22_18	-	1.9e-12	40.0	5.3	1.9e-12	40.0	5.3	1.0	1	0	0	1	1	1	1	[198 - 133] (REVERSE SENSE)
a_Atra_UR_22_19	-	a_Atra_UR_22_19	-	4.8e-13	42.4	6.6	4.9e-13	42.4	6.6	1.0	1	0	0	1	1	1	1	[194 - 129] (REVERSE SENSE)
m_Atra_UR_22_19	-	a_Atra_UR_22_19	-	6.4e-13	42.0	6.3	6.5e-13	42.0	6.3	1.0	1	0	0	1	1	1	1	[194 - 129] (REVERSE SENSE)
a_Atra_UR_22_20	-	a_Atra_UR_22_20	-	5e-19	62.1	15.6	5.3e-19	62.0	15.6	1.0	1	0	0	1	1	1	1	[205 - 110] (REVERSE SENSE)
m_Atra_UR_22_20	-	a_Atra_UR_22_20	-	6.5e-19	61.8	15.3	6.9e-19	61.7	15.3	1.0	1	0	0	1	1	1	1	[205 - 110] (REVERSE SENSE)
a_Atra_UR_22_21	-	a_Atra_UR_22_21	-	1e-08	28.2	4.9	1e-08	28.2	4.9	1.0	1	0	0	1	1	1	1	[94 - 47] (REVERSE SENSE)
m_Atra_UR_22_21	-	a_Atra_UR_22_21	-	1.5e-08	27.8	4.4	1.5e-08	27.8	4.4	1.0	1	0	0	1	1	1	1	[94 - 47] (REVERSE SENSE)
a_Atra_UR_22_12	-	a_Atra_UR_22_21	-	8.5e-05	17.4	3.0	8.5e-05	17.4	3.0	1.7	2	1	0	2	2	2	1	[427 - 323] (REVERSE SENSE)
m_Atra_UR_22_12	-	a_Atra_UR_22_21	-	8.5e-05	17.4	3.0	8.5e-05	17.4	3.0	1.7	2	1	0	2	2	2	1	[427 - 323] (REVERSE SENSE)
a_Atra_UR_22_22	-	a_Atra_UR_22_22	-	3.9e-15	47.9	3.0	4.1e-15	47.8	3.0	1.0	1	0	0	1	1	1	1	[99 - 25] (REVERSE SENSE)
m_Atra_UR_22_22	-	a_Atra_UR_22_22	-	3.9e-15	47.9	3.0	4.1e-15	47.8	3.0	1.0	1	0	0	1	1	1	1	[99 - 25] (REVERSE SENSE)
a_Atra_UR_22_13	-	a_Atra_UR_22_22	-	4.4e-05	18.6	0.6	4.4e-05	18.6	0.6	1.1	1	0	0	1	1	1	1	[369 - 304] (REVERSE SENSE)
m_Atra_UR_22_13	-	a_Atra_UR_22_22	-	8.1e-05	17.9	0.6	8.1e-05	17.9	0.6	1.1	1	0	0	1	1	1	1	[369 - 304] (REVERSE SENSE)
a_Atra_UR_22_23	-	a_Atra_UR_22_23	-	4e-12	38.5	1.6	4.1e-12	38.5	1.6	1.0	1	0	0	1	1	1	1	[62 - 3] (REVERSE SENSE)
m_Atra_UR_22_23	-	a_Atra_UR_22_23	-	6.6e-12	37.9	1.6	6.7e-12	37.9	1.6	1.0	1	0	0	1	1	1	1	[62 - 3] (REVERSE SENSE)
a_Atra_UR_23_1	-	a_Atra_UR_23_1	-	2.3e-06	19.1	2.6	2.3e-06	19.1	2.6	1.0	1	0	0	1	1	1	1	[24 - 56]
m_Atra_UR_23_1	-	a_Atra_UR_23_1	-	2.3e-06	19.1	2.6	2.3e-06	19.1	2.6	1.0	1	0	0	1	1	1	1	[24 - 56]
a_Atra_UR_23_2	-	a_Atra_UR_23_2	-	4.6e-13	40.6	2.4	4.8e-13	40.6	2.4	1.0	1	0	0	1	1	1	1	[22 - 87]
m_Atra_UR_23_2	-	a_Atra_UR_23_2	-	4.6e-13	40.6	2.4	4.8e-13	40.6	2.4	1.0	1	0	0	1	1	1	1	[22 - 87]

a_Atra_UR_23_3	-	a_Atra_UR_23_3	-	4.7e-07	21.9	2.2	4.7e-07	21.9	2.2	1.0	1	0	0	1	1	1	1	[51 - 16] (REVERSE SENSE)
m_Atra_UR_23_3	-	a_Atra_UR_23_3	-	1.1e-06	20.8	2.3	1.1e-06	20.8	2.3	1.0	1	0	0	1	1	1	1	[51 - 16] (REVERSE SENSE)
a_Atra_UR_23_4	-	a_Atra_UR_23_4	-	8.4e-17	53.9	6.2	8.8e-17	53.8	6.2	1.0	1	0	0	1	1	1	1	[80 - 3] (REVERSE SENSE)
m_Atra_UR_23_4	-	a_Atra_UR_23_4	-	1.3e-16	53.3	5.8	1.3e-16	53.3	5.8	1.0	1	0	0	1	1	1	1	[80 - 3] (REVERSE SENSE)
a_Atra_UR_23_5	-	a_Atra_UR_23_5	-	5.5e-05	14.3	0.1	5.5e-05	14.3	0.1	1.0	1	0	0	1	1	1	1	[31 - 2] (REVERSE SENSE)
m_Atra_UR_23_5	-	a_Atra_UR_23_5	-	0.00015	13.3	0.1	0.00015	13.3	0.1	1.1	1	0	0	1	1	1	1	[31 - 2] (REVERSE SENSE)
a_Mdub_UR_4_1	-	a_Mdub_UR_4_1	-	9.2e-06	17.9	0.9	9.2e-06	17.9	0.9	1.0	1	0	0	1	1	1	1	[16 - 45]
m_Mdub_UR_4_1	-	a_Mdub_UR_4_1	-	2.2e-05	17.0	0.9	2.2e-05	17.0	0.9	1.0	1	0	0	1	1	1	1	[16 - 45]
a_Mdub_UR_4_2	-	a_Mdub_UR_4_2	-	2.5e-20	64.5	4.8	2.7e-20	64.4	4.8	1.0	1	0	0	1	1	1	1	[9 - 116]
m_Mdub_UR_4_2	-	a_Mdub_UR_4_2	-	5.5e-20	63.4	5.0	5.8e-20	63.4	5.0	1.0	1	0	0	1	1	1	1	[9 - 116]
a_Mdub_UR_4_3	-	a_Mdub_UR_4_3	-	2.1e-05	16.3	0.4	2.1e-05	16.3	0.4	1.0	1	0	0	1	1	1	1	[77 - 48] (REVERSE SENSE)
m_Mdub_UR_4_3	-	a_Mdub_UR_4_3	-	5.2e-05	15.3	0.4	5.2e-05	15.3	0.4	1.0	1	0	0	1	1	1	1	[77 - 48] (REVERSE SENSE)
a_Mdub_UR_4_4	-	a_Mdub_UR_4_4	-	1e-14	47.1	4.3	1e-14	47.0	4.3	1.0	1	0	0	1	1	1	1	[115 - 41] (REVERSE SENSE)
m_Mdub_UR_4_4	-	a_Mdub_UR_4_4	-	1.3e-14	46.7	3.7	1.3e-14	46.7	3.7	1.0	1	0	0	1	1	1	1	[115 - 41] (REVERSE SENSE)
a_Mdub_UR_15_1	-	a_Mdub_UR_15_1	-	8.6e-06	17.5	1.2	8.6e-06	17.5	1.2	1.0	1	0	0	1	1	1	1	[26 - 64]
m_Mdub_UR_15_1	-	a_Mdub_UR_15_1	-	1.9e-05	16.7	1.1	1.9e-05	16.7	1.1	1.0	1	0	0	1	1	1	1	[26 - 64]
a_Mdub_UR_15_2	-	a_Mdub_UR_15_2	-	3e-07	20.5	0.1	3e-07	20.5	0.1	1.0	1	0	0	1	1	1	1	[75 - 110]
m_Mdub_UR_15_2	-	a_Mdub_UR_15_2	-	7.3e-07	19.6	0.1	7.3e-07	19.6	0.1	1.0	1	0	0	1	1	1	1	[75 - 110]
a_Mdub_UR_15_3	-	a_Mdub_UR_15_3	-	1.6e-06	18.7	0.4	1.6e-06	18.7	0.4	1.0	1	0	0	1	1	1	1	[95 - 130]
m_Mdub_UR_15_3	-	a_Mdub_UR_15_3	-	1.6e-06	18.7	0.4	1.6e-06	18.7	0.4	1.0	1	0	0	1	1	1	1	[95 - 130]
a_Mdub_UR_15_4	-	a_Mdub_UR_15_4	-	3e-05	16.3	0.9	3e-05	16.3	0.9	1.0	1	0	0	1	1	1	1	[134 - 163]
m_Mdub_UR_15_4	-	a_Mdub_UR_15_4	-	7.4e-05	15.3	1.0	7.4e-05	15.3	1.0	1.1	1	0	0	1	1	1	1	[134 - 163]
a_Mdub_UR_15_5	-	a_Mdub_UR_15_5	-	3.2e-31	100.0	2.5	3.4e-31	99.9	2.5	1.0	1	0	0	1	1	1	1	[88 - 231]
m_Mdub_UR_15_5	-	a_Mdub_UR_15_5	-	3.2e-31	100.0	2.5	3.4e-31	99.9	2.5	1.0	1	0	0	1	1	1	1	[88 - 231]
a_Mdub_UR_15_6	-	a_Mdub_UR_15_6	-	5.9e-10	30.5	0.4	5.9e-10	30.5	0.4	1.0	1	0	0	1	1	1	1	[227 - 274]
m_Mdub_UR_15_6	-	a_Mdub_UR_15_6	-	1.3e-09	29.6	0.4	1.3e-09	29.6	0.4	1.0	1	0	0	1	1	1	1	[227 - 274]
a_Mdub_UR_15_7	-	a_Mdub_UR_15_7	-	2.9e-17	54.3	0.1	3.1e-17	54.2	0.1	1.0	1	0	0	1	1	1	1	[238 - 318]
m_Mdub_UR_15_7	-	a_Mdub_UR_15_7	-	5e-17	53.6	0.1	5.2e-17	53.6	0.1	1.0	1	0	0	1	1	1	1	[238 - 318]
a_Mdub_UR_15_8	-	a_Mdub_UR_15_8	-	2.1e-18	58.6	1.3	2.2e-18	58.5	1.3	1.0	1	0	0	1	1	1	1	[358 - 444]
m_Mdub_UR_15_8	-	a_Mdub_UR_15_8	-	4e-18	57.7	1.3	4.2e-18	57.7	1.3	1.0	1	0	0	1	1	1	1	[358 - 444]
a_Mdub_UR_15_9	-	a_Mdub_UR_15_9	-	2e-40	130.8	4.9	2.1e-40	130.7	4.9	1.0	1	0	0	1	1	1	1	[299 - 466]
m_Mdub_UR_15_9	-	a_Mdub_UR_15_9	-	2e-40	130.8	4.9	2.1e-40	130.7	4.9	1.0	1	0	0	1	1	1	1	[299 - 466]
a_Mdub_UR_15_10	-	a_Mdub_UR_15_10	-	1.2e-58	190.9	9.7	1.3e-58	190.7	9.7	1.0	1	0	0	1	1	1	1	[234 - 485]
m_Mdub_UR_15_10	-	a_Mdub_UR_15_10	-	2.2e-58	190.0	9.6	2.4e-58	189.9	9.6	1.0	1	0	0	1	1	1	1	[234 - 485]
a_Mdub_UR_15_11	-	a_Mdub_UR_15_11	-	3.8e-20	64.8	3.1	4e-20	64.7	3.1	1.0	1	0	0	1	1	1	1	[539 - 625]
m_Mdub_UR_15_11	-	a_Mdub_UR_15_11	-	6.8e-20	64.0	2.9	7.1e-20	63.9	2.9	1.0	1	0	0	1	1	1	1	[539 - 625]
a_Mdub_UR_15_12	-	a_Mdub_UR_15_12	-	7.9e-36	115.9	4.1	8.6e-36	115.7	4.1	1.0	1	0	0	1	1	1	1	[514 - 669]
m_Mdub_UR_15_12	-	a_Mdub_UR_15_12	-	7.9e-36	115.9	4.1	8.6e-36	115.7	4.1	1.0	1	0	0	1	1	1	1	[514 - 669]
a_Mdub_UR_15_13	-	a_Mdub_UR_15_13	-	2.6e-06	19.3	0.3	2.6e-06	19.3	0.3	1.0	1	0	0	1	1	1	1	[716 - 745]
m_Mdub_UR_15_13	-	a_Mdub_UR_15_13	-	2.6e-06	19.3	0.3	2.6e-06	19.3	0.3	1.0	1	0	0	1	1	1	1	[716 - 745]
a_Mdub_UR_15_14	-	a_Mdub_UR_15_14	-	6.7e-08	23.9	0.4	6.7e-08	23.9	0.4	1.0	1	0	0	1	1	1	1	[752 - 790]
m_Mdub_UR_15_14	-	a_Mdub_UR_15_14	-	1.6e-07	22.9	0.5	1.6e-07	22.9	0.5	1.0	1	0	0	1	1	1	1	[752 - 790]
a_Mdub_UR_15_15	-	a_Mdub_UR_15_15	-	9.2e-60	194.7	10.5	1e-59	194.6	10.5	1.0	1	0	0	1	1	1	1	[537 - 797]
m_Mdub_UR_15_15	-	a_Mdub_UR_15_15	-	1.9e-59	193.7	10.6	2.1e-59	193.5	10.6	1.0	1	0	0	1	1	1	1	[537 - 797]
a_Mdub_UR_15_16	-	a_Mdub_UR_15_16	-	2.5e-06	19.6	1.2	2.5e-06	19.6	1.2	1.0	1	0	0	1	1	1	1	[807 - 839]
m_Mdub_UR_15_16	-	a_Mdub_UR_15_16	-	6e-06	18.6	1.2	6e-06	18.6	1.2	1.0	1	0	0	1	1	1	1	[807 - 839]
a_Mdub_UR_15_17	-	a_Mdub_UR_15_17	-	3.1e-48	155.9	2.9	3.4e-48	155.8	2.9	1.0	1	0	0	1	1	1	1	[691 - 897]
m_Mdub_UR_15_17	-	a_Mdub_UR_15_17	-	5e-48	155.3	3.0	5.4e-48	155.2	3.0	1.0	1	0	0	1	1	1	1	[691 - 897]
a_Mdub_UR_15_18	-	a_Mdub_UR_15_18	-	9.7e-10	29.7	1.1	9.8e-10	29.7	1.1	1.0	1	0	0	1	1	1	1	[858 - 905]
m_Mdub_UR_15_18	-	a_Mdub_UR_15_18	-	2.1e-09	28.8	1.0	2.1e-09	28.8	1.0	1.0	1	0	0	1	1	1	1	[858 - 905]
a_Mdub_UR_15_19	-	a_Mdub_UR_15_19	-	1e-09	29.9	2.5	1e-09	29.9	2.5	1.0	1	0	0	1	1	1	1	[893 - 846] (REVERSE SENSE)
m_Mdub_UR_15_19	-	a_Mdub_UR_15_19	-	2.3e-09	29.0	2.5	2.3e-09	29.0	2.5	1.0	1	0	0	1	1	1	1	[893 - 846] (REVERSE SENSE)
a_Mdub_UR_15_20	-	a_Mdub_UR_15_20	-	5.2e-12	37.4	1.0	5.2e-12	37.4	1.0	1.0	1	0	0	1	1	1	1	[846 - 793] (REVERSE SENSE)
m_Mdub_UR_15_20	-	a_Mdub_UR_15_20	-	5.2e-12	37.4	1.0	5.2e-12	37.4	1.0	1.0	1	0	0	1	1	1	1	[846 - 793] (REVERSE SENSE)
a_Mdub_UR_15_21	-	a_Mdub_UR_15_21	-	5.7e-14	43.8	0.2	5.9e-14	43.8	0.2	1.0	1	0	0	1	1	1	1	[824 - 756] (REVERSE SENSE)
m_Mdub_UR_15_21	-	a_Mdub_UR_15_21	-	1.1e-13	43.0	0.1	1.2e-13	43.0	0.1	1.0	1	0	0	1	1	1	1	[824 - 756] (REVERSE SENSE)
a_Mdub_UR_15_22	-	a_Mdub_UR_15_22	-	3.6e-09	27.6	0.3	3.6e-09	27.6	0.3	1.0	1	0	0	1	1	1	1	[766 - 725] (REVERSE SENSE)
m_Mdub_UR_15_22	-	a_Mdub_UR_15_22	-	3.6e-09	27.6	0.3	3.6e-09	27.6	0.3	1.0	1	0	0	1	1	1	1	[766 - 725] (REVERSE SENSE)
a_Mdub_UR_15_23	-	a_Mdub_UR_15_23	-	1.2e-15	48.4	0.4	1.3e-15	48.3	0.4	1.0	1	0	0	1	1	1	1	[762 - 685] (REVERSE SENSE)
m_Mdub_UR_15_23	-	a_Mdub_UR_15_23	-	3e-15	47.3	0.7	3e-15	47.3	0.7	1.0	1	0	0	1	1	1	1	[762 - 685] (REVERSE SENSE)
a_Mdub_UR_15_24	-	a_Mdub_UR_15_24	-	1.3e-29	95.2	2.6	1.4e-29	95.1	2.6	1.0	1	0	0	1	1	1	1	[641 - 510] (REVERSE SENSE)
m_Mdub_UR_15_24	-	a_Mdub_UR_15_24	-	3.2e-29	93.9	3.0	3.4e-29	93.8	3.0	1.0	1	0	0	1	1	1	1	[641 - 510] (REVERSE SENSE)
a_Mdub_UR_15_25	-	a_Mdub_UR_15_25	-	4.1e-19	61.0	0.8	4.2e-19	60.9	0.8	1.0	1	0	0	1	1	1	1	[465 - 385] (REVERSE SENSE)
m_Mdub_UR_15_25	-	a_Mdub_UR_15_25	-	6.3e-19	60.4	0.8	6.6e-19	60.4	0.8	1.0	1	0	0	1	1	1	1	[465 - 385] (REVERSE SENSE)
a_Mdub_UR_15_26	-	a_Mdub_UR_15_26	-	2.6e-20	65.2	10.1	2.8e-20	65.1	10.1	1.0	1	0	0	1	1	1	1	[437 - 336] (REVERSE SENSE)
m_Mdub_UR_15_26	-	a_Mdub_UR_15_26	-	2.6e-20	65.2	10.1	2.8e-20	65.1	10.1	1.0	1	0	0	1	1	1	1	[437 - 336] (REVERSE SENSE)
a_Mdub_UR_15_27	-	a_Mdub_UR_15_27	-	2.4e-85	278.8	9.0	2.7e-85	278.7	9.0	1.0	1	0	0	1	1	1	1	[667 - 293] (REVERSE SENSE)
m_Mdub_UR_15_27	-	a_Mdub_UR_15_27	-	2.4e-85	278.8	9.0	2.7e-85	278.7	9.0	1.0	1	0	0	1	1	1	1	[667 - 293] (REVERSE SENSE)
a_Mdub_UR_15_28	-	a_Mdub_UR_15_28	-	5.1e-12	37.2	0.1	5.2e-12	37.1	0.1	1.0	1	0	0	1	1	1	1	[293 - 234] (REVERSE SENSE)
m_Mdub_UR_15_28	-	a_Mdub_UR_15_28	-	5.1e-12	37.2	0.1	5.2e-12	37.1	0.1	1.0	1	0	0	1	1	1	1	[293 - 234] (REVERSE SENSE)
a_Mdub_UR_15_29	-	a_Mdub_UR_15_29	-	3.2e-12	37.6	0.9	3.3e-12	37.6	0.9	1.0	1	0	0	1	1	1	1	[286 - 227] (REVERSE SENSE)
m_Mdub_UR_15_29	-	a_Mdub_UR_15_29	-	5.5e-12	37.0	0.9	5.6e-12	37.0	0.9	1.0	1	0	0	1	1	1	1	[286 - 227] (REVERSE SENSE)
a_Mdub_UR_15_30	-	a_Mdub_UR_15_30	-	2.1e-43	140.2	4.0	2.3e-43	140.1	4.0	1.0	1	0	0	1	1	1	1	[375 - 190] (REVERSE SENSE)
m_Mdub_UR_15_30	-	a_Mdub_UR_15_30	-	2.9e-43	139.8	3.8	3.2e-43	139.7	3.8	1.0	1	0	0	1	1	1	1	[375 - 190] (REVERSE SENSE)
a_Mdub_UR_15_31	-	a_Mdub_UR_15_31	-	1.2e-06	19.6	1.1	1.2e-06	19.6	1.1	1.0	1	0	0	1	1	1	1	[206 - 174] (REVERSE SENSE)
m_Mdub_UR_15_31	-	a_Mdub_UR_15_31	-	3.3e-06	18.6	1.3	3.3e-06	18.6	1.3	1.0	1	0	0	1	1	1	1	[206 - 174] (REVERSE SENSE)
a_Mdub_UR_15_32	-	a_Mdub_UR_15_32	-	5.4e-05	15.0	1.4	5.4e-05	15.0	1.4	1.1	1	0	0	1	1	1	1	[170 - 138] (REVERSE SENSE)
m_Mdub_UR_15_32	-	a_Mdub_UR_15_32	-	0.00011	14.3	1.5	0.00011	14.3	1.5	1.1	1	0	0	1	1	1	1	[170 - 138] (REVERSE SENSE)
a_Mdub_UR_15_33	-	a_Mdub_UR_15_33	-	1e-09	29.8	0.1	1e-09	29.8	0.1	1.0	1	0	0	1	1	1	1	[180 - 133] (REVERSE SENSE)
m_Mdub_UR_15_33	-	a_Mdub_UR_15_33	-	1.8e-09	29.2	0.2	1.8e-09	29.2	0.2	1.0	1	0	0	1	1	1	1	[180 - 133] (REVERSE SENSE)
a_Mdub_UR_15_34	-	a_Mdub_UR_15_34	-	2e-07	23.1	0.6	2e-07	23.1	0.6	1.0	1	0	0	1	1	1	1	[109 - 74] (REVERSE SENSE)
m_Mdub_UR_15_34	-	a_Mdub_UR_15_34	-	3.6e-07	22.5	0.6	3.6e-07	22.5	0.6	1.0	1	0	0	1	1	1	1	[109 - 74] (REVERSE SENSE)
a_Mdub_UR_15_35	-	a_Mdub_UR_15_35	-	2.3e-09	29.4	2.3	2.3e-09	29.4	2.3	1.0	1	0	0	1	1	1	1	[89 - 42] (REVERSE SENSE)
m_Mdub_UR_15_35	-	a_Mdub_UR_15_35	-	5.8e-09	28.3	2.4	5.8e-09	28.3	2.4	1.0	1	0	0	1	1	1	1	[89 - 42] (REVERSE SENSE)
a_Mdub_UR_15_36	-	a_Mdub_UR_15_36	-	1.4e-07	22.5	0.4	1.4e-07	22.5	0.4	1.0	1	0	0	1	1	1	1	[63 - 22] (REVERSE SENSE)
m_Mdub_UR_15_36	-	a_Mdub_UR_15_36	-	1.4e-07	22.5	0.4	1.4e-07	22.5	0.4	1.0	1	0	0	1	1	1	1	[63 - 22] (REVERSE SENSE)
a_Mdub_UR_16_1	-	a_Mdub_UR_16_1	-	2.5e-06	19.3	0.3	2.5e-06	19.3	0.3	1.0	1	0	0	1	1	1	1	[45 - 74]
m_Mdub_UR_16_1	-	a_Mdub_UR_16_1	-	5.6e-06	18.4	0.3	5.6e-06	18.4	0.3	1.0	1	0	0	1	1	1	1	[45 - 74]
a_Mdub_UR_16_2	-	a_Mdub_UR_16_2	-	1.8e-08	26.7	2.6	1.8e-08	26.7	2.6	1.0	1	0	0	1	1	1	1	[40 - 90]
m_Mdub_UR_16_2	-	a_Mdub_UR_16_2	-	3.7e-08	25.8	2.6	3.7e-08	25.8	2.6	1.0	1	0	0	1	1	1	1	[40 - 90]
a_Mdub_UR_16_3	-	a_Mdub_UR_16_3	-	4.2e-06	18.5	1.3	4.2e-06	18.5	1.3	1.0	1	0	0	1	1	1	1	[81 - 110]
m_Mdub_UR_16_3	-	a_Mdub_UR_16_3	-	4.2e-06	18.5	1.3	4.2e-06	18.5	1.3	1.0	1	0	0	1	1	1	1	[81 - 110]
a_Mdub_UR_16_4	-	a_Mdub_UR_16_4	-	1.3e-06	20.0	2.2	1.3e-06	20.0	2.2	1.0	1	0	0	1	1	1	1	[98 - 136]
m_Mdub_UR_16_4	-	a_Mdub_UR_16_4	-	1.3e-06	20.0	2.2	1.3e-06	20.0	2.2	1.0	1	0	0	1	1	1	1	[98 - 136]
a_Mdub_UR_16_5	-	a_Mdub_UR_16_5	-	4.9e-07	22.4	4.5	5e-07	22.3	4.5	1.0	1	0	0	1	1	1	1	[137 - 93] (REVERSE SENSE)
m_Mdub_UR_16_5	-	a_Mdub_UR_16_5	-	8.4e-07	21.8	4.4	8.5e-07	21.7	4.4	1.0	1	0	0	1	1	1	1	[137 - 93] (REVERSE SENSE)
a_Mdub_UR_16_6	-	a_Mdub_UR_16_6	-	2.5e-08	26.2	1.7	2.5e-08	26.2	1.7	1.0	1	0	0	1	1	1	1	[132 - 88] (REVERSE SENSE)
m_Mdub_UR_16_6	-	a_Mdub_UR_16_6	-	4.2e-08	25.6	1.4	4.2e-08	25.6	1.4	1.0	1	0	0	1	1	1	1	[132 - 88] (REVERSE SENSE)
a_Mdub_UR_16_7	-	a_Mdub_UR_16_7	-	7.7e-15	46.3	0.5	8e-15	46.2	0.5	1.0	1	0	0	1	1	1	1	[89 - 12] (REVERSE SENSE)
m_Mdub_UR_16_7	-	a_Mdub_UR_16_7	-	1.6e-14	45.4	0.6	1.7e-14	45.3	0.6	1.0	1	0	0	1	1	1	1	[89 - 12] (REVERSE SENSE)
a_Mdub_UR_16_8	-	a_Mdub_UR_16_8	-	1.2e-11	37.4	2.6	1.2e-11	37.3	2.6	1.0	1	0	0	1	1	1	1	[67 - 8] (REVERSE SENSE)
m_Mdub_UR_16_8	-	a_Mdub_UR_16_8	-	1.9e-11	36.8	2.5	1.9e-11	36.7	2.5	1.0	1	0	0	1	1	1	1	[67 - 8] (REVERSE SENSE)
a_Mdub_UR_16_9	-	a_Mdub_UR_16_9	-	5.8e-06	18.1	0.2	5.8e-06	18.1	0.2	1.0	1	0	0	1	1	1	1	[30 - 1] (REVERSE SENSE)
m_Mdub_UR_16_9	-	a_Mdub_UR_16_9	-	1.5e-05	17.1	0.2	1.5e-05	17.1	0.2	1.0	1	0	0	1	1	1	1	[30 - 1] (REVERSE SENSE)
a_Mdub_UR_23_1	-	a_Mdub_UR_23_1	-	3.4e-05	16.5	1.6	3.4e-05	16.5	1.6	1.0	1	0	0	1	1	1	1	[35 - 3] (REVERSE SENSE)
m_Mdub_UR_23_1	-	a_Mdub_UR_23_1	-	5.3e-05	16.0	1.4	5.3e-05	16.0	1.4	1.1	1	0	0	1	1	1	1	[35 - 3] (REVERSE SENSE)
a_HmenF_UR_1_1	-	a_HmenF_UR_1_1	-	5.4e-08	25.4	3.8	5.4e-08	25.4	3.8	1.0	1	0	0	1	1	1	1	[34 - 69]
m_HmenF_UR_1_1	-	a_HmenF_UR_1_1	-	5.4e-08	25.4	3.8	5.4e-08	25.4	3.8	1.0	1	0	0	1	1	1	1	[34 - 69]
a_HmenF_UR_1_2	-	a_HmenF_UR_1_2	-	1.8e-07	22.9	0.5	1.8e-07	22.9	0.5	1.0	1	0	0	1	1	1	1	[36 - 71]

m_HmenF_UR_1_2	-	a_HmenF_UR_1_2	-	4.5e-07	21.8	0.6	4.5e-07	21.8	0.6	1.0	1	0	0	1	1	1	1	[36 - 71]
m_HmenF_UR_1_3	-	a_HmenF_UR_1_3	-	0.00013	15.7	3.0	0.00013	15.7	3.0	1.1	1	0	0	1	1	1	1	[69 - 40] (REVERSE SENSE)
a_HmenF_UR_1_3	-	a_HmenF_UR_1_3	-	0.00015	15.5	4.0	0.00015	15.5	4.0	1.1	1	0	0	1	1	1	1	[69 - 40] (REVERSE SENSE)
a_HmenF_UR_1_4	-	a_HmenF_UR_1_4	-	3.3e-13	42.2	3.5	3.4e-13	42.2	3.5	1.0	1	0	0	1	1	1	1	[62 - 3] (REVERSE SENSE)
m_HmenF_UR_1_4	-	a_HmenF_UR_1_4	-	5.1e-13	41.7	3.3	5.2e-13	41.7	3.3	1.0	1	0	0	1	1	1	1	[62 - 3] (REVERSE SENSE)
a_HmenF_UR_1_5	-	a_HmenF_UR_1_5	-	4.2e-12	39.3	6.7	4.3e-12	39.2	6.7	1.0	1	0	0	1	1	1	1	[55 - 2] (REVERSE SENSE)
m_HmenF_UR_1_5	-	a_HmenF_UR_1_5	-	4.2e-12	39.3	6.7	4.3e-12	39.2	6.7	1.0	1	0	0	1	1	1	1	[55 - 2] (REVERSE SENSE)
a_HmenF_UR_2_1	-	a_HmenF_UR_2_1	-	4.6e-07	22.1	0.3	4.6e-07	22.1	0.3	1.0	1	0	0	1	1	1	1	[36 - 1] (REVERSE SENSE)
m_HmenF_UR_2_1	-	a_HmenF_UR_2_1	-	7.8e-07	21.5	0.2	7.8e-07	21.5	0.2	1.0	1	0	0	1	1	1	1	[36 - 1] (REVERSE SENSE)
a_HmenF_UR_5_1	-	a_HmenF_UR_5_1	-	4.1e-07	21.3	1.1	4.1e-07	21.3	1.1	1.0	1	0	0	1	1	1	1	[11 - 43]
m_HmenF_UR_5_1	-	a_HmenF_UR_5_1	-	4.1e-07	21.3	1.1	4.1e-07	21.3	1.1	1.0	1	0	0	1	1	1	1	[11 - 43]
a_HmenF_UR_5_2	-	a_HmenF_UR_5_2	-	1.4e-09	31.3	7.7	1.4e-09	31.3	7.7	1.0	1	0	0	1	1	1	1	[43 - 2] (REVERSE SENSE)
m_HmenF_UR_5_2	-	a_HmenF_UR_5_2	-	4.1e-09	30.0	8.1	4.1e-09	30.0	8.1	1.0	1	0	0	1	1	1	1	[43 - 2] (REVERSE SENSE)
a_HmenF_UR_5_3	-	a_HmenF_UR_5_3	-	1.7e-05	16.5	0.2	1.7e-05	16.5	0.2	1.0	1	0	0	1	1	1	1	[30 - 1] (REVERSE SENSE)
m_HmenF_UR_5_3	-	a_HmenF_UR_5_3	-	1.7e-05	16.5	0.2	1.7e-05	16.5	0.2	1.0	1	0	0	1	1	1	1	[30 - 1] (REVERSE SENSE)
a_HmenF_UR_8_1	-	a_HmenF_UR_8_1	-	3.4e-06	18.5	0.4	3.4e-06	18.5	0.4	1.0	1	0	0	1	1	1	1	[3 - 32]
m_HmenF_UR_8_1	-	a_HmenF_UR_8_1	-	8.4e-06	17.5	0.4	8.4e-06	17.5	0.4	1.0	1	0	0	1	1	1	1	[3 - 32]
a_HmenF_UR_8_2	-	a_HmenF_UR_8_2	-	5.8e-07	22.0	1.1	5.8e-07	22.0	1.1	1.0	1	0	0	1	1	1	1	[32 - 3] (REVERSE SENSE)
m_HmenF_UR_8_2	-	a_HmenF_UR_8_2	-	1.2e-06	21.1	0.9	1.2e-06	21.1	0.9	1.0	1	0	0	1	1	1	1	[32 - 3] (REVERSE SENSE)
a_HmenF_UR_10_1	-	a_HmenF_UR_10_1	-	7.8e-08	24.9	2.1	7.8e-08	24.9	2.1	1.0	1	0	0	1	1	1	1	[41 - 3] (REVERSE SENSE)
m_HmenF_UR_10_1	-	a_HmenF_UR_10_1	-	1.2e-07	24.4	2.0	1.2e-07	24.4	2.0	1.0	1	0	0	1	1	1	1	[41 - 3] (REVERSE SENSE)
a_HmenF_UR_13_1	-	a_HmenF_UR_13_1	-	5.5e-08	23.7	0.1	5.5e-08	23.7	0.1	1.0	1	0	0	1	1	1	1	[3 - 44]
m_HmenF_UR_13_1	-	a_HmenF_UR_13_1	-	1.2e-07	22.8	0.1	1.2e-07	22.8	0.1	1.0	1	0	0	1	1	1	1	[3 - 44]
a_HmenF_UR_13_2	-	a_HmenF_UR_13_2	-	6.6e-09	28.5	1.4	6.6e-09	28.5	1.4	1.0	1	0	0	1	1	1	1	[38 - 3] (REVERSE SENSE)
m_HmenF_UR_13_2	-	a_HmenF_UR_13_2	-	1.1e-08	27.8	1.4	1.1e-08	27.8	1.4	1.0	1	0	0	1	1	1	1	[38 - 3] (REVERSE SENSE)
a_HmenF_UR_13_3	-	a_HmenF_UR_13_3	-	1.8e-05	15.3	0.3	1.8e-05	15.3	0.3	1.0	1	0	0	1	1	1	1	[31 - 2] (REVERSE SENSE)
m_HmenF_UR_13_3	-	a_HmenF_UR_13_3	-	1.8e-05	15.3	0.3	1.8e-05	15.3	0.3	1.0	1	0	0	1	1	1	1	[31 - 2] (REVERSE SENSE)
a_HmenF_UR_16_1	-	a_HmenF_UR_16_1	-	3.6e-08	26.1	1.3	3.6e-08	26.1	1.3	1.0	1	0	0	1	1	1	1	[39 - 1] (REVERSE SENSE)
m_HmenF_UR_16_1	-	a_HmenF_UR_16_1	-	7.1e-08	25.3	1.2	7.1e-08	25.3	1.2	1.0	1	0	0	1	1	1	1	[39 - 1] (REVERSE SENSE)
a_HmenF_UR_17_1	-	a_HmenF_UR_17_1	-	7.9e-06	17.3	0.1	7.9e-06	17.3	0.1	1.0	1	0	0	1	1	1	1	[35 - 64]
m_HmenF_UR_17_1	-	a_HmenF_UR_17_1	-	7.9e-06	17.3	0.1	7.9e-06	17.3	0.1	1.0	1	0	0	1	1	1	1	[35 - 64]
a_HmenF_UR_17_2	-	a_HmenF_UR_17_2	-	2.8e-11	34.1	0.2	2.9e-11	34.1	0.2	1.0	1	0	0	1	1	1	1	[69 - 122]
m_HmenF_UR_17_2	-	a_HmenF_UR_17_2	-	6.5e-11	33.2	0.3	6.5e-11	33.2	0.3	1.0	1	0	0	1	1	1	1	[69 - 122]
a_HmenF_UR_17_3	-	a_HmenF_UR_17_3	-	5.7e-19	59.6	1.3	6e-19	59.6	1.3	1.0	1	0	0	1	1	1	1	[37 - 129]
m_HmenF_UR_17_3	-	a_HmenF_UR_17_3	-	1.4e-18	58.5	1.5	1.4e-18	58.5	1.5	1.0	1	0	0	1	1	1	1	[37 - 129]
a_HmenF_UR_17_4	-	a_HmenF_UR_17_4	-	5.2e-13	41.2	0.6	5.3e-13	41.1	0.6	1.0	1	0	0	1	1	1	1	[119 - 60] (REVERSE SENSE)
m_HmenF_UR_17_4	-	a_HmenF_UR_17_4	-	5.2e-13	41.2	0.6	5.3e-13	41.1	0.6	1.0	1	0	0	1	1	1	1	[119 - 60] (REVERSE SENSE)
a_HmenF_UR_17_5	-	a_HmenF_UR_17_5	-	7.7e-17	53.4	0.3	8e-17	53.4	0.3	1.0	1	0	0	1	1	1	1	[100 - 26] (REVERSE SENSE)
m_HmenF_UR_17_5	-	a_HmenF_UR_17_5	-	1.3e-16	52.8	0.3	1.3e-16	52.7	0.3	1.0	1	0	0	1	1	1	1	[100 - 26] (REVERSE SENSE)
a_HmenF_UR_17_6	-	a_HmenF_UR_17_6	-	1.9e-07	23.2	0.2	1.9e-07	23.2	0.2	1.0	1	0	0	1	1	1	1	[48 - 10] (REVERSE SENSE)
m_HmenF_UR_17_6	-	a_HmenF_UR_17_6	-	3.3e-07	22.6	0.2	3.3e-07	22.6	0.2	1.0	1	0	0	1	1	1	1	[48 - 10] (REVERSE SENSE)
a_HmenF_UR_21_1	-	a_HmenF_UR_21_1	-	1.3e-06	21.0	1.0	1.3e-06	21.0	1.0	1.0	1	0	0	1	1	1	1	[39 - 4] (REVERSE SENSE)
m_HmenF_UR_21_1	-	a_HmenF_UR_21_1	-	2e-06	20.5	0.8	2e-06	20.5	0.8	1.0	1	0	0	1	1	1	1	[39 - 4] (REVERSE SENSE)
a_HmenF_UR_24_1	-	a_HmenF_UR_24_1	-	3.2e-10	32.7	6.4	3.2e-10	32.7	6.4	1.0	1	0	0	1	1	1	1	[6 - 62]
m_HmenF_UR_24_1	-	a_HmenF_UR_24_1	-	3.2e-10	32.7	6.4	3.2e-10	32.7	6.4	1.0	1	0	0	1	1	1	1	[6 - 62]
a_HmenF_UR_24_2	-	a_HmenF_UR_24_2	-	1.7e-10	33.9	5.2	1.7e-10	33.9	5.2	1.0	1	0	0	1	1	1	1	[25 - 75]
m_HmenF_UR_24_2	-	a_HmenF_UR_24_2	-	3.6e-10	33.0	5.2	3.6e-10	33.0	5.2	1.0	1	0	0	1	1	1	1	[25 - 75]
a_HmenF_UR_24_3	-	a_HmenF_UR_24_3	-	2.6e-07	22.2	0.7	2.6e-07	22.2	0.7	1.0	1	0	0	1	1	1	1	[53 - 88]
m_HmenF_UR_24_3	-	a_HmenF_UR_24_3	-	5.4e-07	21.4	0.5	5.4e-07	21.4	0.5	1.0	1	0	0	1	1	1	1	[53 - 88]
a_HmenF_UR_24_4	-	a_HmenF_UR_24_4	-	2.1e-06	21.7	5.7	2.1e-06	21.7	5.7	1.0	1	0	0	1	1	1	1	[68 - 36] (REVERSE SENSE)
m_HmenF_UR_24_4	-	a_HmenF_UR_24_4	-	2.4e-06	21.5	5.2	2.4e-06	21.5	5.2	1.0	1	0	0	1	1	1	1	[68 - 36] (REVERSE SENSE)
a_HmenF_UR_24_5	-	a_HmenF_UR_24_5	-	1.4e-11	36.7	1.9	1.4e-11	36.7	1.9	1.0	1	0	0	1	1	1	1	[84 - 25] (REVERSE SENSE)
m_HmenF_UR_24_5	-	a_HmenF_UR_24_5	-	2.3e-11	36.1	1.6	2.3e-11	36.1	1.6	1.0	1	0	0	1	1	1	1	[84 - 25] (REVERSE SENSE)
a_HmenF_UR_24_6	-	a_HmenF_UR_24_6	-	4.1e-12	38.9	2.9	4.2e-12	38.9	2.9	1.0	1	0	0	1	1	1	1	[61 - 2] (REVERSE SENSE)
m_HmenF_UR_24_6	-	a_HmenF_UR_24_6	-	5.8e-12	38.5	2.4	5.8e-12	38.5	2.4	1.0	1	0	0	1	1	1	1	[61 - 2] (REVERSE SENSE)
a_HmenF_UR_25_1	-	a_HmenF_UR_25_1	-	1.5e-06	19.9	0.3	1.5e-06	19.9	0.3	1.0	1	0	0	1	1	1	1	[74 - 103]
m_HmenF_UR_25_1	-	a_HmenF_UR_25_1	-	3.5e-06	19.0	0.2	3.5e-06	19.0	0.2	1.0	1	0	0	1	1	1	1	[74 - 103]
a_HmenF_UR_25_2	-	a_HmenF_UR_25_2	-	9.8e-08	24.2	0.4	9.8e-08	24.2	0.4	1.0	1	0	0	1	1	1	1	[94 - 53] (REVERSE SENSE)
m_HmenF_UR_25_2	-	a_HmenF_UR_25_2	-	9.8e-08	24.2	0.4	9.8e-08	24.2	0.4	1.0	1	0	0	1	1	1	1	[94 - 53] (REVERSE SENSE)
a_HmenF_UR_25_3	-	a_HmenF_UR_25_3	-	1.1e-09	30.1	0.8	1.1e-09	30.1	0.8	1.0	1	0	0	1	1	1	1	[75 - 34] (REVERSE SENSE)
m_HmenF_UR_25_3	-	a_HmenF_UR_25_3	-	1.1e-09	30.1	0.8	1.1e-09	30.1	0.8	1.0	1	0	0	1	1	1	1	[75 - 34] (REVERSE SENSE)
a_HmenF_UR_26_1	-	a_HmenF_UR_26_1	-	1.4e-05	15.4	0.3	1.4e-05	15.4	0.3	1.0	1	0	0	1	1	1	1	[1 - 30]
m_HmenF_UR_26_1	-	a_HmenF_UR_26_1	-	1.4e-05	15.4	0.3	1.4e-05	15.4	0.3	1.0	1	0	0	1	1	1	1	[1 - 30]
a_HmenF_UR_26_2	-	a_HmenF_UR_26_2	-	4.1e-07	21.6	0.1	4.1e-07	21.6	0.1	1.0	1	0	0	1	1	1	1	[36 - 1] (REVERSE SENSE)
m_HmenF_UR_26_2	-	a_HmenF_UR_26_2	-	7.1e-07	21.0	0.1	7.1e-07	21.0	0.1	1.0	1	0	0	1	1	1	1	[36 - 1] (REVERSE SENSE)
a_CmonF_UR_1_1	-	a_CmonF_UR_1_1	-	6.6e-06	17.4	0.5	6.6e-06	17.4	0.5	1.0	1	0	0	1	1	1	1	[31 - 2] (REVERSE SENSE)
m_CmonF_UR_1_1	-	a_CmonF_UR_1_1	-	6.6e-06	17.4	0.5	6.6e-06	17.4	0.5	1.0	1	0	0	1	1	1	1	[31 - 2] (REVERSE SENSE)
a_CmonF_UR_3_1	-	a_CmonF_UR_3_1	-	4.6e-06	19.2	0.4	4.6e-06	19.2	0.4	1.0	1	0	0	1	1	1	1	[31 - 2] (REVERSE SENSE)
m_CmonF_UR_3_1	-	a_CmonF_UR_3_1	-	7.9e-06	18.6	0.3	7.9e-06	18.6	0.3	1.0	1	0	0	1	1	1	1	[31 - 2] (REVERSE SENSE)
a_CmonF_UR_4_1	-	a_CmonF_UR_4_1	-	4.1e-13	41.1	1.0	4.2e-13	41.1	1.0	1.0	1	0	0	1	1	1	1	[32 - 91]
m_CmonF_UR_4_1	-	a_CmonF_UR_4_1	-	9e-13	40.1	1.0	9.2e-13	40.1	1.0	1.0	1	0	0	1	1	1	1	[32 - 91]
a_CmonF_UR_4_2	-	a_CmonF_UR_4_2	-	3.3e-15	46.7	0.6	3.5e-15	46.6	0.6	1.0	1	0	0	1	1	1	1	[61 - 138]
m_CmonF_UR_4_2	-	a_CmonF_UR_4_2	-	7.4e-15	45.7	0.7	7.7e-15	45.7	0.7	1.0	1	0	0	1	1	1	1	[61 - 138]
a_CmonF_UR_4_3	-	a_CmonF_UR_4_3	-	1.3e-05	15.3	1.4	1.3e-05	15.3	1.4	1.1	1	0	0	1	1	1	1	[154 - 183]
m_CmonF_UR_4_3	-	a_CmonF_UR_4_3	-	1.3e-05	15.3	1.4	1.3e-05	15.3	1.4	1.1	1	0	0	1	1	1	1	[154 - 183]
a_CmonF_UR_4_4	-	a_CmonF_UR_4_4	-	3.2e-15	48.1	6.6	3.4e-15	48.0	6.6	1.0	1	0	0	1	1	1	1	[138 - 215]
m_CmonF_UR_4_4	-	a_CmonF_UR_4_4	-	3.2e-15	48.1	6.6	3.4e-15	48.0	6.6	1.0	1	0	0	1	1	1	1	[138 - 215]
a_CmonF_UR_4_5	-	a_CmonF_UR_4_5	-	1.3e-07	24.7	5.3	1.3e-07	24.7	5.3	1.0	1	0	0	1	1	1	1	[162 - 124] (REVERSE SENSE)
m_CmonF_UR_4_5	-	a_CmonF_UR_4_5	-	1.6e-07	24.5	4.9	1.6e-07	24.5	4.9	1.0	1	0	0	1	1	1	1	[162 - 124] (REVERSE SENSE)
m_CmonF_UR_4_6	-	a_CmonF_UR_4_6	-	1.5e-13	43.5	5.1	1.6e-13	43.5	5.1	1.0	1	0	0	1	1	1	1	[196 - 116] (REVERSE SENSE)
a_CmonF_UR_4_6	-	a_CmonF_UR_4_6	-	1.6e-13	43.5	5.7	1.7e-13	43.4	5.7	1.0	1	0	0	1	1	1	1	[196 - 116] (REVERSE SENSE)
a_CmonF_UR_4_7	-	a_CmonF_UR_4_7	-	1.6e-08	26.4	0.9	1.6e-08	26.4	0.9	1.0	1	0	0	1	1	1	1	[108 - 70] (REVERSE SENSE)
m_CmonF_UR_4_7	-	a_CmonF_UR_4_7	-	2.5e-08	25.9	0.7	2.5e-08	25.9	0.7	1.0	1	0	0	1	1	1	1	[108 - 70] (REVERSE SENSE)
a_CmonF_UR_4_8	-	a_CmonF_UR_4_8	-	7.6e-37	120.0	13.5	8.3e-37	119.9	13.5	1.0	1	0	0	1	1	1	1	[179 - 3] (REVERSE SENSE)
m_CmonF_UR_4_8	-	a_CmonF_UR_4_8	-	1.3e-36	119.2	13.4	1.5e-36	119.1	13.4	1.0	1	0	0	1	1	1	1	[179 - 3] (REVERSE SENSE)
a_CmonF_UR_4_9	-	a_CmonF_UR_4_9	-	2.6e-15	49.3	6.0	2.7e-15	49.2	6.0	1.0	1	0	0	1	1	1	1	[73 - 2] (REVERSE SENSE)
m_CmonF_UR_4_9	-	a_CmonF_UR_4_9	-	5e-15	48.4	5.9	5.1e-15	48.4	5.9	1.0	1	0	0	1	1	1	1	[73 - 2] (REVERSE SENSE)
a_CmonF_UR_4_10	-	a_CmonF_UR_4_10	-	2e-06	19.8	0.1	2e-06	19.8	0.1	1.0	1	0	0	1	1	1	1	[33 - 1] (REVERSE SENSE)
m_CmonF_UR_4_10	-	a_CmonF_UR_4_10	-	4.7e-06	18.9	0.1	4.7e-06	18.9	0.1	1.0	1	0	0	1	1	1	1	[33 - 1] (REVERSE SENSE)
a_CmonF_UR_7_1	-	a_CmonF_UR_7_1	-	5.4e-08	24.4	0.2	5.4e-08	24.4	0.2	1.0	1	0	0	1	1	1	1	[6 - 44]
m_CmonF_UR_7_1	-	a_CmonF_UR_7_1	-	1.3e-07	23.4	0.2	1.3e-07	23.4	0.2	1.0	1	0	0	1	1	1	1	[6 - 44]
a_CmonF_UR_7_2	-	a_CmonF_UR_7_2	-	6.2e-10	30.0	0.8	6.2e-10	30.0	0.8	1.0	1	0	0	1	1	1	1	[1 - 45]
m_CmonF_UR_7_2	-	a_CmonF_UR_7_2	-	6.2e-10	30.0	0.8	6.2e-10	30.0	0.8	1.0	1	0	0	1	1	1	1	[1 - 45]
a_CmonF_UR_7_3	-	a_CmonF_UR_7_3	-	1.6e-07	24.3	4.1	1.6e-07	24.3	4.1	1.0	1	0	0	1	1	1	1	[38 - 3] (REVERSE SENSE)
m_CmonF_UR_7_3	-	a_CmonF_UR_7_3	-	1.9e-07	24.1	3.7	1.9e-07	24.1	3.7	1.0	1	0	0	1	1	1	1	[38 - 3] (REVERSE SENSE)
a_CmonF_UR_8_1	-	a_CmonF_UR_8_1	-	4.5e-06	19.1	0.5	4.5e-06	19.1	0.5	1.0	1	0	0	1	1	1	1	[7 - 36]
m_CmonF_UR_8_1	-	a_CmonF_UR_8_1	-	9.5e-06	18.2	0.4	9.5e-06	18.2	0.4	1.0	1	0	0	1	1	1	1	[7 - 36]
a_CmonF_UR_8_2	-	a_CmonF_UR_8_2	-	2.3e-06	19.8	0.1	2.3e-06	19.8	0.1	1.0	1	0	0	1	1	1	1	[33 - 1] (REVERSE SENSE)
m_CmonF_UR_8_2	-	a_CmonF_UR_8_2	-	5.3e-06	19.0	0.1	5.3e-06	19.0	0.1	1.0	1	0	0	1	1	1	1	[33 - 1] (REVERSE SENSE)
a_CmonF_UR_10_1	-	a_CmonF_UR_10_1	-	1.6e-08	25.8	0.1	1.6e-08	25.8	0.1	1.0	1	0	0	1	1	1	1	[24 - 62]
m_CmonF_UR_10_1	-	a_CmonF_UR_10_1	-	3.5e-08	24.9	0.1	3.5e-08	24.9	0.1	1.0	1	0	0	1	1	1	1	[24 - 62]
a_CmonF_UR_10_2	-	a_CmonF_UR_10_2	-	2e-08	26.3	1.6	2e-08	26.3	1.6	1.0	1	0	0	1	1	1	1	[43 - 2] (REVERSE SENSE)
m_CmonF_UR_10_2	-	a_CmonF_UR_10_2	-	2e-08	26.3	1.6	2e-08	26.3	1.6	1.0	1	0	0	1	1	1	1	[43 - 2] (REVERSE SENSE)
a_CmonF_UR_17_1	-	a_CmonF_UR_17_1	-	1.2e-14	45.2	0.5	1.3e-14	45.2	0.5	1.0	1	0	0	1	1	1	1	[9 - 71]
m_CmonF_UR_17_1	-	a_CmonF_UR_17_1	-	1.2e-14	45.2	0.5	1.3e-14	45.2	0.5	1.0	1	0	0	1	1	1	1	[9 - 71]
a_CmonF_UR_17_2	-	a_CmonF_UR_17_2	-	9.9e-12	36.2	0.4	1e-11	36.2	0.4	1.0	1	0	0	1	1	1	1	[19 - 75]
m_CmonF_UR_17_2	-	a_CmonF_UR_17_2	-	2.2e-11	35.3	0.4	2.2e-11	35.2	0.4	1.0	1	0	0	1	1	1	1	[19 - 75]
a_CmonF_UR_17_3	-	a_CmonF_UR_17_3	-	1.9e-15	49.9	5.2	2e-15	49.8	5.2	1.0	1	0	0	1	1	1	1	[71 - 3] (REVERSE SENSE)
m_CmonF_UR_17_3	-	a_CmonF_UR_17_3	-	4.3e-15	48.8	5.4	4.4e-15	48.8	5.4	1.0	1	0	0	1	1	1	1	[71 - 3] (REVERSE SENSE)

a_CmonF_UR_17_4	-	a_CmonF_UR_17_4	-	8.6e-15	47.7	4.5	8.9e-15	47.6	4.5	1.0	1	0	0	1	1	1	1	[75 - 1]	(REVERSE SENSE)
m_CmonF_UR_17_4	-	a_CmonF_UR_17_4	-	2e-14	46.6	4.7	2.1e-14	46.6	4.7	1.0	1	0	0	1	1	1	1	[75 - 1]	(REVERSE SENSE)
a_CmonF_UR_18_1	-	a_CmonF_UR_18_1	-	1.1e-07	23.4	1.1	1.1e-07	23.4	1.1	1.0	1	0	0	1	1	1	1	[2 - 37]	
m_CmonF_UR_18_1	-	a_CmonF_UR_18_1	-	1.1e-07	23.4	1.1	1.1e-07	23.4	1.1	1.0	1	0	0	1	1	1	1	[2 - 37]	
a_CmonF_UR_18_2	-	a_CmonF_UR_18_2	-	2.1e-06	20.4	0.9	2.1e-06	20.4	0.9	1.0	1	0	0	1	1	1	1	[38 - 3]	(REVERSE SENSE)
m_CmonF_UR_18_2	-	a_CmonF_UR_18_2	-	2.1e-06	20.4	0.9	2.1e-06	20.4	0.9	1.0	1	0	0	1	1	1	1	[38 - 3]	(REVERSE SENSE)
a_CmonF_UR_19_1	-	a_CmonF_UR_19_1	-	4.9e-07	20.7	0.5	4.9e-07	20.7	0.5	1.0	1	0	0	1	1	1	1	[1 - 30]	
m_CmonF_UR_19_1	-	a_CmonF_UR_19_1	-	4.9e-07	20.7	0.5	4.9e-07	20.7	0.5	1.0	1	0	0	1	1	1	1	[1 - 30]	
a_CmonF_UR_19_2	-	a_CmonF_UR_19_2	-	1.2e-07	23.8	1.9	1.2e-07	23.8	1.9	1.0	1	0	0	1	1	1	1	[14 - 52]	
m_CmonF_UR_19_2	-	a_CmonF_UR_19_2	-	1.7e-07	23.4	1.4	1.7e-07	23.4	1.4	1.0	1	0	0	1	1	1	1	[14 - 52]	
a_CmonF_UR_19_3	-	a_CmonF_UR_19_3	-	3.9e-06	17.3	0.1	3.9e-06	17.3	0.1	1.0	1	0	0	1	1	1	1	[21 - 53]	
m_CmonF_UR_19_3	-	a_CmonF_UR_19_3	-	1e-05	16.3	0.1	1e-05	16.3	0.1	1.0	1	0	0	1	1	1	1	[21 - 53]	
a_CmonF_UR_19_4	-	a_CmonF_UR_19_4	-	7.7e-07	21.1	0.3	7.7e-07	21.1	0.3	1.0	1	0	0	1	1	1	1	[35 - 3]	(REVERSE SENSE)
m_CmonF_UR_19_4	-	a_CmonF_UR_19_4	-	1.7e-06	20.2	0.2	1.7e-06	20.2	0.2	1.0	1	0	0	1	1	1	1	[35 - 3]	(REVERSE SENSE)
a_CmonF_UR_19_5	-	a_CmonF_UR_19_5	-	1.4e-08	26.4	0.7	1.4e-08	26.4	0.7	1.0	1	0	0	1	1	1	1	[45 - 1]	(REVERSE SENSE)
m_CmonF_UR_19_5	-	a_CmonF_UR_19_5	-	1.4e-08	26.4	0.7	1.4e-08	26.4	0.7	1.0	1	0	0	1	1	1	1	[45 - 1]	(REVERSE SENSE)
a_CmonF_UR_20_1	-	a_CmonF_UR_20_1	-	5.4e-08	23.2	0.2	5.4e-08	23.2	0.2	1.0	1	0	0	1	1	1	1	[5 - 43]	
m_CmonF_UR_20_1	-	a_CmonF_UR_20_1	-	5.4e-08	23.2	0.2	5.4e-08	23.2	0.2	1.0	1	0	0	1	1	1	1	[5 - 43]	
a_CmonF_UR_20_2	-	a_CmonF_UR_20_2	-	3.7e-07	21.2	0.2	3.7e-07	21.2	0.2	1.0	1	0	0	1	1	1	1	[21 - 56]	
m_CmonF_UR_20_2	-	a_CmonF_UR_20_2	-	6.5e-07	20.6	0.2	6.5e-07	20.6	0.2	1.0	1	0	0	1	1	1	1	[21 - 56]	
a_CmonF_UR_20_3	-	a_CmonF_UR_20_3	-	9.6e-09	26.6	0.7	9.6e-09	26.6	0.7	1.0	1	0	0	1	1	1	1	[62 - 21]	(REVERSE SENSE)
m_CmonF_UR_20_3	-	a_CmonF_UR_20_3	-	2e-08	25.7	0.7	2e-08	25.7	0.7	1.0	1	0	0	1	1	1	1	[62 - 21]	(REVERSE SENSE)
a_CmonF_UR_20_4	-	a_CmonF_UR_20_4	-	6e-10	30.6	0.9	6.1e-10	30.6	0.9	1.0	1	0	0	1	1	1	1	[49 - 2]	(REVERSE SENSE)
m_CmonF_UR_20_4	-	a_CmonF_UR_20_4	-	6e-10	30.6	0.9	6.1e-10	30.6	0.9	1.0	1	0	0	1	1	1	1	[49 - 2]	(REVERSE SENSE)
a_CmonF_UR_20_5	-	a_CmonF_UR_20_5	-	2e-08	25.8	0.5	2e-08	25.8	0.5	1.0	1	0	0	1	1	1	1	[42 - 1]	(REVERSE SENSE)
m_CmonF_UR_20_5	-	a_CmonF_UR_20_5	-	3.7e-08	25.1	0.5	3.7e-08	25.1	0.5	1.0	1	0	0	1	1	1	1	[42 - 1]	(REVERSE SENSE)
a_CmonF_UR_25_1	-	a_CmonF_UR_25_1	-	4.5e-08	24.7	0.3	4.5e-08	24.7	0.3	1.0	1	0	0	1	1	1	1	[2 - 46]	
m_CmonF_UR_25_1	-	a_CmonF_UR_25_1	-	9.7e-08	23.8	0.2	9.7e-08	23.8	0.2	1.0	1	0	0	1	1	1	1	[2 - 46]	
a_CmonF_UR_25_2	-	a_CmonF_UR_25_2	-	8.6e-09	28.2	4.5	8.6e-09	28.2	4.5	1.0	1	0	0	1	1	1	1	[44 - 3]	(REVERSE SENSE)
m_CmonF_UR_25_2	-	a_CmonF_UR_25_2	-	2.3e-08	27.0	4.8	2.3e-08	27.0	4.8	1.0	1	0	0	1	1	1	1	[44 - 3]	(REVERSE SENSE)
a_CmonF_UR_26_1	-	a_CmonF_UR_26_1	-	7.1e-07	19.5	0.2	7.1e-07	19.5	0.2	1.0	1	0	0	1	1	1	1	[1 - 33]	
m_CmonF_UR_26_1	-	a_CmonF_UR_26_1	-	7.1e-07	19.5	0.2	7.1e-07	19.5	0.2	1.0	1	0	0	1	1	1	1	[1 - 33]	
a_CmonF_UR_28_1	-	a_CmonF_UR_28_1	-	8.3e-08	25.0	1.0	8.3e-08	25.0	1.0	1.0	1	0	0	1	1	1	1	[51 - 13]	(REVERSE SENSE)
m_CmonF_UR_28_1	-	a_CmonF_UR_28_1	-	1.7e-07	24.1	0.8	1.7e-07	24.1	0.8	1.0	1	0	0	1	1	1	1	[51 - 13]	(REVERSE SENSE)
a_CmonF_UR_28_2	-	a_CmonF_UR_28_2	-	1.8e-07	24.1	2.3	1.8e-07	24.1	2.3	1.0	1	0	0	1	1	1	1	[38 - 3]	(REVERSE SENSE)
m_CmonF_UR_28_2	-	a_CmonF_UR_28_2	-	2.5e-07	23.7	2.0	2.5e-07	23.7	2.0	1.0	1	0	0	1	1	1	1	[38 - 3]	(REVERSE SENSE)
a_CmonF_UR_29_1	-	a_CmonF_UR_29_1	-	1.9e-06	21.6	7.0	1.9e-06	21.6	7.0	1.0	1	0	0	1	1	1	1	[46 - 17]	(REVERSE SENSE)
m_CmonF_UR_29_1	-	a_CmonF_UR_29_1	-	1.9e-06	21.6	7.0	1.9e-06	21.6	7.0	1.0	1	0	0	1	1	1	1	[46 - 17]	(REVERSE SENSE)
a_HmenM_UR_2_1	-	a_HmenM_UR_2_1	-	3.9e-06	19.2	0.8	3.9e-06	19.2	0.8	1.0	1	0	0	1	1	1	1	[30 - 59]	
m_HmenM_UR_2_1	-	a_HmenM_UR_2_1	-	3.9e-06	19.2	0.8	3.9e-06	19.2	0.8	1.0	1	0	0	1	1	1	1	[30 - 59]	
a_HmenM_UR_2_2	-	a_HmenM_UR_2_2	-	5.2e-05	14.9	0.1	5.2e-05	14.9	0.1	1.0	1	0	0	1	1	1	1	[60 - 31]	(REVERSE SENSE)
m_HmenM_UR_2_2	-	a_HmenM_UR_2_2	-	5.2e-05	14.9	0.1	5.2e-05	14.9	0.1	1.0	1	0	0	1	1	1	1	[60 - 31]	(REVERSE SENSE)
a_HmenM_UR_2_3	-	a_HmenM_UR_2_3	-	1.4e-08	27.6	3.2	1.4e-08	27.6	3.2	1.0	1	0	0	1	1	1	1	[49 - 2]	(REVERSE SENSE)
m_HmenM_UR_2_3	-	a_HmenM_UR_2_3	-	2.8e-08	26.8	3.2	2.8e-08	26.8	3.2	1.0	1	0	0	1	1	1	1	[49 - 2]	(REVERSE SENSE)
a_HmenM_UR_3_1	-	a_HmenM_UR_3_1	-	4.2e-11	34.2	2.9	4.3e-11	34.2	2.9	1.0	1	0	0	1	1	1	1	[8 - 64]	
m_HmenM_UR_3_1	-	a_HmenM_UR_3_1	-	1e-10	33.2	3.1	1e-10	33.2	3.1	1.0	1	0	0	1	1	1	1	[8 - 64]	
a_HmenM_UR_3_2	-	a_HmenM_UR_3_2	-	2.6e-17	55.8	6.2	2.7e-17	55.8	6.2	1.0	1	0	0	1	1	1	1	[94 - 177]	
m_HmenM_UR_3_2	-	a_HmenM_UR_3_2	-	4.9e-17	55.0	6.1	5.1e-17	55.0	6.1	1.0	1	0	0	1	1	1	1	[94 - 177]	
a_HmenM_UR_3_3	-	a_HmenM_UR_3_3	-	5.3e-05	14.0	0.1	5.3e-05	14.0	0.1	1.0	1	0	0	1	1	1	1	[310 - 339]	
m_HmenM_UR_3_3	-	a_HmenM_UR_3_3	-	0.00014	13.1	0.1	0.00014	13.1	0.1	1.1	1	0	0	1	1	1	1	[310 - 339]	
a_HmenM_UR_3_4	-	a_HmenM_UR_3_4	-	4.6e-16	51.5	7.4	4.8e-16	51.5	7.4	1.0	1	0	0	1	1	1	1	[386 - 460]	
m_HmenM_UR_3_4	-	a_HmenM_UR_3_4	-	4.6e-16	51.5	7.4	4.8e-16	51.5	7.4	1.0	1	0	0	1	1	1	1	[386 - 460]	
a_HmenM_UR_3_5	-	a_HmenM_UR_3_5	-	3.5e-36	117.8	16.0	3.8e-36	117.7	16.0	1.0	1	0	0	1	1	1	1	[424 - 597]	
m_HmenM_UR_3_5	-	a_HmenM_UR_3_5	-	7.8e-36	116.7	16.2	8.4e-36	116.6	16.2	1.0	1	0	0	1	1	1	1	[424 - 597]	
a_HmenM_UR_17_2	-	a_HmenM_UR_3_5	-	1.7e-07	27.3	10.5	1.7e-07	27.3	10.5	1.0	1	0	0	1	1	1	1	[64 - 132]	
m_HmenM_UR_17_2	-	a_HmenM_UR_3_5	-	3.5e-07	26.3	10.5	3.5e-07	26.3	10.5	1.0	1	0	0	1	1	1	1	[64 - 132]	
a_HmenM_UR_3_6	-	a_HmenM_UR_3_6	-	9.3e-23	72.6	0.8	9.8e-23	72.5	0.8	1.0	1	0	0	1	1	1	1	[506 - 610]	
m_HmenM_UR_3_6	-	a_HmenM_UR_3_6	-	1.7e-22	71.8	0.7	1.8e-22	71.7	0.7	1.0	1	0	0	1	1	1	1	[506 - 610]	
m_HmenM_UR_17_4	-	a_HmenM_UR_3_6	-	5.1e-13	43.2	0.1	5.8e-13	43.1	0.1	1.1	1	0	0	1	1	1	1	[44 - 172]	
a_HmenM_UR_17_4	-	a_HmenM_UR_3_6	-	5.4e-13	43.1	0.1	6.2e-13	43.0	0.1	1.1	1	0	0	1	1	1	1	[44 - 172]	
a_HmenM_UR_3_7	-	a_HmenM_UR_3_7	-	1.6e-127	418.0	16.8	1.8e-127	417.8	16.8	1.0	1	0	0	1	1	1	1	[75 - 638]	
m_HmenM_UR_3_7	-	a_HmenM_UR_3_7	-	3.5e-127	416.8	17.0	3.9e-127	416.7	17.0	1.0	1	0	0	1	1	1	1	[75 - 638]	
a_HmenM_UR_17_3	-	a_HmenM_UR_3_7	-	1.2e-09	33.5	1.2	1.2e-09	33.5	1.2	1.0	1	0	0	1	1	1	1	[48 - 137]	
m_HmenM_UR_17_3	-	a_HmenM_UR_3_7	-	1.2e-09	33.5	1.2	1.2e-09	33.5	1.2	1.0	1	0	0	1	1	1	1	[48 - 137]	
a_HmenM_UR_3_8	-	a_HmenM_UR_3_8	-	4.6e-11	34.3	0.6	4.7e-11	34.3	0.6	1.0	1	0	0	1	1	1	1	[613 - 666]	
m_HmenM_UR_3_8	-	a_HmenM_UR_3_8	-	9.7e-11	33.4	0.6	9.7e-11	33.4	0.6	1.0	1	0	0	1	1	1	1	[613 - 666]	
a_HmenM_UR_3_9	-	a_HmenM_UR_3_9	-	1.2e-06	19.7	0.4	1.2e-06	19.7	0.4	1.0	1	0	0	1	1	1	1	[638 - 667]	
m_HmenM_UR_3_9	-	a_HmenM_UR_3_9	-	1.2e-06	19.7	0.4	1.2e-06	19.7	0.4	1.0	1	0	0	1	1	1	1	[638 - 667]	
a_HmenM_UR_3_10	-	a_HmenM_UR_3_10	-	2.5e-07	23.8	3.9	2.5e-07	23.8	3.9	1.0	1	0	0	1	1	1	1	[656 - 621]	(REVERSE SENSE)
m_HmenM_UR_3_10	-	a_HmenM_UR_3_10	-	2.8e-07	23.7	3.4	2.8e-07	23.7	3.4	1.0	1	0	0	1	1	1	1	[656 - 621]	(REVERSE SENSE)
a_HmenM_UR_3_11	-	a_HmenM_UR_3_11	-	9.3e-06	18.2	0.6	9.3e-06	18.2	0.6	1.0	1	0	0	1	1	1	1	[645 - 613]	(REVERSE SENSE)
m_HmenM_UR_3_11	-	a_HmenM_UR_3_11	-	1.4e-05	17.8	0.5	1.4e-05	17.8	0.5	1.0	1	0	0	1	1	1	1	[645 - 613]	(REVERSE SENSE)
a_HmenM_UR_3_12	-	a_HmenM_UR_3_12	-	1.1e-11	36.2	1.3	1.1e-11	36.2	1.3	1.0	1	0	0	1	1	1	1	[652 - 596]	(REVERSE SENSE)
m_HmenM_UR_3_12	-	a_HmenM_UR_3_12	-	1.8e-11	35.6	1.0	1.8e-11	35.6	1.0	1.0	1	0	0	1	1	1	1	[652 - 596]	(REVERSE SENSE)
a_HmenM_UR_3_13	-	a_HmenM_UR_3_13	-	3.3e-14	46.0	10.7	3.4e-14	45.9	10.7	1.0	1	0	0	1	1	1	1	[609 - 541]	(REVERSE SENSE)
m_HmenM_UR_3_13	-	a_HmenM_UR_3_13	-	3.3e-14	46.0	10.7	3.4e-14	45.9	10.7	1.0	1	0	0	1	1	1	1	[609 - 541]	(REVERSE SENSE)
a_HmenM_UR_3_14	-	a_HmenM_UR_3_14	-	7.7e-27	86.2	0.2	8.3e-27	86.1	0.2	1.0	1	0	0	1	1	1	1	[586 - 458]	(REVERSE SENSE)
m_HmenM_UR_3_14	-	a_HmenM_UR_3_14	-	1.4e-26	85.4	0.2	1.5e-26	85.3	0.2	1.0	1	0	0	1	1	1	1	[586 - 458]	(REVERSE SENSE)
a_HmenM_UR_17_12	-	a_HmenM_UR_3_14	-	1.5e-05	20.9	0.0	1.5e-05	20.9	0.0	1.1	1	0	0	1	1	1	1	[88 - 29]	(REVERSE SENSE)
m_HmenM_UR_17_12	-	a_HmenM_UR_3_14	-	3e-05	20.0	0.0	3e-05	20.0	0.0	1.1	1	0	0	1	1	1	1	[88 - 29]	(REVERSE SENSE)
a_HmenM_UR_3_15	-	a_HmenM_UR_3_15	-	6.4e-07	23.0	3.0	6.4e-07	23.0	3.0	1.0	1	0	0	1	1	1	1	[450 - 412]	(REVERSE SENSE)
m_HmenM_UR_3_15	-	a_HmenM_UR_3_15	-	1.1e-06	22.3	2.7	1.1e-06	22.3	2.7	1.0	1	0	0	1	1	1	1	[450 - 412]	(REVERSE SENSE)
a_HmenM_UR_3_16	-	a_HmenM_UR_3_16	-	1.4e-26	86.2	5.9	1.5e-26	86.1	5.9	1.0	1	0	0	1	1	1	1	[530 - 408]	(REVERSE SENSE)
m_HmenM_UR_3_16	-	a_HmenM_UR_3_16	-	2.3e-26	85.4	5.7	2.5e-26	85.3	5.7	1.0	1	0	0	1	1	1	1	[530 - 408]	(REVERSE SENSE)
a_HmenM_UR_3_17	-	a_HmenM_UR_3_17	-	7.8e-05	14.7	0.5	7.8e-05	14.7	0.5	1.0	1	0	0	1	1	1	1	[349 - 317]	(REVERSE SENSE)
m_HmenM_UR_3_17	-	a_HmenM_UR_3_17	-	0.00013	14.2	0.3	0.00013	14.2	0.3	1.0	1	0	0	1	1	1	1	[349 - 317]	(REVERSE SENSE)
a_HmenM_UR_3_18	-	a_HmenM_UR_3_18	-	4.3e-06	18.3	0.2	4.3e-06	18.3	0.2	1.0	1	0	0	1	1	1	1	[329 - 294]	(REVERSE SENSE)
m_HmenM_UR_3_18	-	a_HmenM_UR_3_18	-	1e-05	17.4	0.2	1e-05	17.4	0.2	1.0	1	0	0	1	1	1	1	[329 - 294]	(REVERSE SENSE)
a_HmenM_UR_3_19	-	a_HmenM_UR_3_19	-	1.8e-13	42.2	2.0	1.8e-13	42.2	2.0	1.0	1	0	0	1	1	1	1	[214 - 143]	(REVERSE SENSE)
m_HmenM_UR_3_19	-	a_HmenM_UR_3_19	-	1.8e-13	42.2	2.0	1.8e-13	42.2	2.0	1.0	1	0	0	1	1	1	1	[214 - 143]	(REVERSE SENSE)
a_HmenM_UR_3_20	-	a_HmenM_UR_3_20	-	4.5e-37	119.8	0.9	4.9e-37	119.6	0.9	1.0	1	0	0	1	1	1	1	[284 - 123]	(REVERSE SENSE)
m_HmenM_UR_3_20	-	a_HmenM_UR_3_20	-	9.2e-37	118.8	1.1	9.9e-37	118.7	1.1	1.0	1	0	0	1	1	1	1	[284 - 123]	(REVERSE SENSE)
a_HmenM_UR_3_21	-	a_HmenM_UR_3_21	-	2.8e-06	20.7	3.9	2.8e-06	20.7	3.9	1.0	1	0	0	1	1	1	1	[133 - 98]	(REVERSE SENSE)
m_HmenM_UR_3_21	-	a_HmenM_UR_3_21	-	2.8e-06	20.7	3.9	2.8e-06	20.7	3.9	1.0	1	0	0	1	1	1	1	[133 - 98]	(REVERSE SENSE)
a_HmenM_UR_3_22	-	a_HmenM_UR_3_22	-	9.4e-13	41.1	4.9	9.7e-13	41.0	4.9	1.0	1	0	0	1	1	1	1	[95 - 27]	(REVERSE SENSE)
m_HmenM_UR_3_22	-	a_HmenM_UR_3_22	-	1.1e-12	40.8	4.3	1.2e-12	40.8	4.3	1.0	1	0	0	1	1	1	1	[95 - 27]	(REVERSE SENSE)
a_HmenM_UR_3_23	-	a_HmenM_UR_3_23	-	5.2e-18	58.4	12.6	5.5e-18	58.3	12.6	1.0	1	0	0	1	1	1	1	[94 - 2]	(REVERSE SENSE)
m_HmenM_UR_3_23	-	a_HmenM_UR_3_23	-	6.7e-18	58.0	12.3	7e-18	58.0	12.3	1.0	1	0	0	1	1	1	1	[94 - 2]	(REVERSE SENSE)
a_HmenM_UR_3_24	-	a_HmenM_UR_3_24	-	1.9e-05	17.5	2.6	1.9e-05	17.5	2.6	1.0	1	0	0	1	1	1	1	[39 - 1]	(REVERSE SENSE)
m_HmenM_UR_3_24	-	a_HmenM_UR_3_24	-	2.7e-05	17.1	2.4	2.7e-05	17.1	2.4	1.0	1	0	0	1	1	1	1	[39 - 1]	(REVERSE SENSE)
a_HmenM_UR_4_1	-	a_HmenM_UR_4_1	-	4.5e-06	18.6	0.2	4.5e-06	18.6	0.2	1.0	1	0	0	1	1	1	1	[38 - 3]	(REVERSE SENSE)
m_HmenM_UR_4_1	-	a_HmenM_UR_4_1	-	7.9e-06	18.0	0.1	7.9e-06	18.0	0.1	1.0	1	0	0	1	1	1	1	[38 - 3]	(REVERSE SENSE)
a_HmenM_UR_6_1	-	a_HmenM_UR_6_1	-	1.2e-05	17.8	2.0	1.2e-05	17.8	2.0	1.0	1	0	0	1	1	1	1	[31 - 2]	(REVERSE SENSE)
m_HmenM_UR_6_1	-	a_HmenM_UR_6_1	-	1.7e-05	17.5	1.4	1.7e-05	17.5	1.4	1.0	1	0	0	1	1	1	1	[31 - 2]	(REVERSE SENSE)
a_HmenM_UR_14_1	-	a_HmenM_UR_14_1	-	5e-12	37.1	2.5	5.1e-12	37.1	2.5	1.0	1	0	0	1	1	1	1	[12 - 68]	
m_HmenM_UR_14_1	-	a_HmenM_UR_14_1	-	5e-12	37.1	2.5	5.1e-12	37.1	2.5	1.0	1	0	0	1	1	1	1	[12 - 68]	
a_HmenM_UR_14_2	-	a_HmenM_UR_14_2	-	6e-06	18.0	0.3	6e-06	18.0	0.3	1.0	1	0	0	1	1	1	1	[63 - 31]	(REVERSE SENSE)
m_HmenM_UR_14_2	-	a_HmenM_UR_14_2	-	1.3e-05	17.2	0.2	1.3e-05	17.2	0.2	1.1	1	0	0	1	1	1	1	[63 - 31]	(REVERSE SENSE)
a_HmenM_UR_14_3	-	a_HmenM_UR_14_3	-	3.4e-15	48.8	4.1	3.5e-15	48.7	4.1	1.0	1	0	0	1	1	1	1	[65 - 3]	(REVERSE SENSE)

m_HmenM_UR_14_3	-	a_HmenM_UR_14_3	-	5.7e-15	48.1	4.1	5.8e-15	48.1	4.1	1.0	1	0	0	1	1	1	1	[65 - 3] (REVERSE SENSE)
a_HmenM_UR_17_1	-	a_HmenM_UR_17_1	-	2.5e-07	21.5	0.4	2.5e-07	21.5	0.4	1.0	1	0	0	1	1	1	1	[10 - 45]
m_HmenM_UR_17_1	-	a_HmenM_UR_17_1	-	2.5e-07	21.5	0.4	2.5e-07	21.5	0.4	1.0	1	0	0	1	1	1	1	[10 - 45]
a_HmenM_UR_17_2	-	a_HmenM_UR_17_2	-	9.3e-13	42.1	11.8	9.7e-13	42.1	11.8	1.0	1	0	0	1	1	1	1	[64 - 132]
m_HmenM_UR_17_2	-	a_HmenM_UR_17_2	-	1.8e-12	41.3	11.7	1.8e-12	41.2	11.7	1.0	1	0	0	1	1	1	1	[64 - 132]
a_HmenM_UR_3_5	-	a_HmenM_UR_17_2	-	3.6e-08	28.5	8.2	3.6e-08	28.5	8.2	2.6	2	1	1	3	3	3	1	[424 - 597]
m_HmenM_UR_3_5	-	a_HmenM_UR_17_2	-	3.6e-08	28.5	8.2	3.6e-08	28.5	8.2	2.6	2	1	1	3	3	3	1	[424 - 597]
a_HmenM_UR_17_3	-	a_HmenM_UR_17_3	-	3.8e-18	57.4	2.7	3.9e-18	57.4	2.7	1.0	1	0	0	1	1	1	1	[48 - 137]
m_HmenM_UR_17_3	-	a_HmenM_UR_17_3	-	3.8e-18	57.4	2.7	3.9e-18	57.4	2.7	1.0	1	0	0	1	1	1	1	[48 - 137]
a_HmenM_UR_3_7	-	a_HmenM_UR_17_3	-	5.1e-10	33.5	0.6	5.1e-10	33.5	0.6	2.9	3	0	0	3	3	3	1	[75 - 638]
m_HmenM_UR_3_7	-	a_HmenM_UR_17_3	-	5.1e-10	33.5	0.6	5.1e-10	33.5	0.6	2.9	3	0	0	3	3	3	1	[75 - 638]
a_HmenM_UR_17_4	-	a_HmenM_UR_17_4	-	7.3e-29	92.2	0.9	7.8e-29	92.1	0.9	1.0	1	0	0	1	1	1	1	[44 - 172]
m_HmenM_UR_17_4	-	a_HmenM_UR_17_4	-	1.4e-28	91.3	0.9	1.5e-28	91.2	0.9	1.0	1	0	0	1	1	1	1	[44 - 172]
a_HmenM_UR_3_6	-	a_HmenM_UR_17_4	-	5.9e-13	43.4	0.2	6.1e-13	43.4	0.2	1.0	1	0	0	1	1	1	1	[506 - 610]
m_HmenM_UR_3_6	-	a_HmenM_UR_17_4	-	6.1e-13	43.4	0.2	6.1e-13	43.4	0.2	1.0	1	0	0	1	1	1	1	[506 - 610]
a_HmenM_UR_17_5	-	a_HmenM_UR_17_5	-	3.1e-06	18.1	1.0	3.1e-06	18.1	1.0	1.0	1	0	0	1	1	1	1	[190 - 222]
m_HmenM_UR_17_5	-	a_HmenM_UR_17_5	-	3.1e-06	18.1	1.0	3.1e-06	18.1	1.0	1.0	1	0	0	1	1	1	1	[190 - 222]
a_HmenM_UR_17_6	-	a_HmenM_UR_17_6	-	1.1e-09	29.5	2.9	1.1e-09	29.5	2.9	1.0	1	0	0	1	1	1	1	[225 - 272]
m_HmenM_UR_17_6	-	a_HmenM_UR_17_6	-	1.1e-09	29.5	2.9	1.1e-09	29.5	2.9	1.0	1	0	0	1	1	1	1	[225 - 272]
a_HmenM_UR_17_7	-	a_HmenM_UR_17_7	-	3.9e-07	22.8	4.6	3.9e-07	22.8	4.6	1.0	1	0	0	1	1	1	1	[266 - 225] (REVERSE SENSE)
m_HmenM_UR_17_7	-	a_HmenM_UR_17_7	-	4.8e-07	22.6	3.9	4.8e-07	22.6	3.9	1.0	1	0	0	1	1	1	1	[266 - 225] (REVERSE SENSE)
a_HmenM_UR_17_8	-	a_HmenM_UR_17_8	-	2.8e-09	30.0	4.5	2.8e-09	30.0	4.5	1.0	1	0	0	1	1	1	1	[268 - 221] (REVERSE SENSE)
m_HmenM_UR_17_8	-	a_HmenM_UR_17_8	-	3.6e-09	29.7	3.9	3.6e-09	29.7	3.9	1.0	1	0	0	1	1	1	1	[268 - 221] (REVERSE SENSE)
a_HmenM_UR_17_9	-	a_HmenM_UR_17_9	-	2.2e-11	36.7	4.5	2.3e-11	36.7	4.5	1.0	1	0	0	1	1	1	1	[237 - 175] (REVERSE SENSE)
m_HmenM_UR_17_9	-	a_HmenM_UR_17_9	-	2.3e-11	36.7	3.8	2.3e-11	36.7	3.8	1.0	1	0	0	1	1	1	1	[237 - 175] (REVERSE SENSE)
a_HmenM_UR_17_10	-	a_HmenM_UR_17_10	-	7.4e-13	41.7	7.8	7.6e-13	41.7	7.8	1.0	1	0	0	1	1	1	1	[215 - 150] (REVERSE SENSE)
m_HmenM_UR_17_10	-	a_HmenM_UR_17_10	-	1.1e-12	41.2	7.7	1.1e-12	41.2	7.7	1.0	1	0	0	1	1	1	1	[215 - 150] (REVERSE SENSE)
a_HmenM_UR_17_11	-	a_HmenM_UR_17_11	-	3.8e-15	49.7	10.5	4e-15	49.6	10.5	1.0	1	0	0	1	1	1	1	[128 - 48] (REVERSE SENSE)
m_HmenM_UR_17_11	-	a_HmenM_UR_17_11	-	3.8e-15	49.7	10.5	4e-15	49.6	10.5	1.0	1	0	0	1	1	1	1	[128 - 48] (REVERSE SENSE)
a_HmenM_UR_17_12	-	a_HmenM_UR_17_12	-	7.1e-12	37.5	0.1	7.2e-12	37.4	0.1	1.0	1	0	0	1	1	1	1	[88 - 29] (REVERSE SENSE)
m_HmenM_UR_17_12	-	a_HmenM_UR_17_12	-	1.5e-11	36.5	0.1	1.5e-11	36.5	0.1	1.0	1	0	0	1	1	1	1	[88 - 29] (REVERSE SENSE)
m_HmenM_UR_3_14	-	a_HmenM_UR_17_12	-	4.4e-06	21.1	0.2	9.8e-06	20.1	0.1	1.6	1	1	0	1	1	1	1	[586 - 458] (REVERSE SENSE)
a_HmenM_UR_3_14	-	a_HmenM_UR_17_12	-	4.4e-06	21.1	0.2	9.8e-06	20.1	0.1	1.6	1	1	0	1	1	1	1	[586 - 458] (REVERSE SENSE)
a_HmenM_UR_17_13	-	a_HmenM_UR_17_13	-	2.2e-08	26.6	0.6	2.2e-08	26.6	0.6	1.0	1	0	0	1	1	1	1	[41 - 3] (REVERSE SENSE)
m_HmenM_UR_17_13	-	a_HmenM_UR_17_13	-	4.4e-08	25.8	0.4	4.4e-08	25.8	0.4	1.0	1	0	0	1	1	1	1	[41 - 3] (REVERSE SENSE)
a_HmenM_UR_17_14	-	a_HmenM_UR_17_14	-	4.6e-21	68.6	9.7	4.9e-21	68.5	9.7	1.0	1	0	0	1	1	1	1	[102 - 1] (REVERSE SENSE)
m_HmenM_UR_17_14	-	a_HmenM_UR_17_14	-	4.6e-21	68.6	9.7	4.9e-21	68.5	9.7	1.0	1	0	0	1	1	1	1	[102 - 1] (REVERSE SENSE)
a_HmenM_UR_20_1	-	a_HmenM_UR_20_1	-	1.5e-08	26.8	0.5	1.5e-08	26.8	0.5	1.0	1	0	0	1	1	1	1	[50 - 3] (REVERSE SENSE)
m_HmenM_UR_20_1	-	a_HmenM_UR_20_1	-	2.4e-08	26.2	0.4	2.4e-08	26.2	0.4	1.0	1	0	0	1	1	1	1	[50 - 3] (REVERSE SENSE)
a_HmenM_UR_21_1	-	a_HmenM_UR_21_1	-	7.1e-06	16.5	0.6	7.1e-06	16.5	0.6	1.0	1	0	0	1	1	1	1	[1 - 30]
m_HmenM_UR_21_1	-	a_HmenM_UR_21_1	-	7.1e-06	16.5	0.6	7.1e-06	16.5	0.6	1.0	1	0	0	1	1	1	1	[1 - 30]
a_HmenM_UR_21_2	-	a_HmenM_UR_21_2	-	1.2e-09	29.5	0.6	1.2e-09	29.5	0.6	1.0	1	0	0	1	1	1	1	[52 - 96]
m_HmenM_UR_21_2	-	a_HmenM_UR_21_2	-	2.4e-09	28.7	0.5	2.4e-09	28.7	0.5	1.0	1	0	0	1	1	1	1	[52 - 96]
a_HmenM_UR_21_3	-	a_HmenM_UR_21_3	-	2e-13	42.9	8.7	2e-13	42.8	8.7	1.0	1	0	0	1	1	1	1	[109 - 174]
m_HmenM_UR_21_3	-	a_HmenM_UR_21_3	-	2e-13	42.9	8.7	2e-13	42.8	8.7	1.0	1	0	0	1	1	1	1	[109 - 174]
a_HmenM_UR_21_4	-	a_HmenM_UR_21_4	-	5.3e-34	109.6	3.6	5.8e-34	109.5	3.6	1.0	1	0	0	1	1	1	1	[21 - 176]
m_HmenM_UR_21_4	-	a_HmenM_UR_21_4	-	1.3e-33	108.5	3.9	1.4e-33	108.4	3.9	1.0	1	0	0	1	1	1	1	[21 - 176]
a_HmenM_UR_21_5	-	a_HmenM_UR_21_5	-	1.8e-07	24.0	2.1	1.8e-07	24.0	2.1	1.0	1	0	0	1	1	1	1	[167 - 126] (REVERSE SENSE)
m_HmenM_UR_21_5	-	a_HmenM_UR_21_5	-	3.1e-07	23.4	2.0	3.1e-07	23.4	2.0	1.0	1	0	0	1	1	1	1	[167 - 126] (REVERSE SENSE)
a_HmenM_UR_21_6	-	a_HmenM_UR_21_6	-	6.9e-25	80.7	9.1	7.4e-25	80.6	9.1	1.0	1	0	0	1	1	1	1	[163 - 50] (REVERSE SENSE)
m_HmenM_UR_21_6	-	a_HmenM_UR_21_6	-	1.4e-24	79.7	9.1	1.5e-24	79.6	9.1	1.0	1	0	0	1	1	1	1	[163 - 50] (REVERSE SENSE)
a_HmenM_UR_21_7	-	a_HmenM_UR_21_7	-	3.8e-07	22.0	2.1	3.8e-07	22.0	2.1	1.0	1	0	0	1	1	1	1	[37 - 2] (REVERSE SENSE)
m_HmenM_UR_21_7	-	a_HmenM_UR_21_7	-	1.1e-06	20.9	2.3	1.1e-06	20.9	2.3	1.0	1	0	0	1	1	1	1	[37 - 2] (REVERSE SENSE)
a_HmenM_UR_21_8	-	a_HmenM_UR_21_8	-	6e-39	126.3	13.3	6.5e-39	126.2	13.3	1.0	1	0	0	1	1	1	1	[174 - 1] (REVERSE SENSE)
m_HmenM_UR_21_8	-	a_HmenM_UR_21_8	-	9.8e-39	125.6	13.4	1.1e-38	125.5	13.4	1.0	1	0	0	1	1	1	1	[174 - 1] (REVERSE SENSE)
a_HmenM_UR_25_1	-	a_HmenM_UR_25_1	-	1.1e-07	24.2	3.2	1.1e-07	24.2	3.2	1.0	1	0	0	1	1	1	1	[3 - 41]
m_HmenM_UR_25_1	-	a_HmenM_UR_25_1	-	3e-07	23.0	3.5	3e-07	23.0	3.5	1.0	1	0	0	1	1	1	1	[3 - 41]
a_HmenM_UR_25_2	-	a_HmenM_UR_25_2	-	5.8e-07	21.3	0.8	5.8e-07	21.3	0.8	1.0	1	0	0	1	1	1	1	[40 - 2] (REVERSE SENSE)
m_HmenM_UR_25_2	-	a_HmenM_UR_25_2	-	1.2e-06	20.5	0.7	1.2e-06	20.5	0.7	1.0	1	0	0	1	1	1	1	[40 - 2] (REVERSE SENSE)
a_HmenM_UR_26_1	-	a_HmenM_UR_26_1	-	2.4e-05	16.1	0.3	2.4e-05	16.1	0.3	1.0	1	0	0	1	1	1	1	[34 - 2] (REVERSE SENSE)
m_HmenM_UR_26_1	-	a_HmenM_UR_26_1	-	4.3e-05	15.5	0.2	4.3e-05	15.5	0.2	1.0	1	0	0	1	1	1	1	[34 - 2] (REVERSE SENSE)
a_HmenM_UR_26_2	-	a_HmenM_UR_26_2	-	3.8e-15	48.7	3.3	3.9e-15	48.6	3.3	1.0	1	0	0	1	1	1	1	[78 - 1] (REVERSE SENSE)
m_HmenM_UR_26_2	-	a_HmenM_UR_26_2	-	7.3e-15	47.8	3.2	7.5e-15	47.8	3.2	1.0	1	0	0	1	1	1	1	[78 - 1] (REVERSE SENSE)
a_HmenM_UR_28_1	-	a_HmenM_UR_28_1	-	6.4e-08	23.8	0.2	6.4e-08	23.8	0.2	1.0	1	0	0	1	1	1	1	[2 - 40]
m_HmenM_UR_28_1	-	a_HmenM_UR_28_1	-	1.4e-07	22.9	0.2	1.4e-07	22.9	0.2	1.0	1	0	0	1	1	1	1	[2 - 40]
a_CmonM_UR_1_1	-	a_CmonM_UR_1_1	-	2.6e-07	21.6	0.1	2.6e-07	21.6	0.1	1.0	1	0	0	1	1	1	1	[20 - 55]
m_CmonM_UR_1_1	-	a_CmonM_UR_1_1	-	6.1e-07	20.7	0.1	6.1e-07	20.7	0.1	1.0	1	0	0	1	1	1	1	[20 - 55]
a_CmonM_UR_1_2	-	a_CmonM_UR_1_2	-	9.6e-09	26.4	0.2	9.6e-09	26.4	0.2	1.0	1	0	0	1	1	1	1	[12 - 56]
m_CmonM_UR_1_2	-	a_CmonM_UR_1_2	-	2.2e-08	25.5	0.2	2.2e-08	25.5	0.2	1.0	1	0	0	1	1	1	1	[12 - 56]
a_CmonM_UR_1_3	-	a_CmonM_UR_1_3	-	2.8e-08	25.3	0.2	2.8e-08	25.3	0.2	1.0	1	0	0	1	1	1	1	[37 - 2] (REVERSE SENSE)
m_CmonM_UR_1_3	-	a_CmonM_UR_1_3	-	6.1e-08	24.4	0.2	6.1e-08	24.4	0.2	1.0	1	0	0	1	1	1	1	[37 - 2] (REVERSE SENSE)
a_CmonM_UR_1_4	-	a_CmonM_UR_1_4	-	9.2e-06	17.9	0.5	9.2e-06	17.9	0.5	1.0	1	0	0	1	1	1	1	[30 - 1] (REVERSE SENSE)
m_CmonM_UR_1_4	-	a_CmonM_UR_1_4	-	1.9e-05	17.1	0.4	1.9e-05	17.1	0.4	1.0	1	0	0	1	1	1	1	[30 - 1] (REVERSE SENSE)
a_CmonM_UR_2_1	-	a_CmonM_UR_2_1	-	2e-30	98.2	8.0	2.1e-30	98.1	8.0	1.0	1	0	0	1	1	1	1	[4 - 144]
m_CmonM_UR_2_1	-	a_CmonM_UR_2_1	-	2e-30	98.2	8.0	2.1e-30	98.1	8.0	1.0	1	0	0	1	1	1	1	[4 - 144]
a_CmonM_UR_2_2	-	a_CmonM_UR_2_2	-	8.5e-15	46.6	1.0	8.8e-15	46.5	1.0	1.0	1	0	0	1	1	1	1	[135 - 67] (REVERSE SENSE)
m_CmonM_UR_2_2	-	a_CmonM_UR_2_2	-	1.4e-14	46.0	0.9	1.4e-14	45.9	0.9	1.0	1	0	0	1	1	1	1	[135 - 67] (REVERSE SENSE)
a_CmonM_UR_2_3	-	a_CmonM_UR_2_3	-	6.8e-18	57.2	0.9	7.2e-18	57.1	0.9	1.0	1	0	0	1	1	1	1	[137 - 51] (REVERSE SENSE)
m_CmonM_UR_2_3	-	a_CmonM_UR_2_3	-	1.4e-17	56.2	0.8	1.4e-17	56.2	0.8	1.0	1	0	0	1	1	1	1	[137 - 51] (REVERSE SENSE)
a_CmonM_UR_2_4	-	a_CmonM_UR_2_4	-	1.5e-06	19.3	0.3	1.5e-06	19.3	0.3	1.0	1	0	0	1	1	1	1	[67 - 32] (REVERSE SENSE)
m_CmonM_UR_2_4	-	a_CmonM_UR_2_4	-	1.5e-06	19.3	0.3	1.5e-06	19.3	0.3	1.0	1	0	0	1	1	1	1	[67 - 32] (REVERSE SENSE)
a_CmonM_UR_2_5	-	a_CmonM_UR_2_5	-	4.1e-10	31.6	0.6	4.2e-10	31.6	0.6	1.0	1	0	0	1	1	1	1	[48 - 1] (REVERSE SENSE)
m_CmonM_UR_2_5	-	a_CmonM_UR_2_5	-	4.1e-10	31.6	0.6	4.2e-10	31.6	0.6	1.0	1	0	0	1	1	1	1	[48 - 1] (REVERSE SENSE)
a_CmonM_UR_15_1	-	a_CmonM_UR_15_1	-	1.1e-05	16.6	0.3	1.1e-05	16.6	0.3	1.0	1	0	0	1	1	1	1	[9 - 38]
m_CmonM_UR_15_1	-	a_CmonM_UR_15_1	-	2.9e-05	15.6	0.4	2.9e-05	15.6	0.4	1.0	1	0	0	1	1	1	1	[9 - 38]
a_CmonM_UR_15_2	-	a_CmonM_UR_15_2	-	6.8e-06	18.2	0.7	6.8e-06	18.2	0.7	1.0	1	0	0	1	1	1	1	[31 - 2] (REVERSE SENSE)
m_CmonM_UR_15_2	-	a_CmonM_UR_15_2	-	1.5e-05	17.3	0.6	1.5e-05	17.3	0.6	1.1	1	0	0	1	1	1	1	[31 - 2] (REVERSE SENSE)
a_CmonM_UR_16_1	-	a_CmonM_UR_16_1	-	1.1e-06	20.2	0.0	1.1e-06	20.2	0.0	1.0	1	0	0	1	1	1	1	[13 - 45]
m_CmonM_UR_16_1	-	a_CmonM_UR_16_1	-	1.1e-06	20.2	0.0	1.1e-06	20.2	0.0	1.0	1	0	0	1	1	1	1	[13 - 45]
a_CmonM_UR_16_5	-	a_CmonM_UR_16_1	-	5.8e-06	18.4	0.1	3.7e-05	16.3	0.0	2.1	2	1	0	2	2	2	1	[147 - 239]
m_CmonM_UR_16_5	-	a_CmonM_UR_16_1	-	5.8e-06	18.4	0.1	3.7e-05	16.3	0.0	2.1	2	1	0	2	2	2	1	[147 - 239]
a_CmonM_UR_16_2	-	a_CmonM_UR_16_2	-	4.3e-09	28.7	2.2	4.3e-09	28.7	2.2	1.0	1	0	0	1	1	1	1	[78 - 125]
a_CmonM_UR_16_6	-	a_CmonM_UR_16_2	-	4.3e-09	28.7	2.2	4.3e-09	28.7	2.2	1.0	1	0	0	1	1	1	1	[272 - 319]
m_CmonM_UR_16_2	-	a_CmonM_UR_16_2	-	4.3e-09	28.7	2.2	4.3e-09	28.7	2.2	1.0	1	0	0	1	1	1	1	[78 - 125]
m_CmonM_UR_16_6	-	a_CmonM_UR_16_2	-	4.3e-09	28.7	2.2	4.3e-09	28.7	2.2	1.0	1	0	0	1	1	1	1	[272 - 319]
a_CmonM_UR_16_3	-	a_CmonM_UR_16_3	-	1.7e-11	36.3	4.1	1.7e-11	36.3	4.1	1.0	1	0	0	1	1	1	1	[94 - 147]
a_CmonM_UR_16_7	-	a_CmonM_UR_16_3	-	1.7e-11	36.3	4.1	1.7e-11	36.3	4.1	1.0	1	0	0	1	1	1	1	[288 - 341]
m_CmonM_UR_16_3	-	a_CmonM_UR_16_3	-	4.5e-11	35.1	4.3	4.6e-11	35.1	4.3	1.0	1	0	0	1	1	1	1	[94 - 147]
m_CmonM_UR_16_7	-	a_CmonM_UR_16_3	-	4.5e-11	35.1	4.3	4.6e-11	35.1	4.3	1.0	1	0	0	1	1	1	1	[288 - 341]
a_CmonM_UR_16_4	-	a_CmonM_UR_16_4	-	5.7e-21	66.5	1.7	6.1e-21	66.4	1.7	1.0	1	0	0	1	1	1	1	[113 - 214]
m_CmonM_UR_16_4	-	a_CmonM_UR_16_4	-	5.7e-21	66.5	1.7	6.1e-21	66.4	1.7	1.0	1	0	0	1	1	1	1	[113 - 214]
a_CmonM_UR_16_8	-	a_CmonM_UR_16_4	-	1.2e-12	41.6	1.6	1.2e-12	41.6	1.6	1.0	1	0	0	1	1	1	1	[307 - 378]
m_CmonM_UR_16_8	-	a_CmonM_UR_16_4	-	1.2e-12	41.6	1.6	1.2e-12	41.6	1.6	1.0	1	0	0	1	1	1	1	[307 - 378]
a_CmonM_UR_16_5	-	a_CmonM_UR_16_5	-	3.1e-19	60.9	0.8	3.3e-19	60.9	0.8	1.0	1	0	0	1	1	1	1	[147 - 239]
m_CmonM_UR_16_5	-	a_CmonM_UR_16_5	-	3.1e-19	60.9	0.8	3.3e-19	60.9	0.8	1.0	1	0	0	1	1	1	1	[147 - 239]
a_CmonM_UR_16_1	-	a_CmonM_UR_16_5	-	0.00016	17.3	0.0	0.00016	17.3	0.0	1.0	1	0	0	1	1	1	1	[13 - 45]
m_CmonM_UR_16_1	-	a_CmonM_UR_16_5	-	0.00016	17.3	0.0	0.00016	17.3	0.0	1.0	1	0	0	1	1	1	1	[13 - 45]
a_CmonM_UR_16_9	-	a_CmonM_UR_16_5	-	0.00018	17.1	0.2	0.00021	16.9	0.2	1.4	1	1	0	1	1	1	1	[341 - 439]
m_CmonM_UR_16_9	-	a_CmonM_UR_16_5	-	0.00018	17.1	0.2	0.00021	16.9	0.2	1.4	1	1	0	1	1	1	1	[341 - 439]

a_CmonM_UR_16_2	-	a_CmonM_UR_16_6	-	4.3e-09	28.7	2.2	4.3e-09	28.7	2.2	1.0	1	0	0	1	1	1	1	[78 - 125]
a_CmonM_UR_16_6	-	a_CmonM_UR_16_6	-	4.3e-09	28.7	2.2	4.3e-09	28.7	2.2	1.0	1	0	0	1	1	1	1	[272 - 319]
m_CmonM_UR_16_2	-	a_CmonM_UR_16_6	-	4.3e-09	28.7	2.2	4.3e-09	28.7	2.2	1.0	1	0	0	1	1	1	1	[78 - 125]
m_CmonM_UR_16_6	-	a_CmonM_UR_16_6	-	4.3e-09	28.7	2.2	4.3e-09	28.7	2.2	1.0	1	0	0	1	1	1	1	[272 - 319]
a_CmonM_UR_16_3	-	a_CmonM_UR_16_7	-	1.7e-11	36.3	4.1	1.7e-11	36.3	4.1	1.0	1	0	0	1	1	1	1	[94 - 147]
a_CmonM_UR_16_7	-	a_CmonM_UR_16_7	-	1.7e-11	36.3	4.1	1.7e-11	36.3	4.1	1.0	1	0	0	1	1	1	1	[288 - 341]
m_CmonM_UR_16_3	-	a_CmonM_UR_16_7	-	4.5e-11	35.1	4.3	4.6e-11	35.1	4.3	1.0	1	0	0	1	1	1	1	[94 - 147]
m_CmonM_UR_16_7	-	a_CmonM_UR_16_7	-	4.5e-11	35.1	4.3	4.6e-11	35.1	4.3	1.0	1	0	0	1	1	1	1	[288 - 341]
a_CmonM_UR_16_8	-	a_CmonM_UR_16_8	-	4.6e-14	44.2	1.8	4.8e-14	44.2	1.8	1.0	1	0	0	1	1	1	1	[307 - 378]
m_CmonM_UR_16_8	-	a_CmonM_UR_16_8	-	4.6e-14	44.2	1.8	4.8e-14	44.2	1.8	1.0	1	0	0	1	1	1	1	[307 - 378]
a_CmonM_UR_16_4	-	a_CmonM_UR_16_8	-	4.7e-13	41.3	1.5	6.5e-13	40.9	1.5	1.2	1	0	0	1	1	1	1	[113 - 214]
m_CmonM_UR_16_4	-	a_CmonM_UR_16_8	-	4.7e-13	41.3	1.5	6.5e-13	40.9	1.5	1.2	1	0	0	1	1	1	1	[113 - 214]
a_CmonM_UR_16_9	-	a_CmonM_UR_16_9	-	1.6e-19	61.9	1.9	1.7e-19	61.8	1.9	1.0	1	0	0	1	1	1	1	[341 - 439]
m_CmonM_UR_16_9	-	a_CmonM_UR_16_9	-	1.6e-19	61.9	1.9	1.7e-19	61.8	1.9	1.0	1	0	0	1	1	1	1	[341 - 439]
a_CmonM_UR_16_5	-	a_CmonM_UR_16_9	-	0.00018	17.1	0.3	0.0002	17.0	0.3	1.4	1	1	0	1	1	1	1	[147 - 239]
m_CmonM_UR_16_5	-	a_CmonM_UR_16_9	-	0.00018	17.1	0.3	0.0002	17.0	0.3	1.4	1	1	0	1	1	1	1	[147 - 239]
a_CmonM_UR_16_10	-	a_CmonM_UR_16_10	-	6e-10	30.6	0.1	6e-10	30.6	0.1	1.0	1	0	0	1	1	1	1	[450 - 497]
m_CmonM_UR_16_10	-	a_CmonM_UR_16_10	-	1.2e-09	29.8	0.1	1.2e-09	29.8	0.1	1.0	1	0	0	1	1	1	1	[450 - 497]
a_CmonM_UR_16_11	-	a_CmonM_UR_16_11	-	1.6e-11	36.1	1.1	1.6e-11	36.0	1.1	1.0	1	0	0	1	1	1	1	[466 - 519]
m_CmonM_UR_16_11	-	a_CmonM_UR_16_11	-	4.5e-11	34.8	1.4	4.5e-11	34.8	1.4	1.0	1	0	0	1	1	1	1	[466 - 519]
a_CmonM_UR_16_12	-	a_CmonM_UR_16_12	-	3.1e-08	25.4	1.1	3.1e-08	25.3	1.1	1.0	1	0	0	1	1	1	1	[510 - 463] (REVERSE SENSE)
m_CmonM_UR_16_12	-	a_CmonM_UR_16_12	-	5.4e-08	24.7	0.9	5.4e-08	24.7	0.9	1.0	1	0	0	1	1	1	1	[510 - 463] (REVERSE SENSE)
a_CmonM_UR_16_13	-	a_CmonM_UR_16_13	-	8.2e-18	56.9	4.3	8.6e-18	56.9	4.3	1.0	1	0	0	1	1	1	1	[518 - 438] (REVERSE SENSE)
m_CmonM_UR_16_13	-	a_CmonM_UR_16_13	-	8.2e-18	56.9	4.3	8.6e-18	56.9	4.3	1.0	1	0	0	1	1	1	1	[518 - 438] (REVERSE SENSE)
a_CmonM_UR_16_14	-	a_CmonM_UR_16_14	-	4.4e-07	22.3	0.3	4.4e-07	22.3	0.3	1.0	1	0	0	1	1	1	1	[416 - 381] (REVERSE SENSE)
m_CmonM_UR_16_14	-	a_CmonM_UR_16_14	-	7.6e-07	21.7	0.3	7.6e-07	21.7	0.3	1.0	1	0	0	1	1	1	1	[416 - 381] (REVERSE SENSE)
a_CmonM_UR_16_15	-	a_CmonM_UR_16_15	-	1.9e-12	39.3	0.6	1.9e-12	39.3	0.6	1.0	1	0	0	1	1	1	1	[432 - 370] (REVERSE SENSE)
m_CmonM_UR_16_15	-	a_CmonM_UR_16_15	-	3.5e-12	38.6	0.4	3.5e-12	38.6	0.4	1.0	1	0	0	1	1	1	1	[432 - 370] (REVERSE SENSE)
a_CmonM_UR_16_16	-	a_CmonM_UR_16_16	-	3.7e-07	22.0	0.5	3.7e-07	22.0	0.5	1.0	1	0	0	1	1	1	1	[370 - 335] (REVERSE SENSE)
a_CmonM_UR_16_21	-	a_CmonM_UR_16_16	-	3.7e-07	22.0	0.5	3.7e-07	22.0	0.5	1.0	1	0	0	1	1	1	1	[176 - 141] (REVERSE SENSE)
m_CmonM_UR_16_16	-	a_CmonM_UR_16_16	-	3.7e-07	22.0	0.5	3.7e-07	22.0	0.5	1.0	1	0	0	1	1	1	1	[370 - 335] (REVERSE SENSE)
m_CmonM_UR_16_21	-	a_CmonM_UR_16_16	-	3.7e-07	22.0	0.5	3.7e-07	22.0	0.5	1.0	1	0	0	1	1	1	1	[176 - 141] (REVERSE SENSE)
a_CmonM_UR_16_17	-	a_CmonM_UR_16_17	-	1.4e-09	29.7	0.1	1.4e-09	29.7	0.1	1.0	1	0	0	1	1	1	1	[308 - 264] (REVERSE SENSE)
a_CmonM_UR_16_22	-	a_CmonM_UR_16_17	-	1.4e-09	29.7	0.1	1.4e-09	29.7	0.1	1.0	1	0	0	1	1	1	1	[114 - 70] (REVERSE SENSE)
m_CmonM_UR_16_17	-	a_CmonM_UR_16_17	-	1.4e-09	29.7	0.1	1.4e-09	29.7	0.1	1.0	1	0	0	1	1	1	1	[308 - 264] (REVERSE SENSE)
m_CmonM_UR_16_22	-	a_CmonM_UR_16_17	-	1.4e-09	29.7	0.1	1.4e-09	29.7	0.1	1.0	1	0	0	1	1	1	1	[114 - 70] (REVERSE SENSE)
a_CmonM_UR_16_18	-	a_CmonM_UR_16_18	-	4.9e-06	18.1	0.1	4.9e-06	18.1	0.1	1.0	1	0	0	1	1	1	1	[277 - 248] (REVERSE SENSE)
a_CmonM_UR_16_23	-	a_CmonM_UR_16_18	-	4.9e-06	18.1	0.1	4.9e-06	18.1	0.1	1.0	1	0	0	1	1	1	1	[83 - 54] (REVERSE SENSE)
m_CmonM_UR_16_18	-	a_CmonM_UR_16_18	-	1.2e-05	17.2	0.1	1.2e-05	17.2	0.1	1.0	1	0	0	1	1	1	1	[277 - 248] (REVERSE SENSE)
m_CmonM_UR_16_23	-	a_CmonM_UR_16_18	-	1.2e-05	17.2	0.1	1.2e-05	17.2	0.1	1.0	1	0	0	1	1	1	1	[83 - 54] (REVERSE SENSE)
a_CmonM_UR_16_19	-	a_CmonM_UR_16_19	-	2.8e-06	19.3	0.1	2.8e-06	19.3	0.1	1.0	1	0	0	1	1	1	1	[257 - 228] (REVERSE SENSE)
a_CmonM_UR_16_24	-	a_CmonM_UR_16_19	-	2.8e-06	19.3	0.1	2.8e-06	19.3	0.1	1.0	1	0	0	1	1	1	1	[63 - 34] (REVERSE SENSE)
m_CmonM_UR_16_19	-	a_CmonM_UR_16_19	-	6.4e-06	18.4	0.1	6.4e-06	18.4	0.1	1.0	1	0	0	1	1	1	1	[257 - 228] (REVERSE SENSE)
m_CmonM_UR_16_24	-	a_CmonM_UR_16_19	-	6.4e-06	18.4	0.1	6.4e-06	18.4	0.1	1.0	1	0	0	1	1	1	1	[63 - 34] (REVERSE SENSE)
a_CmonM_UR_16_20	-	a_CmonM_UR_16_20	-	1.7e-20	65.3	2.7	1.8e-20	65.2	2.7	1.0	1	0	0	1	1	1	1	[249 - 148] (REVERSE SENSE)
m_CmonM_UR_16_20	-	a_CmonM_UR_16_20	-	3.8e-20	64.2	2.8	4e-20	64.2	2.8	1.0	1	0	0	1	1	1	1	[249 - 148] (REVERSE SENSE)
a_CmonM_UR_16_25	-	a_CmonM_UR_16_20	-	1.5e-08	29.5	1.9	1.5e-08	29.5	1.9	1.0	1	0	0	1	1	1	1	[55 - 2] (REVERSE SENSE)
m_CmonM_UR_16_25	-	a_CmonM_UR_16_20	-	3.2e-08	28.5	2.0	3.2e-08	28.5	2.0	1.0	1	0	0	1	1	1	1	[55 - 2] (REVERSE SENSE)
a_CmonM_UR_16_16	-	a_CmonM_UR_16_21	-	3.7e-07	22.0	0.5	3.7e-07	22.0	0.5	1.0	1	0	0	1	1	1	1	[370 - 335] (REVERSE SENSE)
a_CmonM_UR_16_21	-	a_CmonM_UR_16_21	-	3.7e-07	22.0	0.5	3.7e-07	22.0	0.5	1.0	1	0	0	1	1	1	1	[176 - 141] (REVERSE SENSE)
m_CmonM_UR_16_16	-	a_CmonM_UR_16_21	-	3.7e-07	22.0	0.5	3.7e-07	22.0	0.5	1.0	1	0	0	1	1	1	1	[370 - 335] (REVERSE SENSE)
m_CmonM_UR_16_21	-	a_CmonM_UR_16_21	-	3.7e-07	22.0	0.5	3.7e-07	22.0	0.5	1.0	1	0	0	1	1	1	1	[176 - 141] (REVERSE SENSE)
a_CmonM_UR_16_17	-	a_CmonM_UR_16_22	-	1.4e-09	29.7	0.1	1.4e-09	29.7	0.1	1.0	1	0	0	1	1	1	1	[308 - 264] (REVERSE SENSE)
a_CmonM_UR_16_22	-	a_CmonM_UR_16_22	-	1.4e-09	29.7	0.1	1.4e-09	29.7	0.1	1.0	1	0	0	1	1	1	1	[114 - 70] (REVERSE SENSE)
m_CmonM_UR_16_17	-	a_CmonM_UR_16_22	-	1.4e-09	29.7	0.1	1.4e-09	29.7	0.1	1.0	1	0	0	1	1	1	1	[308 - 264] (REVERSE SENSE)
m_CmonM_UR_16_22	-	a_CmonM_UR_16_22	-	1.4e-09	29.7	0.1	1.4e-09	29.7	0.1	1.0	1	0	0	1	1	1	1	[114 - 70] (REVERSE SENSE)
a_CmonM_UR_16_18	-	a_CmonM_UR_16_23	-	4.9e-06	18.1	0.1	4.9e-06	18.1	0.1	1.0	1	0	0	1	1	1	1	[277 - 248] (REVERSE SENSE)
a_CmonM_UR_16_23	-	a_CmonM_UR_16_23	-	4.9e-06	18.1	0.1	4.9e-06	18.1	0.1	1.0	1	0	0	1	1	1	1	[83 - 54] (REVERSE SENSE)
m_CmonM_UR_16_18	-	a_CmonM_UR_16_23	-	1.2e-05	17.2	0.1	1.2e-05	17.2	0.1	1.0	1	0	0	1	1	1	1	[277 - 248] (REVERSE SENSE)
m_CmonM_UR_16_23	-	a_CmonM_UR_16_23	-	1.2e-05	17.2	0.1	1.2e-05	17.2	0.1	1.0	1	0	0	1	1	1	1	[83 - 54] (REVERSE SENSE)
a_CmonM_UR_16_19	-	a_CmonM_UR_16_24	-	2.8e-06	19.3	0.1	2.8e-06	19.3	0.1	1.0	1	0	0	1	1	1	1	[257 - 228] (REVERSE SENSE)
a_CmonM_UR_16_24	-	a_CmonM_UR_16_24	-	2.8e-06	19.3	0.1	2.8e-06	19.3	0.1	1.0	1	0	0	1	1	1	1	[63 - 34] (REVERSE SENSE)
m_CmonM_UR_16_19	-	a_CmonM_UR_16_24	-	6.4e-06	18.4	0.1	6.4e-06	18.4	0.1	1.0	1	0	0	1	1	1	1	[257 - 228] (REVERSE SENSE)
m_CmonM_UR_16_24	-	a_CmonM_UR_16_24	-	6.4e-06	18.4	0.1	6.4e-06	18.4	0.1	1.0	1	0	0	1	1	1	1	[63 - 34] (REVERSE SENSE)
a_CmonM_UR_16_25	-	a_CmonM_UR_16_25	-	4.7e-10	31.3	1.9	4.7e-10	31.3	1.9	1.0	1	0	0	1	1	1	1	[55 - 2] (REVERSE SENSE)
m_CmonM_UR_16_25	-	a_CmonM_UR_16_25	-	1.1e-09	30.3	2.0	1.1e-09	30.3	2.0	1.0	1	0	0	1	1	1	1	[55 - 2] (REVERSE SENSE)
a_CmonM_UR_16_20	-	a_CmonM_UR_16_25	-	4.6e-09	28.6	1.9	4.6e-09	28.6	1.9	1.6	2	0	0	2	2	2	1	[249 - 148] (REVERSE SENSE)
m_CmonM_UR_16_20	-	a_CmonM_UR_16_25	-	1e-08	27.6	2.0	1e-08	27.6	2.0	1.6	2	0	0	2	2	2	1	[249 - 148] (REVERSE SENSE)
a_CmonM_UR_24_1	-	a_CmonM_UR_24_1	-	6.6e-15	47.0	0.9	6.8e-15	47.0	0.9	1.0	1	0	0	1	1	1	1	[63 - 131]
m_CmonM_UR_24_1	-	a_CmonM_UR_24_1	-	1.7e-14	45.8	1.1	1.7e-14	45.8	1.1	1.0	1	0	0	1	1	1	1	[63 - 131]
a_CmonM_UR_24_2	-	a_CmonM_UR_24_2	-	2.2e-36	116.9	7.2	2.4e-36	116.8	7.2	1.0	1	0	0	1	1	1	1	[98 - 271]
m_CmonM_UR_24_2	-	a_CmonM_UR_24_2	-	5.6e-36	115.7	7.6	6e-36	115.6	7.6	1.0	1	0	0	1	1	1	1	[98 - 271]
a_CmonM_UR_24_3	-	a_CmonM_UR_24_3	-	2.9e-10	32.0	7.4	3e-10	32.0	7.4	1.0	1	0	0	1	1	1	1	[271 - 324]
m_CmonM_UR_24_3	-	a_CmonM_UR_24_3	-	2.9e-10	32.0	7.4	3e-10	32.0	7.4	1.0	1	0	0	1	1	1	1	[271 - 324]
a_CmonM_UR_24_4	-	a_CmonM_UR_24_4	-	8.7e-21	64.8	1.9	9.3e-21	64.7	1.9	1.0	1	0	0	1	1	1	1	[281 - 382]
m_CmonM_UR_24_4	-	a_CmonM_UR_24_4	-	8.7e-21	64.8	1.9	9.3e-21	64.7	1.9	1.0	1	0	0	1	1	1	1	[281 - 382]
a_CmonM_UR_24_5	-	a_CmonM_UR_24_5	-	1.7e-11	34.9	0.1	1.8e-11	34.9	0.1	1.0	1	0	0	1	1	1	1	[343 - 396]
m_CmonM_UR_24_5	-	a_CmonM_UR_24_5	-	3.8e-11	34.0	0.1	3.8e-11	34.0	0.1	1.0	1	0	0	1	1	1	1	[343 - 396]
a_CmonM_UR_24_6	-	a_CmonM_UR_24_6	-	5e-29	92.9	2.5	5.3e-29	92.8	2.5	1.0	1	0	0	1	1	1	1	[330 - 458]
m_CmonM_UR_24_6	-	a_CmonM_UR_24_6	-	5e-29	92.9	2.5	5.3e-29	92.8	2.5	1.0	1	0	0	1	1	1	1	[330 - 458]
a_CmonM_UR_24_7	-	a_CmonM_UR_24_7	-	2.3e-44	144.6	15.5	2.5e-44	144.5	15.5	1.0	1	0	0	1	1	1	1	[442 - 251] (REVERSE SENSE)
m_CmonM_UR_24_7	-	a_CmonM_UR_24_7	-	4.4e-44	143.8	15.5	4.7e-44	143.7	15.5	1.0	1	0	0	1	1	1	1	[442 - 251] (REVERSE SENSE)
a_CmonM_UR_24_8	-	a_CmonM_UR_24_8	-	1.4e-49	161.4	11.1	1.5e-49	161.3	11.1	1.0	1	0	0	1	1	1	1	[395 - 168] (REVERSE SENSE)
m_CmonM_UR_24_8	-	a_CmonM_UR_24_8	-	1.4e-49	161.4	11.1	1.5e-49	161.3	11.1	1.0	1	0	0	1	1	1	1	[395 - 168] (REVERSE SENSE)
a_CmonM_UR_24_9	-	a_CmonM_UR_24_9	-	5.2e-08	24.5	0.1	5.2e-08	24.5	0.1	1.0	1	0	0	1	1	1	1	[178 - 134] (REVERSE SENSE)
m_CmonM_UR_24_9	-	a_CmonM_UR_24_9	-	9.1e-08	23.9	0.1	9.1e-08	23.9	0.1	1.0	1	0	0	1	1	1	1	[178 - 134] (REVERSE SENSE)
a_CmonM_UR_24_10	-	a_CmonM_UR_24_10	-	1.8e-09	30.5	6.7	1.9e-09	30.5	6.7	1.0	1	0	0	1	1	1	1	[122 - 72] (REVERSE SENSE)
m_CmonM_UR_24_10	-	a_CmonM_UR_24_10	-	3.8e-09	29.6	6.6	3.8e-09	29.6	6.6	1.0	1	0	0	1	1	1	1	[122 - 72] (REVERSE SENSE)
a_CmonM_UR_24_11	-	a_CmonM_UR_24_11	-	2.8e-81	265.9	15.4	3e-81	265.8	15.4	1.0	1	0	0	1	1	1	1	[447 - 67] (REVERSE SENSE)
m_CmonM_UR_24_11	-	a_CmonM_UR_24_11	-	4.2e-81	265.3	15.1	4.6e-81	265.2	15.1	1.0	1	0	0	1	1	1	1	[447 - 67] (REVERSE SENSE)
CmonM_MORF	-	a_CmonM_UR_24_11	-	6.4e-05	18.9	3.5	6.4e-05	18.9	3.5	1.4	1	1	0	1	1	1	1	-
a_CmonM_UR_24_12	-	a_CmonM_UR_24_12	-	4.2e-19	61.2	6.7	4.4e-19	61.1	6.7	1.0	1	0	0	1	1	1	1	[124 - 32] (REVERSE SENSE)
m_CmonM_UR_24_12	-	a_CmonM_UR_24_12	-	6.1e-19	60.7	6.5	6.4e-19	60.6	6.5	1.0	1	0	0	1	1	1	1	[124 - 32] (REVERSE SENSE)
a_CmonM_UR_27_1	-	a_CmonM_UR_27_1	-	7.8e-37	118.7	6.5	8.5e-37	118.5	6.5	1.0	1	0	0	1	1	1	1	[2 - 181]
m_CmonM_UR_27_1	-	a_CmonM_UR_27_1	-	1.7e-36	117.6	6.6	1.8e-36	117.5	6.6	1.0	1	0	0	1	1	1	1	[2 - 181]
a_CmonM_UR_27_2	-	a_CmonM_UR_27_2	-	6.2e-22	69.6	2.4	6.6e-22	69.5	2.4	1.0	1	0	0	1	1	1	1	[91 - 195]
m_CmonM_UR_27_2	-	a_CmonM_UR_27_2	-	1.4e-21	68.6	2.5	1.4e-21	68.5	2.5	1.0	1	0	0	1	1	1	1	[91 - 195]
a_CmonM_UR_27_3	-	a_CmonM_UR_27_3	-	4.2e-09	28.0	1.1	4.3e-09	28.0	1.1	1.0	1	0	0	1	1	1	1	[194 - 138] (REVERSE SENSE)
m_CmonM_UR_27_3	-	a_CmonM_UR_27_3	-	8.3e-09	27.2	0.9	8.4e-09	27.2	0.9	1.0	1	0	0	1	1	1	1	[194 - 138] (REVERSE SENSE)
a_CmonM_UR_27_4	-	a_CmonM_UR_27_4	-	2e-24	78.3	3.6	2.2e-24	78.2	3.6	1.0	1	0	0	1	1	1	1	[132 - 7] (REVERSE SENSE)
m_CmonM_UR_27_4	-	a_CmonM_UR_27_4	-	2e-24	78.3	3.6	2.2e-24	78.2	3.6	1.0	1	0	0	1	1	1	1	[132 - 7] (REVERSE SENSE)
a_CmonM_UR_27_5	-	a_CmonM_UR_27_5	-	1.5e-26	86.0	7.2	1.6e-26	85.9	7.2	1.0	1	0	0	1	1	1	1	[128 - 3] (REVERSE SENSE)
m_CmonM_UR_27_5	-	a_CmonM_UR_27_5	-	2e-26	85.7	6.9	2.1e-26	85.6	6.9	1.0	1	0	0	1	1	1	1	[128 - 3] (REVERSE SENSE)
m_Nmar_UR_2_1	-	m_Nmar_UR_2_1	-	8.4e-06	17.3	0.4	8.4e-06	17.3	0.4	1.0	1	0	0	1	1	1	1	[1 - 30]
a_Nmar_UR_2_1	-	m_Nmar_UR_2_1	-	2.7e-05	16.1	0.3	2.7e-05	16.1	0.3	1.0	1	0	0	1	1	1	1	[1 - 30]
a_Nmar_UR_2_2	-	m_Nmar_UR_2_2	-	4.3e-06	18.4	0.3	4.3e-06	18.4	0.3	1.0	1	0	0	1	1	1	1	[30 - 59]
m_Nmar_UR_2_2	-	m_Nmar_UR_2_2	-	4.3e-06	18.4	0.3	4.3e-06	18.4	0.3	1.0	1	0	0	1	1	1	1	[30 - 59]
m_Nmar_UR_2_3	-	m_Nmar_UR_2_3	-	1e-12	40.2	0.5	1.1e-12	40.1	0.5	1.0	1	0	0	1	1	1	1	[5 - 61]
a_Nmar_UR_2_3	-	m_Nmar_UR_2_3	-	3.1e-12	38.8	0.5	3.2e-12	38.8	0.5	1.0	1	0	0	1	1	1	1	[5 - 61]

a_Nmar_UR_2_4	-	m_Nmar_UR_2_4	-	9e-07	19.6	0.3	9e-07	19.6	0.3	1.0	1	0	0	1	1	1	1	[54 - 19] (REVERSE SENSE)
m_Nmar_UR_2_4	-	m_Nmar_UR_2_4	-	9e-07	19.6	0.3	9e-07	19.6	0.3	1.0	1	0	0	1	1	1	1	[54 - 19] (REVERSE SENSE)
a_Nmar_UR_2_5	-	m_Nmar_UR_2_5	-	9.1e-10	31.0	1.3	9.2e-10	30.9	1.3	1.0	1	0	0	1	1	1	1	[47 - 3] (REVERSE SENSE)
m_Nmar_UR_2_5	-	m_Nmar_UR_2_5	-	9.1e-10	31.0	1.3	9.2e-10	30.9	1.3	1.0	1	0	0	1	1	1	1	[47 - 3] (REVERSE SENSE)
m_Nmar_UR_3_1	-	m_Nmar_UR_3_1	-	5.5e-06	17.8	0.7	5.5e-06	17.8	0.7	1.0	1	0	0	1	1	1	1	[6 - 35]
a_Nmar_UR_3_1	-	m_Nmar_UR_3_1	-	1.8e-05	16.6	0.6	1.8e-05	16.6	0.6	1.1	1	0	0	1	1	1	1	[6 - 35]
m_Nmar_UR_3_2	-	m_Nmar_UR_3_2	-	2.3e-06	19.1	0.1	2.3e-06	19.1	0.1	1.0	1	0	0	1	1	1	1	[57 - 92]
a_Nmar_UR_3_2	-	m_Nmar_UR_3_2	-	7.2e-06	17.9	0.1	7.2e-06	17.9	0.1	1.0	1	0	0	1	1	1	1	[57 - 92]
m_Nmar_UR_3_3	-	m_Nmar_UR_3_3	-	2.9e-05	16.0	3.0	2.9e-05	16.0	3.0	1.0	1	0	0	1	1	1	1	[91 - 120]
a_Nmar_UR_3_3	-	m_Nmar_UR_3_3	-	3.4e-05	15.9	1.9	3.4e-05	15.9	1.9	1.0	1	0	0	1	1	1	1	[91 - 120]
a_Nmar_UR_3_4	-	m_Nmar_UR_3_4	-	1.9e-26	84.9	6.6	2e-26	84.8	6.6	1.0	1	0	0	1	1	1	1	[35 - 154]
m_Nmar_UR_3_4	-	m_Nmar_UR_3_4	-	1.9e-26	84.9	6.6	2e-26	84.8	6.6	1.0	1	0	0	1	1	1	1	[35 - 154]
m_Nmar_UR_3_5	-	m_Nmar_UR_3_5	-	1e-10	34.2	5.2	1e-10	34.2	5.2	1.0	1	0	0	1	1	1	1	[167 - 220]
a_Nmar_UR_3_5	-	m_Nmar_UR_3_5	-	2.5e-10	33.1	5.0	2.5e-10	33.1	5.0	1.0	1	0	0	1	1	1	1	[167 - 220]
a_Nmar_UR_3_6	-	m_Nmar_UR_3_6	-	1.5e-26	85.2	12.3	1.6e-26	85.1	12.3	1.0	1	0	0	1	1	1	1	[120 - 254]
m_Nmar_UR_3_6	-	m_Nmar_UR_3_6	-	1.5e-26	85.2	12.3	1.6e-26	85.1	12.3	1.0	1	0	0	1	1	1	1	[120 - 254]
a_Nmar_UR_3_7	-	m_Nmar_UR_3_7	-	2.2e-43	140.5	11.0	2.4e-43	140.4	11.0	1.0	1	0	0	1	1	1	1	[154 - 357]
m_Nmar_UR_3_7	-	m_Nmar_UR_3_7	-	2.2e-43	140.5	11.0	2.4e-43	140.4	11.0	1.0	1	0	0	1	1	1	1	[154 - 357]
a_Nmar_UR_3_8	-	m_Nmar_UR_3_8	-	2.9e-09	28.0	0.4	2.9e-09	28.0	0.4	1.0	1	0	0	1	1	1	1	[357 - 407]
m_Nmar_UR_3_8	-	m_Nmar_UR_3_8	-	2.9e-09	28.0	0.4	2.9e-09	28.0	0.4	1.0	1	0	0	1	1	1	1	[357 - 407]
a_Nmar_UR_3_9	-	m_Nmar_UR_3_9	-	4e-34	109.7	3.1	4.3e-34	109.6	3.1	1.0	1	0	0	1	1	1	1	[332 - 487]
m_Nmar_UR_3_9	-	m_Nmar_UR_3_9	-	4e-34	109.7	3.1	4.3e-34	109.6	3.1	1.0	1	0	0	1	1	1	1	[332 - 487]
m_Nmar_UR_3_10	-	m_Nmar_UR_3_10	-	1.9e-12	38.7	0.5	1.9e-12	38.6	0.5	1.0	1	0	0	1	1	1	1	[484 - 537]
a_Nmar_UR_3_10	-	m_Nmar_UR_3_10	-	4.8e-12	37.5	0.3	4.8e-12	37.5	0.3	1.0	1	0	0	1	1	1	1	[484 - 537]
a_Nmar_UR_3_11	-	m_Nmar_UR_3_11	-	3.6e-07	19.9	0.2	3.6e-07	19.9	0.2	1.0	1	0	0	1	1	1	1	[516 - 554]
m_Nmar_UR_3_11	-	m_Nmar_UR_3_11	-	3.6e-07	19.9	0.2	3.6e-07	19.9	0.2	1.0	1	0	0	1	1	1	1	[516 - 554]
m_Nmar_UR_3_12	-	m_Nmar_UR_3_12	-	2.4e-19	60.9	0.1	2.5e-19	60.9	0.1	1.0	1	0	0	1	1	1	1	[524 - 607]
a_Nmar_UR_3_12	-	m_Nmar_UR_3_12	-	6.5e-19	59.6	0.1	6.8e-19	59.6	0.1	1.0	1	0	0	1	1	1	1	[524 - 607]
m_Nmar_UR_3_13	-	m_Nmar_UR_3_13	-	1.1e-10	32.7	0.4	1.1e-10	32.7	0.4	1.0	1	0	0	1	1	1	1	[576 - 623]
a_Nmar_UR_3_13	-	m_Nmar_UR_3_13	-	3.3e-10	31.5	0.4	3.3e-10	31.5	0.4	1.0	1	0	0	1	1	1	1	[576 - 623]
a_Nmar_UR_3_14	-	m_Nmar_UR_3_14	-	2.1e-18	58.2	1.4	2.2e-18	58.2	1.4	1.0	1	0	0	1	1	1	1	[565 - 654]
m_Nmar_UR_3_14	-	m_Nmar_UR_3_14	-	2.1e-18	58.2	1.4	2.2e-18	58.2	1.4	1.0	1	0	0	1	1	1	1	[565 - 654]
m_Nmar_UR_3_15	-	m_Nmar_UR_3_15	-	1.2e-14	46.7	7.2	1.3e-14	46.6	7.2	1.0	1	0	0	1	1	1	1	[639 - 713]
a_Nmar_UR_3_15	-	m_Nmar_UR_3_15	-	2.4e-14	45.8	6.9	2.5e-14	45.8	6.9	1.0	1	0	0	1	1	1	1	[639 - 713]
a_Nmar_UR_3_16	-	m_Nmar_UR_3_16	-	5.2e-12	37.3	1.3	5.3e-12	37.3	1.3	1.0	1	0	0	1	1	1	1	[710 - 766]
m_Nmar_UR_3_16	-	m_Nmar_UR_3_16	-	5.2e-12	37.3	1.3	5.3e-12	37.3	1.3	1.0	1	0	0	1	1	1	1	[710 - 766]
a_Nmar_UR_3_17	-	m_Nmar_UR_3_17	-	1.1e-08	27.1	0.1	1.1e-08	27.1	0.1	1.0	1	0	0	1	1	1	1	[776 - 814]
m_Nmar_UR_3_17	-	m_Nmar_UR_3_17	-	1.1e-08	27.1	0.1	1.1e-08	27.1	0.1	1.0	1	0	0	1	1	1	1	[776 - 814]
m_Nmar_UR_3_18	-	m_Nmar_UR_3_18	-	3.9e-17	53.8	0.3	4e-17	53.8	0.3	1.0	1	0	0	1	1	1	1	[753 - 830]
a_Nmar_UR_3_18	-	m_Nmar_UR_3_18	-	9.4e-17	52.7	0.2	9.7e-17	52.7	0.2	1.0	1	0	0	1	1	1	1	[753 - 830]
m_Nmar_UR_3_19	-	m_Nmar_UR_3_19	-	4.7e-19	59.9	0.1	4.9e-19	59.9	0.1	1.0	1	0	0	1	1	1	1	[748 - 840]
a_Nmar_UR_3_19	-	m_Nmar_UR_3_19	-	1.2e-18	58.7	0.1	1.3e-18	58.6	0.1	1.0	1	0	0	1	1	1	1	[748 - 840]
m_Nmar_UR_3_20	-	m_Nmar_UR_3_20	-	4.7e-33	106.5	4.5	5.2e-33	106.4	4.5	1.0	1	0	0	1	1	1	1	[848 - 1006]
a_Nmar_UR_3_20	-	m_Nmar_UR_3_20	-	1.2e-32	105.3	4.5	1.3e-32	105.2	4.5	1.0	1	0	0	1	1	1	1	[848 - 1006]
m_Nmar_UR_3_21	-	m_Nmar_UR_3_21	-	2.2e-35	114.1	2.5	2.3e-35	114.0	2.5	1.0	1	0	0	1	1	1	1	[898 - 1050]
a_Nmar_UR_3_21	-	m_Nmar_UR_3_21	-	5e-35	113.0	2.4	5.3e-35	112.9	2.4	1.0	1	0	0	1	1	1	1	[898 - 1050]
m_Nmar_UR_3_22	-	m_Nmar_UR_3_22	-	2.8e-06	18.5	0.4	2.8e-06	18.5	0.4	1.0	1	0	0	1	1	1	1	[1031 - 1060]
a_Nmar_UR_3_22	-	m_Nmar_UR_3_22	-	6.8e-06	17.6	0.3	6.8e-06	17.6	0.3	1.0	1	0	0	1	1	1	1	[1031 - 1060]
a_Nmar_UR_3_23	-	m_Nmar_UR_3_23	-	8.4e-52	167.9	9.4	9.2e-52	167.8	9.4	1.0	1	0	0	1	1	1	1	[840 - 1088]
m_Nmar_UR_3_23	-	m_Nmar_UR_3_23	-	8.4e-52	167.9	9.4	9.2e-52	167.8	9.4	1.0	1	0	0	1	1	1	1	[840 - 1088]
a_Nmar_UR_3_24	-	m_Nmar_UR_3_24	-	3.5e-06	18.3	0.3	3.5e-06	18.3	0.3	1.0	1	0	0	1	1	1	1	[1146 - 1181]
m_Nmar_UR_3_24	-	m_Nmar_UR_3_24	-	3.5e-06	18.3	0.3	3.5e-06	18.3	0.3	1.0	1	0	0	1	1	1	1	[1146 - 1181]
a_Nmar_UR_3_25	-	m_Nmar_UR_3_25	-	3.3e-33	106.6	6.2	3.6e-33	106.5	6.2	1.0	1	0	0	1	1	1	1	[1060 - 1206]
m_Nmar_UR_3_25	-	m_Nmar_UR_3_25	-	3.3e-33	106.6	6.2	3.6e-33	106.5	6.2	1.0	1	0	0	1	1	1	1	[1060 - 1206]
a_Nmar_UR_3_26	-	m_Nmar_UR_3_26	-	1.1e-11	36.7	3.0	1.2e-11	36.7	3.0	1.0	1	0	0	1	1	1	1	[1328 - 1381]
m_Nmar_UR_3_26	-	m_Nmar_UR_3_26	-	1.1e-11	36.7	3.0	1.2e-11	36.7	3.0	1.0	1	0	0	1	1	1	1	[1328 - 1381]
m_Nmar_UR_3_27	-	m_Nmar_UR_3_27	-	2.4e-39	126.6	6.4	2.6e-39	126.5	6.4	1.0	1	0	0	1	1	1	1	[1203 - 1391]
a_Nmar_UR_3_27	-	m_Nmar_UR_3_27	-	4.9e-39	125.7	6.1	5.3e-39	125.6	6.1	1.0	1	0	0	1	1	1	1	[1203 - 1391]
a_Nmar_UR_3_28	-	m_Nmar_UR_3_28	-	1.2e-07	22.9	0.6	1.2e-07	22.9	0.6	1.0	1	0	0	1	1	1	1	[1378 - 1416]
m_Nmar_UR_3_28	-	m_Nmar_UR_3_28	-	1.2e-07	22.9	0.6	1.2e-07	22.9	0.6	1.0	1	0	0	1	1	1	1	[1378 - 1416]
m_Nmar_UR_3_29	-	m_Nmar_UR_3_29	-	2.7e-09	29.6	1.0	2.7e-09	29.6	1.0	1.0	1	0	0	1	1	1	1	[1371 - 1327] (REVERSE SENSE)
a_Nmar_UR_3_29	-	m_Nmar_UR_3_29	-	7e-09	28.5	1.2	7e-09	28.5	1.2	1.0	1	0	0	1	1	1	1	[1371 - 1327] (REVERSE SENSE)
a_Nmar_UR_3_30	-	m_Nmar_UR_3_30	-	4.5e-22	71.0	7.3	4.7e-22	70.9	7.3	1.0	1	0	0	1	1	1	1	[1405 - 1298] (REVERSE SENSE)
m_Nmar_UR_3_30	-	m_Nmar_UR_3_30	-	4.5e-22	71.0	7.3	4.7e-22	70.9	7.3	1.0	1	0	0	1	1	1	1	[1405 - 1298] (REVERSE SENSE)
m_Nmar_UR_3_31	-	m_Nmar_UR_3_31	-	2.6e-06	18.7	1.4	2.6e-06	18.7	1.4	1.0	1	0	0	1	1	1	1	[1323 - 1291] (REVERSE SENSE)
a_Nmar_UR_3_31	-	m_Nmar_UR_3_31	-	6.9e-06	17.7	1.4	6.9e-06	17.7	1.4	1.0	1	0	0	1	1	1	1	[1323 - 1291] (REVERSE SENSE)
m_Nmar_UR_3_32	-	m_Nmar_UR_3_32	-	3.5e-09	28.8	1.9	3.5e-09	28.8	1.9	1.0	1	0	0	1	1	1	1	[1199 - 1158] (REVERSE SENSE)
a_Nmar_UR_3_32	-	m_Nmar_UR_3_32	-	1.1e-08	27.5	2.0	1.1e-08	27.5	2.0	1.0	1	0	0	1	1	1	1	[1199 - 1158] (REVERSE SENSE)
a_Nmar_UR_3_33	-	m_Nmar_UR_3_33	-	8.5e-31	98.6	4.5	9.3e-31	98.5	4.5	1.0	1	0	0	1	1	1	1	[1288 - 1133] (REVERSE SENSE)
m_Nmar_UR_3_33	-	m_Nmar_UR_3_33	-	8.5e-31	98.6	4.5	9.3e-31	98.5	4.5	1.0	1	0	0	1	1	1	1	[1288 - 1133] (REVERSE SENSE)
a_Nmar_UR_3_34	-	m_Nmar_UR_3_34	-	1.1e-06	20.2	0.4	1.1e-06	20.2	0.4	1.0	1	0	0	1	1	1	1	[1127 - 1089] (REVERSE SENSE)
m_Nmar_UR_3_34	-	m_Nmar_UR_3_34	-	1.1e-06	20.2	0.4	1.1e-06	20.2	0.4	1.0	1	0	0	1	1	1	1	[1127 - 1089] (REVERSE SENSE)
m_Nmar_UR_3_35	-	m_Nmar_UR_3_35	-	2.4e-36	117.4	5.3	2.6e-36	117.3	5.3	1.0	1	0	0	1	1	1	1	[1167 - 1009] (REVERSE SENSE)
a_Nmar_UR_3_35	-	m_Nmar_UR_3_35	-	3.8e-36	116.8	5.0	4.1e-36	116.7	5.0	1.0	1	0	0	1	1	1	1	[1167 - 1009] (REVERSE SENSE)
a_Nmar_UR_3_36	-	m_Nmar_UR_3_36	-	1e-34	111.9	2.0	1.1e-34	111.8	2.0	1.0	1	0	0	1	1	1	1	[1079 - 930] (REVERSE SENSE)
m_Nmar_UR_3_36	-	m_Nmar_UR_3_36	-	1e-34	111.9	2.0	1.1e-34	111.8	2.0	1.0	1	0	0	1	1	1	1	[1079 - 930] (REVERSE SENSE)
a_Nmar_UR_3_37	-	m_Nmar_UR_3_37	-	1.2e-34	111.7	3.4	1.3e-34	111.6	3.4	1.0	1	0	0	1	1	1	1	[1072 - 902] (REVERSE SENSE)
m_Nmar_UR_3_37	-	m_Nmar_UR_3_37	-	1.2e-34	111.7	3.4	1.3e-34	111.6	3.4	1.0	1	0	0	1	1	1	1	[1072 - 902] (REVERSE SENSE)
a_Nmar_UR_3_38	-	m_Nmar_UR_3_38	-	4.8e-08	24.1	0.6	4.8e-08	24.1	0.6	1.0	1	0	0	1	1	1	1	[899 - 855] (REVERSE SENSE)
m_Nmar_UR_3_38	-	m_Nmar_UR_3_38	-	4.8e-08	24.1	0.6	4.8e-08	24.1	0.6	1.0	1	0	0	1	1	1	1	[899 - 855] (REVERSE SENSE)
a_Nmar_UR_3_39	-	m_Nmar_UR_3_39	-	6.7e-35	112.5	1.2	7.3e-35	112.4	1.2	1.0	1	0	0	1	1	1	1	[1002 - 841] (REVERSE SENSE)
m_Nmar_UR_3_39	-	m_Nmar_UR_3_39	-	6.7e-35	112.5	1.2	7.3e-35	112.4	1.2	1.0	1	0	0	1	1	1	1	[1002 - 841] (REVERSE SENSE)
m_Nmar_UR_3_40	-	m_Nmar_UR_3_40	-	6.7e-15	47.2	0.5	6.9e-15	47.2	0.5	1.0	1	0	0	1	1	1	1	[829 - 767] (REVERSE SENSE)
a_Nmar_UR_3_40	-	m_Nmar_UR_3_40	-	1.5e-14	46.2	0.6	1.5e-14	46.2	0.6	1.0	1	0	0	1	1	1	1	[829 - 767] (REVERSE SENSE)
m_Nmar_UR_3_41	-	m_Nmar_UR_3_41	-	2.8e-31	100.6	3.2	3e-31	100.5	3.2	1.0	1	0	0	1	1	1	1	[848 - 702] (REVERSE SENSE)
a_Nmar_UR_3_41	-	m_Nmar_UR_3_41	-	5.9e-31	99.6	3.2	6.3e-31	99.5	3.2	1.0	1	0	0	1	1	1	1	[848 - 702] (REVERSE SENSE)
m_Nmar_UR_3_42	-	m_Nmar_UR_3_42	-	5e-38	122.6	1.8	5.4e-38	122.5	1.8	1.0	1	0	0	1	1	1	1	[807 - 640] (REVERSE SENSE)
a_Nmar_UR_3_42	-	m_Nmar_UR_3_42	-	1.5e-37	121.1	2.0	1.6e-37	121.0	2.0	1.0	1	0	0	1	1	1	1	[807 - 640] (REVERSE SENSE)
m_Nmar_UR_3_43	-	m_Nmar_UR_3_43	-	3.8e-30	97.4	11.8	4.1e-30	97.3	11.8	1.0	1	0	0	1	1	1	1	[760 - 626] (REVERSE SENSE)
a_Nmar_UR_3_43	-	m_Nmar_UR_3_43	-	6.8e-30	96.7	11.6	7.3e-30	96.6	11.6	1.0	1	0	0	1	1	1	1	[760 - 626] (REVERSE SENSE)
a_Nmar_UR_3_44	-	m_Nmar_UR_3_44	-	1e-09	29.8	0.1	1e-09	29.8	0.1	1.0	1	0	0	1	1	1	1	[622 - 575] (REVERSE SENSE)
m_Nmar_UR_3_44	-	m_Nmar_UR_3_44	-	1e-09	29.8	0.1	1e-09	29.8	0.1	1.0	1	0	0	1	1	1	1	[622 - 575] (REVERSE SENSE)
m_Nmar_UR_3_45	-	m_Nmar_UR_3_45	-	4.6e-12	38.4	1.6	4.7e-12	38.3	1.6	1.0	1	0	0	1	1	1	1	[582 - 529] (REVERSE SENSE)
a_Nmar_UR_3_45	-	m_Nmar_UR_3_45	-	1.4e-11	37.0	1.6	1.4e-11	37.0	1.6	1.0	1	0	0	1	1	1	1	[582 - 529] (REVERSE SENSE)
a_Nmar_UR_3_46	-	m_Nmar_UR_3_46	-	2.4e-10	31.2	0.1	2.4e-10	31.2	0.1	1.0	1	0	0	1	1	1	1	[547 - 494] (REVERSE SENSE)
m_Nmar_UR_3_46	-	m_Nmar_UR_3_46	-	2.4e-10	31.2	0.1	2.4e-10	31.2	0.1	1.0	1	0	0	1	1	1	1	[547 - 494] (REVERSE SENSE)
m_Nmar_UR_3_47	-	m_Nmar_UR_3_47	-	9.3e-08	23.8	0.3	9.3e-08	23.8	0.3	1.0	1	0	0	1	1	1	1	[525 - 490] (REVERSE SENSE)
a_Nmar_UR_3_47	-	m_Nmar_UR_3_47	-	2.7e-07	22.6	0.1	2.7e-07	22.6	0.1	1.0	1	0	0	1	1	1	1	[525 - 490] (REVERSE SENSE)
m_Nmar_UR_3_48	-	m_Nmar_UR_3_48	-	2.5e-60	196.0	3.3	2.8e-60	195.9	3.3	1.0	1	0	0	1	1	1	1	[653 - 381] (REVERSE SENSE)
a_Nmar_UR_3_48	-	m_Nmar_UR_3_48	-	5.8e-60	194.9	3.2	6.4e-60	194.8	3.2	1.0	1	0	0	1	1	1	1	[653 - 381] (REVERSE SENSE)
m_Nmar_UR_3_49	-	m_Nmar_UR_3_49	-	5.7e-21	66.8	5.5	6.1e-21	66.7	5.5	1.0	1	0	0	1	1	1	1	[406 - 305] (REVERSE SENSE)
a_Nmar_UR_3_49	-	m_Nmar_UR_3_49	-	1e-20	66.0	5.1	1.1e-20	65.9	5.1	1.0	1	0	0	1	1	1	1	[406 - 305] (REVERSE SENSE)
a_Nmar_UR_3_50	-	m_Nmar_UR_3_50	-	2.2e-13	41.7	0.3	2.3e-13	41.6	0.3	1.0	1	0	0	1	1	1	1	[314 - 249] (REVERSE SENSE)
m_Nmar_UR_3_50	-	m_Nmar_UR_3_50	-	2.2e-13	41.7	0.3	2.3e-13	41.6	0.3	1.0	1	0	0	1	1	1	1	[314 - 249] (REVERSE SENSE)
m_Nmar_UR_3_51	-	m_Nmar_UR_3_51	-	1.2e-12	40.4	1.7	1.3e-12	40.3	1.7	1.0	1	0	0	1	1	1	1	[286 - 233] (REVERSE SENSE)
a_Nmar_UR_3_51	-	m_Nmar_UR_3_51	-	2.9e-12	39.3	1.7	2.9e-12	39.3	1.7	1.0	1	0	0	1	1	1	1	[286 - 233] (REVERSE SENSE)
m_Nmar_UR_3_52	-	m_Nmar_UR_3_52	-	3e-12	38.3	0.5	3.1e-12	38.3	0.5	1.0	1	0	0	1	1	1	1	[255 - 202] (REVERSE SENSE)
a_Nmar_UR_3_52	-	m_Nmar_UR_3_52	-	9.4e-12	36.9	0.6	9.4e-12	36.9	0.6	1.0	1	0	0	1	1	1	1	[255 - 202] (REVERSE SENSE)
m_Nmar_UR_3_53	-	m_Nmar_UR_3_53	-	3.1e-21	68.0	4.6	3.3e-21	68.0	4.6	1.0	1	0	0	1	1	1	1	[245 - 141] (REVERSE SENSE)

a_Nmar_UR_3_53	-	m_Nmar_UR_3_53	-	5.4e-21	67.3	4.3	5.7e-21	67.2	4.3	1.0	1	0	0	1	1	1	1	[245 - 141] (REVERSE SENSE)
m_Nmar_UR_3_54	-	m_Nmar_UR_3_54	-	2.1e-28	90.6	2.9	2.3e-28	90.5	2.9	1.0	1	0	0	1	1	1	1	[229 - 95] (REVERSE SENSE)
a_Nmar_UR_3_54	-	m_Nmar_UR_3_54	-	4e-28	89.8	2.8	4.3e-28	89.7	2.8	1.0	1	0	0	1	1	1	1	[229 - 95] (REVERSE SENSE)
a_Nmar_UR_3_55	-	m_Nmar_UR_3_55	-	2.8e-08	27.1	6.0	2.8e-08	27.1	6.0	1.0	1	0	0	1	1	1	1	[119 - 78] (REVERSE SENSE)
m_Nmar_UR_3_55	-	m_Nmar_UR_3_55	-	2.8e-08	27.1	6.0	2.8e-08	27.1	6.0	1.0	1	0	0	1	1	1	1	[119 - 78] (REVERSE SENSE)
a_Nmar_UR_3_56	-	m_Nmar_UR_3_56	-	6.3e-24	77.7	15.9	6.8e-24	77.6	15.9	1.0	1	0	0	1	1	1	1	[183 - 70] (REVERSE SENSE)
m_Nmar_UR_3_56	-	m_Nmar_UR_3_56	-	6.3e-24	77.7	15.9	6.8e-24	77.6	15.9	1.0	1	0	0	1	1	1	1	[183 - 70] (REVERSE SENSE)
m_Nmar_UR_3_57	-	m_Nmar_UR_3_57	-	2.1e-05	15.8	0.1	2.1e-05	15.8	0.1	1.0	1	0	0	1	1	1	1	[39 - 10] (REVERSE SENSE)
a_Nmar_UR_3_57	-	m_Nmar_UR_3_57	-	7.6e-05	14.5	0.0	7.6e-05	14.5	0.0	1.0	1	0	0	1	1	1	1	[39 - 10] (REVERSE SENSE)
a_Nmar_UR_3_58	-	m_Nmar_UR_3_58	-	7.2e-15	47.0	2.0	7.4e-15	46.9	2.0	1.0	1	0	0	1	1	1	1	[70 - 2] (REVERSE SENSE)
m_Nmar_UR_3_58	-	m_Nmar_UR_3_58	-	7.2e-15	47.0	2.0	7.4e-15	46.9	2.0	1.0	1	0	0	1	1	1	1	[70 - 2] (REVERSE SENSE)
a_Nmar_UR_14_1	-	m_Nmar_UR_14_1	-	4.5e-08	24.7	2.8	4.5e-08	24.7	2.8	1.0	1	0	0	1	1	1	1	[9 - 47]
m_Nmar_UR_14_1	-	m_Nmar_UR_14_1	-	4.5e-08	24.7	2.8	4.5e-08	24.7	2.8	1.0	1	0	0	1	1	1	1	[9 - 47]
a_Nmar_UR_14_2	-	m_Nmar_UR_14_2	-	2.8e-14	44.4	4.9	2.9e-14	44.4	4.9	1.0	1	0	0	1	1	1	1	[5 - 79]
m_Nmar_UR_14_2	-	m_Nmar_UR_14_2	-	2.8e-14	44.4	4.9	2.9e-14	44.4	4.9	1.0	1	0	0	1	1	1	1	[5 - 79]
m_Nmar_UR_14_3	-	m_Nmar_UR_14_3	-	3.3e-09	28.6	3.7	3.3e-09	28.6	3.7	1.0	1	0	0	1	1	1	1	[78 - 25] (REVERSE SENSE)
a_Nmar_UR_14_3	-	m_Nmar_UR_14_3	-	7.2e-09	27.7	3.4	7.3e-09	27.6	3.4	1.0	1	0	0	1	1	1	1	[78 - 25] (REVERSE SENSE)
a_Nmar_UR_14_4	-	m_Nmar_UR_14_4	-	1.1e-06	20.5	1.0	1.1e-06	20.5	1.0	1.0	1	0	0	1	1	1	1	[35 - 3] (REVERSE SENSE)
m_Nmar_UR_14_4	-	m_Nmar_UR_14_4	-	1.1e-06	20.5	1.0	1.1e-06	20.5	1.0	1.0	1	0	0	1	1	1	1	[35 - 3] (REVERSE SENSE)
m_Nmar_UR_14_5	-	m_Nmar_UR_14_5	-	2.5e-08	26.0	2.5	2.5e-08	26.0	2.5	1.0	1	0	0	1	1	1	1	[43 - 2] (REVERSE SENSE)
a_Nmar_UR_14_5	-	m_Nmar_UR_14_5	-	5.8e-08	25.0	2.2	5.8e-08	25.0	2.2	1.0	1	0	0	1	1	1	1	[43 - 2] (REVERSE SENSE)
m_Nmar_UR_20_1	-	m_Nmar_UR_20_1	-	2e-08	24.3	0.6	2e-08	24.3	0.6	1.0	1	0	0	1	1	1	1	[14 - 58]
a_Nmar_UR_20_1	-	m_Nmar_UR_20_1	-	5.9e-08	23.2	0.5	5.9e-08	23.2	0.5	1.0	1	0	0	1	1	1	1	[14 - 58]
m_Nmar_UR_20_2	-	m_Nmar_UR_20_2	-	5e-11	35.1	3.5	5.2e-11	35.1	3.5	1.0	1	0	0	1	1	1	1	[57 - 1] (REVERSE SENSE)
a_Nmar_UR_20_2	-	m_Nmar_UR_20_2	-	9.2e-11	34.4	3.3	9.4e-11	34.4	3.3	1.0	1	0	0	1	1	1	1	[57 - 1] (REVERSE SENSE)
m_Nmar_UR_21_1	-	m_Nmar_UR_21_1	-	1.5e-05	15.3	0.1	1.5e-05	15.3	0.1	1.0	1	0	0	1	1	1	1	[10 - 39]
a_Nmar_UR_21_1	-	m_Nmar_UR_21_1	-	5.5e-05	14.0	0.1	5.5e-05	14.0	0.1	1.1	1	0	0	1	1	1	1	[10 - 39]
a_Nmar_UR_22_1	-	m_Nmar_UR_22_1	-	6.4e-05	14.4	2.2	6.4e-05	14.4	2.2	1.1	1	0	0	1	1	1	1	[6 - 35]
m_Nmar_UR_22_1	-	m_Nmar_UR_22_1	-	6.4e-05	14.4	2.2	6.4e-05	14.4	2.2	1.1	1	0	0	1	1	1	1	[6 - 35]
m_Nmar_UR_22_2	-	m_Nmar_UR_22_2	-	3.3e-07	21.6	1.2	3.3e-07	21.6	1.2	1.0	1	0	0	1	1	1	1	[8 - 40]
a_Nmar_UR_22_2	-	m_Nmar_UR_22_2	-	7.4e-07	20.7	0.7	7.4e-07	20.7	0.7	1.0	1	0	0	1	1	1	1	[8 - 40]
a_Nmar_UR_23_1	-	m_Nmar_UR_23_1	-	2.4e-08	25.5	1.7	2.4e-08	25.5	1.7	1.0	1	0	0	1	1	1	1	[11 - 52]
m_Nmar_UR_23_1	-	m_Nmar_UR_23_1	-	2.4e-08	25.5	1.7	2.4e-08	25.5	1.7	1.0	1	0	0	1	1	1	1	[11 - 52]
a_Nmar_UR_23_2	-	m_Nmar_UR_23_2	-	1.6e-12	38.3	0.8	1.7e-12	38.3	0.8	1.0	1	0	0	1	1	1	1	[39 - 95]
m_Nmar_UR_23_2	-	m_Nmar_UR_23_2	-	1.6e-12	38.3	0.8	1.7e-12	38.3	0.8	1.0	1	0	0	1	1	1	1	[39 - 95]
m_Nmar_UR_23_3	-	m_Nmar_UR_23_3	-	1.3e-09	30.1	0.6	1.3e-09	30.1	0.6	1.0	1	0	0	1	1	1	1	[95 - 45] (REVERSE SENSE)
a_Nmar_UR_23_3	-	m_Nmar_UR_23_3	-	3.5e-09	28.9	0.6	3.6e-09	28.9	0.6	1.0	1	0	0	1	1	1	1	[95 - 45] (REVERSE SENSE)
a_Nmar_UR_23_4	-	m_Nmar_UR_23_4	-	8.6e-09	27.0	2.0	8.6e-09	27.0	2.0	1.0	1	0	0	1	1	1	1	[42 - 1] (REVERSE SENSE)
m_Nmar_UR_23_4	-	m_Nmar_UR_23_4	-	8.6e-09	27.0	2.0	8.6e-09	27.0	2.0	1.0	1	0	0	1	1	1	1	[42 - 1] (REVERSE SENSE)
m_Nmar_UR_24_1	-	m_Nmar_UR_24_1	-	2.2e-06	18.7	0.2	2.2e-06	18.7	0.2	1.0	1	0	0	1	1	1	1	[24 - 56]
a_Nmar_UR_24_1	-	m_Nmar_UR_24_1	-	7.6e-06	17.4	0.2	7.6e-06	17.4	0.2	1.0	1	0	0	1	1	1	1	[24 - 56]
a_Nmar_UR_24_2	-	m_Nmar_UR_24_2	-	4.5e-07	20.5	0.3	4.5e-07	20.5	0.3	1.0	1	0	0	1	1	1	1	[28 - 63]
m_Nmar_UR_24_2	-	m_Nmar_UR_24_2	-	4.5e-07	20.5	0.3	4.5e-07	20.5	0.3	1.0	1	0	0	1	1	1	1	[28 - 63]
m_Atra_UR_2_1	-	m_Atra_UR_2_1	-	9e-07	20.8	1.2	9e-07	20.8	1.2	1.0	1	0	0	1	1	1	1	[1 - 33]
a_Atra_UR_2_1	-	m_Atra_UR_2_1	-	1.6e-06	20.2	0.5	1.6e-06	20.2	0.5	1.0	1	0	0	1	1	1	1	[1 - 33]
a_Atra_UR_2_2	-	m_Atra_UR_2_2	-	2.3e-06	20.4	0.8	2.3e-06	20.4	0.8	1.0	1	0	0	1	1	1	1	[32 - 3] (REVERSE SENSE)
m_Atra_UR_2_2	-	m_Atra_UR_2_2	-	2.3e-06	20.4	0.8	2.3e-06	20.4	0.8	1.0	1	0	0	1	1	1	1	[32 - 3] (REVERSE SENSE)
m_Atra_UR_3_1	-	m_Atra_UR_3_1	-	8.7e-09	26.9	0.6	8.8e-09	26.9	0.6	1.0	1	0	0	1	1	1	1	[68 - 24] (REVERSE SENSE)
a_Atra_UR_3_1	-	m_Atra_UR_3_1	-	2e-08	25.9	0.5	2e-08	25.9	0.5	1.0	1	0	0	1	1	1	1	[68 - 24] (REVERSE SENSE)
m_Atra_UR_13_1	-	m_Atra_UR_13_1	-	3.3e-06	17.7	0.5	3.3e-06	17.7	0.5	1.0	1	0	0	1	1	1	1	[2 - 31]
a_Atra_UR_13_1	-	m_Atra_UR_13_1	-	9.4e-06	16.6	0.3	9.4e-06	16.6	0.3	1.0	1	0	0	1	1	1	1	[2 - 31]
a_Atra_UR_13_2	-	m_Atra_UR_13_2	-	6.3e-09	28.4	3.6	6.3e-09	28.4	3.6	1.0	1	0	0	1	1	1	1	[37 - 78]
m_Atra_UR_13_2	-	m_Atra_UR_13_2	-	6.3e-09	28.4	3.6	6.3e-09	28.4	3.6	1.0	1	0	0	1	1	1	1	[37 - 78]
m_Atra_UR_13_3	-	m_Atra_UR_13_3	-	3.7e-16	51.4	5.4	3.9e-16	51.3	5.4	1.0	1	0	0	1	1	1	1	[27 - 104]
a_Atra_UR_13_3	-	m_Atra_UR_13_3	-	5.9e-16	50.8	4.7	6.1e-16	50.7	4.7	1.0	1	0	0	1	1	1	1	[27 - 104]
a_Atra_UR_13_4	-	m_Atra_UR_13_4	-	1e-07	24.6	3.6	1e-07	24.6	3.6	1.0	1	0	0	1	1	1	1	[115 - 150]
m_Atra_UR_13_4	-	m_Atra_UR_13_4	-	1e-07	24.6	3.6	1e-07	24.6	3.6	1.0	1	0	0	1	1	1	1	[115 - 150]
m_Atra_UR_13_5	-	m_Atra_UR_13_5	-	4.1e-24	77.2	5.3	4.4e-24	77.2	5.3	1.0	1	0	0	1	1	1	1	[41 - 154]
a_Atra_UR_13_5	-	m_Atra_UR_13_5	-	1.1e-23	76.0	5.3	1.1e-23	75.9	5.3	1.0	1	0	0	1	1	1	1	[41 - 154]
m_Atra_UR_13_6	-	m_Atra_UR_13_6	-	3.6e-09	27.9	2.4	3.6e-09	27.9	2.4	1.0	1	0	0	1	1	1	1	[153 - 200]
a_Atra_UR_13_6	-	m_Atra_UR_13_6	-	7.5e-09	27.1	2.0	7.5e-09	27.1	2.0	1.0	1	0	0	1	1	1	1	[153 - 200]
m_Atra_UR_13_7	-	m_Atra_UR_13_7	-	5.9e-11	34.4	3.3	5.9e-11	34.4	3.3	1.0	1	0	0	1	1	1	1	[242 - 189] (REVERSE SENSE)
a_Atra_UR_13_7	-	m_Atra_UR_13_7	-	1.2e-10	33.5	2.9	1.2e-10	33.5	2.9	1.0	1	0	0	1	1	1	1	[242 - 189] (REVERSE SENSE)
m_Atra_UR_13_8	-	m_Atra_UR_13_8	-	7.9e-06	17.8	1.7	7.9e-06	17.8	1.7	1.0	1	0	0	1	1	1	1	[182 - 153] (REVERSE SENSE)
a_Atra_UR_13_8	-	m_Atra_UR_13_8	-	2e-05	16.9	1.3	2e-05	16.9	1.3	1.0	1	0	0	1	1	1	1	[182 - 153] (REVERSE SENSE)
a_Atra_UR_13_9	-	m_Atra_UR_13_9	-	6.6e-14	42.1	1.6	6.9e-14	42.0	1.6	1.0	1	0	0	1	1	1	1	[147 - 73] (REVERSE SENSE)
m_Atra_UR_13_9	-	m_Atra_UR_13_9	-	6.6e-14	42.1	1.6	6.9e-14	42.0	1.6	1.0	1	0	0	1	1	1	1	[147 - 73] (REVERSE SENSE)
m_Atra_UR_13_10	-	m_Atra_UR_13_10	-	2.5e-33	107.1	5.8	2.7e-33	107.0	5.8	1.0	1	0	0	1	1	1	1	[223 - 62] (REVERSE SENSE)
a_Atra_UR_13_10	-	m_Atra_UR_13_10	-	6.5e-33	105.8	5.9	7e-33	105.7	5.9	1.0	1	0	0	1	1	1	1	[223 - 62] (REVERSE SENSE)
a_Atra_UR_13_11	-	m_Atra_UR_13_11	-	1e-07	22.5	0.4	1e-07	22.5	0.4	1.0	1	0	0	1	1	1	1	[69 - 34] (REVERSE SENSE)
m_Atra_UR_13_11	-	m_Atra_UR_13_11	-	1e-07	22.5	0.4	1e-07	22.5	0.4	1.0	1	0	0	1	1	1	1	[69 - 34] (REVERSE SENSE)
a_Atra_UR_13_12	-	m_Atra_UR_13_12	-	3.6e-08	25.7	2.6	3.6e-08	25.7	2.6	1.0	1	0	0	1	1	1	1	[50 - 15] (REVERSE SENSE)
m_Atra_UR_13_12	-	m_Atra_UR_13_12	-	3.6e-08	25.7	2.6	3.6e-08	25.7	2.6	1.0	1	0	0	1	1	1	1	[50 - 15] (REVERSE SENSE)
m_Atra_UR_14_1	-	m_Atra_UR_14_1	-	1e-11	35.8	0.5	1e-11	35.8	0.5	1.0	1	0	0	1	1	1	1	[11 - 64]
a_Atra_UR_14_1	-	m_Atra_UR_14_1	-	2.8e-11	34.6	0.4	2.8e-11	34.6	0.4	1.0	1	0	0	1	1	1	1	[11 - 64]
m_Atra_UR_14_2	-	m_Atra_UR_14_2	-	1.7e-08	25.5	1.2	1.8e-08	25.5	1.2	1.0	1	0	0	1	1	1	1	[37 - 84]
a_Atra_UR_14_2	-	m_Atra_UR_14_2	-	4.4e-08	24.4	1.0	4.4e-08	24.4	1.0	1.0	1	0	0	1	1	1	1	[37 - 84]
m_Atra_UR_14_3	-	m_Atra_UR_14_3	-	5.3e-06	19.1	2.9	5.3e-06	19.1	2.9	1.0	1	0	0	1	1	1	1	[96 - 67] (REVERSE SENSE)
a_Atra_UR_14_3	-	m_Atra_UR_14_3	-	1.5e-05	17.9	2.7	1.5e-05	17.9	2.7	1.0	1	0	0	1	1	1	1	[96 - 67] (REVERSE SENSE)
m_Atra_UR_14_4	-	m_Atra_UR_14_4	-	2.5e-08	26.9	4.2	2.5e-08	26.9	4.2	1.0	1	0	0	1	1	1	1	[94 - 50] (REVERSE SENSE)
a_Atra_UR_14_4	-	m_Atra_UR_14_4	-	6.3e-08	25.8	4.0	6.3e-08	25.8	4.0	1.0	1	0	0	1	1	1	1	[94 - 50] (REVERSE SENSE)
m_Atra_UR_14_5	-	m_Atra_UR_14_5	-	1.6e-05	15.1	0.4	1.6e-05	15.1	0.4	1.0	1	0	0	1	1	1	1	[53 - 24] (REVERSE SENSE)
a_Atra_UR_14_5	-	m_Atra_UR_14_5	-	5.2e-05	14.0	0.2	5.2e-05	14.0	0.2	1.1	1	0	0	1	1	1	1	[53 - 24] (REVERSE SENSE)
m_Atra_UR_21_1	-	m_Atra_UR_21_1	-	6.1e-10	31.5	1.3	6.2e-10	31.5	1.3	1.0	1	0	0	1	1	1	1	[16 - 69]
a_Atra_UR_21_1	-	m_Atra_UR_21_1	-	1.5e-09	30.5	1.0	1.5e-09	30.5	1.0	1.0	1	0	0	1	1	1	1	[16 - 69]
m_Atra_UR_21_2	-	m_Atra_UR_21_2	-	2.5e-11	35.4	0.9	2.6e-11	35.3	0.9	1.0	1	0	0	1	1	1	1	[41 - 94]
a_Atra_UR_21_2	-	m_Atra_UR_21_2	-	5.2e-11	34.5	0.7	5.2e-11	34.5	0.7	1.0	1	0	0	1	1	1	1	[41 - 94]
m_Atra_UR_21_3	-	m_Atra_UR_21_3	-	6.7e-07	21.6	1.7	6.7e-07	21.6	1.7	1.0	1	0	0	1	1	1	1	[66 - 98]
a_Atra_UR_21_3	-	m_Atra_UR_21_3	-	1.3e-06	20.9	1.1	1.3e-06	20.9	1.1	1.0	1	0	0	1	1	1	1	[66 - 98]
m_Atra_UR_21_4	-	m_Atra_UR_21_4	-	4.9e-07	20.3	0.4	4.9e-07	20.3	0.4	1.0	1	0	0	1	1	1	1	[102 - 137]
a_Atra_UR_21_4	-	m_Atra_UR_21_4	-	1.4e-06	19.2	0.2	1.4e-06	19.2	0.2	1.0	1	0	0	1	1	1	1	[102 - 137]
a_Atra_UR_21_5	-	m_Atra_UR_21_5	-	3.1e-11	34.7	2.2	3.1e-11	34.7	2.2	1.0	1	0	0	1	1	1	1	[137 - 187]
m_Atra_UR_21_5	-	m_Atra_UR_21_5	-	3.1e-11	34.7	2.2	3.1e-11	34.7	2.2	1.0	1	0	0	1	1	1	1	[137 - 187]
m_Atra_UR_21_6	-	m_Atra_UR_21_6	-	6.2e-28	89.2	5.8	6.7e-28	89.1	5.8	1.0	1	0	0	1	1	1	1	[73 - 213]
a_Atra_UR_21_6	-	m_Atra_UR_21_6	-	1.2e-27	88.4	5.4	1.2e-27	88.3	5.4	1.0	1	0	0	1	1	1	1	[73 - 213]
m_Atra_UR_21_7	-	m_Atra_UR_21_7	-	3.8e-13	41.9	4.4	3.9e-13	41.9	4.4	1.0	1	0	0	1	1	1	1	[208 - 146] (REVERSE SENSE)
a_Atra_UR_21_7	-	m_Atra_UR_21_7	-	8.4e-13	40.9	4.4	8.6e-13	40.9	4.4	1.0	1	0	0	1	1	1	1	[208 - 146] (REVERSE SENSE)
m_Atra_UR_21_8	-	m_Atra_UR_21_8	-	1.7e-06	21.7	7.4	1.7e-06	21.7	7.4	1.0	1	0	0	1	1	1	1	[177 - 136] (REVERSE SENSE)
a_Atra_UR_21_8	-	m_Atra_UR_21_8	-	3.3e-06	20.9	7.1	3.3e-06	20.9	7.1	1.0	1	0	0	1	1	1	1	[177 - 136] (REVERSE SENSE)
m_Atra_UR_21_9	-	m_Atra_UR_21_9	-	1.7e-42	137.5	5.0	1.9e-42	137.4	5.0	1.0	1	0	0	1	1	1	1	[209 - 9] (REVERSE SENSE)
a_Atra_UR_21_9	-	m_Atra_UR_21_9	-	4.4e-42	136.2	5.0	4.8e-42	136.1	5.0	1.0	1	0	0	1	1	1	1	[209 - 9] (REVERSE SENSE)
a_Atra_UR_21_10	-	m_Atra_UR_21_10	-	2.1e-13	42.0	0.6	2.1e-13	41.9	0.6	1.0	1	0	0	1	1	1	1	[70 - 2] (REVERSE SENSE)
m_Atra_UR_21_10	-	m_Atra_UR_21_10	-	2.1e-13	42.0	0.6	2.1e-13	41.9	0.6	1.0	1	0	0	1	1	1	1	[70 - 2] (REVERSE SENSE)
a_Atra_UR_22_1	-	m_Atra_UR_22_1	-	7.2e-14	42.5	2.5	7.5e-14	42.4	2.5	1.0	1	0	0	1	1	1	1	[7 - 78]
m_Atra_UR_22_1	-	m_Atra_UR_22_1	-	7.2e-14	42.5	2.5	7.5e-14	42.4	2.5	1.0	1	0	0	1	1	1	1	[7 - 78]
a_Atra_UR_22_6	-	m_Atra_UR_22_1	-	3.5e-06	21.1	0.7	3.5e-06	21.1	0.7	1.1	1	0	0	1	1	1	1	[292 - 357]
m_Atra_UR_22_6	-	m_Atra_UR_22_1	-	3.5e-06	21.1	0.7	3.5e-06	21.1	0.7	1.1	1	0	0	1	1	1	1	[292 - 357]
m_Atra_UR_22_2	-	m_Atra_UR_22_2	-	1.6e-15	48.3	4.5	1.7e-15	48.2	4.5	1.0	1	0	0	1	1	1	1	[9 - 86]
a_Atra_UR_22_2	-	m_Atra_UR_22_2	-	4.5e-15	47.0	4.5	4.7e-15	46.9	4.5	1.0	1	0	0	1	1	1	1	[9 - 86]

m_Atra_UR_22_7	-	m_Atra_UR_22_2	-	2.9e-06	21.8	0.6	2.9e-06	21.8	0.6	1.0	1	0	0	1	1	1	1	[318 - 365]
a_Atra_UR_22_7	-	m_Atra_UR_22_2	-	3.1e-06	21.7	0.6	3.1e-06	21.7	0.6	1.0	1	0	0	1	1	1	1	[318 - 365]
m_Atra_UR_22_3	-	m_Atra_UR_22_3	-	1.4e-17	54.4	3.8	1.4e-17	54.4	3.8	1.0	1	0	0	1	1	1	1	[111 - 197]
a_Atra_UR_22_3	-	m_Atra_UR_22_3	-	1.8e-17	54.1	2.9	1.8e-17	54.0	2.9	1.0	1	0	0	1	1	1	1	[111 - 197]
m_Atra_UR_22_4	-	m_Atra_UR_22_4	-	1.4e-38	124.2	11.4	1.5e-38	124.1	11.4	1.0	1	0	0	1	1	1	1	[35 - 220]
a_Atra_UR_22_4	-	m_Atra_UR_22_4	-	2.2e-38	123.6	10.8	2.4e-38	123.5	10.8	1.0	1	0	0	1	1	1	1	[35 - 220]
m_Atra_UR_22_8	-	m_Atra_UR_22_4	-	6.1e-05	19.1	0.8	7.8e-05	18.7	0.8	1.2	1	0	0	1	1	1	1	[269 - 382]
a_Atra_UR_22_8	-	m_Atra_UR_22_4	-	6.1e-05	19.0	0.8	7.8e-05	18.7	0.8	1.2	1	0	0	1	1	1	1	[269 - 382]
a_Atra_UR_22_5	-	m_Atra_UR_22_5	-	1.2e-12	38.9	1.1	1.2e-12	38.8	1.1	1.0	1	0	0	1	1	1	1	[213 - 275]
m_Atra_UR_22_5	-	m_Atra_UR_22_5	-	1.2e-12	38.9	1.1	1.2e-12	38.8	1.1	1.0	1	0	0	1	1	1	1	[213 - 275]
a_Atra_UR_22_6	-	m_Atra_UR_22_6	-	3.1e-13	40.9	2.5	3.2e-13	40.9	2.5	1.0	1	0	0	1	1	1	1	[292 - 357]
m_Atra_UR_22_6	-	m_Atra_UR_22_6	-	3.1e-13	40.9	2.5	3.2e-13	40.9	2.5	1.0	1	0	0	1	1	1	1	[292 - 357]
a_Atra_UR_22_1	-	m_Atra_UR_22_6	-	4.5e-06	20.9	1.1	4.6e-06	20.9	1.1	1.2	1	0	0	1	1	1	1	[7 - 78]
m_Atra_UR_22_1	-	m_Atra_UR_22_6	-	4.5e-06	20.9	1.1	4.6e-06	20.9	1.1	1.2	1	0	0	1	1	1	1	[7 - 78]
m_Atra_UR_22_7	-	m_Atra_UR_22_7	-	3.8e-10	30.6	2.4	3.8e-10	30.6	2.4	1.0	1	0	0	1	1	1	1	[318 - 365]
a_Atra_UR_22_7	-	m_Atra_UR_22_7	-	6.7e-10	30.0	1.7	6.7e-10	30.0	1.7	1.0	1	0	0	1	1	1	1	[318 - 365]
a_Atra_UR_22_2	-	m_Atra_UR_22_7	-	1.2e-06	21.4	1.4	2e-06	20.8	0.9	1.6	1	1	0	1	1	1	1	[9 - 86]
m_Atra_UR_22_2	-	m_Atra_UR_22_7	-	1.4e-06	21.2	1.7	2e-06	20.8	0.9	1.6	1	1	1	2	2	2	1	[9 - 86]
m_Atra_UR_22_8	-	m_Atra_UR_22_8	-	1.9e-25	81.5	4.5	2e-25	81.4	4.5	1.0	1	0	0	1	1	1	1	[269 - 382]
a_Atra_UR_22_8	-	m_Atra_UR_22_8	-	3.7e-25	80.6	4.1	4e-25	80.5	4.1	1.0	1	0	0	1	1	1	1	[269 - 382]
a_Atra_UR_22_4	-	m_Atra_UR_22_8	-	5.7e-05	19.1	0.7	5.7e-05	19.1	0.7	2.0	2	1	0	2	2	2	1	[35 - 220]
m_Atra_UR_22_4	-	m_Atra_UR_22_8	-	9.7e-05	18.4	0.8	9.7e-05	18.4	0.8	2.0	2	1	0	2	2	2	1	[35 - 220]
m_Atra_UR_22_9	-	m_Atra_UR_22_9	-	1.3e-07	22.7	0.2	1.3e-07	22.7	0.2	1.0	1	0	0	1	1	1	1	[398 - 436]
a_Atra_UR_22_9	-	m_Atra_UR_22_9	-	3.1e-07	21.7	0.1	3.1e-07	21.7	0.1	1.0	1	0	0	1	1	1	1	[398 - 436]
m_Atra_UR_22_10	-	m_Atra_UR_22_10	-	3.1e-14	44.4	0.5	3.2e-14	44.3	0.5	1.0	1	0	0	1	1	1	1	[385 - 453]
a_Atra_UR_22_10	-	m_Atra_UR_22_10	-	8.1e-14	43.2	0.5	8.2e-14	43.2	0.5	1.0	1	0	0	1	1	1	1	[385 - 453]
a_Atra_UR_22_11	-	m_Atra_UR_22_11	-	1.6e-08	26.2	1.4	1.6e-08	26.2	1.4	1.0	1	0	0	1	1	1	1	[429 - 391] (REVERSE SENSE)
m_Atra_UR_22_11	-	m_Atra_UR_22_11	-	1.6e-08	26.2	1.4	1.6e-08	26.2	1.4	1.0	1	0	0	1	1	1	1	[429 - 391] (REVERSE SENSE)
a_Atra_UR_22_12	-	m_Atra_UR_22_12	-	2.1e-21	68.9	6.8	2.2e-21	68.8	6.8	1.0	1	0	0	1	1	1	1	[427 - 323] (REVERSE SENSE)
m_Atra_UR_22_12	-	m_Atra_UR_22_12	-	2.1e-21	68.9	6.8	2.2e-21	68.8	6.8	1.0	1	0	0	1	1	1	1	[427 - 323] (REVERSE SENSE)
a_Atra_UR_22_21	-	m_Atra_UR_22_12	-	0.00013	18.1	3.6	0.00013	18.1	3.6	1.0	1	0	0	1	1	1	1	[94 - 47] (REVERSE SENSE)
m_Atra_UR_22_21	-	m_Atra_UR_22_12	-	0.00018	17.7	3.4	0.00018	17.7	3.4	1.0	1	0	0	1	1	1	1	[94 - 47] (REVERSE SENSE)
m_Atra_UR_22_13	-	m_Atra_UR_22_13	-	6.3e-14	44.7	2.5	6.6e-14	44.6	2.5	1.0	1	0	0	1	1	1	1	[369 - 304] (REVERSE SENSE)
a_Atra_UR_22_13	-	m_Atra_UR_22_13	-	1.4e-13	43.7	2.2	1.5e-13	43.6	2.2	1.0	1	0	0	1	1	1	1	[369 - 304] (REVERSE SENSE)
a_Atra_UR_22_22	-	m_Atra_UR_22_13	-	0.00012	17.6	0.8	0.00014	17.5	0.8	1.2	1	0	0	1	1	1	1	[99 - 25] (REVERSE SENSE)
m_Atra_UR_22_22	-	m_Atra_UR_22_13	-	0.00012	17.6	0.8	0.00014	17.5	0.8	1.2	1	0	0	1	1	1	1	[99 - 25] (REVERSE SENSE)
m_Atra_UR_22_14	-	m_Atra_UR_22_14	-	1.3e-20	66.0	5.5	1.4e-20	65.9	5.5	1.0	1	0	0	1	1	1	1	[356 - 261] (REVERSE SENSE)
a_Atra_UR_22_14	-	m_Atra_UR_22_14	-	2.9e-20	65.0	5.2	3e-20	64.9	5.2	1.0	1	0	0	1	1	1	1	[356 - 261] (REVERSE SENSE)
a_Atra_UR_22_15	-	m_Atra_UR_22_15	-	3e-07	23.1	1.0	3e-07	23.1	1.0	1.0	1	0	0	1	1	1	1	[270 - 238] (REVERSE SENSE)
m_Atra_UR_22_15	-	m_Atra_UR_22_15	-	3e-07	23.1	1.0	3e-07	23.1	1.0	1.0	1	0	0	1	1	1	1	[270 - 238] (REVERSE SENSE)
m_Atra_UR_22_16	-	m_Atra_UR_22_16	-	4.3e-07	22.6	1.7	4.3e-07	22.6	1.7	1.0	1	0	0	1	1	1	1	[256 - 212] (REVERSE SENSE)
a_Atra_UR_22_16	-	m_Atra_UR_22_16	-	1.4e-06	21.2	1.8	1.4e-06	21.2	1.8	1.0	1	0	0	1	1	1	1	[256 - 212] (REVERSE SENSE)
m_Atra_UR_22_17	-	m_Atra_UR_22_17	-	6.4e-09	27.5	0.6	6.4e-09	27.5	0.6	1.0	1	0	0	1	1	1	1	[242 - 198] (REVERSE SENSE)
a_Atra_UR_22_17	-	m_Atra_UR_22_17	-	1.9e-08	26.2	0.6	1.9e-08	26.2	0.6	1.0	1	0	0	1	1	1	1	[242 - 198] (REVERSE SENSE)
a_Atra_UR_22_18	-	m_Atra_UR_22_18	-	1.9e-12	40.0	5.3	1.9e-12	40.0	5.3	1.0	1	0	0	1	1	1	1	[198 - 133] (REVERSE SENSE)
m_Atra_UR_22_18	-	m_Atra_UR_22_18	-	1.9e-12	40.0	5.3	1.9e-12	40.0	5.3	1.0	1	0	0	1	1	1	1	[198 - 133] (REVERSE SENSE)
m_Atra_UR_22_19	-	m_Atra_UR_22_19	-	3.1e-13	42.9	6.5	3.2e-13	42.8	6.5	1.0	1	0	0	1	1	1	1	[194 - 129] (REVERSE SENSE)
a_Atra_UR_22_19	-	m_Atra_UR_22_19	-	6.2e-13	42.0	6.4	6.4e-13	41.9	6.4	1.0	1	0	0	1	1	1	1	[194 - 129] (REVERSE SENSE)
m_Atra_UR_22_20	-	m_Atra_UR_22_20	-	2.9e-19	62.8	15.3	3e-19	62.7	15.3	1.0	1	0	0	1	1	1	1	[205 - 110] (REVERSE SENSE)
a_Atra_UR_22_20	-	m_Atra_UR_22_20	-	6.8e-19	61.7	15.4	7.2e-19	61.6	15.4	1.0	1	0	0	1	1	1	1	[205 - 110] (REVERSE SENSE)
m_Atra_UR_22_21	-	m_Atra_UR_22_21	-	5.3e-09	29.0	4.6	5.3e-09	28.9	4.6	1.0	1	0	0	1	1	1	1	[94 - 47] (REVERSE SENSE)
a_Atra_UR_22_21	-	m_Atra_UR_22_21	-	1.6e-08	27.6	4.6	1.6e-08	27.6	4.6	1.0	1	0	0	1	1	1	1	[94 - 47] (REVERSE SENSE)
a_Atra_UR_22_12	-	m_Atra_UR_22_21	-	0.00012	16.9	2.9	0.00012	16.9	2.9	1.7	2	1	0	2	2	2	1	[427 - 323] (REVERSE SENSE)
m_Atra_UR_22_12	-	m_Atra_UR_22_21	-	0.00012	16.9	2.9	0.00012	16.9	2.9	1.7	2	1	0	2	2	2	1	[427 - 323] (REVERSE SENSE)
a_Atra_UR_22_22	-	m_Atra_UR_22_22	-	3.9e-15	47.9	3.0	4.1e-15	47.8	3.0	1.0	1	0	0	1	1	1	1	[99 - 25] (REVERSE SENSE)
m_Atra_UR_22_22	-	m_Atra_UR_22_22	-	3.9e-15	47.9	3.0	4.1e-15	47.8	3.0	1.0	1	0	0	1	1	1	1	[99 - 25] (REVERSE SENSE)
a_Atra_UR_22_13	-	m_Atra_UR_22_22	-	4.4e-05	18.6	0.6	4.4e-05	18.6	0.6	1.1	1	0	0	1	1	1	1	[369 - 304] (REVERSE SENSE)
m_Atra_UR_22_13	-	m_Atra_UR_22_22	-	8.1e-05	17.9	0.6	8.1e-05	17.9	0.6	1.1	1	0	0	1	1	1	1	[369 - 304] (REVERSE SENSE)
m_Atra_UR_22_23	-	m_Atra_UR_22_23	-	3e-12	38.8	1.8	3.1e-12	38.7	1.8	1.0	1	0	0	1	1	1	1	[62 - 3] (REVERSE SENSE)
a_Atra_UR_22_23	-	m_Atra_UR_22_23	-	6.1e-12	37.9	1.6	6.2e-12	37.9	1.6	1.0	1	0	0	1	1	1	1	[62 - 3] (REVERSE SENSE)
a_Atra_UR_23_1	-	m_Atra_UR_23_1	-	2.3e-06	19.1	2.6	2.3e-06	19.1	2.6	1.0	1	0	0	1	1	1	1	[24 - 56]
m_Atra_UR_23_1	-	m_Atra_UR_23_1	-	2.3e-06	19.1	2.6	2.3e-06	19.1	2.6	1.0	1	0	0	1	1	1	1	[24 - 56]
a_Atra_UR_23_2	-	m_Atra_UR_23_2	-	4.6e-13	40.6	2.4	4.8e-13	40.6	2.4	1.0	1	0	0	1	1	1	1	[22 - 87]
m_Atra_UR_23_2	-	m_Atra_UR_23_2	-	4.6e-13	40.6	2.4	4.8e-13	40.6	2.4	1.0	1	0	0	1	1	1	1	[22 - 87]
m_Atra_UR_23_3	-	m_Atra_UR_23_3	-	4.7e-07	21.8	2.7	4.7e-07	21.8	2.7	1.0	1	0	0	1	1	1	1	[51 - 16] (REVERSE SENSE)
a_Atra_UR_23_3	-	m_Atra_UR_23_3	-	9.7e-07	21.0	2.2	9.7e-07	21.0	2.2	1.0	1	0	0	1	1	1	1	[51 - 16] (REVERSE SENSE)
m_Atra_UR_23_4	-	m_Atra_UR_23_4	-	4.7e-17	54.6	5.9	4.9e-17	54.5	5.9	1.0	1	0	0	1	1	1	1	[80 - 3] (REVERSE SENSE)
a_Atra_UR_23_4	-	m_Atra_UR_23_4	-	1.4e-16	53.2	6.0	1.4e-16	53.1	6.0	1.0	1	0	0	1	1	1	1	[80 - 3] (REVERSE SENSE)
m_Atra_UR_23_5	-	m_Atra_UR_23_5	-	3.3e-05	14.7	0.2	3.3e-05	14.7	0.2	1.0	1	0	0	1	1	1	1	[31 - 2] (REVERSE SENSE)
a_Atra_UR_23_5	-	m_Atra_UR_23_5	-	0.00013	13.4	0.1	0.00013	13.4	0.1	1.0	1	0	0	1	1	1	1	[31 - 2] (REVERSE SENSE)
m_Mdub_UR_4_1	-	m_Mdub_UR_4_1	-	7.1e-06	18.1	1.2	7.1e-06	18.1	1.2	1.0	1	0	0	1	1	1	1	[16 - 45]
a_Mdub_UR_4_1	-	m_Mdub_UR_4_1	-	1.9e-05	17.0	0.9	1.9e-05	17.0	0.9	1.0	1	0	0	1	1	1	1	[16 - 45]
m_Mdub_UR_4_2	-	m_Mdub_UR_4_2	-	2.5e-20	64.4	5.3	2.7e-20	64.3	5.3	1.0	1	0	0	1	1	1	1	[9 - 116]
a_Mdub_UR_4_2	-	m_Mdub_UR_4_2	-	4.7e-20	63.6	4.9	5e-20	63.5	4.9	1.0	1	0	0	1	1	1	1	[9 - 116]
m_Mdub_UR_4_3	-	m_Mdub_UR_4_3	-	1.3e-05	16.6	0.5	1.3e-05	16.6	0.5	1.0	1	0	0	1	1	1	1	[77 - 48] (REVERSE SENSE)
a_Mdub_UR_4_3	-	m_Mdub_UR_4_3	-	4.4e-05	15.3	0.4	4.4e-05	15.3	0.4	1.0	1	0	0	1	1	1	1	[77 - 48] (REVERSE SENSE)
m_Mdub_UR_4_4	-	m_Mdub_UR_4_4	-	4.8e-15	48.0	3.8	4.9e-15	47.9	3.8	1.0	1	0	0	1	1	1	1	[115 - 41] (REVERSE SENSE)
a_Mdub_UR_4_4	-	m_Mdub_UR_4_4	-	1.5e-14	46.5	3.9	1.5e-14	46.5	3.9	1.0	1	0	0	1	1	1	1	[115 - 41] (REVERSE SENSE)
m_Mdub_UR_15_1	-	m_Mdub_UR_15_1	-	5.9e-06	17.8	1.4	5.9e-06	17.8	1.4	1.0	1	0	0	1	1	1	1	[26 - 64]
a_Mdub_UR_15_1	-	m_Mdub_UR_15_1	-	1.6e-05	16.8	1.1	1.6e-05	16.8	1.1	1.0	1	0	0	1	1	1	1	[26 - 64]
m_Mdub_UR_15_2	-	m_Mdub_UR_15_2	-	1.8e-07	20.8	0.2	1.8e-07	20.8	0.2	1.0	1	0	0	1	1	1	1	[75 - 110]
a_Mdub_UR_15_2	-	m_Mdub_UR_15_2	-	5.8e-07	19.6	0.1	5.8e-07	19.6	0.1	1.0	1	0	0	1	1	1	1	[75 - 110]
a_Mdub_UR_15_3	-	m_Mdub_UR_15_3	-	1.6e-06	18.7	0.4	1.6e-06	18.7	0.4	1.0	1	0	0	1	1	1	1	[95 - 130]
m_Mdub_UR_15_3	-	m_Mdub_UR_15_3	-	1.6e-06	18.7	0.4	1.6e-06	18.7	0.4	1.0	1	0	0	1	1	1	1	[95 - 130]
m_Mdub_UR_15_4	-	m_Mdub_UR_15_4	-	2.5e-05	16.3	1.3	2.5e-05	16.3	1.3	1.0	1	0	0	1	1	1	1	[134 - 163]
a_Mdub_UR_15_4	-	m_Mdub_UR_15_4	-	6e-05	15.4	0.9	6e-05	15.4	0.9	1.0	1	0	0	1	1	1	1	[134 - 163]
a_Mdub_UR_15_5	-	m_Mdub_UR_15_5	-	3.2e-31	100.0	2.5	3.4e-31	99.9	2.5	1.0	1	0	0	1	1	1	1	[88 - 231]
m_Mdub_UR_15_5	-	m_Mdub_UR_15_5	-	3.2e-31	100.0	2.5	3.4e-31	99.9	2.5	1.0	1	0	0	1	1	1	1	[88 - 231]
m_Mdub_UR_15_6	-	m_Mdub_UR_15_6	-	4e-10	30.8	0.5	4e-10	30.8	0.5	1.0	1	0	0	1	1	1	1	[227 - 274]
a_Mdub_UR_15_6	-	m_Mdub_UR_15_6	-	1.1e-09	29.6	0.4	1.1e-09	29.6	0.4	1.0	1	0	0	1	1	1	1	[227 - 274]
m_Mdub_UR_15_7	-	m_Mdub_UR_15_7	-	2.1e-17	54.6	0.1	2.2e-17	54.6	0.1	1.0	1	0	0	1	1	1	1	[238 - 318]
a_Mdub_UR_15_7	-	m_Mdub_UR_15_7	-	4.6e-17	53.7	0.1	4.8e-17	53.6	0.1	1.0	1	0	0	1	1	1	1	[238 - 318]
m_Mdub_UR_15_8	-	m_Mdub_UR_15_8	-	1.7e-18	58.8	1.4	1.7e-18	58.8	1.4	1.0	1	0	0	1	1	1	1	[358 - 444]
a_Mdub_UR_15_8	-	m_Mdub_UR_15_8	-	3.7e-18	57.8	1.2	3.9e-18	57.7	1.2	1.0	1	0	0	1	1	1	1	[358 - 444]
a_Mdub_UR_15_9	-	m_Mdub_UR_15_9	-	2e-40	130.8	4.9	2.1e-40	130.7	4.9	1.0	1	0	0	1	1	1	1	[299 - 466]
m_Mdub_UR_15_9	-	m_Mdub_UR_15_9	-	2e-40	130.8	4.9	2.1e-40	130.7	4.9	1.0	1	0	0	1	1	1	1	[299 - 466]
m_Mdub_UR_15_10	-	m_Mdub_UR_15_10	-	8.9e-59	191.3	9.7	9.7e-59	191.1	9.7	1.0	1	0	0	1	1	1	1	[234 - 485]
a_Mdub_UR_15_10	-	m_Mdub_UR_15_10	-	2.2e-58	190.0	9.6	2.4e-58	189.9	9.6	1.0	1	0	0	1	1	1	1	[234 - 485]
m_Mdub_UR_15_11	-	m_Mdub_UR_15_11	-	2.6e-20	65.2	3.0	2.8e-20	65.1	3.0	1.0	1	0	0	1	1	1	1	[539 - 625]
a_Mdub_UR_15_11	-	m_Mdub_UR_15_11	-	6.7e-20	64.0	3.0	7e-20	63.9	3.0	1.0	1	0	0	1	1	1	1	[539 - 625]
a_Mdub_UR_15_12	-	m_Mdub_UR_15_12	-	7.9e-36	115.9	4.1	8.6e-36	115.7	4.1	1.0	1	0	0	1	1	1	1	[514 - 669]
m_Mdub_UR_15_12	-	m_Mdub_UR_15_12	-	7.9e-36	115.9	4.1	8.6e-36	115.7	4.1	1.0	1	0	0	1	1	1	1	[514 - 669]
a_Mdub_UR_15_13	-	m_Mdub_UR_15_13	-	2.6e-06	19.3	0.3	2.6e-06	19.3	0.3	1.0	1	0	0	1	1	1	1	[716 - 745]
m_Mdub_UR_15_13	-	m_Mdub_UR_15_13	-	2.6e-06	19.3	0.3	2.6e-06	19.3	0.3	1.0	1	0	0	1	1	1	1	[716 - 745]
m_Mdub_UR_15_14	-	m_Mdub_UR_15_14	-	5.3e-08	24.0	0.7	5.3e-08	24.0	0.7	1.0	1	0	0	1	1	1	1	[752 - 790]
a_Mdub_UR_15_14	-	m_Mdub_UR_15_14	-	1.3e-07	23.0	0.4	1.3e-07	23.0	0.4	1.0	1	0	0	1	1	1	1	[752 - 790]
m_Mdub_UR_15_15	-	m_Mdub_UR_15_15	-	8e-60	194.9	10.7	8.7e-60	194.7	10.7	1.0	1	0	0	1	1	1	1	[537 - 797]
a_Mdub_UR_15_15	-	m_Mdub_UR_15_15	-	1.8e-59	193.8	10.5	1.9e-59	193.6	10.5	1.0	1	0	0	1	1	1	1	[537 - 797]
m_Mdub_UR_15_16	-	m_Mdub_UR_15_16	-	2e-06	19.7	1.4	2e-06	19.7	1.4	1.0	1	0	0	1	1	1	1	[807 - 839]

a_Mdub_UR_15_16	-	m_Mdub_UR_15_16	-	5e-06	18.7	1.1	5e-06	18.7	1.1	1.0	1	0	0	1	1	1	1	[807 - 839]
m_Mdub_UR_15_17	-	m_Mdub_UR_15_17	-	2.6e-48	156.1	3.1	2.9e-48	156.0	3.1	1.0	1	0	0	1	1	1	1	[691 - 897]
a_Mdub_UR_15_17	-	m_Mdub_UR_15_17	-	4.7e-48	155.3	2.9	5.1e-48	155.2	2.9	1.0	1	0	0	1	1	1	1	[691 - 897]
m_Mdub_UR_15_18	-	m_Mdub_UR_15_18	-	7.5e-10	29.9	1.2	7.5e-10	29.9	1.2	1.0	1	0	0	1	1	1	1	[858 - 905]
a_Mdub_UR_15_18	-	m_Mdub_UR_15_18	-	1.9e-09	28.8	1.0	1.9e-09	28.8	1.0	1.0	1	0	0	1	1	1	1	[858 - 905]
m_Mdub_UR_15_19	-	m_Mdub_UR_15_19	-	8.4e-10	30.0	2.8	8.5e-10	30.0	2.8	1.0	1	0	0	1	1	1	1	[893 - 846] (REVERSE SENSE)
a_Mdub_UR_15_19	-	m_Mdub_UR_15_19	-	1.9e-09	29.0	2.5	1.9e-09	29.0	2.5	1.0	1	0	0	1	1	1	1	[893 - 846] (REVERSE SENSE)
a_Mdub_UR_15_20	-	m_Mdub_UR_15_20	-	5.2e-12	37.4	1.0	5.2e-12	37.4	1.0	1.0	1	0	0	1	1	1	1	[846 - 793] (REVERSE SENSE)
m_Mdub_UR_15_20	-	m_Mdub_UR_15_20	-	5.2e-12	37.4	1.0	5.2e-12	37.4	1.0	1.0	1	0	0	1	1	1	1	[846 - 793] (REVERSE SENSE)
m_Mdub_UR_15_21	-	m_Mdub_UR_15_21	-	3.8e-14	44.3	0.1	3.9e-14	44.2	0.1	1.0	1	0	0	1	1	1	1	[824 - 756] (REVERSE SENSE)
a_Mdub_UR_15_21	-	m_Mdub_UR_15_21	-	1.1e-13	43.0	0.2	1.1e-13	43.0	0.2	1.0	1	0	0	1	1	1	1	[824 - 756] (REVERSE SENSE)
a_Mdub_UR_15_22	-	m_Mdub_UR_15_22	-	3.6e-09	27.6	0.3	3.6e-09	27.6	0.3	1.0	1	0	0	1	1	1	1	[766 - 725] (REVERSE SENSE)
m_Mdub_UR_15_22	-	m_Mdub_UR_15_22	-	3.6e-09	27.6	0.3	3.6e-09	27.6	0.3	1.0	1	0	0	1	1	1	1	[766 - 725] (REVERSE SENSE)
m_Mdub_UR_15_23	-	m_Mdub_UR_15_23	-	1.2e-15	48.3	0.9	1.2e-15	48.3	0.9	1.0	1	0	0	1	1	1	1	[762 - 685] (REVERSE SENSE)
a_Mdub_UR_15_23	-	m_Mdub_UR_15_23	-	2.4e-15	47.4	0.5	2.5e-15	47.4	0.5	1.0	1	0	0	1	1	1	1	[762 - 685] (REVERSE SENSE)
m_Mdub_UR_15_24	-	m_Mdub_UR_15_24	-	1.5e-29	94.9	3.3	1.6e-29	94.8	3.3	1.0	1	0	0	1	1	1	1	[641 - 510] (REVERSE SENSE)
a_Mdub_UR_15_24	-	m_Mdub_UR_15_24	-	2.6e-29	94.2	2.8	2.8e-29	94.1	2.8	1.0	1	0	0	1	1	1	1	[641 - 510] (REVERSE SENSE)
m_Mdub_UR_15_25	-	m_Mdub_UR_15_25	-	2.9e-19	61.4	0.8	3e-19	61.3	0.8	1.0	1	0	0	1	1	1	1	[465 - 385] (REVERSE SENSE)
a_Mdub_UR_15_25	-	m_Mdub_UR_15_25	-	6e-19	60.4	0.8	6.2e-19	60.4	0.8	1.0	1	0	0	1	1	1	1	[465 - 385] (REVERSE SENSE)
a_Mdub_UR_15_26	-	m_Mdub_UR_15_26	-	2.6e-20	65.2	10.1	2.8e-20	65.1	10.1	1.0	1	0	0	1	1	1	1	[437 - 336] (REVERSE SENSE)
m_Mdub_UR_15_26	-	m_Mdub_UR_15_26	-	2.6e-20	65.2	10.1	2.8e-20	65.1	10.1	1.0	1	0	0	1	1	1	1	[437 - 336] (REVERSE SENSE)
a_Mdub_UR_15_27	-	m_Mdub_UR_15_27	-	2.4e-85	278.8	9.0	2.7e-85	278.7	9.0	1.0	1	0	0	1	1	1	1	[667 - 293] (REVERSE SENSE)
m_Mdub_UR_15_27	-	m_Mdub_UR_15_27	-	2.4e-85	278.8	9.0	2.7e-85	278.7	9.0	1.0	1	0	0	1	1	1	1	[667 - 293] (REVERSE SENSE)
a_Mdub_UR_15_28	-	m_Mdub_UR_15_28	-	5.1e-12	37.2	0.1	5.2e-12	37.1	0.1	1.0	1	0	0	1	1	1	1	[293 - 234] (REVERSE SENSE)
m_Mdub_UR_15_28	-	m_Mdub_UR_15_28	-	5.1e-12	37.2	0.1	5.2e-12	37.1	0.1	1.0	1	0	0	1	1	1	1	[293 - 234] (REVERSE SENSE)
m_Mdub_UR_15_29	-	m_Mdub_UR_15_29	-	2.4e-12	37.9	1.1	2.5e-12	37.8	1.1	1.0	1	0	0	1	1	1	1	[286 - 227] (REVERSE SENSE)
a_Mdub_UR_15_29	-	m_Mdub_UR_15_29	-	4.7e-12	37.0	0.9	4.8e-12	37.0	0.9	1.0	1	0	0	1	1	1	1	[286 - 227] (REVERSE SENSE)
m_Mdub_UR_15_30	-	m_Mdub_UR_15_30	-	1.4e-43	140.8	3.8	1.5e-43	140.7	3.8	1.0	1	0	0	1	1	1	1	[375 - 190] (REVERSE SENSE)
a_Mdub_UR_15_30	-	m_Mdub_UR_15_30	-	3e-43	139.7	3.9	3.2e-43	139.6	3.9	1.0	1	0	0	1	1	1	1	[375 - 190] (REVERSE SENSE)
m_Mdub_UR_15_31	-	m_Mdub_UR_15_31	-	1.2e-06	19.4	1.7	1.2e-06	19.4	1.7	1.0	1	0	0	1	1	1	1	[206 - 174] (REVERSE SENSE)
a_Mdub_UR_15_31	-	m_Mdub_UR_15_31	-	2.4e-06	18.7	1.1	2.4e-06	18.7	1.1	1.0	1	0	0	1	1	1	1	[206 - 174] (REVERSE SENSE)
m_Mdub_UR_15_32	-	m_Mdub_UR_15_32	-	4.8e-05	15.0	1.8	4.8e-05	15.0	1.8	1.1	1	0	0	1	1	1	1	[170 - 138] (REVERSE SENSE)
a_Mdub_UR_15_32	-	m_Mdub_UR_15_32	-	8.3e-05	14.4	1.4	8.3e-05	14.4	1.4	1.1	1	0	0	1	1	1	1	[170 - 138] (REVERSE SENSE)
m_Mdub_UR_15_33	-	m_Mdub_UR_15_33	-	6.9e-10	30.2	0.2	6.9e-10	30.2	0.2	1.0	1	0	0	1	1	1	1	[180 - 133] (REVERSE SENSE)
a_Mdub_UR_15_33	-	m_Mdub_UR_15_33	-	1.6e-09	29.2	0.2	1.6e-09	29.2	0.2	1.0	1	0	0	1	1	1	1	[180 - 133] (REVERSE SENSE)
m_Mdub_UR_15_34	-	m_Mdub_UR_15_34	-	1.5e-07	23.3	0.8	1.5e-07	23.3	0.8	1.0	1	0	0	1	1	1	1	[109 - 74] (REVERSE SENSE)
a_Mdub_UR_15_34	-	m_Mdub_UR_15_34	-	3e-07	22.5	0.6	3e-07	22.5	0.6	1.0	1	0	0	1	1	1	1	[109 - 74] (REVERSE SENSE)
m_Mdub_UR_15_35	-	m_Mdub_UR_15_35	-	2.3e-09	29.2	2.8	2.3e-09	29.2	2.8	1.0	1	0	0	1	1	1	1	[89 - 42] (REVERSE SENSE)
a_Mdub_UR_15_35	-	m_Mdub_UR_15_35	-	4.4e-09	28.4	2.3	4.4e-09	28.4	2.3	1.0	1	0	0	1	1	1	1	[89 - 42] (REVERSE SENSE)
a_Mdub_UR_15_36	-	m_Mdub_UR_15_36	-	1.4e-07	22.5	0.4	1.4e-07	22.5	0.4	1.0	1	0	0	1	1	1	1	[63 - 22] (REVERSE SENSE)
m_Mdub_UR_15_36	-	m_Mdub_UR_15_36	-	1.4e-07	22.5	0.4	1.4e-07	22.5	0.4	1.0	1	0	0	1	1	1	1	[63 - 22] (REVERSE SENSE)
m_Mdub_UR_16_1	-	m_Mdub_UR_16_1	-	1.5e-06	19.7	0.3	1.5e-06	19.7	0.3	1.0	1	0	0	1	1	1	1	[45 - 74]
a_Mdub_UR_16_1	-	m_Mdub_UR_16_1	-	4.8e-06	18.4	0.3	4.8e-06	18.4	0.3	1.0	1	0	0	1	1	1	1	[45 - 74]
m_Mdub_UR_16_2	-	m_Mdub_UR_16_2	-	1.6e-08	26.7	3.0	1.6e-08	26.7	3.0	1.0	1	0	0	1	1	1	1	[40 - 90]
a_Mdub_UR_16_2	-	m_Mdub_UR_16_2	-	3.1e-08	26.0	2.5	3.1e-08	26.0	2.5	1.0	1	0	0	1	1	1	1	[40 - 90]
a_Mdub_UR_16_3	-	m_Mdub_UR_16_3	-	4.2e-06	18.5	1.3	4.2e-06	18.5	1.3	1.0	1	0	0	1	1	1	1	[81 - 110]
m_Mdub_UR_16_3	-	m_Mdub_UR_16_3	-	4.2e-06	18.5	1.3	4.2e-06	18.5	1.3	1.0	1	0	0	1	1	1	1	[81 - 110]
a_Mdub_UR_16_4	-	m_Mdub_UR_16_4	-	1.3e-06	20.0	2.2	1.3e-06	20.0	2.2	1.0	1	0	0	1	1	1	1	[98 - 136]
m_Mdub_UR_16_4	-	m_Mdub_UR_16_4	-	1.3e-06	20.0	2.2	1.3e-06	20.0	2.2	1.0	1	0	0	1	1	1	1	[98 - 136]
m_Mdub_UR_16_5	-	m_Mdub_UR_16_5	-	4.3e-07	22.4	4.8	4.3e-07	22.4	4.8	1.0	1	0	0	1	1	1	1	[137 - 93] (REVERSE SENSE)
a_Mdub_UR_16_5	-	m_Mdub_UR_16_5	-	7e-07	21.8	4.4	7e-07	21.8	4.4	1.0	1	0	0	1	1	1	1	[137 - 93] (REVERSE SENSE)
m_Mdub_UR_16_6	-	m_Mdub_UR_16_6	-	1.3e-08	26.8	1.5	1.3e-08	26.8	1.5	1.0	1	0	0	1	1	1	1	[132 - 88] (REVERSE SENSE)
a_Mdub_UR_16_6	-	m_Mdub_UR_16_6	-	4e-08	25.5	1.5	4e-08	25.5	1.5	1.0	1	0	0	1	1	1	1	[132 - 88] (REVERSE SENSE)
m_Mdub_UR_16_7	-	m_Mdub_UR_16_7	-	5.8e-15	46.5	0.7	6.1e-15	46.5	0.7	1.0	1	0	0	1	1	1	1	[89 - 12] (REVERSE SENSE)
a_Mdub_UR_16_7	-	m_Mdub_UR_16_7	-	1.5e-14	45.4	0.5	1.5e-14	45.4	0.5	1.0	1	0	0	1	1	1	1	[89 - 12] (REVERSE SENSE)
m_Mdub_UR_16_8	-	m_Mdub_UR_16_8	-	9e-12	37.6	2.7	9.2e-12	37.6	2.7	1.0	1	0	0	1	1	1	1	[67 - 8] (REVERSE SENSE)
a_Mdub_UR_16_8	-	m_Mdub_UR_16_8	-	1.7e-11	36.8	2.5	1.8e-11	36.8	2.5	1.0	1	0	0	1	1	1	1	[67 - 8] (REVERSE SENSE)
m_Mdub_UR_16_9	-	m_Mdub_UR_16_9	-	3.8e-06	18.4	0.3	3.8e-06	18.4	0.3	1.0	1	0	0	1	1	1	1	[30 - 1] (REVERSE SENSE)
a_Mdub_UR_16_9	-	m_Mdub_UR_16_9	-	1.2e-05	17.1	0.2	1.2e-05	17.1	0.2	1.0	1	0	0	1	1	1	1	[30 - 1] (REVERSE SENSE)
m_Mdub_UR_23_1	-	m_Mdub_UR_23_1	-	2.2e-05	16.7	1.7	2.2e-05	16.7	1.7	1.0	1	0	0	1	1	1	1	[35 - 3] (REVERSE SENSE)
a_Mdub_UR_23_1	-	m_Mdub_UR_23_1	-	4.7e-05	15.9	1.5	4.7e-05	15.9	1.5	1.1	1	0	0	1	1	1	1	[35 - 3] (REVERSE SENSE)
a_HmenF_UR_1_1	-	m_HmenF_UR_1_1	-	5.4e-08	25.4	3.8	5.4e-08	25.4	3.8	1.0	1	0	0	1	1	1	1	[34 - 69]
m_HmenF_UR_1_1	-	m_HmenF_UR_1_1	-	5.4e-08	25.4	3.8	5.4e-08	25.4	3.8	1.0	1	0	0	1	1	1	1	[34 - 69]
m_HmenF_UR_1_2	-	m_HmenF_UR_1_2	-	1.6e-07	22.9	0.8	1.6e-07	22.9	0.8	1.0	1	0	0	1	1	1	1	[36 - 71]
a_HmenF_UR_1_2	-	m_HmenF_UR_1_2	-	3.7e-07	21.9	0.5	3.7e-07	21.9	0.5	1.0	1	0	0	1	1	1	1	[36 - 71]
m_HmenF_UR_1_3	-	m_HmenF_UR_1_3	-	4e-05	16.9	3.1	4e-05	16.9	3.1	1.0	1	0	0	1	1	1	1	[69 - 40] (REVERSE SENSE)
a_HmenF_UR_1_3	-	m_HmenF_UR_1_3	-	0.00017	15.3	3.4	0.00017	15.3	3.4	1.1	1	0	0	1	1	1	1	[69 - 40] (REVERSE SENSE)
m_HmenF_UR_1_4	-	m_HmenF_UR_1_4	-	2.3e-13	42.6	3.5	2.4e-13	42.5	3.5	1.0	1	0	0	1	1	1	1	[62 - 3] (REVERSE SENSE)
a_HmenF_UR_1_4	-	m_HmenF_UR_1_4	-	4.7e-13	41.7	3.4	4.8e-13	41.7	3.4	1.0	1	0	0	1	1	1	1	[62 - 3] (REVERSE SENSE)
a_HmenF_UR_1_5	-	m_HmenF_UR_1_5	-	4.2e-12	39.3	6.7	4.3e-12	39.2	6.7	1.0	1	0	0	1	1	1	1	[55 - 2] (REVERSE SENSE)
m_HmenF_UR_1_5	-	m_HmenF_UR_1_5	-	4.2e-12	39.3	6.7	4.3e-12	39.2	6.7	1.0	1	0	0	1	1	1	1	[55 - 2] (REVERSE SENSE)
m_HmenF_UR_2_1	-	m_HmenF_UR_2_1	-	3e-07	22.5	0.3	3e-07	22.5	0.3	1.0	1	0	0	1	1	1	1	[36 - 1] (REVERSE SENSE)
a_HmenF_UR_2_1	-	m_HmenF_UR_2_1	-	7e-07	21.6	0.2	7e-07	21.6	0.2	1.0	1	0	0	1	1	1	1	[36 - 1] (REVERSE SENSE)
a_HmenF_UR_5_1	-	m_HmenF_UR_5_1	-	4.1e-07	21.3	1.1	4.1e-07	21.3	1.1	1.0	1	0	0	1	1	1	1	[11 - 43]
m_HmenF_UR_5_1	-	m_HmenF_UR_5_1	-	4.1e-07	21.3	1.1	4.1e-07	21.3	1.1	1.0	1	0	0	1	1	1	1	[11 - 43]
m_HmenF_UR_5_2	-	m_HmenF_UR_5_2	-	2.2e-09	30.8	8.7	2.2e-09	30.8	8.7	1.0	1	0	0	1	1	1	1	[43 - 2] (REVERSE SENSE)
a_HmenF_UR_5_2	-	m_HmenF_UR_5_2	-	3e-09	30.4	7.7	3e-09	30.4	7.7	1.0	1	0	0	1	1	1	1	[43 - 2] (REVERSE SENSE)
a_HmenF_UR_5_3	-	m_HmenF_UR_5_3	-	1.7e-05	16.5	0.2	1.7e-05	16.5	0.2	1.0	1	0	0	1	1	1	1	[30 - 1] (REVERSE SENSE)
m_HmenF_UR_5_3	-	m_HmenF_UR_5_3	-	1.7e-05	16.5	0.2	1.7e-05	16.5	0.2	1.0	1	0	0	1	1	1	1	[30 - 1] (REVERSE SENSE)
m_HmenF_UR_8_1	-	m_HmenF_UR_8_1	-	2.4e-06	18.7	0.6	2.4e-06	18.7	0.6	1.0	1	0	0	1	1	1	1	[3 - 32]
a_HmenF_UR_8_1	-	m_HmenF_UR_8_1	-	6.9e-06	17.6	0.4	6.9e-06	17.6	0.4	1.0	1	0	0	1	1	1	1	[3 - 32]
m_HmenF_UR_8_2	-	m_HmenF_UR_8_2	-	4e-07	22.3	1.1	4e-07	22.3	1.1	1.0	1	0	0	1	1	1	1	[32 - 3] (REVERSE SENSE)
a_HmenF_UR_8_2	-	m_HmenF_UR_8_2	-	1.1e-06	21.2	0.9	1.1e-06	21.2	0.9	1.0	1	0	0	1	1	1	1	[32 - 3] (REVERSE SENSE)
m_HmenF_UR_10_1	-	m_HmenF_UR_10_1	-	5.3e-08	25.2	2.2	5.3e-08	25.2	2.2	1.0	1	0	0	1	1	1	1	[41 - 3] (REVERSE SENSE)
a_HmenF_UR_10_1	-	m_HmenF_UR_10_1	-	1.1e-07	24.4	2.0	1.1e-07	24.4	2.0	1.0	1	0	0	1	1	1	1	[41 - 3] (REVERSE SENSE)
m_HmenF_UR_13_1	-	m_HmenF_UR_13_1	-	3.5e-08	24.1	0.1	3.5e-08	24.1	0.1	1.0	1	0	0	1	1	1	1	[3 - 44]
a_HmenF_UR_13_1	-	m_HmenF_UR_13_1	-	1.1e-07	22.8	0.1	1.1e-07	22.8	0.1	1.0	1	0	0	1	1	1	1	[3 - 44]
m_HmenF_UR_13_2	-	m_HmenF_UR_13_2	-	5.5e-09	28.6	1.7	5.5e-09	28.6	1.7	1.0	1	0	0	1	1	1	1	[38 - 3] (REVERSE SENSE)
a_HmenF_UR_13_2	-	m_HmenF_UR_13_2	-	9.7e-09	28.0	1.3	9.7e-09	28.0	1.3	1.0	1	0	0	1	1	1	1	[38 - 3] (REVERSE SENSE)
a_HmenF_UR_13_3	-	m_HmenF_UR_13_3	-	1.8e-05	15.3	0.3	1.8e-05	15.3	0.3	1.0	1	0	0	1	1	1	1	[31 - 2] (REVERSE SENSE)
m_HmenF_UR_13_3	-	m_HmenF_UR_13_3	-	1.8e-05	15.3	0.3	1.8e-05	15.3	0.3	1.0	1	0	0	1	1	1	1	[31 - 2] (REVERSE SENSE)
m_HmenF_UR_16_1	-	m_HmenF_UR_16_1	-	2.5e-08	26.4	1.4	2.5e-08	26.4	1.4	1.0	1	0	0	1	1	1	1	[39 - 1] (REVERSE SENSE)
a_HmenF_UR_16_1	-	m_HmenF_UR_16_1	-	6.5e-08	25.3	1.2	6.5e-08	25.3	1.2	1.0	1	0	0	1	1	1	1	[39 - 1] (REVERSE SENSE)
a_HmenF_UR_17_1	-	m_HmenF_UR_17_1	-	7.9e-06	17.3	0.1	7.9e-06	17.3	0.1	1.0	1	0	0	1	1	1	1	[35 - 64]
m_HmenF_UR_17_1	-	m_HmenF_UR_17_1	-	7.9e-06	17.3	0.1	7.9e-06	17.3	0.1	1.0	1	0	0	1	1	1	1	[35 - 64]
m_HmenF_UR_17_2	-	m_HmenF_UR_17_2	-	2e-11	34.4	0.3	2.1e-11	34.4	0.3	1.0	1	0	0	1	1	1	1	[69 - 122]
a_HmenF_UR_17_2	-	m_HmenF_UR_17_2	-	5.6e-11	33.2	0.2	5.6e-11	33.2	0.2	1.0	1	0	0	1	1	1	1	[69 - 122]
m_HmenF_UR_17_3	-	m_HmenF_UR_17_3	-	5.6e-19	59.6	1.7	5.9e-19	59.5	1.7	1.0	1	0	0	1	1	1	1	[37 - 129]
a_HmenF_UR_17_3	-	m_HmenF_UR_17_3	-	1.2e-18	58.6	1.4	1.2e-18	58.6	1.4	1.0	1	0	0	1	1	1	1	[37 - 129]
a_HmenF_UR_17_4	-	m_HmenF_UR_17_4	-	5.2e-13	41.2	0.6	5.3e-13	41.1	0.6	1.0	1	0	0	1	1	1	1	[119 - 60] (REVERSE SENSE)
m_HmenF_UR_17_4	-	m_HmenF_UR_17_4	-	5.2e-13	41.2	0.6	5.3e-13	41.1	0.6	1.0	1	0	0	1	1	1	1	[119 - 60] (REVERSE SENSE)
m_HmenF_UR_17_5	-	m_HmenF_UR_17_5	-	5.5e-17	53.8	0.3	5.7e-17	53.7	0.3	1.0	1	0	0	1	1	1	1	[100 - 26] (REVERSE SENSE)
a_HmenF_UR_17_5	-	m_HmenF_UR_17_5	-	1.2e-16	52.8	0.3	1.2e-16	52.8	0.3	1.0	1	0	0	1	1	1	1	[100 - 26] (REVERSE SENSE)
m_HmenF_UR_17_6	-	m_HmenF_UR_17_6	-	1.3e-07	23.5	0.3	1.3e-07	23.5	0.3	1.0	1	0	0	1	1	1	1	[48 - 10] (REVERSE SENSE)
a_HmenF_UR_17_6	-	m_HmenF_UR_17_6	-	2.9e-07	22.6	0.2	2.9e-07	22.6	0.2	1.0	1	0	0	1	1	1	1	[48 - 10] (REVERSE SENSE)
m_HmenF_UR_21_1	-	m_HmenF_UR_21_1	-	8e-07	21.4	0.9	8e-07	21.4	0.9	1.0	1	0	0	1	1	1	1	[39 - 4] (REVERSE SENSE)
a_HmenF_UR_21_1	-	m_HmenF_UR_21_1	-	1.8e-06	20.5	0.9	1.8e-06	20.5	0.9	1.0	1	0	0	1	1	1	1	[39 - 4] (REVERSE SENSE)
a_HmenF_UR_24_1	-	m_HmenF_UR_24_1	-	3.2e-10	32.7	6.4	3.2e-10	32.7	6.4	1.0	1	0	0	1	1	1	1	[6 - 62]
m_HmenF_UR_24_1	-	m_HmenF_UR_24_1	-	3.2e-10	32.7	6.4	3.2e-10	32.7	6.4	1.0	1	0	0	1	1	1	1	[6 - 62]

m_HmenF_UR_24_2	-	m_HmenF_UR_24_2	-	1.5e-10	33.9	5.5	1.6e-10	33.9	5.5	1.0	1	0	0	1	1	1	1	[25 - 75]
a_HmenF_UR_24_2	-	m_HmenF_UR_24_2	-	2.8e-10	33.2	5.0	2.8e-10	33.2	5.0	1.0	1	0	0	1	1	1	1	[25 - 75]
m_HmenF_UR_24_3	-	m_HmenF_UR_24_3	-	1.6e-07	22.6	0.6	1.6e-07	22.6	0.6	1.0	1	0	0	1	1	1	1	[53 - 88]
a_HmenF_UR_24_3	-	m_HmenF_UR_24_3	-	4.8e-07	21.4	0.6	4.8e-07	21.4	0.6	1.0	1	0	0	1	1	1	1	[53 - 88]
m_HmenF_UR_24_4	-	m_HmenF_UR_24_4	-	1.1e-06	22.4	5.4	1.1e-06	22.4	5.4	1.0	1	0	0	1	1	1	1	[68 - 36] (REVERSE SENSE)
a_HmenF_UR_24_4	-	m_HmenF_UR_24_4	-	2.4e-06	21.4	5.3	2.4e-06	21.4	5.3	1.0	1	0	0	1	1	1	1	[68 - 36] (REVERSE SENSE)
m_HmenF_UR_24_5	-	m_HmenF_UR_24_5	-	8.6e-12	37.3	1.8	8.8e-12	37.3	1.8	1.0	1	0	0	1	1	1	1	[84 - 25] (REVERSE SENSE)
a_HmenF_UR_24_5	-	m_HmenF_UR_24_5	-	2.3e-11	36.1	1.7	2.3e-11	36.0	1.7	1.0	1	0	0	1	1	1	1	[84 - 25] (REVERSE SENSE)
m_HmenF_UR_24_6	-	m_HmenF_UR_24_6	-	2.3e-12	39.6	2.6	2.3e-12	39.6	2.6	1.0	1	0	0	1	1	1	1	[61 - 2] (REVERSE SENSE)
a_HmenF_UR_24_6	-	m_HmenF_UR_24_6	-	6.5e-12	38.3	2.6	6.6e-12	38.3	2.6	1.0	1	0	0	1	1	1	1	[61 - 2] (REVERSE SENSE)
m_HmenF_UR_25_1	-	m_HmenF_UR_25_1	-	9.7e-07	20.2	0.3	9.7e-07	20.2	0.3	1.0	1	0	0	1	1	1	1	[74 - 103]
a_HmenF_UR_25_1	-	m_HmenF_UR_25_1	-	3e-06	19.0	0.2	3e-06	19.0	0.2	1.0	1	0	0	1	1	1	1	[74 - 103]
a_HmenF_UR_25_2	-	m_HmenF_UR_25_2	-	9.8e-08	24.2	0.4	9.8e-08	24.2	0.4	1.0	1	0	0	1	1	1	1	[94 - 53] (REVERSE SENSE)
m_HmenF_UR_25_2	-	m_HmenF_UR_25_2	-	9.8e-08	24.2	0.4	9.8e-08	24.2	0.4	1.0	1	0	0	1	1	1	1	[94 - 53] (REVERSE SENSE)
a_HmenF_UR_25_3	-	m_HmenF_UR_25_3	-	1.1e-09	30.1	0.8	1.1e-09	30.1	0.8	1.0	1	0	0	1	1	1	1	[75 - 34] (REVERSE SENSE)
m_HmenF_UR_25_3	-	m_HmenF_UR_25_3	-	1.1e-09	30.1	0.8	1.1e-09	30.1	0.8	1.0	1	0	0	1	1	1	1	[75 - 34] (REVERSE SENSE)
a_HmenF_UR_26_1	-	m_HmenF_UR_26_1	-	1.4e-05	15.4	0.3	1.4e-05	15.4	0.3	1.0	1	0	0	1	1	1	1	[1 - 30]
m_HmenF_UR_26_1	-	m_HmenF_UR_26_1	-	1.4e-05	15.4	0.3	1.4e-05	15.4	0.3	1.0	1	0	0	1	1	1	1	[1 - 30]
m_HmenF_UR_26_2	-	m_HmenF_UR_26_2	-	2.5e-07	22.0	0.1	2.5e-07	22.0	0.1	1.0	1	0	0	1	1	1	1	[36 - 1] (REVERSE SENSE)
a_HmenF_UR_26_2	-	m_HmenF_UR_26_2	-	6.1e-07	21.0	0.1	6.1e-07	21.0	0.1	1.0	1	0	0	1	1	1	1	[36 - 1] (REVERSE SENSE)
a_CmonF_UR_1_1	-	m_CmonF_UR_1_1	-	6.6e-06	17.4	0.5	6.6e-06	17.4	0.5	1.0	1	0	0	1	1	1	1	[31 - 2] (REVERSE SENSE)
m_CmonF_UR_1_1	-	m_CmonF_UR_1_1	-	6.6e-06	17.4	0.5	6.6e-06	17.4	0.5	1.0	1	0	0	1	1	1	1	[31 - 2] (REVERSE SENSE)
m_CmonF_UR_3_1	-	m_CmonF_UR_3_1	-	2.8e-06	19.6	0.4	2.8e-06	19.6	0.4	1.0	1	0	0	1	1	1	1	[31 - 2] (REVERSE SENSE)
a_CmonF_UR_3_1	-	m_CmonF_UR_3_1	-	6.9e-06	18.6	0.3	6.9e-06	18.6	0.3	1.0	1	0	0	1	1	1	1	[31 - 2] (REVERSE SENSE)
m_CmonF_UR_4_1	-	m_CmonF_UR_4_1	-	3.3e-13	41.3	1.2	3.4e-13	41.3	1.2	1.0	1	0	0	1	1	1	1	[32 - 91]
a_CmonF_UR_4_1	-	m_CmonF_UR_4_1	-	7.9e-13	40.2	0.9	8e-13	40.2	0.9	1.0	1	0	0	1	1	1	1	[32 - 91]
m_CmonF_UR_4_2	-	m_CmonF_UR_4_2	-	2.7e-15	46.8	0.9	2.8e-15	46.8	0.9	1.0	1	0	0	1	1	1	1	[61 - 138]
a_CmonF_UR_4_2	-	m_CmonF_UR_4_2	-	6.5e-15	45.8	0.7	6.7e-15	45.7	0.7	1.0	1	0	0	1	1	1	1	[61 - 138]
a_CmonF_UR_4_3	-	m_CmonF_UR_4_3	-	1.3e-05	15.3	1.4	1.3e-05	15.3	1.4	1.1	1	0	0	1	1	1	1	[154 - 183]
m_CmonF_UR_4_3	-	m_CmonF_UR_4_3	-	1.3e-05	15.3	1.4	1.3e-05	15.3	1.4	1.1	1	0	0	1	1	1	1	[154 - 183]
a_CmonF_UR_4_4	-	m_CmonF_UR_4_4	-	3.2e-15	48.1	6.6	3.4e-15	48.0	6.6	1.0	1	0	0	1	1	1	1	[138 - 215]
m_CmonF_UR_4_4	-	m_CmonF_UR_4_4	-	3.2e-15	48.1	6.6	3.4e-15	48.0	6.6	1.0	1	0	0	1	1	1	1	[138 - 215]
m_CmonF_UR_4_5	-	m_CmonF_UR_4_5	-	7.1e-08	25.3	5.1	7.1e-08	25.3	5.1	1.0	1	0	0	1	1	1	1	[162 - 124] (REVERSE SENSE)
a_CmonF_UR_4_5	-	m_CmonF_UR_4_5	-	1.6e-07	24.4	5.0	1.6e-07	24.4	5.0	1.0	1	0	0	1	1	1	1	[162 - 124] (REVERSE SENSE)
m_CmonF_UR_4_6	-	m_CmonF_UR_4_6	-	6.4e-14	44.6	5.0	6.7e-14	44.5	5.0	1.0	1	0	0	1	1	1	1	[196 - 116] (REVERSE SENSE)
a_CmonF_UR_4_6	-	m_CmonF_UR_4_6	-	1.8e-13	43.3	5.3	1.9e-13	43.2	5.3	1.0	1	0	0	1	1	1	1	[196 - 116] (REVERSE SENSE)
m_CmonF_UR_4_7	-	m_CmonF_UR_4_7	-	1e-08	26.8	0.8	1e-08	26.8	0.8	1.0	1	0	0	1	1	1	1	[108 - 70] (REVERSE SENSE)
a_CmonF_UR_4_7	-	m_CmonF_UR_4_7	-	2.3e-08	25.9	0.8	2.3e-08	25.9	0.8	1.0	1	0	0	1	1	1	1	[108 - 70] (REVERSE SENSE)
m_CmonF_UR_4_8	-	m_CmonF_UR_4_8	-	5.5e-37	120.4	13.4	6e-37	120.3	13.4	1.0	1	0	0	1	1	1	1	[179 - 3] (REVERSE SENSE)
a_CmonF_UR_4_8	-	m_CmonF_UR_4_8	-	1.3e-36	119.2	13.4	1.4e-36	119.1	13.4	1.0	1	0	0	1	1	1	1	[179 - 3] (REVERSE SENSE)
m_CmonF_UR_4_9	-	m_CmonF_UR_4_9	-	2.1e-15	49.5	6.1	2.1e-15	49.5	6.1	1.0	1	0	0	1	1	1	1	[73 - 2] (REVERSE SENSE)
a_CmonF_UR_4_9	-	m_CmonF_UR_4_9	-	4.4e-15	48.5	5.8	4.5e-15	48.5	5.8	1.0	1	0	0	1	1	1	1	[73 - 2] (REVERSE SENSE)
m_CmonF_UR_4_10	-	m_CmonF_UR_4_10	-	1.3e-06	20.2	0.1	1.3e-06	20.2	0.1	1.0	1	0	0	1	1	1	1	[33 - 1] (REVERSE SENSE)
a_CmonF_UR_4_10	-	m_CmonF_UR_4_10	-	4.1e-06	18.9	0.1	4.1e-06	18.9	0.1	1.0	1	0	0	1	1	1	1	[33 - 1] (REVERSE SENSE)
m_CmonF_UR_7_1	-	m_CmonF_UR_7_1	-	3.7e-08	24.7	0.3	3.7e-08	24.7	0.3	1.0	1	0	0	1	1	1	1	[6 - 44]
a_CmonF_UR_7_1	-	m_CmonF_UR_7_1	-	1.1e-07	23.5	0.2	1.1e-07	23.5	0.2	1.0	1	0	0	1	1	1	1	[6 - 44]
a_CmonF_UR_7_2	-	m_CmonF_UR_7_2	-	6.2e-10	30.0	0.8	6.2e-10	30.0	0.8	1.0	1	0	0	1	1	1	1	[1 - 45]
m_CmonF_UR_7_2	-	m_CmonF_UR_7_2	-	6.2e-10	30.0	0.8	6.2e-10	30.0	0.8	1.0	1	0	0	1	1	1	1	[1 - 45]
m_CmonF_UR_7_3	-	m_CmonF_UR_7_3	-	8.3e-08	25.0	3.8	8.3e-08	25.0	3.8	1.0	1	0	0	1	1	1	1	[38 - 3] (REVERSE SENSE)
a_CmonF_UR_7_3	-	m_CmonF_UR_7_3	-	2e-07	24.0	3.8	2e-07	24.0	3.8	1.0	1	0	0	1	1	1	1	[38 - 3] (REVERSE SENSE)
m_CmonF_UR_8_1	-	m_CmonF_UR_8_1	-	2.7e-06	19.5	0.5	2.7e-06	19.5	0.5	1.0	1	0	0	1	1	1	1	[7 - 36]
a_CmonF_UR_8_1	-	m_CmonF_UR_8_1	-	8.2e-06	18.2	0.4	8.2e-06	18.2	0.4	1.0	1	0	0	1	1	1	1	[7 - 36]
m_CmonF_UR_8_2	-	m_CmonF_UR_8_2	-	1.6e-06	20.3	0.1	1.6e-06	20.3	0.1	1.0	1	0	0	1	1	1	1	[33 - 1] (REVERSE SENSE)
a_CmonF_UR_8_2	-	m_CmonF_UR_8_2	-	4.9e-06	19.0	0.1	4.9e-06	19.0	0.1	1.0	1	0	0	1	1	1	1	[33 - 1] (REVERSE SENSE)
m_CmonF_UR_10_1	-	m_CmonF_UR_10_1	-	1e-08	26.2	0.1	1e-08	26.2	0.1	1.0	1	0	0	1	1	1	1	[24 - 62]
a_CmonF_UR_10_1	-	m_CmonF_UR_10_1	-	3.1e-08	24.9	0.1	3.1e-08	24.9	0.1	1.0	1	0	0	1	1	1	1	[24 - 62]
a_CmonF_UR_10_2	-	m_CmonF_UR_10_2	-	2e-08	26.3	1.6	2e-08	26.3	1.6	1.0	1	0	0	1	1	1	1	[43 - 2] (REVERSE SENSE)
m_CmonF_UR_10_2	-	m_CmonF_UR_10_2	-	2e-08	26.3	1.6	2e-08	26.3	1.6	1.0	1	0	0	1	1	1	1	[43 - 2] (REVERSE SENSE)
a_CmonF_UR_17_1	-	m_CmonF_UR_17_1	-	1.2e-14	45.2	0.5	1.3e-14	45.2	0.5	1.0	1	0	0	1	1	1	1	[9 - 71]
m_CmonF_UR_17_1	-	m_CmonF_UR_17_1	-	1.2e-14	45.2	0.5	1.3e-14	45.2	0.5	1.0	1	0	0	1	1	1	1	[9 - 71]
m_CmonF_UR_17_2	-	m_CmonF_UR_17_2	-	7e-12	36.5	0.4	7.1e-12	36.5	0.4	1.0	1	0	0	1	1	1	1	[19 - 75]
a_CmonF_UR_17_2	-	m_CmonF_UR_17_2	-	1.9e-11	35.3	0.3	1.9e-11	35.3	0.3	1.0	1	0	0	1	1	1	1	[19 - 75]
m_CmonF_UR_17_3	-	m_CmonF_UR_17_3	-	2e-15	49.7	5.7	2.1e-15	49.7	5.7	1.0	1	0	0	1	1	1	1	[71 - 3] (REVERSE SENSE)
a_CmonF_UR_17_3	-	m_CmonF_UR_17_3	-	3.5e-15	49.0	5.2	3.6e-15	49.0	5.2	1.0	1	0	0	1	1	1	1	[71 - 3] (REVERSE SENSE)
m_CmonF_UR_17_4	-	m_CmonF_UR_17_4	-	8.9e-15	47.6	5.0	9.2e-15	47.5	5.0	1.0	1	0	0	1	1	1	1	[75 - 1] (REVERSE SENSE)
a_CmonF_UR_17_4	-	m_CmonF_UR_17_4	-	1.7e-14	46.8	4.6	1.7e-14	46.7	4.6	1.0	1	0	0	1	1	1	1	[75 - 1] (REVERSE SENSE)
a_CmonF_UR_18_1	-	m_CmonF_UR_18_1	-	1.1e-07	23.4	1.1	1.1e-07	23.4	1.1	1.0	1	0	0	1	1	1	1	[2 - 37]
m_CmonF_UR_18_1	-	m_CmonF_UR_18_1	-	1.1e-07	23.4	1.1	1.1e-07	23.4	1.1	1.0	1	0	0	1	1	1	1	[2 - 37]
a_CmonF_UR_18_2	-	m_CmonF_UR_18_2	-	2.1e-06	20.4	0.9	2.1e-06	20.4	0.9	1.0	1	0	0	1	1	1	1	[38 - 3] (REVERSE SENSE)
m_CmonF_UR_18_2	-	m_CmonF_UR_18_2	-	2.1e-06	20.4	0.9	2.1e-06	20.4	0.9	1.0	1	0	0	1	1	1	1	[38 - 3] (REVERSE SENSE)
a_CmonF_UR_19_1	-	m_CmonF_UR_19_1	-	4.9e-07	20.7	0.5	4.9e-07	20.7	0.5	1.0	1	0	0	1	1	1	1	[1 - 30]
m_CmonF_UR_19_1	-	m_CmonF_UR_19_1	-	4.9e-07	20.7	0.5	4.9e-07	20.7	0.5	1.0	1	0	0	1	1	1	1	[1 - 30]
m_CmonF_UR_19_2	-	m_CmonF_UR_19_2	-	5.2e-08	24.6	1.5	5.2e-08	24.6	1.5	1.0	1	0	0	1	1	1	1	[14 - 52]
a_CmonF_UR_19_2	-	m_CmonF_UR_19_2	-	1.8e-07	23.2	1.6	1.8e-07	23.2	1.6	1.0	1	0	0	1	1	1	1	[14 - 52]
m_CmonF_UR_19_3	-	m_CmonF_UR_19_3	-	2.5e-06	17.6	0.2	2.5e-06	17.6	0.2	1.0	1	0	0	1	1	1	1	[21 - 53]
a_CmonF_UR_19_3	-	m_CmonF_UR_19_3	-	8.5e-06	16.4	0.1	8.5e-06	16.4	0.1	1.0	1	0	0	1	1	1	1	[21 - 53]
m_CmonF_UR_19_4	-	m_CmonF_UR_19_4	-	4.7e-07	21.5	0.3	4.7e-07	21.5	0.3	1.0	1	0	0	1	1	1	1	[35 - 3] (REVERSE SENSE)
a_CmonF_UR_19_4	-	m_CmonF_UR_19_4	-	1.5e-06	20.2	0.3	1.5e-06	20.2	0.3	1.0	1	0	0	1	1	1	1	[35 - 3] (REVERSE SENSE)
a_CmonF_UR_19_5	-	m_CmonF_UR_19_5	-	1.4e-08	26.4	0.7	1.4e-08	26.4	0.7	1.0	1	0	0	1	1	1	1	[45 - 1] (REVERSE SENSE)
m_CmonF_UR_19_5	-	m_CmonF_UR_19_5	-	1.4e-08	26.4	0.7	1.4e-08	26.4	0.7	1.0	1	0	0	1	1	1	1	[45 - 1] (REVERSE SENSE)
a_CmonF_UR_20_1	-	m_CmonF_UR_20_1	-	5.4e-08	23.2	0.2	5.4e-08	23.2	0.2	1.0	1	0	0	1	1	1	1	[5 - 43]
m_CmonF_UR_20_1	-	m_CmonF_UR_20_1	-	5.4e-08	23.2	0.2	5.4e-08	23.2	0.2	1.0	1	0	0	1	1	1	1	[5 - 43]
m_CmonF_UR_20_2	-	m_CmonF_UR_20_2	-	2.2e-07	21.6	0.2	2.2e-07	21.6	0.2	1.0	1	0	0	1	1	1	1	[21 - 56]
a_CmonF_UR_20_2	-	m_CmonF_UR_20_2	-	5.4e-07	20.6	0.2	5.4e-07	20.6	0.2	1.0	1	0	0	1	1	1	1	[21 - 56]
m_CmonF_UR_20_3	-	m_CmonF_UR_20_3	-	6.7e-09	26.8	0.9	6.7e-09	26.8	0.9	1.0	1	0	0	1	1	1	1	[62 - 21] (REVERSE SENSE)
a_CmonF_UR_20_3	-	m_CmonF_UR_20_3	-	1.7e-08	25.7	0.7	1.7e-08	25.7	0.7	1.0	1	0	0	1	1	1	1	[62 - 21] (REVERSE SENSE)
a_CmonF_UR_20_4	-	m_CmonF_UR_20_4	-	6e-10	30.6	0.9	6.1e-10	30.6	0.9	1.0	1	0	0	1	1	1	1	[49 - 2] (REVERSE SENSE)
m_CmonF_UR_20_4	-	m_CmonF_UR_20_4	-	6e-10	30.6	0.9	6.1e-10	30.6	0.9	1.0	1	0	0	1	1	1	1	[49 - 2] (REVERSE SENSE)
m_CmonF_UR_20_5	-	m_CmonF_UR_20_5	-	1.4e-08	26.1	0.6	1.4e-08	26.1	0.6	1.0	1	0	0	1	1	1	1	[42 - 1] (REVERSE SENSE)
a_CmonF_UR_20_5	-	m_CmonF_UR_20_5	-	3.1e-08	25.2	0.5	3.1e-08	25.2	0.5	1.0	1	0	0	1	1	1	1	[42 - 1] (REVERSE SENSE)
m_CmonF_UR_25_1	-	m_CmonF_UR_25_1	-	2.9e-08	25.1	0.3	2.9e-08	25.1	0.3	1.0	1	0	0	1	1	1	1	[2 - 46]
a_CmonF_UR_25_1	-	m_CmonF_UR_25_1	-	8.7e-08	23.8	0.2	8.7e-08	23.8	0.2	1.0	1	0	0	1	1	1	1	[2 - 46]
m_CmonF_UR_25_2	-	m_CmonF_UR_25_2	-	1.2e-08	27.8	5.3	1.2e-08	27.8	5.3	1.0	1	0	0	1	1	1	1	[44 - 3] (REVERSE SENSE)
a_CmonF_UR_25_2	-	m_CmonF_UR_25_2	-	1.7e-08	27.4	4.5	1.7e-08	27.4	4.5	1.0	1	0	0	1	1	1	1	[44 - 3] (REVERSE SENSE)
a_CmonF_UR_26_1	-	m_CmonF_UR_26_1	-	7.1e-07	19.5	0.2	7.1e-07	19.5	0.2	1.0	1	0	0	1	1	1	1	[1 - 33]
m_CmonF_UR_26_1	-	m_CmonF_UR_26_1	-	7.1e-07	19.5	0.2	7.1e-07	19.5	0.2	1.0	1	0	0	1	1	1	1	[1 - 33]
m_CmonF_UR_28_1	-	m_CmonF_UR_28_1	-	5.7e-08	25.4	1.0	5.7e-08	25.4	1.0	1.0	1	0	0	1	1	1	1	[51 - 13] (REVERSE SENSE)
a_CmonF_UR_28_1	-	m_CmonF_UR_28_1	-	1.6e-07	24.2	0.8	1.6e-07	24.2	0.8	1.0	1	0	0	1	1	1	1	[51 - 13] (REVERSE SENSE)
m_CmonF_UR_28_2	-	m_CmonF_UR_28_2	-	1.1e-07	24.5	2.3	1.1e-07	24.5	2.3	1.0	1	0	0	1	1	1	1	[38 - 3] (REVERSE SENSE)
a_CmonF_UR_28_2	-	m_CmonF_UR_28_2	-	2.4e-07	23.7	2.1	2.4e-07	23.7	2.1	1.0	1	0	0	1	1	1	1	[38 - 3] (REVERSE SENSE)
a_CmonF_UR_29_1	-	m_CmonF_UR_29_1	-	1.9e-06	21.6	7.0	1.9e-06	21.6	7.0	1.0	1	0	0	1	1	1	1	[46 - 17] (REVERSE SENSE)
m_CmonF_UR_29_1	-	m_CmonF_UR_29_1	-	1.9e-06	21.6	7.0	1.9e-06	21.6	7.0	1.0	1	0	0	1	1	1	1	[46 - 17] (REVERSE SENSE)
a_HmenM_UR_2_1	-	m_HmenM_UR_2_1	-	3.9e-06	19.2	0.8	3.9e-06	19.2	0.8	1.0	1	0	0	1	1	1	1	[30 - 59]
m_HmenM_UR_2_1	-	m_HmenM_UR_2_1	-	3.9e-06	19.2	0.8	3.9e-06	19.2	0.8	1.0	1	0	0	1	1	1	1	[30 - 59]
a_HmenM_UR_2_2	-	m_HmenM_UR_2_2	-	5.2e-05	14.9	0.1	5.2e-05	14.9	0.1	1.0	1	0	0	1	1	1	1	[60 - 31] (REVERSE SENSE)
m_HmenM_UR_2_2	-	m_HmenM_UR_2_2	-	5.2e-05	14.9	0.1	5.2e-05	14.9	0.1	1.0	1	0	0	1	1	1	1	[60 - 31] (REVERSE SENSE)
m_HmenM_UR_2_3	-	m_HmenM_UR_2_3	-	1.1e-08	27.7	3.5	1.1e-08	27.7	3.5	1.0	1	0	0	1	1	1	1	[49 - 2] (REVERSE SENSE)
a_HmenM_UR_2_3	-	m_HmenM_UR_2_3	-	2.4e-08	26.8	3.1	2.4e-08	26.8	3.1	1.0	1	0	0	1	1	1	1	[49 - 2] (REVERSE SENSE)
m_HmenM_UR_3_1	-	m_HmenM_UR_3_1	-	4e-11	34.2	3.4	4.1e-11	34.1	3.4	1.0	1	0	0	1	1	1	1	[8 - 64]

a_HmenM_UR_3_1	-	m_HmenM_UR_3_1	-	7.8e-11	33.4	2.9	7.9e-11	33.4	2.9	1.0	1	0	0	1	1	1	1	[8 - 64]
m_HmenM_UR_3_2	-	m_HmenM_UR_3_2	-	2e-17	56.1	6.3	2.1e-17	56.1	6.3	1.0	1	0	0	1	1	1	1	[94 - 177]
a_HmenM_UR_3_2	-	m_HmenM_UR_3_2	-	4.5e-17	55.0	6.1	4.7e-17	55.0	6.1	1.0	1	0	0	1	1	1	1	[94 - 177]
m_HmenM_UR_3_3	-	m_HmenM_UR_3_3	-	2.9e-05	14.3	0.1	2.9e-05	14.3	0.1	1.0	1	0	0	1	1	1	1	[310 - 339]
a_HmenM_UR_3_3	-	m_HmenM_UR_3_3	-	0.00011	13.1	0.1	0.00011	13.1	0.1	1.0	1	0	0	1	1	1	1	[310 - 339]
a_HmenM_UR_3_4	-	m_HmenM_UR_3_4	-	4.6e-16	51.5	7.4	4.8e-16	51.5	7.4	1.0	1	0	0	1	1	1	1	[386 - 460]
m_HmenM_UR_3_4	-	m_HmenM_UR_3_4	-	4.6e-16	51.5	7.4	4.8e-16	51.5	7.4	1.0	1	0	0	1	1	1	1	[386 - 460]
m_HmenM_UR_3_5	-	m_HmenM_UR_3_5	-	3.5e-36	117.8	16.4	3.8e-36	117.6	16.4	1.0	1	0	0	1	1	1	1	[424 - 597]
a_HmenM_UR_3_5	-	m_HmenM_UR_3_5	-	7.1e-36	116.8	16.1	7.7e-36	116.7	16.1	1.0	1	0	0	1	1	1	1	[424 - 597]
a_HmenM_UR_17_2	-	m_HmenM_UR_3_5	-	1.7e-07	27.3	10.5	1.7e-07	27.3	10.5	1.0	1	0	0	1	1	1	1	[64 - 132]
m_HmenM_UR_17_2	-	m_HmenM_UR_3_5	-	3.5e-07	26.3	10.5	3.5e-07	26.3	10.5	1.0	1	0	0	1	1	1	1	[64 - 132]
m_HmenM_UR_3_6	-	m_HmenM_UR_3_6	-	6.3e-23	73.1	0.8	6.7e-23	73.0	0.8	1.0	1	0	0	1	1	1	1	[506 - 610]
a_HmenM_UR_3_6	-	m_HmenM_UR_3_6	-	1.6e-22	71.8	0.8	1.7e-22	71.7	0.8	1.0	1	0	0	1	1	1	1	[506 - 610]
m_HmenM_UR_17_4	-	m_HmenM_UR_3_6	-	4.7e-13	43.3	0.1	5.4e-13	43.1	0.1	1.1	1	0	0	1	1	1	1	[44 - 172]
a_HmenM_UR_17_4	-	m_HmenM_UR_3_6	-	5.4e-13	43.1	0.1	6.2e-13	42.9	0.1	1.1	1	0	0	1	1	1	1	[44 - 172]
m_HmenM_UR_3_7	-	m_HmenM_UR_3_7	-	1.6e-127	417.9	17.1	1.8e-127	417.8	17.1	1.0	1	0	0	1	1	1	1	[75 - 638]
a_HmenM_UR_3_7	-	m_HmenM_UR_3_7	-	3.2e-127	417.0	16.9	3.5e-127	416.8	16.9	1.0	1	0	0	1	1	1	1	[75 - 638]
a_HmenM_UR_17_3	-	m_HmenM_UR_3_7	-	1.2e-09	33.5	1.2	1.2e-09	33.5	1.2	1.0	1	0	0	1	1	1	1	[48 - 137]
m_HmenM_UR_17_3	-	m_HmenM_UR_3_7	-	1.2e-09	33.5	1.2	1.2e-09	33.5	1.2	1.0	1	0	0	1	1	1	1	[48 - 137]
m_HmenM_UR_3_8	-	m_HmenM_UR_3_8	-	3.3e-11	34.6	0.7	3.3e-11	34.6	0.7	1.0	1	0	0	1	1	1	1	[613 - 666]
a_HmenM_UR_3_8	-	m_HmenM_UR_3_8	-	8.5e-11	33.4	0.5	8.5e-11	33.4	0.5	1.0	1	0	0	1	1	1	1	[613 - 666]
a_HmenM_UR_3_9	-	m_HmenM_UR_3_9	-	1.2e-06	19.7	0.4	1.2e-06	19.7	0.4	1.0	1	0	0	1	1	1	1	[638 - 667]
m_HmenM_UR_3_9	-	m_HmenM_UR_3_9	-	1.2e-06	19.7	0.4	1.2e-06	19.7	0.4	1.0	1	0	0	1	1	1	1	[638 - 667]
m_HmenM_UR_3_10	-	m_HmenM_UR_3_10	-	1.2e-07	24.7	3.4	1.2e-07	24.7	3.4	1.0	1	0	0	1	1	1	1	[656 - 621] (REVERSE SENSE)
a_HmenM_UR_3_10	-	m_HmenM_UR_3_10	-	2.9e-07	23.6	3.5	2.9e-07	23.6	3.5	1.0	1	0	0	1	1	1	1	[656 - 621] (REVERSE SENSE)
m_HmenM_UR_3_11	-	m_HmenM_UR_3_11	-	5.2e-06	18.7	0.5	5.2e-06	18.7	0.5	1.0	1	0	0	1	1	1	1	[645 - 613] (REVERSE SENSE)
a_HmenM_UR_3_11	-	m_HmenM_UR_3_11	-	1.3e-05	17.7	0.5	1.3e-05	17.7	0.5	1.0	1	0	0	1	1	1	1	[645 - 613] (REVERSE SENSE)
m_HmenM_UR_3_12	-	m_HmenM_UR_3_12	-	6.6e-12	36.8	1.1	6.7e-12	36.8	1.1	1.0	1	0	0	1	1	1	1	[652 - 596] (REVERSE SENSE)
a_HmenM_UR_3_12	-	m_HmenM_UR_3_12	-	1.9e-11	35.5	1.1	1.9e-11	35.5	1.1	1.0	1	0	0	1	1	1	1	[652 - 596] (REVERSE SENSE)
a_HmenM_UR_3_13	-	m_HmenM_UR_3_13	-	3.3e-14	46.0	10.7	3.4e-14	45.9	10.7	1.0	1	0	0	1	1	1	1	[609 - 541] (REVERSE SENSE)
m_HmenM_UR_3_13	-	m_HmenM_UR_3_13	-	3.3e-14	46.0	10.7	3.4e-14	45.9	10.7	1.0	1	0	0	1	1	1	1	[609 - 541] (REVERSE SENSE)
m_HmenM_UR_3_14	-	m_HmenM_UR_3_14	-	5.4e-27	86.6	0.2	5.8e-27	86.5	0.2	1.0	1	0	0	1	1	1	1	[586 - 458] (REVERSE SENSE)
a_HmenM_UR_3_14	-	m_HmenM_UR_3_14	-	1.4e-26	85.4	0.2	1.5e-26	85.3	0.2	1.0	1	0	0	1	1	1	1	[586 - 458] (REVERSE SENSE)
a_HmenM_UR_17_12	-	m_HmenM_UR_3_14	-	1.5e-05	20.9	0.0	1.5e-05	20.9	0.0	1.1	1	0	0	1	1	1	1	[88 - 29] (REVERSE SENSE)
m_HmenM_UR_17_12	-	m_HmenM_UR_3_14	-	3e-05	20.0	0.0	3e-05	20.0	0.0	1.1	1	0	0	1	1	1	1	[88 - 29] (REVERSE SENSE)
m_HmenM_UR_3_15	-	m_HmenM_UR_3_15	-	4.5e-07	23.4	3.0	4.5e-07	23.4	3.0	1.0	1	0	0	1	1	1	1	[450 - 412] (REVERSE SENSE)
a_HmenM_UR_3_15	-	m_HmenM_UR_3_15	-	1e-06	22.4	2.7	1e-06	22.4	2.7	1.0	1	0	0	1	1	1	1	[450 - 412] (REVERSE SENSE)
m_HmenM_UR_3_16	-	m_HmenM_UR_3_16	-	9e-27	86.7	5.8	9.7e-27	86.6	5.8	1.0	1	0	0	1	1	1	1	[530 - 408] (REVERSE SENSE)
a_HmenM_UR_3_16	-	m_HmenM_UR_3_16	-	2.4e-26	85.4	5.8	2.5e-26	85.3	5.8	1.0	1	0	0	1	1	1	1	[530 - 408] (REVERSE SENSE)
m_HmenM_UR_3_17	-	m_HmenM_UR_3_17	-	4e-05	15.1	0.4	4e-05	15.1	0.4	1.0	1	0	0	1	1	1	1	[349 - 317] (REVERSE SENSE)
a_HmenM_UR_3_17	-	m_HmenM_UR_3_17	-	0.00011	14.1	0.4	0.00011	14.1	0.4	1.0	1	0	0	1	1	1	1	[349 - 317] (REVERSE SENSE)
m_HmenM_UR_3_18	-	m_HmenM_UR_3_18	-	2.7e-06	18.6	0.3	2.7e-06	18.6	0.3	1.0	1	0	0	1	1	1	1	[329 - 294] (REVERSE SENSE)
a_HmenM_UR_3_18	-	m_HmenM_UR_3_18	-	8.5e-06	17.4	0.2	8.5e-06	17.4	0.2	1.0	1	0	0	1	1	1	1	[329 - 294] (REVERSE SENSE)
a_HmenM_UR_3_19	-	m_HmenM_UR_3_19	-	1.8e-13	42.2	2.0	1.8e-13	42.2	2.0	1.0	1	0	0	1	1	1	1	[214 - 143] (REVERSE SENSE)
m_HmenM_UR_3_19	-	m_HmenM_UR_3_19	-	1.8e-13	42.2	2.0	1.8e-13	42.2	2.0	1.0	1	0	0	1	1	1	1	[214 - 143] (REVERSE SENSE)
m_HmenM_UR_3_20	-	m_HmenM_UR_3_20	-	3.8e-37	119.9	1.1	4.1e-37	119.8	1.1	1.0	1	0	0	1	1	1	1	[284 - 123] (REVERSE SENSE)
a_HmenM_UR_3_20	-	m_HmenM_UR_3_20	-	8.4e-37	118.9	1.0	9e-37	118.8	1.0	1.0	1	0	0	1	1	1	1	[284 - 123] (REVERSE SENSE)
a_HmenM_UR_3_21	-	m_HmenM_UR_3_21	-	2.8e-06	20.7	3.9	2.8e-06	20.7	3.9	1.0	1	0	0	1	1	1	1	[133 - 98] (REVERSE SENSE)
m_HmenM_UR_3_21	-	m_HmenM_UR_3_21	-	2.8e-06	20.7	3.9	2.8e-06	20.7	3.9	1.0	1	0	0	1	1	1	1	[133 - 98] (REVERSE SENSE)
m_HmenM_UR_3_22	-	m_HmenM_UR_3_22	-	4.1e-13	42.1	4.3	4.3e-13	42.0	4.3	1.0	1	0	0	1	1	1	1	[95 - 27] (REVERSE SENSE)
a_HmenM_UR_3_22	-	m_HmenM_UR_3_22	-	1.3e-12	40.6	4.5	1.4e-12	40.6	4.5	1.0	1	0	0	1	1	1	1	[95 - 27] (REVERSE SENSE)
m_HmenM_UR_3_23	-	m_HmenM_UR_3_23	-	3.3e-18	58.9	12.4	3.5e-18	58.9	12.4	1.0	1	0	0	1	1	1	1	[94 - 2] (REVERSE SENSE)
a_HmenM_UR_3_23	-	m_HmenM_UR_3_23	-	6.8e-18	58.0	12.4	7.2e-18	57.9	12.4	1.0	1	0	0	1	1	1	1	[94 - 2] (REVERSE SENSE)
m_HmenM_UR_3_24	-	m_HmenM_UR_3_24	-	1.1e-05	18.0	2.5	1.1e-05	18.0	2.5	1.0	1	0	0	1	1	1	1	[39 - 1] (REVERSE SENSE)
a_HmenM_UR_3_24	-	m_HmenM_UR_3_24	-	2.5e-05	17.0	2.5	2.5e-05	17.0	2.5	1.0	1	0	0	1	1	1	1	[39 - 1] (REVERSE SENSE)
m_HmenM_UR_4_1	-	m_HmenM_UR_4_1	-	2.7e-06	19.0	0.2	2.7e-06	19.0	0.2	1.0	1	0	0	1	1	1	1	[38 - 3] (REVERSE SENSE)
a_HmenM_UR_4_1	-	m_HmenM_UR_4_1	-	6.8e-06	18.0	0.1	6.8e-06	18.0	0.1	1.0	1	0	0	1	1	1	1	[38 - 3] (REVERSE SENSE)
m_HmenM_UR_6_1	-	m_HmenM_UR_6_1	-	4.6e-06	18.8	1.4	4.6e-06	18.8	1.4	1.0	1	0	0	1	1	1	1	[31 - 2] (REVERSE SENSE)
a_HmenM_UR_6_1	-	m_HmenM_UR_6_1	-	1.9e-05	17.2	1.6	1.9e-05	17.2	1.6	1.1	1	0	0	1	1	1	1	[31 - 2] (REVERSE SENSE)
a_HmenM_UR_14_1	-	m_HmenM_UR_14_1	-	5e-12	37.1	2.5	5.1e-12	37.1	2.5	1.0	1	0	0	1	1	1	1	[12 - 68]
m_HmenM_UR_14_1	-	m_HmenM_UR_14_1	-	5e-12	37.1	2.5	5.1e-12	37.1	2.5	1.0	1	0	0	1	1	1	1	[12 - 68]
m_HmenM_UR_14_2	-	m_HmenM_UR_14_2	-	3.6e-06	18.4	0.3	3.6e-06	18.4	0.3	1.0	1	0	0	1	1	1	1	[63 - 31] (REVERSE SENSE)
a_HmenM_UR_14_2	-	m_HmenM_UR_14_2	-	1.1e-05	17.2	0.3	1.1e-05	17.2	0.3	1.1	1	0	0	1	1	1	1	[63 - 31] (REVERSE SENSE)
m_HmenM_UR_14_3	-	m_HmenM_UR_14_3	-	2.8e-15	48.9	4.3	2.8e-15	48.9	4.3	1.0	1	0	0	1	1	1	1	[65 - 3] (REVERSE SENSE)
a_HmenM_UR_14_3	-	m_HmenM_UR_14_3	-	5.2e-15	48.1	4.1	5.3e-15	48.1	4.1	1.0	1	0	0	1	1	1	1	[65 - 3] (REVERSE SENSE)
a_HmenM_UR_17_1	-	m_HmenM_UR_17_1	-	2.5e-07	21.5	0.4	2.5e-07	21.5	0.4	1.0	1	0	0	1	1	1	1	[10 - 45]
m_HmenM_UR_17_1	-	m_HmenM_UR_17_1	-	2.5e-07	21.5	0.4	2.5e-07	21.5	0.4	1.0	1	0	0	1	1	1	1	[10 - 45]
m_HmenM_UR_17_2	-	m_HmenM_UR_17_2	-	7.3e-13	42.4	11.9	7.6e-13	42.3	11.9	1.0	1	0	0	1	1	1	1	[64 - 132]
a_HmenM_UR_17_2	-	m_HmenM_UR_17_2	-	1.6e-12	41.4	11.6	1.7e-12	41.3	11.6	1.0	1	0	0	1	1	1	1	[64 - 132]
a_HmenM_UR_3_5	-	m_HmenM_UR_17_2	-	7.5e-08	27.5	8.3	7.5e-08	27.5	8.3	2.6	2	1	0	2	2	2	1	[424 - 597]
m_HmenM_UR_3_5	-	m_HmenM_UR_17_2	-	7.5e-08	27.5	8.3	7.5e-08	27.5	8.3	2.6	2	1	0	2	2	2	1	[424 - 597]
a_HmenM_UR_17_3	-	m_HmenM_UR_17_3	-	3.8e-18	57.4	2.7	3.9e-18	57.4	2.7	1.0	1	0	0	1	1	1	1	[48 - 137]
m_HmenM_UR_17_3	-	m_HmenM_UR_17_3	-	3.8e-18	57.4	2.7	3.9e-18	57.4	2.7	1.0	1	0	0	1	1	1	1	[48 - 137]
a_HmenM_UR_3_7	-	m_HmenM_UR_17_3	-	5.1e-10	33.5	0.6	5.1e-10	33.5	0.6	2.9	3	0	0	3	3	3	1	[75 - 638]
m_HmenM_UR_3_7	-	m_HmenM_UR_17_3	-	5.1e-10	33.5	0.6	5.1e-10	33.5	0.6	2.9	3	0	0	3	3	3	1	[75 - 638]
m_HmenM_UR_17_4	-	m_HmenM_UR_17_4	-	5.4e-29	92.5	1.0	5.9e-29	92.4	1.0	1.0	1	0	0	1	1	1	1	[44 - 172]
a_HmenM_UR_17_4	-	m_HmenM_UR_17_4	-	1.3e-28	91.3	0.9	1.4e-28	91.2	0.9	1.0	1	0	0	1	1	1	1	[44 - 172]
m_HmenM_UR_3_6	-	m_HmenM_UR_17_4	-	5.2e-13	43.6	0.2	5.4e-13	43.5	0.2	1.0	1	0	0	1	1	1	1	[506 - 610]
a_HmenM_UR_3_6	-	m_HmenM_UR_17_4	-	5.4e-13	43.5	0.2	5.4e-13	43.5	0.2	1.0	1	0	0	1	1	1	1	[506 - 610]
a_HmenM_UR_17_5	-	m_HmenM_UR_17_5	-	3.1e-06	18.1	1.0	3.1e-06	18.1	1.0	1.0	1	0	0	1	1	1	1	[190 - 222]
m_HmenM_UR_17_5	-	m_HmenM_UR_17_5	-	3.1e-06	18.1	1.0	3.1e-06	18.1	1.0	1.0	1	0	0	1	1	1	1	[190 - 222]
a_HmenM_UR_17_6	-	m_HmenM_UR_17_6	-	1.1e-09	29.5	2.9	1.1e-09	29.5	2.9	1.0	1	0	0	1	1	1	1	[225 - 272]
m_HmenM_UR_17_6	-	m_HmenM_UR_17_6	-	1.1e-09	29.5	2.9	1.1e-09	29.5	2.9	1.0	1	0	0	1	1	1	1	[225 - 272]
m_HmenM_UR_17_7	-	m_HmenM_UR_17_7	-	1.6e-07	23.8	4.1	1.6e-07	23.8	4.1	1.0	1	0	0	1	1	1	1	[266 - 225] (REVERSE SENSE)
a_HmenM_UR_17_7	-	m_HmenM_UR_17_7	-	5.7e-07	22.4	4.2	5.7e-07	22.4	4.2	1.0	1	0	0	1	1	1	1	[266 - 225] (REVERSE SENSE)
m_HmenM_UR_17_8	-	m_HmenM_UR_17_8	-	1.1e-09	31.0	3.9	1.1e-09	31.0	3.9	1.0	1	0	0	1	1	1	1	[268 - 221] (REVERSE SENSE)
a_HmenM_UR_17_8	-	m_HmenM_UR_17_8	-	4.1e-09	29.5	4.1	4.1e-09	29.5	4.1	1.0	1	0	0	1	1	1	1	[268 - 221] (REVERSE SENSE)
m_HmenM_UR_17_9	-	m_HmenM_UR_17_9	-	8.5e-12	37.8	3.8	8.8e-12	37.8	3.8	1.0	1	0	0	1	1	1	1	[237 - 175] (REVERSE SENSE)
a_HmenM_UR_17_9	-	m_HmenM_UR_17_9	-	2.6e-11	36.4	4.1	2.7e-11	36.4	4.1	1.0	1	0	0	1	1	1	1	[237 - 175] (REVERSE SENSE)
m_HmenM_UR_17_10	-	m_HmenM_UR_17_10	-	5.1e-13	42.1	7.8	5.3e-13	42.0	7.8	1.0	1	0	0	1	1	1	1	[215 - 150] (REVERSE SENSE)
a_HmenM_UR_17_10	-	m_HmenM_UR_17_10	-	1.1e-12	41.2	7.7	1.1e-12	41.1	7.7	1.0	1	0	0	1	1	1	1	[215 - 150] (REVERSE SENSE)
a_HmenM_UR_17_11	-	m_HmenM_UR_17_11	-	3.8e-15	49.7	10.5	4e-15	49.6	10.5	1.0	1	0	0	1	1	1	1	[128 - 48] (REVERSE SENSE)
m_HmenM_UR_17_11	-	m_HmenM_UR_17_11	-	3.8e-15	49.7	10.5	4e-15	49.6	10.5	1.0	1	0	0	1	1	1	1	[128 - 48] (REVERSE SENSE)
m_HmenM_UR_17_12	-	m_HmenM_UR_17_12	-	4.9e-12	37.9	0.1	5e-12	37.9	0.1	1.0	1	0	0	1	1	1	1	[88 - 29] (REVERSE SENSE)
a_HmenM_UR_17_12	-	m_HmenM_UR_17_12	-	1.4e-11	36.6	0.1	1.4e-11	36.6	0.1	1.0	1	0	0	1	1	1	1	[88 - 29] (REVERSE SENSE)
m_HmenM_UR_3_14	-	m_HmenM_UR_17_12	-	8.8e-06	20.2	0.1	1.5e-05	19.6	0.1	1.6	1	1	0	1	1	1	1	[586 - 458] (REVERSE SENSE)
a_HmenM_UR_3_14	-	m_HmenM_UR_17_12	-	8.9e-06	20.2	0.1	1.5e-05	19.6	0.1	1.6	1	1	0	1	1	1	1	[586 - 458] (REVERSE SENSE)
m_HmenM_UR_17_13	-	m_HmenM_UR_17_13	-	1.3e-08	27.1	0.4	1.3e-08	27.1	0.4	1.0	1	0	0	1	1	1	1	[41 - 3] (REVERSE SENSE)
a_HmenM_UR_17_13	-	m_HmenM_UR_17_13	-	4.1e-08	25.8	0.4	4.1e-08	25.8	0.4	1.0	1	0	0	1	1	1	1	[41 - 3] (REVERSE SENSE)
a_HmenM_UR_17_14	-	m_HmenM_UR_17_14	-	4.6e-21	68.6	9.7	4.9e-21	68.5	9.7	1.0	1	0	0	1	1	1	1	[102 - 1] (REVERSE SENSE)
m_HmenM_UR_17_14	-	m_HmenM_UR_17_14	-	4.6e-21	68.6	9.7	4.9e-21	68.5	9.7	1.0	1	0	0	1	1	1	1	[102 - 1] (REVERSE SENSE)
m_HmenM_UR_20_1	-	m_HmenM_UR_20_1	-	9.8e-09	27.2	0.5	9.8e-09	27.2	0.5	1.0	1	0	0	1	1	1	1	[50 - 3] (REVERSE SENSE)
a_HmenM_UR_20_1	-	m_HmenM_UR_20_1	-	2.2e-08	26.2	0.5	2.2e-08	26.2	0.5	1.0	1	0	0	1	1	1	1	[50 - 3] (REVERSE SENSE)
a_HmenM_UR_21_1	-	m_HmenM_UR_21_1	-	7.1e-06	16.5	0.6	7.1e-06	16.5	0.6	1.0	1	0	0	1	1	1	1	[1 - 30]
m_HmenM_UR_21_1	-	m_HmenM_UR_21_1	-	7.1e-06	16.5	0.6	7.1e-06	16.5	0.6	1.0	1	0	0	1	1	1	1	[1 - 30]
m_HmenM_UR_21_2	-	m_HmenM_UR_21_2	-	8.3e-10	29.9	0.6	8.3e-10	29.9	0.6	1.0	1	0	0	1	1	1	1	[52 - 96]
a_HmenM_UR_21_2	-	m_HmenM_UR_21_2	-	2.2e-09	28.7	0.5	2.2e-09	28.7	0.5	1.0	1	0	0	1	1	1	1	[52 - 96]
a_HmenM_UR_21_3	-	m_HmenM_UR_21_3	-	2e-13	42.9	8.7	2e-13	42.8	8.7	1.0	1	0	0	1	1	1	1	[109 - 174]
m_HmenM_UR_21_3	-	m_HmenM_UR_21_3	-	2e-13	42.9	8.7	2e-13	42.8	8.7	1.0	1	0	0	1	1	1	1	[109 - 174]

m_HmenM_UR_21_4	-	m_HmenM_UR_21_4	-	5.6e-34	109.5	4.1	6.1e-34	109.4	4.1	1.0	1	0	0	1	1	1	1	[21 - 176]
a_HmenM_UR_21_4	-	m_HmenM_UR_21_4	-	1.1e-33	108.6	3.7	1.1e-33	108.5	3.7	1.0	1	0	0	1	1	1	1	[21 - 176]
m_HmenM_UR_21_5	-	m_HmenM_UR_21_5	-	1.5e-07	24.1	2.3	1.5e-07	24.1	2.3	1.0	1	0	0	1	1	1	1	[167 - 126] (REVERSE SENSE)
a_HmenM_UR_21_5	-	m_HmenM_UR_21_5	-	2.7e-07	23.4	2.0	2.7e-07	23.4	2.0	1.0	1	0	0	1	1	1	1	[167 - 126] (REVERSE SENSE)
m_HmenM_UR_21_6	-	m_HmenM_UR_21_6	-	5.9e-25	80.9	9.3	6.3e-25	80.8	9.3	1.0	1	0	0	1	1	1	1	[163 - 50] (REVERSE SENSE)
a_HmenM_UR_21_6	-	m_HmenM_UR_21_6	-	1.3e-24	79.8	9.1	1.4e-24	79.7	9.1	1.0	1	0	0	1	1	1	1	[163 - 50] (REVERSE SENSE)
m_HmenM_UR_21_7	-	m_HmenM_UR_21_7	-	4.7e-07	21.7	2.8	4.7e-07	21.7	2.8	1.0	1	0	0	1	1	1	1	[37 - 2] (REVERSE SENSE)
a_HmenM_UR_21_7	-	m_HmenM_UR_21_7	-	7.9e-07	21.1	2.1	7.9e-07	21.1	2.1	1.0	1	0	0	1	1	1	1	[37 - 2] (REVERSE SENSE)
m_HmenM_UR_21_8	-	m_HmenM_UR_21_8	-	5.3e-39	126.5	13.6	5.7e-39	126.3	13.6	1.0	1	0	0	1	1	1	1	[174 - 1] (REVERSE SENSE)
a_HmenM_UR_21_8	-	m_HmenM_UR_21_8	-	9.2e-39	125.7	13.4	1e-38	125.6	13.4	1.0	1	0	0	1	1	1	1	[174 - 1] (REVERSE SENSE)
m_HmenM_UR_25_1	-	m_HmenM_UR_25_1	-	1.4e-07	23.8	4.0	1.4e-07	23.8	4.0	1.0	1	0	0	1	1	1	1	[3 - 41]
a_HmenM_UR_25_1	-	m_HmenM_UR_25_1	-	2.1e-07	23.3	3.2	2.1e-07	23.3	3.2	1.0	1	0	0	1	1	1	1	[3 - 41]
m_HmenM_UR_25_2	-	m_HmenM_UR_25_2	-	4e-07	21.6	0.8	4e-07	21.6	0.8	1.0	1	0	0	1	1	1	1	[40 - 2] (REVERSE SENSE)
a_HmenM_UR_25_2	-	m_HmenM_UR_25_2	-	1.1e-06	20.5	0.7	1.1e-06	20.5	0.7	1.0	1	0	0	1	1	1	1	[40 - 2] (REVERSE SENSE)
m_HmenM_UR_26_1	-	m_HmenM_UR_26_1	-	1.4e-05	16.5	0.3	1.4e-05	16.5	0.3	1.0	1	0	0	1	1	1	1	[34 - 2] (REVERSE SENSE)
a_HmenM_UR_26_1	-	m_HmenM_UR_26_1	-	3.5e-05	15.5	0.2	3.5e-05	15.5	0.2	1.0	1	0	0	1	1	1	1	[34 - 2] (REVERSE SENSE)
m_HmenM_UR_26_2	-	m_HmenM_UR_26_2	-	2.9e-15	48.9	3.4	3e-15	48.9	3.4	1.0	1	0	0	1	1	1	1	[78 - 1] (REVERSE SENSE)
a_HmenM_UR_26_2	-	m_HmenM_UR_26_2	-	6.8e-15	47.8	3.2	7e-15	47.8	3.2	1.0	1	0	0	1	1	1	1	[78 - 1] (REVERSE SENSE)
m_HmenM_UR_28_1	-	m_HmenM_UR_28_1	-	4e-08	24.2	0.2	4e-08	24.2	0.2	1.0	1	0	0	1	1	1	1	[2 - 40]
a_HmenM_UR_28_1	-	m_HmenM_UR_28_1	-	1.2e-07	22.9	0.2	1.2e-07	22.9	0.2	1.0	1	0	0	1	1	1	1	[2 - 40]
m_CmonM_UR_1_1	-	m_CmonM_UR_1_1	-	1.7e-07	21.9	0.2	1.7e-07	21.9	0.2	1.0	1	0	0	1	1	1	1	[20 - 55]
a_CmonM_UR_1_1	-	m_CmonM_UR_1_1	-	5.2e-07	20.7	0.1	5.2e-07	20.7	0.1	1.0	1	0	0	1	1	1	1	[20 - 55]
m_CmonM_UR_1_2	-	m_CmonM_UR_1_2	-	6.2e-09	26.7	0.2	6.2e-09	26.7	0.2	1.0	1	0	0	1	1	1	1	[12 - 56]
a_CmonM_UR_1_2	-	m_CmonM_UR_1_2	-	1.9e-08	25.5	0.2	1.9e-08	25.5	0.2	1.0	1	0	0	1	1	1	1	[12 - 56]
m_CmonM_UR_1_3	-	m_CmonM_UR_1_3	-	2e-08	25.7	0.3	2e-08	25.7	0.3	1.0	1	0	0	1	1	1	1	[37 - 2] (REVERSE SENSE)
a_CmonM_UR_1_3	-	m_CmonM_UR_1_3	-	5.7e-08	24.5	0.2	5.7e-08	24.5	0.2	1.0	1	0	0	1	1	1	1	[37 - 2] (REVERSE SENSE)
m_CmonM_UR_1_4	-	m_CmonM_UR_1_4	-	5.6e-06	18.4	0.4	5.6e-06	18.4	0.4	1.0	1	0	0	1	1	1	1	[30 - 1] (REVERSE SENSE)
a_CmonM_UR_1_4	-	m_CmonM_UR_1_4	-	1.7e-05	17.1	0.4	1.7e-05	17.1	0.4	1.0	1	0	0	1	1	1	1	[30 - 1] (REVERSE SENSE)
a_CmonM_UR_2_1	-	m_CmonM_UR_2_1	-	2e-30	98.2	8.0	2.1e-30	98.1	8.0	1.0	1	0	0	1	1	1	1	[4 - 144]
m_CmonM_UR_2_1	-	m_CmonM_UR_2_1	-	2e-30	98.2	8.0	2.1e-30	98.1	8.0	1.0	1	0	0	1	1	1	1	[4 - 144]
m_CmonM_UR_2_2	-	m_CmonM_UR_2_2	-	6e-15	46.9	1.0	6.2e-15	46.9	1.0	1.0	1	0	0	1	1	1	1	[135 - 67] (REVERSE SENSE)
a_CmonM_UR_2_2	-	m_CmonM_UR_2_2	-	1.3e-14	46.0	0.9	1.3e-14	46.0	0.9	1.0	1	0	0	1	1	1	1	[135 - 67] (REVERSE SENSE)
m_CmonM_UR_2_3	-	m_CmonM_UR_2_3	-	5e-18	57.5	0.9	5.3e-18	57.4	0.9	1.0	1	0	0	1	1	1	1	[137 - 51] (REVERSE SENSE)
a_CmonM_UR_2_3	-	m_CmonM_UR_2_3	-	1.3e-17	56.3	0.8	1.4e-17	56.2	0.8	1.0	1	0	0	1	1	1	1	[137 - 51] (REVERSE SENSE)
a_CmonM_UR_2_4	-	m_CmonM_UR_2_4	-	1.5e-06	19.3	0.3	1.5e-06	19.3	0.3	1.0	1	0	0	1	1	1	1	[67 - 32] (REVERSE SENSE)
m_CmonM_UR_2_4	-	m_CmonM_UR_2_4	-	1.5e-06	19.3	0.3	1.5e-06	19.3	0.3	1.0	1	0	0	1	1	1	1	[67 - 32] (REVERSE SENSE)
a_CmonM_UR_2_5	-	m_CmonM_UR_2_5	-	4.1e-10	31.6	0.6	4.2e-10	31.6	0.6	1.0	1	0	0	1	1	1	1	[48 - 1] (REVERSE SENSE)
m_CmonM_UR_2_5	-	m_CmonM_UR_2_5	-	4.1e-10	31.6	0.6	4.2e-10	31.6	0.6	1.0	1	0	0	1	1	1	1	[48 - 1] (REVERSE SENSE)
m_CmonM_UR_15_1	-	m_CmonM_UR_15_1	-	7.4e-06	16.8	0.5	7.4e-06	16.8	0.5	1.0	1	0	0	1	1	1	1	[9 - 38]
a_CmonM_UR_15_1	-	m_CmonM_UR_15_1	-	2.2e-05	15.7	0.3	2.2e-05	15.7	0.3	1.0	1	0	0	1	1	1	1	[9 - 38]
m_CmonM_UR_15_2	-	m_CmonM_UR_15_2	-	4.8e-06	18.4	0.8	4.8e-06	18.4	0.8	1.0	1	0	0	1	1	1	1	[31 - 2] (REVERSE SENSE)
a_CmonM_UR_15_2	-	m_CmonM_UR_15_2	-	1.3e-05	17.3	0.6	1.3e-05	17.3	0.6	1.1	1	0	0	1	1	1	1	[31 - 2] (REVERSE SENSE)
a_CmonM_UR_16_1	-	m_CmonM_UR_16_1	-	1.1e-06	20.2	0.0	1.1e-06	20.2	0.0	1.0	1	0	0	1	1	1	1	[13 - 45]
m_CmonM_UR_16_1	-	m_CmonM_UR_16_1	-	1.1e-06	20.2	0.0	1.1e-06	20.2	0.0	1.0	1	0	0	1	1	1	1	[13 - 45]
a_CmonM_UR_16_5	-	m_CmonM_UR_16_1	-	5.8e-06	18.4	0.1	3.7e-05	16.3	0.0	2.1	2	1	0	2	2	2	1	[147 - 239]
m_CmonM_UR_16_5	-	m_CmonM_UR_16_1	-	5.8e-06	18.4	0.1	3.7e-05	16.3	0.0	2.1	2	1	0	2	2	2	1	[147 - 239]
a_CmonM_UR_16_2	-	m_CmonM_UR_16_2	-	4.3e-09	28.7	2.2	4.3e-09	28.7	2.2	1.0	1	0	0	1	1	1	1	[78 - 125]
a_CmonM_UR_16_6	-	m_CmonM_UR_16_2	-	4.3e-09	28.7	2.2	4.3e-09	28.7	2.2	1.0	1	0	0	1	1	1	1	[272 - 319]
m_CmonM_UR_16_2	-	m_CmonM_UR_16_2	-	4.3e-09	28.7	2.2	4.3e-09	28.7	2.2	1.0	1	0	0	1	1	1	1	[78 - 125]
m_CmonM_UR_16_6	-	m_CmonM_UR_16_2	-	4.3e-09	28.7	2.2	4.3e-09	28.7	2.2	1.0	1	0	0	1	1	1	1	[272 - 319]
m_CmonM_UR_16_3	-	m_CmonM_UR_16_3	-	2.1e-11	36.0	4.8	2.1e-11	36.0	4.8	1.0	1	0	0	1	1	1	1	[94 - 147]
m_CmonM_UR_16_7	-	m_CmonM_UR_16_3	-	2.1e-11	36.0	4.8	2.1e-11	36.0	4.8	1.0	1	0	0	1	1	1	1	[288 - 341]
a_CmonM_UR_16_3	-	m_CmonM_UR_16_3	-	3.6e-11	35.3	4.1	3.6e-11	35.3	4.1	1.0	1	0	0	1	1	1	1	[94 - 147]
a_CmonM_UR_16_7	-	m_CmonM_UR_16_3	-	3.6e-11	35.3	4.1	3.6e-11	35.3	4.1	1.0	1	0	0	1	1	1	1	[288 - 341]
a_CmonM_UR_16_4	-	m_CmonM_UR_16_4	-	5.7e-21	66.5	1.7	6.1e-21	66.4	1.7	1.0	1	0	0	1	1	1	1	[113 - 214]
m_CmonM_UR_16_4	-	m_CmonM_UR_16_4	-	5.7e-21	66.5	1.7	6.1e-21	66.4	1.7	1.0	1	0	0	1	1	1	1	[113 - 214]
a_CmonM_UR_16_8	-	m_CmonM_UR_16_4	-	1.2e-12	41.6	1.6	1.2e-12	41.6	1.6	1.0	1	0	0	1	1	1	1	[307 - 378]
m_CmonM_UR_16_8	-	m_CmonM_UR_16_4	-	1.2e-12	41.6	1.6	1.2e-12	41.6	1.6	1.0	1	0	0	1	1	1	1	[307 - 378]
a_CmonM_UR_16_5	-	m_CmonM_UR_16_5	-	3.1e-19	60.9	0.8	3.3e-19	60.9	0.8	1.0	1	0	0	1	1	1	1	[147 - 239]
m_CmonM_UR_16_5	-	m_CmonM_UR_16_5	-	3.1e-19	60.9	0.8	3.3e-19	60.9	0.8	1.0	1	0	0	1	1	1	1	[147 - 239]
a_CmonM_UR_16_1	-	m_CmonM_UR_16_5	-	0.00016	17.3	0.0	0.00016	17.3	0.0	1.0	1	0	0	1	1	1	1	[13 - 45]
m_CmonM_UR_16_1	-	m_CmonM_UR_16_5	-	0.00016	17.3	0.0	0.00016	17.3	0.0	1.0	1	0	0	1	1	1	1	[13 - 45]
a_CmonM_UR_16_9	-	m_CmonM_UR_16_5	-	0.00018	17.1	0.2	0.00021	16.9	0.2	1.4	1	1	0	1	1	1	1	[341 - 439]
m_CmonM_UR_16_9	-	m_CmonM_UR_16_5	-	0.00018	17.1	0.2	0.00021	16.9	0.2	1.4	1	1	0	1	1	1	1	[341 - 439]
a_CmonM_UR_16_2	-	m_CmonM_UR_16_6	-	4.3e-09	28.7	2.2	4.3e-09	28.7	2.2	1.0	1	0	0	1	1	1	1	[78 - 125]
a_CmonM_UR_16_6	-	m_CmonM_UR_16_6	-	4.3e-09	28.7	2.2	4.3e-09	28.7	2.2	1.0	1	0	0	1	1	1	1	[272 - 319]
m_CmonM_UR_16_2	-	m_CmonM_UR_16_6	-	4.3e-09	28.7	2.2	4.3e-09	28.7	2.2	1.0	1	0	0	1	1	1	1	[78 - 125]
m_CmonM_UR_16_6	-	m_CmonM_UR_16_6	-	4.3e-09	28.7	2.2	4.3e-09	28.7	2.2	1.0	1	0	0	1	1	1	1	[272 - 319]
m_CmonM_UR_16_3	-	m_CmonM_UR_16_7	-	2.1e-11	36.0	4.8	2.1e-11	36.0	4.8	1.0	1	0	0	1	1	1	1	[94 - 147]
m_CmonM_UR_16_7	-	m_CmonM_UR_16_7	-	2.1e-11	36.0	4.8	2.1e-11	36.0	4.8	1.0	1	0	0	1	1	1	1	[288 - 341]
a_CmonM_UR_16_3	-	m_CmonM_UR_16_7	-	3.6e-11	35.3	4.1	3.6e-11	35.3	4.1	1.0	1	0	0	1	1	1	1	[94 - 147]
a_CmonM_UR_16_7	-	m_CmonM_UR_16_7	-	3.6e-11	35.3	4.1	3.6e-11	35.3	4.1	1.0	1	0	0	1	1	1	1	[288 - 341]
a_CmonM_UR_16_8	-	m_CmonM_UR_16_8	-	4.6e-14	44.2	1.8	4.8e-14	44.2	1.8	1.0	1	0	0	1	1	1	1	[307 - 378]
m_CmonM_UR_16_8	-	m_CmonM_UR_16_8	-	4.6e-14	44.2	1.8	4.8e-14	44.2	1.8	1.0	1	0	0	1	1	1	1	[307 - 378]
a_CmonM_UR_16_4	-	m_CmonM_UR_16_8	-	4.7e-13	41.3	1.5	6.5e-13	40.9	1.5	1.2	1	0	0	1	1	1	1	[113 - 214]
m_CmonM_UR_16_4	-	m_CmonM_UR_16_8	-	4.7e-13	41.3	1.5	6.5e-13	40.9	1.5	1.2	1	0	0	1	1	1	1	[113 - 214]
a_CmonM_UR_16_9	-	m_CmonM_UR_16_9	-	1.6e-19	61.9	1.9	1.7e-19	61.8	1.9	1.0	1	0	0	1	1	1	1	[341 - 439]
m_CmonM_UR_16_9	-	m_CmonM_UR_16_9	-	1.6e-19	61.9	1.9	1.7e-19	61.8	1.9	1.0	1	0	0	1	1	1	1	[341 - 439]
a_CmonM_UR_16_5	-	m_CmonM_UR_16_9	-	0.00018	17.1	0.3	0.0002	17.0	0.3	1.4	1	1	0	1	1	1	1	[147 - 239]
m_CmonM_UR_16_5	-	m_CmonM_UR_16_9	-	0.00018	17.1	0.3	0.0002	17.0	0.3	1.4	1	1	0	1	1	1	1	[147 - 239]
m_CmonM_UR_16_10	-	m_CmonM_UR_16_10	-	4e-10	31.0	0.1	4e-10	31.0	0.1	1.0	1	0	0	1	1	1	1	[450 - 497]
a_CmonM_UR_16_10	-	m_CmonM_UR_16_10	-	1.1e-09	29.8	0.1	1.1e-09	29.8	0.1	1.0	1	0	0	1	1	1	1	[450 - 497]
m_CmonM_UR_16_11	-	m_CmonM_UR_16_11	-	1.9e-11	35.8	1.8	1.9e-11	35.8	1.8	1.0	1	0	0	1	1	1	1	[466 - 519]
a_CmonM_UR_16_11	-	m_CmonM_UR_16_11	-	3.4e-11	35.0	1.2	3.4e-11	35.0	1.2	1.0	1	0	0	1	1	1	1	[466 - 519]
m_CmonM_UR_16_12	-	m_CmonM_UR_16_12	-	1.7e-08	25.9	1.0	1.7e-08	25.9	1.0	1.0	1	0	0	1	1	1	1	[510 - 463] (REVERSE SENSE)
a_CmonM_UR_16_12	-	m_CmonM_UR_16_12	-	5.1e-08	24.6	1.0	5.1e-08	24.6	1.0	1.0	1	0	0	1	1	1	1	[510 - 463] (REVERSE SENSE)
a_CmonM_UR_16_13	-	m_CmonM_UR_16_13	-	8.2e-18	56.9	4.3	8.6e-18	56.9	4.3	1.0	1	0	0	1	1	1	1	[518 - 438] (REVERSE SENSE)
m_CmonM_UR_16_13	-	m_CmonM_UR_16_13	-	8.2e-18	56.9	4.3	8.6e-18	56.9	4.3	1.0	1	0	0	1	1	1	1	[518 - 438] (REVERSE SENSE)
m_CmonM_UR_16_14	-	m_CmonM_UR_16_14	-	2.9e-07	22.6	0.4	2.9e-07	22.6	0.4	1.0	1	0	0	1	1	1	1	[416 - 381] (REVERSE SENSE)
a_CmonM_UR_16_14	-	m_CmonM_UR_16_14	-	6.5e-07	21.7	0.3	6.5e-07	21.7	0.3	1.0	1	0	0	1	1	1	1	[416 - 381] (REVERSE SENSE)
m_CmonM_UR_16_15	-	m_CmonM_UR_16_15	-	1.1e-12	39.9	0.4	1.2e-12	39.8	0.4	1.0	1	0	0	1	1	1	1	[432 - 370] (REVERSE SENSE)
a_CmonM_UR_16_15	-	m_CmonM_UR_16_15	-	3.3e-12	38.6	0.5	3.4e-12	38.5	0.5	1.0	1	0	0	1	1	1	1	[432 - 370] (REVERSE SENSE)
a_CmonM_UR_16_16	-	m_CmonM_UR_16_16	-	3.7e-07	22.0	0.5	3.7e-07	22.0	0.5	1.0	1	0	0	1	1	1	1	[370 - 335] (REVERSE SENSE)
a_CmonM_UR_16_21	-	m_CmonM_UR_16_16	-	3.7e-07	22.0	0.5	3.7e-07	22.0	0.5	1.0	1	0	0	1	1	1	1	[176 - 141] (REVERSE SENSE)
m_CmonM_UR_16_16	-	m_CmonM_UR_16_16	-	3.7e-07	22.0	0.5	3.7e-07	22.0	0.5	1.0	1	0	0	1	1	1	1	[370 - 335] (REVERSE SENSE)
m_CmonM_UR_16_21	-	m_CmonM_UR_16_16	-	3.7e-07	22.0	0.5	3.7e-07	22.0	0.5	1.0	1	0	0	1	1	1	1	[176 - 141] (REVERSE SENSE)
a_CmonM_UR_16_17	-	m_CmonM_UR_16_17	-	1.4e-09	29.7	0.1	1.4e-09	29.7	0.1	1.0	1	0	0	1	1	1	1	[308 - 264] (REVERSE SENSE)
a_CmonM_UR_16_22	-	m_CmonM_UR_16_17	-	1.4e-09	29.7	0.1	1.4e-09	29.7	0.1	1.0	1	0	0	1	1	1	1	[114 - 70] (REVERSE SENSE)
m_CmonM_UR_16_17	-	m_CmonM_UR_16_17	-	1.4e-09	29.7	0.1	1.4e-09	29.7	0.1	1.0	1	0	0	1	1	1	1	[308 - 264] (REVERSE SENSE)
m_CmonM_UR_16_22	-	m_CmonM_UR_16_17	-	1.4e-09	29.7	0.1	1.4e-09	29.7	0.1	1.0	1	0	0	1	1	1	1	[114 - 70] (REVERSE SENSE)
m_CmonM_UR_16_18	-	m_CmonM_UR_16_18	-	2.9e-06	18.5	0.1	2.9e-06	18.5	0.1	1.0	1	0	0	1	1	1	1	[277 - 248] (REVERSE SENSE)
m_CmonM_UR_16_23	-	m_CmonM_UR_16_18	-	2.9e-06	18.5	0.1	2.9e-06	18.5	0.1	1.0	1	0	0	1	1	1	1	[83 - 54] (REVERSE SENSE)
a_CmonM_UR_16_18	-	m_CmonM_UR_16_18	-	1e-05	17.2	0.1	1e-05	17.2	0.1	1.0	1	0	0	1	1	1	1	[277 - 248] (REVERSE SENSE)
a_CmonM_UR_16_23	-	m_CmonM_UR_16_18	-	1e-05	17.2	0.1	1e-05	17.2	0.1	1.0	1	0	0	1	1	1	1	[83 - 54] (REVERSE SENSE)
m_CmonM_UR_16_19	-	m_CmonM_UR_16_19	-	1.7e-06	19.7	0.1	1.7e-06	19.7	0.1	1.0	1	0	0	1	1	1	1	[257 - 228] (REVERSE SENSE)
m_CmonM_UR_16_24	-	m_CmonM_UR_16_19	-	1.7e-06	19.7	0.1	1.7e-06	19.7	0.1	1.0	1	0	0	1	1	1	1	[63 - 34] (REVERSE SENSE)
a_CmonM_UR_16_19	-	m_CmonM_UR_16_19	-	5.6e-06	18.4	0.1	5.6e-06	18.4	0.1	1.0	1	0	0	1	1	1	1	[257 - 228] (REVERSE SENSE)
a_CmonM_UR_16_24	-	m_CmonM_UR_16_19	-	5.6e-06	18.4	0.1	5.6e-06	18.4	0.1	1.0	1	0	0	1	1	1	1	[63 - 34] (REVERSE SENSE)
m_CmonM_UR_16_20	-	m_CmonM_UR_16_20	-	1.7e-20	65.2	3.1	1.8e-20	65.1	3.1	1.0	1	0	0	1	1	1	1	[249 - 148] (REVERSE SENSE)

a_CmonM_UR_16_20	-	m_CmonM_UR_16_20	-	3.2e-20	64.4	2.7	3.3e-20	64.3	2.7	1.0	1	0	0	1	1	1	1	[249 - 148] (REVERSE SENSE)
m_CmonM_UR_16_25	-	m_CmonM_UR_16_20	-	1.5e-08	29.5	2.3	1.5e-08	29.5	2.3	1.0	1	0	0	1	1	1	1	[55 - 2] (REVERSE SENSE)
a_CmonM_UR_16_25	-	m_CmonM_UR_16_20	-	2.8e-08	28.7	1.9	2.8e-08	28.7	1.9	1.0	1	0	0	1	1	1	1	[55 - 2] (REVERSE SENSE)
a_CmonM_UR_16_16	-	m_CmonM_UR_16_21	-	3.7e-07	22.0	0.5	3.7e-07	22.0	0.5	1.0	1	0	0	1	1	1	1	[370 - 335] (REVERSE SENSE)
a_CmonM_UR_16_21	-	m_CmonM_UR_16_21	-	3.7e-07	22.0	0.5	3.7e-07	22.0	0.5	1.0	1	0	0	1	1	1	1	[176 - 141] (REVERSE SENSE)
m_CmonM_UR_16_16	-	m_CmonM_UR_16_21	-	3.7e-07	22.0	0.5	3.7e-07	22.0	0.5	1.0	1	0	0	1	1	1	1	[370 - 335] (REVERSE SENSE)
m_CmonM_UR_16_21	-	m_CmonM_UR_16_21	-	3.7e-07	22.0	0.5	3.7e-07	22.0	0.5	1.0	1	0	0	1	1	1	1	[176 - 141] (REVERSE SENSE)
a_CmonM_UR_16_17	-	m_CmonM_UR_16_22	-	1.4e-09	29.7	0.1	1.4e-09	29.7	0.1	1.0	1	0	0	1	1	1	1	[308 - 264] (REVERSE SENSE)
a_CmonM_UR_16_22	-	m_CmonM_UR_16_22	-	1.4e-09	29.7	0.1	1.4e-09	29.7	0.1	1.0	1	0	0	1	1	1	1	[114 - 70] (REVERSE SENSE)
m_CmonM_UR_16_17	-	m_CmonM_UR_16_22	-	1.4e-09	29.7	0.1	1.4e-09	29.7	0.1	1.0	1	0	0	1	1	1	1	[308 - 264] (REVERSE SENSE)
m_CmonM_UR_16_22	-	m_CmonM_UR_16_22	-	1.4e-09	29.7	0.1	1.4e-09	29.7	0.1	1.0	1	0	0	1	1	1	1	[114 - 70] (REVERSE SENSE)
m_CmonM_UR_16_18	-	m_CmonM_UR_16_23	-	2.9e-06	18.5	0.1	2.9e-06	18.5	0.1	1.0	1	0	0	1	1	1	1	[277 - 248] (REVERSE SENSE)
m_CmonM_UR_16_23	-	m_CmonM_UR_16_23	-	2.9e-06	18.5	0.1	2.9e-06	18.5	0.1	1.0	1	0	0	1	1	1	1	[83 - 54] (REVERSE SENSE)
a_CmonM_UR_16_18	-	m_CmonM_UR_16_23	-	1e-05	17.2	0.1	1e-05	17.2	0.1	1.0	1	0	0	1	1	1	1	[277 - 248] (REVERSE SENSE)
a_CmonM_UR_16_23	-	m_CmonM_UR_16_23	-	1e-05	17.2	0.1	1e-05	17.2	0.1	1.0	1	0	0	1	1	1	1	[83 - 54] (REVERSE SENSE)
m_CmonM_UR_16_19	-	m_CmonM_UR_16_24	-	1.7e-06	19.7	0.1	1.7e-06	19.7	0.1	1.0	1	0	0	1	1	1	1	[257 - 228] (REVERSE SENSE)
m_CmonM_UR_16_24	-	m_CmonM_UR_16_24	-	1.7e-06	19.7	0.1	1.7e-06	19.7	0.1	1.0	1	0	0	1	1	1	1	[63 - 34] (REVERSE SENSE)
a_CmonM_UR_16_19	-	m_CmonM_UR_16_24	-	5.6e-06	18.4	0.1	5.6e-06	18.4	0.1	1.0	1	0	0	1	1	1	1	[257 - 228] (REVERSE SENSE)
a_CmonM_UR_16_24	-	m_CmonM_UR_16_24	-	5.6e-06	18.4	0.1	5.6e-06	18.4	0.1	1.0	1	0	0	1	1	1	1	[63 - 34] (REVERSE SENSE)
m_CmonM_UR_16_25	-	m_CmonM_UR_16_25	-	4.3e-10	31.2	2.3	4.3e-10	31.2	2.3	1.0	1	0	0	1	1	1	1	[55 - 2] (REVERSE SENSE)
a_CmonM_UR_16_25	-	m_CmonM_UR_16_25	-	8.6e-10	30.4	1.9	8.6e-10	30.4	1.9	1.0	1	0	0	1	1	1	1	[55 - 2] (REVERSE SENSE)
m_CmonM_UR_16_20	-	m_CmonM_UR_16_25	-	4.2e-09	28.5	2.3	4.2e-09	28.5	2.3	1.6	2	0	0	2	2	2	1	[249 - 148] (REVERSE SENSE)
a_CmonM_UR_16_20	-	m_CmonM_UR_16_25	-	8.2e-09	27.8	1.8	8.2e-09	27.8	1.8	1.6	2	0	0	2	2	2	1	[249 - 148] (REVERSE SENSE)
m_CmonM_UR_24_1	-	m_CmonM_UR_24_1	-	7e-15	46.8	1.4	7.2e-15	46.8	1.4	1.0	1	0	0	1	1	1	1	[63 - 131]
a_CmonM_UR_24_1	-	m_CmonM_UR_24_1	-	1.3e-14	46.0	0.9	1.4e-14	46.0	0.9	1.0	1	0	0	1	1	1	1	[63 - 131]
m_CmonM_UR_24_2	-	m_CmonM_UR_24_2	-	2.7e-36	116.6	7.9	2.9e-36	116.5	7.9	1.0	1	0	0	1	1	1	1	[98 - 271]
a_CmonM_UR_24_2	-	m_CmonM_UR_24_2	-	4.6e-36	115.9	7.4	5e-36	115.8	7.4	1.0	1	0	0	1	1	1	1	[98 - 271]
a_CmonM_UR_24_3	-	m_CmonM_UR_24_3	-	2.9e-10	32.0	7.4	3e-10	32.0	7.4	1.0	1	0	0	1	1	1	1	[271 - 324]
m_CmonM_UR_24_3	-	m_CmonM_UR_24_3	-	2.9e-10	32.0	7.4	3e-10	32.0	7.4	1.0	1	0	0	1	1	1	1	[271 - 324]
a_CmonM_UR_24_4	-	m_CmonM_UR_24_4	-	8.7e-21	64.8	1.9	9.3e-21	64.7	1.9	1.0	1	0	0	1	1	1	1	[281 - 382]
m_CmonM_UR_24_4	-	m_CmonM_UR_24_4	-	8.7e-21	64.8	1.9	9.3e-21	64.7	1.9	1.0	1	0	0	1	1	1	1	[281 - 382]
m_CmonM_UR_24_5	-	m_CmonM_UR_24_5	-	1.2e-11	35.3	0.2	1.2e-11	35.3	0.2	1.0	1	0	0	1	1	1	1	[343 - 396]
a_CmonM_UR_24_5	-	m_CmonM_UR_24_5	-	3.4e-11	34.1	0.1	3.4e-11	34.1	0.1	1.0	1	0	0	1	1	1	1	[343 - 396]
a_CmonM_UR_24_6	-	m_CmonM_UR_24_6	-	5e-29	92.9	2.5	5.3e-29	92.8	2.5	1.0	1	0	0	1	1	1	1	[330 - 458]
m_CmonM_UR_24_6	-	m_CmonM_UR_24_6	-	5e-29	92.9	2.5	5.3e-29	92.8	2.5	1.0	1	0	0	1	1	1	1	[330 - 458]
m_CmonM_UR_24_7	-	m_CmonM_UR_24_7	-	1.8e-44	144.9	15.6	2e-44	144.8	15.6	1.0	1	0	0	1	1	1	1	[442 - 251] (REVERSE SENSE)
a_CmonM_UR_24_7	-	m_CmonM_UR_24_7	-	4.1e-44	143.8	15.5	4.5e-44	143.7	15.5	1.0	1	0	0	1	1	1	1	[442 - 251] (REVERSE SENSE)
a_CmonM_UR_24_8	-	m_CmonM_UR_24_8	-	1.4e-49	161.4	11.1	1.5e-49	161.3	11.1	1.0	1	0	0	1	1	1	1	[395 - 168] (REVERSE SENSE)
m_CmonM_UR_24_8	-	m_CmonM_UR_24_8	-	1.4e-49	161.4	11.1	1.5e-49	161.3	11.1	1.0	1	0	0	1	1	1	1	[395 - 168] (REVERSE SENSE)
m_CmonM_UR_24_9	-	m_CmonM_UR_24_9	-	3.3e-08	24.9	0.1	3.3e-08	24.9	0.1	1.0	1	0	0	1	1	1	1	[178 - 134] (REVERSE SENSE)
a_CmonM_UR_24_9	-	m_CmonM_UR_24_9	-	7.9e-08	23.9	0.1	7.9e-08	23.9	0.1	1.0	1	0	0	1	1	1	1	[178 - 134] (REVERSE SENSE)
m_CmonM_UR_24_10	-	m_CmonM_UR_24_10	-	1.5e-09	30.7	6.9	1.6e-09	30.6	6.9	1.0	1	0	0	1	1	1	1	[122 - 72] (REVERSE SENSE)
a_CmonM_UR_24_10	-	m_CmonM_UR_24_10	-	3.3e-09	29.7	6.5	3.3e-09	29.7	6.5	1.0	1	0	0	1	1	1	1	[122 - 72] (REVERSE SENSE)
m_CmonM_UR_24_11	-	m_CmonM_UR_24_11	-	1.7e-81	266.5	15.2	1.9e-81	266.4	15.2	1.0	1	0	0	1	1	1	1	[447 - 67] (REVERSE SENSE)
a_CmonM_UR_24_11	-	m_CmonM_UR_24_11	-	4.5e-81	265.2	15.3	5e-81	265.1	15.3	1.0	1	0	0	1	1	1	1	[447 - 67] (REVERSE SENSE)
CmonM_MORF	-	m_CmonM_UR_24_11	-	6.2e-05	19.0	3.5	6.2e-05	19.0	3.5	1.4	1	1	0	1	1	1	1	-
m_CmonM_UR_24_12	-	m_CmonM_UR_24_12	-	2.8e-19	61.6	6.6	2.9e-19	61.6	6.6	1.0	1	0	0	1	1	1	1	[124 - 32] (REVERSE SENSE)
a_CmonM_UR_24_12	-	m_CmonM_UR_24_12	-	6e-19	60.6	6.6	6.3e-19	60.6	6.6	1.0	1	0	0	1	1	1	1	[124 - 32] (REVERSE SENSE)
m_CmonM_UR_27_1	-	m_CmonM_UR_27_1	-	7.3e-37	118.7	6.8	7.9e-37	118.6	6.8	1.0	1	0	0	1	1	1	1	[2 - 181]
a_CmonM_UR_27_1	-	m_CmonM_UR_27_1	-	1.6e-36	117.7	6.5	1.7e-36	117.6	6.5	1.0	1	0	0	1	1	1	1	[2 - 181]
m_CmonM_UR_27_2	-	m_CmonM_UR_27_2	-	5.7e-22	69.6	2.7	6.1e-22	69.6	2.7	1.0	1	0	0	1	1	1	1	[91 - 195]
a_CmonM_UR_27_2	-	m_CmonM_UR_27_2	-	1.2e-21	68.7	2.4	1.3e-21	68.6	2.4	1.0	1	0	0	1	1	1	1	[91 - 195]
m_CmonM_UR_27_3	-	m_CmonM_UR_27_3	-	2.7e-09	28.4	1.1	2.7e-09	28.4	1.1	1.0	1	0	0	1	1	1	1	[194 - 138] (REVERSE SENSE)
a_CmonM_UR_27_3	-	m_CmonM_UR_27_3	-	7.6e-09	27.2	1.0	7.6e-09	27.2	1.0	1.0	1	0	0	1	1	1	1	[194 - 138] (REVERSE SENSE)
a_CmonM_UR_27_4	-	m_CmonM_UR_27_4	-	2e-24	78.3	3.6	2.2e-24	78.2	3.6	1.0	1	0	0	1	1	1	1	[132 - 7] (REVERSE SENSE)
m_CmonM_UR_27_4	-	m_CmonM_UR_27_4	-	2e-24	78.3	3.6	2.2e-24	78.2	3.6	1.0	1	0	0	1	1	1	1	[132 - 7] (REVERSE SENSE)
m_CmonM_UR_27_5	-	m_CmonM_UR_27_5	-	9.7e-27	86.6	7.0	1e-26	86.5	7.0	1.0	1	0	0	1	1	1	1	[128 - 3] (REVERSE SENSE)
a_CmonM_UR_27_5	-	m_CmonM_UR_27_5	-	2e-26	85.6	7.0	2.1e-26	85.5	7.0	1.0	1	0	0	1	1	1	1	[128 - 3] (REVERSE SENSE)

`hmmsearch` ANALYSIS RESULTS

Search was performed using the unionoid F-ORF and M-ORF HMM profiles built with `hmmbuild` from Mitchell et al. (2016) (refer to that paper for details on these HMM profiles). The "per-target" output table of each analysis is shown. Notable hits cited in the paper are highlighted in yellow for reader's convenience.

• F-ORF profile built with default settings

#				--- full sequence ----			--- best 1 domain ----			--- domain number estimation ----								
# target name	accession	query name	accession	E-value	score	bias	E-value	score	bias	exp	reg	clu	ov	env	dom	rep	inc	description of target
CmonF_FORF	-	FORF_profile00	-	3.2e-21	71.4	10.8	4.1e-21	71.0	10.8	1.1	1	0	0	1	1	1	1	-
HmenF_FORF	-	FORF_profile00	-	4.9e-20	67.6	13.7	5.9e-20	67.3	13.7	1.0	1	0	0	1	1	1	1	-

• F-ORF profile built with custom settings

#				--- full sequence ----			--- best 1 domain ----			--- domain number estimation ----								
# target name	accession	query name	accession	E-value	score	bias	E-value	score	bias	exp	reg	clu	ov	env	dom	rep	inc	description of target
CmonF_FORF	-	FORF_profile01	-	1.1e-33	112.6	21.9	1.2e-33	112.5	21.9	1.0	1	0	0	1	1	1	1	-
HmenF_FORF	-	FORF_profile01	-	7.4e-27	90.1	24.0	8.5e-27	89.9	24.0	1.0	1	0	0	1	1	1	1	-

• M-ORF profile built with default settings

#				--- full sequence ----			--- best 1 domain ----			--- domain number estimation ----								
# target name	accession	query name	accession	E-value	score	bias	E-value	score	bias	exp	reg	clu	ov	env	dom	rep	inc	description of target
HmenM_MORF	-	MORF_profile00	-	1.2e-47	157.7	64.5	3.3e-45	149.8	64.5	2.2	1	1	0	1	1	1	1	-
CmonM_MORF	-	MORF_profile00	-	1.3e-11	40.4	14.3	2.5e-11	39.5	14.3	1.4	1	1	0	1	1	1	1	-

• M-ORF profile built with custom settings

#				--- full sequence ----			--- best 1 domain ----			--- domain number estimation ----								
# target name	accession	query name	accession	E-value	score	bias	E-value	score	bias	exp	reg	clu	ov	env	dom	rep	inc	description of target
HmenM_MORF	-	MORF_profile01	-	4.3e-196	648.8	63.8	4.8e-196	648.7	63.8	1.0	1	0	0	1	1	1	1	-
CmonM_MORF	-	MORF_profile01	-	7.5e-19	62.1	37.3	2.6e-16	53.7	9.3	2.3	1	1	1	2	2	2	2	-
a_Atra_UR_22_18	-	MORF_profile01	-	0.048	6.5	3.6	0.042	6.7	3.6	1.0	1	0	0	1	1	1	0	[198 - 133] (REVERSE SENSE)
m_Atra_UR_22_18	-	MORF_profile01	-	0.048	6.5	3.6	0.042	6.7	3.6	1.0	1	0	0	1	1	1	0	[198 - 133] (REVERSE SENSE)
