# Supplementary material for: Evolution of sex-dependent mtDNA transmission in freshwater mussels (Bivalvia: Unionida)
Source: Sci Rep. 2017 May 8;7:1551. doi: 10.1038/s41598-017-01708-1 (PMC5431520; doi:10.1038/s41598-017-01708-1)
Supplement: Supplementary file 5 — Supplementary Information 5 [file 41598_2017_1708_MOESM5_ESM.pdf]

Evolution of sex-dependent mtDNA transmission in freshwater mussels (Bivalvia: Unionida)

Davide Guerra[1], Federico Plazzi[2], Donald T. Stewart[3], Arthur E. Bogan[4], Walter R. Hoeh[5] & Sophie Breton[1]

[1]Département de Sciences Biologiques, Université de Montréal, Montréal H2V 2S9, Quebéc, Canada. [2]Dipartimento di Scienze Biologiche, Geologiche ed Ambientali (BiGeA), Università di Bologna, Bologna 40126, Italy. [3]Department of Biology, Acadia University, Wolfville B4P 2R6, Nova Scotia, Canada. [4]North Carolina Museum of Natural Sciences, Raleigh, NC 27607, USA. [5]Department of Biological Sciences, Kent State University, Kent, OH 44242, USA.

Supplementary Material 5

Structural characterization of proteins

(Supplementary Figure S4)

Supplementary Figure S4 [pages 3-18]. Structural alignment of protein sequences of interest made with T-Coffee. Abbreviations: Atra, *Anodontites trapesialis*; CmonF, *Cumberlandia monodonta* F; CmonM, *C. monodonta* M; HmenF, *Hyridella menziesii* F; HmenM, *H. menziesii* M.

(a) [page 3]: CmonM M-*ORF* vs CmonM_UR_24_11.

(b) [page 4]: HmenM *COX2* vs CmonM *COX2*.

(c) [page 6]: HmenF F-*ORF* vs CmonF F-*ORF*.

(d) [page 7]: HmenM M-*ORF* vs CmonM M-*ORF*.

(e) [page 8]: Atra *ATP8* vs Atra_UR_21_9.

(f) [page 9]: HmenM M-*ORF* vs Atra_UR_21_9.

(g) [page 10]: CmonM M-*ORF* vs Atra_UR_21_9.

(h) [page 11]: CmonM_UR_24_11 vs Atra_UR_21_9.

(i) [page 12]: HmenF F-*ORF* vs Atra_UR_21_9.

(l) [page 13]: CmonF F-*ORF* vs Atra_UR_21_9.

(m) [page 14]: Atra *ATP8* vs Atra_UR_22_18.

(n) [page 15]: Atra_UR_21_9 vs Atra_UR_22_18.

(o) [page 16]: Atra_UR_22_18 vs HmenM M-*ORF*.

(p) [page 17]: Atra_UR_22_18 vs CmonM M-*ORF*.

(q) [page 18]: Atra_UR_22_18 vs CmonM_UR_24_11.

a

```
T-COFFEE, Version_11.00.8cbe486 2014-08-12 22:05:29 - Revision 8cbe486 - Build 477
  Cedric Notredame
  CPU TIME:0 sec.
  SCORE=91
  *
   BAD AVG GOOD
  *
  CmonM_MORF       :  91
  CmonM_UR_24_11   :  91
  cons             :   9

  CmonM_MORF         1 ----------------------MKATLCKVIEFVLDNGWLCLFYF    23
  CmonM_UR_24_11     1 VVCLSSVDCGVEVPLVLDGALYLKALFGAIREFFHENSLFLLIIA    45

  cons               1                       :**  :   :  **. :*.  :  *:      45


  CmonM_MORF        24 VLFMACSNVLWRVYKVRKGLYKKVKRVIGVIWKPTAVSVKIKKDK    68
  CmonM_UR_24_11    46 VLVCICVSAFMWVYERRKVFVKKVKRGLKGLWKSIKVDFSVGGSG    90

  cons              46 **.  *  ..:  **: **  :  *****  :   :**.   *...:  .   90


  CmonM_MORF        69 VEKPKVMEKAKKGKKAAVSGKVGKKSG------------------    95
  CmonM_UR_24_11    91 WVRLGAM-----------SMKVLKWFSRFCVRVLWLVTLFVLLSV   124

  cons              91    :   .*           *  ** *   .                      135


  CmonM_MORF        96 --G   96
  CmonM_UR_24_11   125 FLG  127

  cons             136   *  138
```

b

```
T-COFFEE, Version_11.00.d625267 2016-01-11 15:25:41 - Revision d625267 - Build 507
  Cedric Notredame
  CPU TIME:0 sec.
  SCORE=84
  *
   BAD AVG GOOD
  *
  HmenM_MCOX2      :   84
  CmonM_MCOX2      :   84
  cons             :    8

  HmenM_MCOX2         1 ------------------MSLWGQLGLQDGLSSAALEIGSLHDHAMAI   30
  CmonM_MCOX2         1 VFSVEVLAGLSCLWTVSEMSLWGQFGFQEGATVLGIEISDLYDHVMFF   48

  cons                1                   ******:*:*:* :  .:**..*:**.* :   48


  HmenM_MCOX2        31 MVLIFSFVGFVFFKVVLEEGGCTTMLDSQWVEFLWTSLPLGLLIMLGL   78
  CmonM_MCOX2        49 SVLVFSFVGYLFCKMLMSVFTCRVLLEKQWLEFIWTFLPAGVLLAIGL   96

  cons               49  **:*****::* *:::.   * .:*:.**:**:** ** *:*: :**   96


  HmenM_MCOX2        79 PSIRLLYLLDDPADALISMKVLGRQWYWTYEYSDFIGGWGDEPIYFDS  126
  CmonM_MCOX2        97 PSVHLLYVMEDVGAPQITVKAIGHQWYWSYEYCDLG---GEDSISFDS  141

  cons               97 **::***:::* . . *::*.:*:****:***.*:    *::.* ***  144


  HmenM_MCOX2       127 YMMSSVDLSDGGYRLLEVDNRCVVPYGVGVRVLVSSSDVVHSWAVPSL  174
  CmonM_MCOX2       142 YMVPSVDLVVGSYRLLDVDNRFVVPYGVPMRVLVSSSDVIHSWAIPSA  189

  cons              145 **:.****  *.****:**** ****** :*********:****:**   192


  HmenM_MCOX2       175 GVKVDSIPGRINQVILSLVGSGVVYGQCSELCGVNHSFMPICLEAVRS  222
  CmonM_MCOX2       190 GVKVDGVVGRVNQAGLGFFGPGVVYGQCSELCGVNHSFMPICGEVVSC  237

  cons              193 *****.: **:**. *.:.*.********************* *.* .  240


  HmenM_MCOX2       223 DVYGLWVSPPVAEEVSEAVKAFDGEGESVGWWGWLIGKVMKAMWVVGN  270
  CmonM_MCOX2       238 EAYALWLLPKNCP-AGKSIWDYCLYWGGLLWWG--CGRV--AYWT---  277

  cons              241 :.*.**: *  .  ..:::  :     .: ***   *:*  * *.     288


  HmenM_MCOX2       271 GLMLVGITFVKVYMGWWVFFFNVFIYAPVELFIGFLFSVSWWSFSLLT  318
  CmonM_MCOX2       278 -------S--FAYLGWWKIFGYYFVYMPVKVSVDTTVSVVEGSVRSCC  316

  cons              289        :   .*:*** :*   *:* ** :: :.  .**   *.      336


  HmenM_MCOX2       319 GVVEWLVWLVEAPYDALGFGYSYVVMLAKGFGATIFEFPYLVAGKACK  366
  CmonM_MCOX2       317 GVIEWGWWFLMSPREAGS----YAVTKVDGWV--DFLFTTILVGPVKA  358

  cons              337 **:**  *:: :* :* .    *.*  ..*:    * *. ::.* .    384
```

```
HmenM_MCOX2      367 VVHLMGTAFVYSYLSAIWFVGFLVSCAGSGGSAVGLFVGESFFKAIMA 414
CmonM_MCOX2      359 T------------WNAFGTIGSIIK------------------------ 371

cons             385 .             .*:  :* ::.                        432


HmenM_MCOX2      415 SGFGAKGSGLTSKYSGFSGVMG---ESVLSKAEFIKYKKCGVNLFKP- 458
CmonM_MCOX2      372 ----SAGSGLMHLIESLMGEMGGPDESATKRAVSEEVRVQMVRFFRVM 415

cons             433     : ****    ..: * **   **. .:*   : :   *.:*:   480


HmenM_MCOX2      459 ------L 459
CmonM_MCOX2      416 VSRYRGD 422

cons             481         487
```

C

```
T-COFFEE, Version_11.00.8cbe486 2014-08-12 22:05:29 - Revision 8cbe486 - Build 477
 Cedric Notredame
 CPU TIME:0 sec.
 SCORE=56
 *
  BAD AVG GOOD
 *
 HmenF_FORF    :  56
 CmonF_FORF    :  56
 cons          :   5

 HmenF_FORF       1 VSLTIKKPSLSSPKNPMIIMAALLTL-LLITIILLYLMSHG---QDST  44
 CmonF_FORF       1 IAI--------------------MTLIILIPLSYLPLIWSNTDNLKTA  28

 cons             1 :::                    :** :**.:  * *:  .    .::  48


 HmenF_FORF      45 TSLTITSMDITDMTSENLQTKGTNPQQNDTPT-------GHTPHKSKA  85
 CmonF_FORF      29 NNLKMKPIAHDLKPSKHPTSNITKPQPNDTQTSNEHSPNTYKPKKSKA  76

 cons            49 ..*.:..:     .*::  :: *:** *** *        :.*:****  96


 HmenF_FORF      86 HTNLNTK--------  92
 CmonF_FORF      77 STNLTNDKPNATKEP  91

 cons            97  ***...         111
```

d

```
T-COFFEE, Version_11.00.8cbe486 2014-08-12 22:05:29 - Revision 8cbe486 - Build 477
  Cedric Notredame
  CPU TIME:0 sec.
  SCORE=42
  *
   BAD AVG GOOD
  *
  HmenM_MORF     :  42
  CmonM_MORF     :  42
  cons           :   4

  HmenM_MORF        1 MWGQNELHSMSDFVLEYSDFVCFLLLV-IISTWSVFARAAKLIGVSKA   47
  CmonM_MORF        1 MK--ATLCKVIEFVLDNG-WLCLFYFVLFMACSNVLWRVYKV------   39

  cons              1 *     * .: :***: . ::*:: :* :::  .*: *. *:         48

  HmenM_MORF       48 WETLKRYWFSLFVLWVGFEVFLYITWTCVYKLFTYLSDPIRGLFFLSM   95
  CmonM_MORF       40 --------------------------------RKGLY-----------   44

  cons             49                                  . *              96

  HmenM_MORF       96 VFGLLTFWALWLFFDGISTGGYFSLGEEVSAGSSTAKSGKGKGKVLKG  143
  CmonM_MORF       45 ------------------------------------------------   44

  cons             97                                                  144

  HmenM_MORF      144 GVVEGDLALGGEVTEGLSDDAGDDKTKKVNKEGTDSPKKVKKETKDKP  191
  CmonM_MORF       45 -------------------------------------KKVKRVIG---   52

  cons            145                                      ****:       192

  HmenM_MORF      192 KKVKKEVTGEPEEVEKKVMNKAKKVKKETADEPEKVKKEAGDKPKKVK  239
  CmonM_MORF       53 ------VIWKPTAVS-----------------------------VKIK   65

  cons            193       *  :*  *.                              *:*  240

  HmenM_MORF      240 KEPADKPKKVKKSATDEPEKVSKEAVSKPKKAKMESVGKPKKVKKEAM  287
  CmonM_MORF       66 KDKVEKPKVMEK---------------------------AKKGKKAAV   86

  cons            241 *: .:*** ::*                           .** ** *:  288

  HmenM_MORF      288 NKPEKVKVSGGATDKLESDGSDKGNKLQ  315
  CmonM_MORF       87 SG-KVGKKSGG-----------------   96

  cons            289 .  :  * ***                  316
```

e

```
T-COFFEE, Version_11.00.d625267 2016-01-11 15:25:41 - Revision d625267 - Build 507
 Cedric Notredame
 CPU TIME:0 sec.
 SCORE=38
 *
  BAD AVG GOOD
 *
 Atra_ATP8      :  38
 Atra_UR_21_9   :  38
 cons           :   3

 Atra_ATP8      IPQLSPMSWLLVFISVC--FLFFCWCLGLLGGVG-------------V
 Atra_UR_21_9   IEFCI-----SVMGTSCGLFLLFFLCLILFSSDSYKYGFFNLVSEKVV

 cons           *          *:  :  *  **:*   **  *:.. .             *


 Atra_ATP8      SMIL---LV---------------
 Atra_UR_21_9   SSIYNLLSVVLPPSPKKGKPKIKK

 cons           *  *     *
```

f

```
T-COFFEE, Version_11.00.d625267 2016-01-11 15:25:41 - Revision d625267 - Build 507
  Cedric Notredame
  CPU TIME:0 sec.
  SCORE=53
  *
   BAD AVG GOOD
  *
  HmenM_MORF    :  53
  Atra_UR_21_9  :  53
  cons          :   5

  HmenM_MORF         1 MWGQNELHSMSDFVLEYSDFVCFLLLVIISTWSVFARAAKLIGVSKAW    48
  Atra_UR_21_9       1 ----------IEFCISVMGTSCGLF-----------------------    15

  cons               1            :* :.  .   * *:                       48


  HmenM_MORF        49 ETLKRYWFSLFVLWVGFEVFLYITWTCVYKLFTYLSDPIRGLFFLSMV    96
  Atra_UR_21_9      16 ------------------------------------------------    15

  cons              49                                                  96


  HmenM_MORF        97 FGLLTFWALWLFFDGISTGGYFSLGEEVSAGSST-AKSGKGKGKVLKG   143
  Atra_UR_21_9      16 --LLFFLCLILFSSDSYKYGFFNLVSEKVVSSIYNLL------------    50

  cons              97   ** * .* ** ..  . *:*.* .*  ..*                 144


  HmenM_MORF       144 GVVEGDLALGGEVTEGLSDDAGDDKTKKVNKEGTDSPKKVKKETKDKP   191
  Atra_UR_21_9      51 -----------------------------SVVLPPSPKK---------    60

  cons             145                              .   . ****          192


  HmenM_MORF       192 KKVKKEVTGEPEEVEKKVMNKAKKVKKETADEPEKVKKEAGDKPKKVK   239
  Atra_UR_21_9      61 ------------------------------------------------    60

  cons             193                                                 240


  HmenM_MORF       240 KEPADKPKKVKKSATDEPEKVSKEAVSKPKKAKMESVGKPKKVKKEAM   287
  Atra_UR_21_9      61 -------------------------------------GKPK-IKK---    67

  cons             241                                      **** :**      288


  HmenM_MORF       288 NKPEKVKVSGGATDKLESDGSDKGNKLQ   315
  Atra_UR_21_9      68 ----------------------------    67

  cons             289                              316
```

g

```
T-COFFEE, Version_11.00.d625267 2016-01-11 15:25:41 - Revision d625267 - Build 507
  Cedric Notredame
  CPU TIME:0 sec.
  SCORE=67
  *
   BAD AVG GOOD
  *
  CmonM_MORF     :  67
  Atra_UR_21_9   :  67
  cons           :   6

  CmonM_MORF           1 MKATLCKVIEFVLDNGWLCLFYFVLFMACSNVLWRVYKVRKGLYKKV-    47
  Atra_UR_21_9         1 --------IEFCISVMGTSCGLFLLFFLCL-ILFSSDSYKYGFFNLVS    39

  cons                 1         *** :.    .   *:**: *  :*:   . : *::: *     48


  CmonM_MORF          48 KRVIGVIWKPTAVSVKIKKDKVEKPKVMEKAKKGKKAAVSGKVGKKSG    95
  Atra_UR_21_9        40 EKVVSSIYNLLSVVLP-PSPKKGKPKIKK-------------------    67

  cons                49 ::*:. *::  :* :   . *  ***: :                       96


  CmonM_MORF          96 G    96
  Atra_UR_21_9        68 -    67

  cons                97      97
```

h

```
T-COFFEE, Version_11.00.d625267 2016-01-11 15:25:41 - Revision d625267 - Build 507
  Cedric Notredame
  CPU TIME:0 sec.
  SCORE=56
  *
   BAD AVG GOOD
  *
  CmonM_UR_24_11    :  56
  Atra_UR_21_9      :  56
  cons              :   5

  CmonM_UR_24_11        1 V-VCLSS--VDCGVEVPLVLDGALYLKALFGAIREFFHENSLFLL   42
  Atra_UR_21_9          1 IEFCISVMGTSCG----------------------------LFLL   17

  cons                  1 :  .*:*   ..**                            ****   45


  CmonM_UR_24_11       43 IIAVLVCICVSAFMWVYERRKVFVKKVKRGLKGLWKSIKVDFSVG   87
  Atra_UR_21_9         18 FFLCLILFSSDS--YKYGFFNLVSEKV------------------   42

  cons                 46 ::   *: :. .:  : *   ::. :**                     90


  CmonM_UR_24_11       88 GSGWVRLGAMSMKVLKWFSRFCVRVLWLVTLFVLLSVFLG-----  127
  Atra_UR_21_9         43 ---------------------------VSSIYNLLSVVLPPSPKK   60

  cons                 91                            : ::: ****.*          135


  CmonM_UR_24_11      128 -------  127
  Atra_UR_21_9         61 GKPKIKK   67

  cons                136          142
```

i

```
T-COFFEE, Version_11.00.d625267 2016-01-11 15:25:41 - Revision d625267 - Build 507
  Cedric Notredame
  CPU TIME:0 sec.
  SCORE=45
  *
   BAD AVG GOOD
  *
  HmenF_FORF     :  45
  Atra_UR_21_9   :  45
  cons           :   4

  HmenF_FORF          1 VSLTIKKPSLSSPKNPMIIMAALLTLLLITIILLYLMSHGQDSTTSLT    48
  Atra_UR_21_9        1 IEFCISV------------MGTSCGLFLLFFLCLILFSSDSYKYGFFN    36

  cons                1 :.: *.             *.:  *:*: :: * *:* .. .   :.    48


  HmenF_FORF         49 ITSMDITDMTSENLQTKGTNPQQNDTPTGHTPHKSKAHTNLNTK    92
  Atra_UR_21_9       37 LVSEKVVS--------SIYNLLSVVLPP--SPKKGKPKI---KK    67

  cons               49 :.* .:..        .  *  .   *.  :*:*.*.:    .*    92
```

1

```
T-COFFEE, Version_11.00.d625267 2016-01-11 15:25:41 - Revision d625267 - Build 507
  Cedric Notredame
  CPU TIME:0 sec.
  SCORE=70
  *
   BAD AVG GOOD
  *
  CmonF_FORF     :  70
  Atra_UR_21_9   :  70
  cons           :   7

  CmonF_FORF            1 IAIM-----TLIILIPLSYLPLIWSNTDNLKTAN-NLKMKPIAHDLKP    42
  Atra_UR_21_9          1 IEFCISVMGTSCGLFLLFFLCLILFSSDSYKYGFFNLVSEKVVSS---    45

  cons                  1 * :      *   *: * :* **  .:*. * .  **  : :. .       48


  CmonF_FORF           43 SKHPTSNITKPQPNDTQTSNEHSPNTYKPKKSKASTNLTNDKPNATKE    90
  Atra_UR_21_9         46 -----------IYNLLSVVLPPSPKKGKPKIKK---------------    67

  cons                 49              *  ..    **:. *** .*                  96


  CmonF_FORF           91 P    91
  Atra_UR_21_9         68 -    67

  cons                 97      97
```

```
m
T-COFFEE, Version_11.00.d625267 2016-01-11 15:25:41 - Revision d625267 - Build 507
  Cedric Notredame
  CPU TIME:0 sec.
  SCORE=58
  *
   BAD AVG GOOD
  *
  Atra_ATP8        :  58
  Atra_UR_22_18    :  58
  cons             :   5

  Atra_ATP8        IPQLSPMSWLLVFISVCFLFFCWCLGLLGGVGVSMILLV
  Atra_UR_22_18    MI-VGVLSWYVCLFFVCLS-----LFVS-----------

  cons             :  :. :** :  :: **:      * :
```

n

```
T-COFFEE, Version_11.00.d625267 2016-01-11 15:25:41 - Revision d625267 - Build 507
  Cedric Notredame
  CPU TIME:0 sec.
  SCORE=94
  *
   BAD AVG GOOD
  *
  Atra_UR_21_9     :  94
  Atra_UR_22_18    :  94
  cons             :   9

  Atra_UR_21_9     IEFCISVMGTSCGLFLLFFLCLILFSSDSYKYGFFNLVSEKVVSS
  Atra_UR_22_18    -----MIVGVLSWYVCLFFVCLSLFVS------------------

  cons                  ::*. .  .  ***:**  **  *


  Atra_UR_21_9     IYNLLSVVLPPSPKKGKPKIKK
  Atra_UR_22_18    ----------------------

  cons
```

```
 O
T-COFFEE, Version_11.00.d625267 2016-01-11 15:25:41 - Revision d625267 - Build 507
 Cedric Notredame
 CPU TIME:0 sec.
 SCORE=61
 *
  BAD AVG GOOD
 *
 Atra_UR_22_18    :   61
 HmenM_MORF       :   61
 cons             :    6

 Atra_UR_22_18    ---------------------------------------------
 HmenM_MORF       MWGQNELHSMSDFVLEYSDFVCFLLLVIISTWSVFARAAKLIGVS

 cons


 Atra_UR_22_18    ---------------------------------------------
 HmenM_MORF       KAWETLKRYWFSLFVLWVGFEVFLYITWTCVYKLFTYLSDPIRGL

 cons


 Atra_UR_22_18    ----MIVGVLS-WYVCLFFVCLSLFVS------------------
 HmenM_MORF       FFLSMVFGLLTFWALWLFFDGISTGGYFSLGEEVSAGSSTAKSGK

 cons                 *:.*:*: * :  ***  :*


 Atra_UR_22_18    ---------------------------------------------
 HmenM_MORF       GKGKVLKGGVVEGDLALGGEVTEGLSDDAGDDKTKKVNKEGTDSP

 cons


 Atra_UR_22_18    ---------------------------------------------
 HmenM_MORF       KKVKKETKDKPKKVKKEVTGEPEEVEKKVMNKAKKVKKETADEPE

 cons


 Atra_UR_22_18    ---------------------------------------------
 HmenM_MORF       KVKKEAGDKPKKVKKEPADKPKKVKKSATDEPEKVSKEAVSKPKK

 cons


 Atra_UR_22_18    ---------------------------------------------
 HmenM_MORF       AKMESVGKPKKVKKEAMNKPEKVKVSGGATDKLESDGSDKGNKLQ

 cons
```

p

```
T-COFFEE, Version_11.00.d625267 2016-01-11 15:25:41 - Revision d625267 - Build 507
  Cedric Notredame
  CPU TIME:0 sec.
  SCORE=57
  *
   BAD AVG GOOD
  *
  Atra_UR_22_18   :  57
  CmonM_MORF      :  57
  cons            :   5

  Atra_UR_22_18   MIVGV-------L-SWYVCLFFVCLSLFVS---------------
  CmonM_MORF      MKATLCKVIEFVLDNGWLCLFYFVLFMACSNVLWRVYKVRKGLYK

  cons            *  . :       *  .  ::***:.  *  :   *


  Atra_UR_22_18   ---------------------------------------------
  CmonM_MORF      KVKRVIGVIWKPTAVSVKIKKDKVEKPKVMEKAKKGKKAAVSGKV

  cons


  Atra_UR_22_18   ------
  CmonM_MORF      GKKSGG

  cons
```

q

```
T-COFFEE, Version_11.00.d625267 2016-01-11 15:25:41 - Revision d625267 - Build 507
 Cedric Notredame
 CPU TIME:0 sec.
 SCORE=72
 *
  BAD AVG GOOD
 *
 Atra_UR_22_18     :  72
 CmonM_UR_24_11    :  72
 cons              :   7

 Atra_UR_22_18      ---------------------------------------------
 CmonM_UR_24_11     VVCLSSVDCGVEVPLVLDGALYLKALFGAIREFFHENSLFLLIIA

 cons


 Atra_UR_22_18      ---------------------------------------------
 CmonM_UR_24_11     VLVCICVSAFMWVYERRKVFVKKVKRGLKGLWKSIKVDFSVGGSG

 cons


 Atra_UR_22_18      ------MIVGVLSWYV---------CLFFVCLSLFVS
 CmonM_UR_24_11     WVRLGAMSMKVLKWFSRFCVRVLWLVTLFVLLSVFLG

 cons                     * :  **.*:            :**  **:*:.
```

QUICK2D OUTPUT

Legend

```
SS = Secondary Structure ('H' = Alpha-Helix, 'E' = Beta-Sheet)
CC = Coiled Coils
TM = Transmembrane (= 'X') ('+' = outside, '-' = inside)
DO = Disorder (= 'D')
SP = Signal Peptide ('S' = signal peptide, 'C' = Cleavage Site)
```

• ***Hyridella menziesii*** **F**

F-*ORF* (92 aa)

```
QUERY                 VSLTIKKPSLSSPKNPMIIMAALLTLLLITIILLYLMSHGQDSTTSLTITSMDITDMTSENLQTKGTNPQQNDTPTGHTP
SS PSIPRED              EEE           HHHHHHHHHHHHHHHHHHHHHH       EEEEEEEEHHHHHHHHH
CONF                  91153477768999835699999999999999999996377775137888630012024554037899565789899787
SS JNET                              HHHHHHHHHHHHHHHHHHHHHH        EEEEEEE
CONF                  41663167888988620814667888878899998732588875126872144640444100004588888888866667
SS PROF (Ouali)          EEE          HHHHHHHHHHHHHHHHHHHHHH       EEEEEEEE   H HHHH
CONF                  86456457788888865788888888888888888875578876478888764444445665457778888888888887
SS PROF (Rost)         EEEEE         HHHHHHHHHHHHHHHHHHHHHHH       EEEEEEEEE
CONF                  92566436657766678888888878788888888875066676236775201111331000135767765777766666
TM HMMTOP             -----------------XXXXXXXXXXXXXXXXXXXX+++++++++++++++++++++++++++++++++++++++++++
CONF
SP PREDISI            SSSSSSSSSSSSSSSSSSSSSSSSSSSSSSSSSSSSSSSC
SP HMMTOP
TM PHOBIUS            ++++++++++++++++XXXXXXXXXXXXXXXXXXXX--------------------------------------------
CONF
TM PROF (Rost)        ------------------XXXXXXXXXXXXXXXXXX++++++++++++++++++++++++++++++++++++++++++++
CONF                  99999999999998862256788888888888776531567889999999999999999999999999999999999999
DO IUPRED                DDD                                         DDD      DDDDDDDDDDDDDDDDDDDDDDDD
CONF

QUERY                 HKSKAHTNLNTK
SS PSIPRED
CONF                  633101265779
SS JNET
CONF                  665113467877
SS PROF (Ouali)
CONF                  776555666778
SS PROF (Rost)
CONF                  533222234768
TM HMMTOP             ++++++++++++
CONF
SP PREDISI
SP HMMTOP
TM PHOBIUS            ------------
CONF
TM PROF (Rost)        ++++++++++++
CONF                  999999999999
DO IUPRED             DDDDDDDDDDDD
CONF
```

• ***Cumberlandia monodonta* F**

F-*ORF* (91 aa)

```
QUERY            IAIMTLIILIPLSYLPLIWSNTDNLKTANNLKMKPIAHDLKPSKHPTSNITKPQPNDTQTSNEHSPNTYKPKKSKASTNL
SS PSIPRED        HHHHHHHHHHHHHHHHHHH    HHHH
CONF             90678899999876354221563132102233454457767778864346788987412346789765686534234334
SS JNET             HHHHHHHHHHHHHHHH     HHHH
CONF             89999999887322011324776156653201223357888888887678888888877778888888778887777766
SS PROF (Ouali)   HHHHHHHHHHHHHHHHHHH      HHH
CONF             56666777788877656654567655444456776667888888887777778888777777888887788776666566
SS PROF (Rost)    EEEEEEEEE       EEE    HHHH
CONF             82334556641221431220353403441445552002457665775565467877654566757675455635323245
TM HMMTOP        ++++++++++++++++++++++++++++++++++++++++++++++++++++++++++++++++++++++++++++++++
CONF
SP PREDISI       SSSSSSSSSSSSSSSSSSSC
SP HMMTOP
DO IUPRED                                   DDDDDDDDDDDDDDDDDDDDDDDDDDDDDDDDDDDDDDDDDDDDDDDDDDDDD
CONF

QUERY            TNDKPNATKEP
SS PSIPRED
CONF             45887666788
SS JNET
CONF             56889888888
SS PROF (Ouali)
CONF             67788877788
SS PROF (Rost)
CONF             56776557678
TM HMMTOP        +++++++++++
CONF
SP PREDISI
SP HMMTOP
DO IUPRED        DDDDDDDDDDD
CONF
```

• ***Hyridella menziesii* M**

COX2 (459 aa)

```
QUERY            MSLWGQLGLQDGLSSAALEIGSLHDHAMAIMVLIFSFVGFVFFKVVLEEGGCTTMLDSQWVEFLWTSLPLGLLIMLGLPS
SS PSIPRED                     HHHHHHHHHHHHHHHHHHHHHHHHHHHHHHHHHH          EEEEEEEEEHHHHHHHHHHHHH
CONF             98631267988537889999999999999776566774654544443333678766798489987440678999999999
SS JNET                        HHHHHHHHHHHHHHHHEEHHHHHHHHHHHHHHHH           EEEEEEEEHHHEEEEEEE  H
CONF             34774228775326899999999999888876543335234567767641788888888607888886234331001 2124
SS PROF (Ouali)    EEEEEE      HHHHHHHHHHH  EEEEEEEEEEEEEEEEEEEEE            EEEEEEEE EEEEEEEEE
CONF             83555544467764467777776543357888888887777777765446777777777568888765456788876443
SS PROF (Rost)       EE     HHHHHHHHHHHHHHHHHHHHHHHHHHEEEEEEEEEEE    EEEE HHHHHHHHHH  HHHHHH
CONF             9110001410003457775420446788877763102047788875147713432103567667420000332105532
TM HMMTOP        ++++++++++++++++++++++++++XXXXXXXXXXXXXXXXXXXX----------------XXXXXXXXXXXXXXXXXX
CONF
SP PREDISI
SP HMMTOP
TM PHOBIUS       --------------------------XXXXXXXXXXXXXXXXXXXXX+++++++++++++++++++XXXXXXXXXXXXXX
CONF
TM PROF (Rost)   -------------------------XXXXXXXXXXXXXXXXXX++++++++++++++++++++++XXXXXXXXXXXXXXX
CONF             99999999999999999998765315677888887788887765312577788888998776403456778887777777

QUERY            IRLLYLLDDPADALISMKVLGRQWYWTYEYSDFIGGWGDEPIYFDSYMMSSVDLSDGGYRLLEVDNRCVVPYGVGVRVLV
SS PSIPRED       HHHHHH         EEEEEEEEEEEEEE            EEEE     HHH             EEEEE   EEEEEE
CONF             99886233557777339998766899997256666557664057402045001145665433447528973798389999
SS JNET          HHHHHHH       EEEEEE  EEEEEE                                      EEEE     EEEEE
CONF             6546530267898810687454515772504788877664266205666555667888710178637866156218998
SS PROF (Ouali)  EEEEEEE        EEEEEEEEEEEEEEE            EEEEE                    EEEEE   EEEEE
CONF             34555544677776647888777777776457778777777456664567776677777776777765777566568888
SS PROF (Rost)   EEEEEE   HHHHHHHHHHH  EEEEEEEEE          EEEEEEEEEEEE     EEEEEE   EEEE   EEEEEE
CONF             03543226825566556662221257775300035576520343232545651777257787516067520312577777
TM HMMTOP        X+++++++++++++++++++++++++++++++++++++++++++++++++++++++++++++++++++++++++++++++
CONF
SP PREDISI
SP HMMTOP
TM PHOBIUS       XXXXXX--------------------------------------------------------------------------
CONF
TM PROF (Rost)   XXX-----------------------------------------------------------------------------
CONF             65432115655666789998898999999999999999999998888999999999999999999998753200011331

QUERY            SSSDVVHSWAVPSLGVKVDSIPGRINQVILSLVGSGVVYGQCSELCGVNHSFMPICLEAVRSDVYGLWVSPPVAEEVSEA
SS PSIPRED       EE HHHHH    HH  EE      EEEEEEEE    EEEEE HHHH       EEEEEEE HHHHHHHHHHHHHHHHHHH
CONF             96513330132032553115798257899995798169998677747061488169999889999999723344678888
SS JNET          E   EEEEEE                EEEEEE     EE              EEEEEE     HHEHH    HHHHH
CONF             42511003563103652221578534478887077658966656627788850178998360677797643655034665
SS PROF (Ouali)  EE   EEEEEEE   EEEEE      EEEEEEE    EEEEEEEE        EEEEEEE   HHHHHHH       HH
CONF             74564345555433344555456665468887467755666654335667765578888746456777654677654444
SS PROF (Rost)   E    EEEEEE    EEEE    EEEEEEEEEE   EEEEEEE        HHHHHHHHHHH  EEEEE   HHHHHHHH
CONF             23670676432234524512133010256565167168764201013343201566666541230244155068888899
TM HMMTOP        ++++++++++++++++++++++++++++++++++++++++++++++++++++++++++++++++++++++++++++++++
CONF
SP PREDISI
SP HMMTOP
TM PHOBIUS       --------------------------------------------------------------------------------
CONF
TM PROF (Rost)   ---------------------XXXXXXXXXXXXXXXXXX+++++++++++++++++++++++++++++++++++++++++
CONF             01134564456788788777654520135555432100356778888887543223342255656888899999999999

QUERY            VKAFDGEGESVGWWGWLIGKVMKAMWVVGNGLMLVGITFVKVYMGWWVFFFNVFIYAPVELFIGFLFSVSWWSFSLLTGV
SS PSIPRED       HHHH        EEHHHHHHHHHHEEEEEEEEEEHHHHHHHHHHHHHHHHEEEEEEEEEEEEEEEEEEEEEEEEEEEEEE
CONF             87526689731100122888764204651020023767765788876311689999748999982476788753205113
SS JNET                              EEEEEEE    EEE  EEEHHHHHHHHHHEEEEEEEEEEEEEEE EEEEEEEE    EEE
CONF             42157877641134566666543102101223334566635553422002047898525777872355577765300013
SS PROF (Ouali)  HHH        EEEEEEEEEEEEEEEEEEEEEEEEEEEE   EEEEEEEEEEEEEEEEEEEEEEEE EEEEEEEEEEEEE
CONF             44347888764445555665556777776677776554454577666665456777666778764446777765444445
SS PROF (Rost)   HHHH        HHHHHHHHHHHHHH     EEEEEEEEEEEEEEEEEEEEEEEEEE  EEEEEEEEEEEHHHHHHHHHH
CONF             77523776320235788888765521002331456578877653124544545786120003210233303456677535
TM HMMTOP        ++++++++++++++++++++++++++++++++++++++++++++++++++++++++++++++++++++++++++++++++
CONF
SP PREDISI
SP HMMTOP
TM PHOBIUS       ------------------------------XXXXXXXXXXXXXXXXXXXXXXXX-+++++XXXXXXXXXXXXXXXXXXXX
CONF
TM PROF (Rost)   ++++++++++++++++++++++++++++XXXXXXXXXXXXXXXXXXXXXXXXX----XXXXXXXXXXXXXXXXXXXXXXX
CONF             99999999998632012334566677777777777777777777777777777777777777777777777777777777
```

```
QUERY              VEWLVWLVEAPYDALGFGYSYVVMLAKGFGATIFEFPYLVAGKACKVVHLMGTAFVYSYLSAIWFVGFLVSCAGSGGSAV
SS PSIPRED         EEEEEEEE      EEEEEEHHHHHH     EEEE HHHH    EEEEEE HHHHHHHHHHHHHHHHHHEEE      EE
CONF               45787875386543434521021113346736875003123652036775004899999999998865213037888347
SS JNET            E  EEEEE    HHHHHHHHHHHHHHH    EEEH HHHH    EEEEEEE  EEEHHHHHHHEEEEEEEEE      EE
CONF               32234677148640221102354320077733003001010652113220014788888777663014688726888043
SS PROF (Ouali)    EEEEEEEEE      EEEEEEEEEEEE    EEEEE EEEE    EEEEEE   HHHHHHHHHHEEEEEEEEE      E
CONF               66766777646766456666777776456764676533344466467887557567777766653566787755777646
SS PROF (Rost)     HHHHHHHHH  HHHH   EEEEEEEE    EEEEEE HHH     EEEEEE HHHHHHHHHHHHHHEEEEEE      EE
CONF               77654331011444056413354421045505774011110321036775213456767766663101445307666056
TM HMMTOP          ++++++++++++++XXXXXXXXXXXXXXXXXXXX--------------------XXXXXXXXXXXXXXXXXXXX++++++
CONF
SP PREDISI
SP HMMTOP
TM PHOBIUS         XXXXX-----------XXXXXXXXXXXXXXXXXXXXXXX--+++++++++++++++++++XXXXXXXXXXXXXXXXXXXX
CONF
TM PROF (Rost)     XX+++++++++++++XXXXXXXXXXXXXXXXXXXXXXXXX---------XXXXXXXXXXXXXXXXXXXXXXXX++++XXX
CONF               77765432000024566667777766555555566777776532012456777777777777777777777776655555

QUERY              GLFVGESFFKAIMASGFGAKGSGLTSKYSGFSGVMGESVLSKAEFIKYKKCGVNLFKPL
SS PSIPRED         EEEE HHHHHHHHH                     HHHHHHHHHHHHHHH
CONF               88731888999986046767776531245635200154540555410131350020579
SS JNET            EEEE HHHHHHHHHH         E           HHHHHHHHHHHHH
CONF               04403257888752356656776311213531220034576677211213453117888
SS PROF (Ouali)    EEEE  HHHHHHHHH         EEEEE       HHH   HHHEEEEE    EEE
CONF               78865557787654367765776445554555455544335444445555466545468
SS PROF (Rost)     EEE  HHHHHHHHHH         EEE         HHHH HHHHHHHHH
CONF               75401578888775113543353012102220001143422566300110243112568
TM HMMTOP          +++++++++++++++++++++++++++++++++++++++++++++++++++++++++++
CONF
SP PREDISI
SP HMMTOP
TM PHOBIUS         X----------------------------------------------------------
CONF
TM PROF (Rost)     XXXXXXXXXXXXXXX--------------------------------------------
CONF               66666555665542000137778888999988999998899999999999999999999
```

M-*ORF* (315 aa)

```
QUERY              MWGQNELHSMSDFVLEYSDFVCFLLLVIISTWSVFARAAKLIGVSKAWETLKRYWFSLFVLWVGFEVFLYITWTCVYKLF
SS PSIPRED             HHHHHHHHHHHHHHHHHHHHHHHHHHHHHHHHHHHHHHHHHHHHHHHHHHHHHHHHHHHHHHHHHHHHHHHHHHHH
CONF               96510124367788875418999999999888999999988616889999999999999998546654567999999999
SS JNET                        HEEEH HHHHEEEEEEE   HHHHHHHHHHHHHHHHHHHHHHHHHHHEEE  EEEEEEEEEEEEEEEE
CONF               77766433210364112114666544311117567877764622412678887777641566212758888675046665
SS PROF (Ouali)              HHHHHH    HEEEEEEEE  HHHHHHHHHHH   HHHHHHHHHHHHHEEEEEE  EEEEEEEEHHHHHH
CONF               85433333343445433334434555555444788888888765656788888876534666535467888765356677
SS PROF (Rost)                  EEEE HHHEEEEEEEEEHHHHHHHHHHH   HHHHHHHHHHHHHEEEEEE EEEEEEEEEEEHHHHH
CONF               965332110000011210222023443343126688888875264178988877653103554301456777531012231
TM HMMTOP          +++++++++++++++++++XXXXXXXXXXXXXXXXX------------------XXXXXXXXXXXXXXXXXXX+++++++
CONF
SP PREDISI         SSSSSSSSSSSSSSSSSSSSSSSSSSSSSSSSSSSSSC
SP HMMTOP
TM PHOBIUS         -------------------XXXXXXXXXXXXXXXXXXXXXXX+++++++++++XXXXXXXXXXXXXXXXXXXXXXX----
CONF
TM PROF (Rost)     ++++++++++++++++++++XXXXXXXXXXXXXXXXXX------------------XXXXXXXXXXXXXXXXXXXXXX++
CONF               99999999999999887621467788888888877776652231014323234403567778887777777777776665
DO IUPRED
CONF

QUERY              TYLSDPIRGLFFLSMVFGLLTFWALWLFFDGISTGGYFSLGEEVSAGSSTAKSGKGKGKVLKGGVVEGDLALGGEVTEGL
SS PSIPRED         HHH   HHHHHHHHHHHHHHHHHHHHHHH            HHHH
CONF               87233036789999999999999999716766677731123665426654447776776567763015645566545566
SS JNET            EE       EEHHHHHHHHHHHHHHHHHE      EEEEEEEEEE
CONF               32477622135878888765543776304377654534311012134787888888883378730267787888887888
SS PROF (Ouali)    HHH      HHHHHHHHHHHHHHHHHHEE        EEE  EHH
CONF               6544675433467887767777666444455677654444333334565445777777655565444655677544556
SS PROF (Rost)     H     HHHHHHHHHHHHHHHHHHHHHHH       EE    EEE             EEEE  EEEEEE    EEEE
CONF               00025301033356887778888888775243344310035133223100124677732675032555310365022113
TM HMMTOP          +++++++++XXXXXXXXXXXXXXXXXXXX---------------------------------------------------
CONF
SP PREDISI
SP HMMTOP
TM PHOBIUS         -------XXXXXXXXXXXXXXXXXXXXX-+++++++++++++++++++++++++++++++++++++++++++++++++++
CONF
TM PROF (Rost)     +++++++++++XXXXXXXXXXXXXXXXXX---------------------------------------------------
CONF               54431001456777888777777777777776542103688999999999999999999999999999999999999999
DO IUPRED
CONF
```

```
QUERY                 SDDAGDDKTKKVNKEGTDSPKKVKKETKDKPKKVKKEVTGEPEEVEKKVMNKAKKVKKETADEPKVKKEAGDKPKKVKK
SS PSIPRED
CONF                  7654321134323445677654323334444433233344421113432233443323222454433344555555 4444
SS JNET
CONF                  8888888888888888888888888888888888888888888888888888888888888888888888888888888
SS PROF (Ouali)
CONF                  6666665444555556777765555555655556655667765566555566666665556666656776777777 6666
SS PROF (Rost)                           HHHHHHHH       EEEE  HHHHHHHHHHHHHHH HHH   HHHHHHHH
CONF                  4531454010002457887455323320686412023136668998888878754012102846788767357853 0203
TM HMMTOP             -------------------------------------------------------------------------------
CONF
SP PREDISI
SP HMMTOP
TM PHOBIUS            +++++++++++++++++++++++++++++++++++++++++++++++++++++++++++++++++++++++++++++++
CONF
TM PROF (Rost)        -------------------------------------------------------------------------------
CONF                  9999999999999999999999999999999999999999999999999999999999999999999999999999999
DO IUPRED              DDDDDDDDDDDDDDDDDDDDDDDDDDDDDDDDDDDDDDDDDDDDDDDDDDDDDDDDDDDDDDDDDDDDDDDDDDDDDD
CONF

QUERY                 EPADKPKKVKKSATDEPEKVSKEAVSKPKKAKMESVGKPKKVKKEAMNKPEKVKVSGGATDKLESDGSDKGNKLQ
SS PSIPRED                                                                                HHH
CONF                  422345555555311233334454454433455555566555555565433445456566666654210120159
SS JNET
CONF                  888888888888888888888888888888888888888888888888888888888888888883201011223
SS PROF (Ouali)
CONF                  666777767776666666656666777766666666667776677666677655555455677776555555555678
SS PROF (Rost)                        HHHH HHHH     EEEE    HHHHHHH      EEEE
CONF                  633342101115577444411665246310023205872353356507876146613533221223343444358
TM HMMTOP             ---------------------------------------------------------------------------
CONF
SP PREDISI
SP HMMTOP
TM PHOBIUS            +++++++++++++++++++++++++++++++++++++++++++++++++++++++++++++++++++++++++++
CONF
TM PROF (Rost)        ---------------------------------------------------------------------------
CONF                  999999999999999999999999999999999999999999999999999999999999999999999999999
DO IUPRED             DDDDDDDDDDDDDDDDDDDDDDDDDDDDDDDDDDDDDDDDDDDDDDDDDDDDDDDDDDDDDDDDDDDDDDDDDDD
CONF
```

• ***Cumberlandia monodonta* M**

COX2 (422 aa)

```
QUERY             VFSVEVLAGLSCLWTVSEMSLWGQFGFQEGATVLGIEISDLYDHVMFFSVLVFSFVGYLFCKMLMSVFTCRVLLEKQWLE
SS PSIPRED         HHHHHHHHHHHHHHHHHHHHHH         HHHHHHHHHHHHHHHHHHHHHHHHHHHHHHHHHHH          EEE
CONF              93889999999999877776333214799967778999999999999989999999998877654412467777884688
SS JNET              HHHHHHHHHHHHHHHHHH           H HHHHHHHHHHHHHHHHHHHHHHHHHHHHHHHHHHH        EEE
CONF              68899997611701133553244567767522177888887553040233210252677889987740547888886177
SS PROF (Ouali)    EEEEEEEEEHHHHH HHHHHH           HHHHHHHHHH EEEEEEEEEEEEEEEEEEEEEE            EE
CONF              75777766433444344444434557777665544566665534457888888777777776655445567777777568
SS PROF (Rost)     EEEEEEE   EEEEEEEEEEEEE       EEEEEEEE    EEEEEEEEEEEHHHHHHHHHHHHHHHHHHHHHHHHHH
CONF              84666652321356654223221004200214776787412241578775442112346655643133345575675788
TM HMMTOP         ++++++++++++++++++++++++++++++++++++++++++++XXXXXXXXXXXXXXXXX-------------------
CONF
SP PREDISI
SP HMMTOP
TM PHOBIUS        -----------------------------------++++++++XXXXXXXXXXXXXXXXXXXXXXXXXXXXX------XX
CONF
TM PROF (Rost)    +++++++++++++++++++++++++++++++++++++++++++XXXXXXXXXXXXXXXXXXXXXXXXX------------
CONF              99999999999999999998999999999998887543023335677777777777777777777776641003443232
DO IUPRED
CONF

QUERY             FIWTFLPAGVLLAIGLPSVHLLYVMEDVGAPQITVKAIGHQWYWSYEYCDLGGEDSISFDSYMVPSVDLVVGSYRLLDVD
SS PSIPRED        EEEEHHHHHHHHHHHHHHHHHHHHHH       EEEEEEEEEEEEEEE          EEEEE
CONF              77156899999999999999987520356543068865579999972356777654100231346654566555444447
SS JNET           EEEEEHHHHHHHHHHHHHHHHHHHH      HHHHHHHHHHHHHHHH
CONF              87643248865885422123100002456765226777656677764157777676123213215777777777777788
SS PROF (Ouali)   EEEEEEEEEEEEEEEEE  EEEEE          EEEEEEEEEEEEE           EEEEE
CONF              88877643466666544444555435677777755788765666665467777777645676557777777777777777
SS PROF (Rost)    HHHHHH    EEEE    EEEEEEEEE    EEEEEEEEEEEEEEEEEE      EEEEE EEEEEEEEEEEEEEEEE
CONF              88865010002465166036888754157752556441112577777623686412334001331244677567777315
TM HMMTOP         -XXXXXXXXXXXXXXXXXX+++++++++++++++++++++++++++++++++++++++++++++++++++++++++++++
CONF
SP PREDISI
SP HMMTOP
TM PHOBIUS        XXXXXXXXXXXXXXXXXXXXX-++++++++++++++++++++++++++++++++++++++++++++++++++++++++++
CONF
TM PROF (Rost)    --XXXXXXXXXXXXXXXXXXXX++++++++++++++++++++++++++++++++++++++++++++++++++++++++++
CONF              03567778887777777777664205666678999998889999999999999999999999988766678898877889
DO IUPRED
CONF

QUERY             NRFVVPYGVPMRVLVSSSDVIHSWAIPSAGVKDGVVGRVNQAGLGFFGPGVVYGQCSELCGVNHSFMPICGEVVSCEAY
SS PSIPRED         EEE     EEEEEEEE         HHH  EEE     EEEEEEEE   EEEEEE HHH    HH  EEEEEEE HHHH
CONF              62882279889999994632341054000754424698156899993787389998856518122168679999889999
SS JNET             EEE     EEEEEE   EEEEEE                EEEEEE      E  HEEH         EEEEE     H
CONF              70786514625999970420002452002564201278245899984045368977706617788761268998471457
SS PROF (Ouali)     EEEE    EEEEEEE   EEEEEE    EEEEEE     EEEEEEE    EEEEEEEE        EEEEEEE   HH
CONF              75566556647888874675455554443344555456665578887567746776654435666665478888746456
SS PROF (Rost)     EEEEE   EEEEEEE   EEEEEEE    EEEEEEEEEEE     EE   EEEEEEE       EE     EE  HHHH
CONF              50786204315788713550564333246715732786651021202056157644100001231000110011115677
TM HMMTOP         ++++++++++++++++++++++++++++++++++++++++++++++++++++++++++++++++++++++++++++++++
CONF
SP PREDISI
SP HMMTOP
TM PHOBIUS        ++++++++++++++++++++++++++++++++++++++++++++++++++++++++++++++++++++++++++++++++
CONF
TM PROF (Rost)    ++++++++++++++++++++++++++++++++++++++++++++++++++++++++++++++++++++++++++++++++
CONF              99876432221021010034320012345447778899999999888888766677788888887533211002443222
DO IUPRED
CONF
```

```
QUERY            ALWLLPKNCPAGKSIWDYCLYWGGLLWWGCGRVAYWTSFAYLGWWKIFGYYFVYMPVKVSVDTTVSVVEGSVRSCCGVIE
SS PSIPRED       HHHHHH            EEEE  EEHHHHHHHHEEHHHHHHHHHHHHEEEEEEEEEEEEEEEEEEEEEEEEEHEHHHEE
CONF             99987376450035566316512111000110023056679887752088999984799999667567664122101100
SS JNET          HHHHHH           HHEEH  EEEEEHH HHHHHHEEEEEHHHHHEEEEEE   EEEEEEHHHHHHHHHH  EEEEE
CONF             78877256777777363134542125665422346521456771052104899852488998445546665300110011
SS PROF (Ouali)  HHHHH              EEEEEEEEEEEEEEEEEEEEEEEEEEEEEEEEEEEEEEEEEEEE EEEEEEEEEEEEEEEE
CONF             66654367777766767645554467777655567775445655556655677775457787644467766555555566
SS PROF (Rost)   HHHHH         HHHHHHHHH  EEE    EEEEHHHHHHHHHHHEEEEEEEEEEEEEEEEEEEEEEEEEEE  EEEE
CONF             67540234766643356765331001101440243113443212222025676631366650167776424432011354
TM HMMTOP        ++++++++++++++++++++++++++++++++++++++++++++++++++++++++++++++++++++++++++++++++
CONF
SP PREDISI
SP HMMTOP
TM PHOBIUS       ++++++++++++++++++++++++++++++++++++++++++++++++++++++++++++++++++++++++++++++++
CONF
TM PROF (Rost)   +++++++++++++XXXXXXXXXXXXXXXXXX----XXXXXXXXXXXXXXXXXX+++++++++++++++++++++++++++
CONF             01434678776777541011224666555445666777777777777777777766410123788889988999875432
DO IUPRED
CONF

QUERY            WGWWFLMSPREAGSYAVTKVDGWVDFLFTTILVGPVKATWNAFGTIGSIIKSAGSGLMHLIESLMGEMGGPDESATKRAV
SS PSIPRED       EEEEEE   HHHHEEEEEEE  EEEEEEEEEE   EEEEEEEE  EEEEEEEEEE HHHHHHHHHHH        HHHHH
CONF             22011358022126888899026879999998276489989300651699999950566489999752588875444563
SS JNET          EEEEEEE      EEEEEEEEEEEEEEEEEEE    EEHEEEEEEEEEEEEEEEE  EEEEEEEEEEE E      EEEE
CONF             12577742654303578888544035889997324477888774222788888715367740344126777888462566
SS PROF (Ouali)  EEEEEEE      EEEEEEEE  EEEEEEEEEEE  EEEEEEEE EEEEEEEEEE   EEEEEEHHH          EEE
CONF             66677764675555677888645467888888747647777665446788888765655665434433466777666478
SS PROF (Rost)   EEEEEEE       EEEEEE  EEEEEEEEEEEE EEEEHHHH HHHHHHHHHHHHHHHHHHHHHHH     HHHHHHHH
CONF             20024421210154267863331433466887610021102210124544431124687888785213688744555543
TM HMMTOP        ++++++++++++++++++++++++++++++++++++++++++++++++++++++++++++++++++++++++++++++++
CONF
SP PREDISI
SP HMMTOP
TM PHOBIUS       ++++++++++++++++++++++++++++++++++++++++++++++++++++++++++++++++++++++++++++++++
CONF
TM PROF (Rost)   ++++++++++++++++++++++++++XXXXXXXXXXXXXXXXXXXXXXXXX-----------------------------
CONF             21001236464457888888753322466777777655445555667777665431234689999999999999999999
DO IUPRED                                                                               D   D
CONF

QUERY            SEEVRVQMVRFFRVMVSRYRGD
SS PSIPRED       HHHHHHHHHHHHHHHHHH
CONF             6677760899999998860699
SS JNET          EEEE     EEEEEEE
CONF             6676223068888898408988
SS PROF (Ouali)  EEEEE   HHHHHHHHHHH
CONF             8887546555566656663688
SS PROF (Rost)    HHHHEEEEEEEEEEEEEEE
CONF             1532100243567666666258
TM HMMTOP        ++++++++++++++++++++++
CONF
SP PREDISI
SP HMMTOP
TM PHOBIUS       +++++++++++++++++++++-
CONF
TM PROF (Rost)   ----------------------
CONF             9999999999999999999999
DO IUPRED
CONF
```

M-*ORF* (96 aa)

```
QUERY            MKATLCKVIEFVLDNGWLCLFYFVLFMACSNVLWRVYKVRKGLYKKVKRVIGVIWKPTAVSVKIKKDKVEKPKVMEKAKK
SS PSIPRED           HHEEEEEEE    HHHHHHHHHHHHHHHHHHHHHHHHHHHHHHHHHEEEEE   EEEEEEE      HHHHHHHH
CONF             95320123576753956499999999997088999999987899987762488844637899974133437078898754
SS JNET               HEEEEEE     EEHHEEHHHHH   HHHEHHHHHHHHEEEEEEEEEEEE   EEEEEE         HHHHHH
CONF             47888888887644883136888776642112566677342546530362448883714677750565355152650650
SS PROF (Ouali)    H HHHHHHHEE     EEHHHHHHHHHHHHHHHHHHHHHHHHHHHH EEEEEEEE EEEEEEE       HHHHHHH
CONF             86434654444444677454455677765556766655554467664435677776454677764566778656777764
SS PROF (Rost)       HHHEEEEEE     HHHHHHHHHHHHHHHHHHHHHHHHHHHHH EEEEEEE   EEEEEEEE     HHHHHHHH
CONF             93202210002314640001243343355424552233554345430124655640342677663055005468777632
TM HMMTOP        ++++++++++++++++XXXXXXXXXXXXXXXXXX----------------------------------------------
CONF
SP PREDISI
SP HMMTOP
TM PHOBIUS       +++++++++++++++++++XXXXXXXXXXXXXXXXX--------------------------------------------
CONF
TM PROF (Rost)   +++++++++++++++XXXXXXXXXXXXXXXXXX-----------------------------------------------
CONF             99999999887420046778888888888877642015777778999998522211234447889999999999999999

QUERY            GKKAAVSGKVGKKSGG
SS PSIPRED         HHH
CONF             5500012544565799
SS JNET            EEEEE
CONF             6742542221568898
SS PROF (Ouali)     EEEEEEE
CONF             7865776544567788
SS PROF (Rost)      EEEE
CONF             8812574010003579
TM HMMTOP        ----------------
CONF
SP PREDISI
SP HMMTOP
TM PHOBIUS       ----------------
CONF
TM PROF (Rost)   ----------------
CONF             9999999999999999
```

CmonM_UR_24_11 (127 aa)

```
QUERY            VVCLSSVDCGVEVPLVLDGALYLKALFGAIREFFHENSLFLLIIAVLVCICVSAFMWVYERRKVFVKKVKRGLKGLWKSI
SS PSIPRED        EEE       EEEEEE  HHHHHHHHHHHHHHHH   HHHHHHHHHHHHHHHHHHHHHHHHHHHHHHHHHHHHHHHHHE
CONF             95632336575513665318999999999999981153899999999999999999999999999999987878768784
SS JNET               E      EHHHHHHHHHHHHHHHHHHHHHHH HHHHHHHHHHHHHHHHHHHHHHHHHHHEEEEHH       EEE
CONF             3776216333476775251155778887776661166222456778777662546654112564202442100102246
SS PROF (Ouali)  HEEEEEEE   EEEEEE   HHHHHHHHHHHHHH     EEEEHHHHHHHHHHHHHHHHHH HHHHHHHHHHHHHHHEEE
CONF             34443344355466565564678888888888864578656555678888765664555444455777665454543456
SS PROF (Rost)    EEEEE    EEEEEEE HHHHHHHHHHHHHHHHHHHHHHHHHHHHHEEEEEHHHHHHHHHHHHHHHHHHHHHHHHHHEE
CONF             86776224642555530358887888788887641201666655320122200312145555544677764331001103
TM HMMTOP        ++++++++++++++++++++++++++++++++++++++++XXXXXXXXXXXXXXXXXXX---------------------
CONF
SP PREDISI       SSSSSSSSSSSSSSSSSSSSSSSSSSSSSSSSSSSSSSSSSSSSSSSSSSSSSC
SP HMMTOP
TM PHOBIUS       ++++++++++++++++++++++++++++++++++++++XXXXXXXXXXXXXXXXXXXX----------------------
CONF
TM PROF (Rost)   +++++++++++++++++++++++++++++++++++++XXXXXXXXXXXXXXXXXX-------------------------
CONF             99999999999999988754554311222342023457788877877777888877652267889999999999999999

QUERY            KVDFSVGGSGWVRLGAMSMKVLKWFSRFCVRVLWLVTLFVLLSVFLG
SS PSIPRED       EEEEEE    EEEE HHHHHHHHHHHHHHHHHHHHHHHHHHHHHH
CONF             77387068634664277899999999999999999999999999709
SS JNET          EEEEE     HHHHHHHHHHHHHHHHHHHHHHHHHHHHHHHHH
CONF             77777168725774001648888873332011110012440156628
SS PROF (Ouali)  EEEEEEE    EEEE   HHHHHHHHHHHHHHHHHHHHHHHHHHH
CONF             78888756765577544457888877887767777888888776468
SS PROF (Rost)   EEEEEE   HHHHHHHHHHHHHHHHHHHHHHHHHHHHHHHHHHHH
CONF             56564166312112345478888986666655566766432100029
TM HMMTOP        ---------------------------XXXXXXXXXXXXXXXXXXX+
CONF
SP PREDISI
SP HMMTOP
TM PHOBIUS       --------------------------XXXXXXXXXXXXXXXXXXX-+
CONF
TM PROF (Rost)   ---------------------------XXXXXXXXXXXXXXXXXX++
CONF             99998888755551022123311222024677888888887775311
```

• ***Anodontites trapesialis***

Atra_UR_21_9 (67 aa)

```
QUERY             IEFCISVMGTSCGLFLLFFLCLILFSSDSYKYGFFNLVSEKVVSSIYNLLSVVLPPSPKKGKPKIKK
SS PSIPRED         EEEEEE     HHHHHHHHHHHHH    HHHHHHHHHHHHHHHHHHHHHEEE
CONF              9588755044508999999999995566301136778689999999976336458998788877689
SS JNET             E EEE      HHHHHHHHHHHH       EEEEEEEEEHHHEEE  EEEE
CONF              7555421115602056677775053467643551176413677653030478703788896663078
SS PROF (Ouali)    EEEEEEE      HHHHHHHHHEEE     EEEEEEE HHHHHHHHH EEEEE
CONF              8578887656656476778876545456664334566434678877653457755678878887778
SS PROF (Rost)     EEEEEE   HHHHHHHHHHHHHHE        EEEEE HHHHHHHHHHHEEEE
CONF              8577740342057877777655302144421121433203323444422112404676667743368
TM HMMTOP         ------XXXXXXXXXXXXXXXXXXXX++++++++XXXXXXXXXXXXXXXXXXXX-------------
CONF
SP PREDISI        SSSSSSSSSSSSSSSSSSSSSSSSSSSSC
SP HMMTOP
TM PHOBIUS        +++++XXXXXXXXXXXXXXXXXXX-------------------------------------------
CONF
TM PROF (Rost)    +++++++++XXXXXXXXXXXXXXXXXXX----XXXXXXXXXXXXXXXXXX+++++++++++++++++
CONF              7877520367788888888888888877764023210000146777665431257889999999999
DO IUPRED                                                                         DD
CONF
```
